# Expanding the substrate scope of ugi five-center, four-component reaction U-5C-4CR): ketones as coupling partners for secondary amino acids

**DOI:** 10.1007/s11030-013-9488-0

**Published:** 2013-10-24

**Authors:** Maciej Dawidowski, Sławomir Sobczak, Marcin Wilczek, Artur Kulesza, Jadwiga Turło

**Affiliations:** 1 Department of Drug Technology and Pharmaceutical Biotechnology, Medical University of Warsaw, Banacha 1 Str., 02-097 Warsaw, Poland; 2 Medical University of Warsaw, Żwirki i Wigury 61 Str., 02-091 Warsaw, Poland; 3 Laboratory of NMR Spectroscopy, University of Warsaw, Pasteura 1 Str., 02-093 Warsaw, Poland; 4 Faculty of Chemistry, University of Warsaw, Pasteura 1 Str., 02-093 Warsaw, Poland

**Keywords:** Multicomponent reactions, Ugi reaction, Molecular diversity, Diastereoselectivity, Isocyanides

## Abstract

Various symmetrical and unsymmetrical ketones were successfully coupled with secondary amino acids in the course of Ugi five-center, four-component reaction (U-5C-4CR), thus expanding the molecular diversity possible to be achieved by the reaction. The chemical yields depended on the degree of hindrance of the components employed and were satisfactory in view of possible steric interactions in the U-5C-4CR zwitterionic intermediate. The sense of diastereoinduction for reactions employing unsymmetrical ketones was examined by converting the resulting Ugi adducts into the corresponding rigid 2,6-diketopiperazine derivatives.

**Electronic supplementary material:**

The online version of this article (doi:10.1007/s11030-013-9488-0) contains supplementary material, which is available to authorized users.

## Introduction

**Scheme 1:**
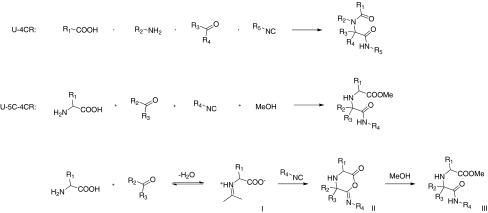
Ugi U-4CR and U-5C-4CR

Over the past several decades, multicomponent reactions (MRCs) have become attractive tools in modern synthetic organic chemistry. Among their many advantages, they allow the creation of large chemical libraries of diverse, complex molecular structures, starting from simple materials within a short time frame. Not surprisingly, these particular features have made MCRs especially appealing to medicinal chemists [1–6]. The well-known Ugi four-component reaction (U-4CR, Scheme 1) is one of the most widely used isocyanide-based multicomponent reactions (IMCRs). The classical variant of U-4CR comprises a one-pot sequential condensation of an amine, a carbonyl compound, an isocyanide and a carboxylic acid, to produce a linear, peptide-like adduct with high yield and high atom economy [7–9]. Since its discovery in 1959, the U-4CR has received growing attention for its potential to quickly assemble complex molecules. Initially, use of U-4CR in this capacity was restricted by limited availability of various isocyanide components. Since then, these components, including the so-called ‘convertible’ isocyanides [10–13], have become readily available, expanding the molecular diversity that can be achieved using the reaction.

Numerous variants and post-condensation modifications of the original U-4CR have emerged [14–18]. One of them is an Ugi five-center four-component reaction (U-5C-4CR, Scheme 1), which differs from the parent U-4CR not only in the number of reacting functional groups, but also in its mechanism. It is based on the condensation of a carbonyl compound, an isocyanide, a nucleophile and an amino acid as a bifunctional component. The reaction is initiated by the reversible formation of the zwitterionic imine **I** from the amino acid and carbonyl components. The subsequent addition of the isocyanide and intramolecular addition of the carboxylate give the cyclic intermediate **II**, which undergoes irreversible nucleophilic attack to form the 1,1$^{\prime }$-iminodicarboxylic acid derivative **III**. It is important to note that reaction usually proceeds with high diastereoselectivity if a chiral amino acid is used as an input [19].

Despite its high potential to generate interesting adducts for medicinal chemistry purposes, U-5C-4CR variant has received less attention when compared to parent U-4CR. The reaction has been applied to $\upalpha$- and $\upbeta$-amino acids, aldehydes, ketones, isocyanides and simple alcohols [19–25]. However, while ketones have been reported to react successfully with primary amino acids in the course of the U-5C-4CR and related Ugi five-center three component reaction (U-5C-3CR) [21, 23, 26, 27], their condensation with secondary amino acids has not been explored. Arguably, these coupling partners can be regarded as insufficiently reactive because of possible steric interactions in the initial imine zwitterionic intermediate of the postulated reaction mechanism (Scheme 1). Further, conversely to the condensations of primary amines or amino acids, in case of secondary amino acids the imine intermediates can not be preformed.

Recently, we described the application of U-5C-4CR adducts derived from aldehydes and secondary amino acids as intermediates for biologically active 2,6-diketopiperazine (2,6-DKP) derivatives [28]. Encouraged by the wide substrate scope of U-5C-4CR encountered, we decided to investigate the possible use of various aliphatic ketones as carbonyl inputs for the condensations with secondary amino acids. Since U-5C-4CR of an enantiopure amino acid and an unsymmetrical ketone proceeds with the formation of a new stereogenic centre, we investigated the degree and the sense of diastereoinduction.

## Results and discussion

We chose L-proline, acetone, *tert-*butyl isocyanide and methanol as model inputs for preliminary experiments (Table 1). Propitiously, the Ugi product **1a** formed with an acceptable yield, after 1 day, at room temperature, without use of any catalyst (Entry 1). When the reaction time was prolonged to 3 days (Entry 2), the yield improved, indicating that U-5C-4CRs of secondary amino acids with ketones might require longer completion times than analogous processes for aldehydes. The reaction was inhibited by addition of 1 eq. of an organic base (Entry 3), whereas a significant improvement of chemical yields was achieved with catalytic amounts of Lewis acids (Entries 4–5). $\text {TiCl}_{4}$ proved superior to $\text {FeCl}_{3}$ and was chosen for further studies. Neither increasing the time of the catalysed reaction to 5 days (Entry 6) nor performing the reaction at the lower $(-20\,^{\circ }\text {C})$ or higher $(50\, ^{\circ }\text {C})$ temperature (Entries 7–8) improved the reaction yield.

**Table 1:** Optimization of U-5C-4CRconditions
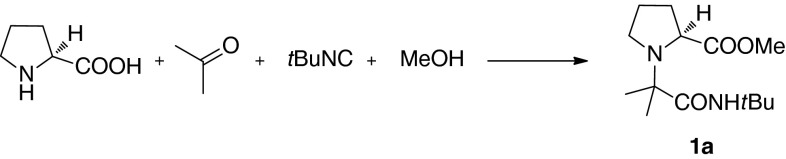

Entry$^\mathrm{a}$	Additive	Temperature	Time (days)	Conversion (%)$^\mathrm{b}$
1	–	rt	1	30
2	–	rt	3	39
3	$\text {TEA}^\mathrm{c}$	rt	3	12
4	$\text {FeCl}_{3}^\mathrm{d}$	rt	3	53
5	$\text {TiCl}_{4}^\mathrm{d}$	rt	3	67
6	$\text {TiCl}_{4}^\mathrm{d}$	rt	5	65
7	$\text {TiCl}_{4}^\mathrm{d}$	$-20\,^{\circ }\text {C}$	3	50
8	$\text {TiCl}_{4}^\mathrm{d}$	$50\,^{\circ }\text {C}$	3	60

$^\mathrm{a}$ Unless stated otherwise, the reactions were carried out in 0.1 M MeOH solutions on a 0.2 mmol scale. $^\mathrm{b}$ Estimated by HPLC. $^\mathrm{c}$ 1.0 eq. $^\mathrm{d}$ 5 mol%

**Fig. 1:**
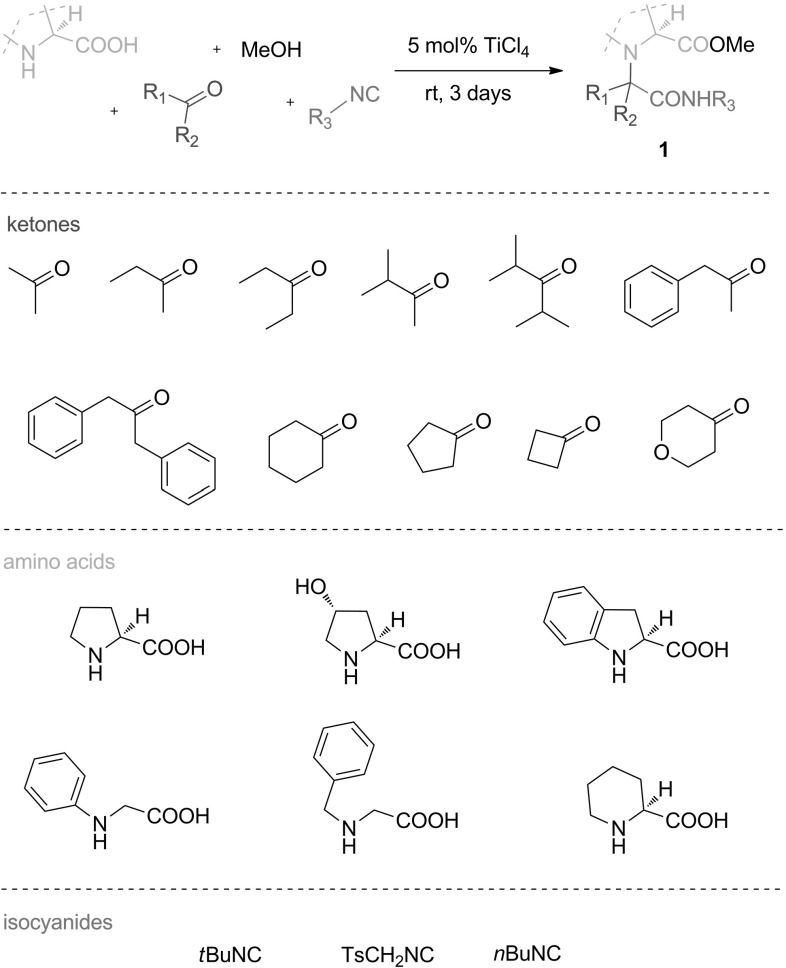
Ketones, secondary amino acids and isocyanides used in the U-5C-4CR

With the optimized reaction conditions in hand (Table 1, Entry 5), we initiated investigations of the substrate scope and limitations of the U-5C-4CR of secondary amino acids and ketones (Fig. 1).

**Fig. 2:**
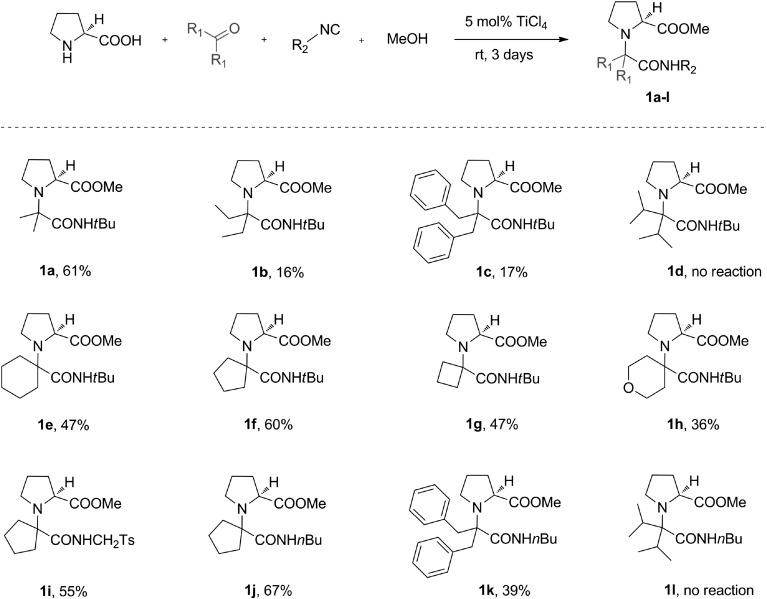
U-5C-4CR of L-proline, various symmetrical ketones, isocyanides and methanol. Reactions were carried out in 0.2 M MeOH solutions on a 4.2–8.4 mmol scale. All yields are isolated yields

We initially examined symmetrical ketones as coupling partners for L-proline, *tert*-butyl isocyanide and methanol (Fig. 2). Chemical yields ranged from 0 to 61 % and were largely dependent on the steric properties of the ketones employed. The highest yield was achieved for the derivative of the least bulky acetone, **1a**; however, when the aliphatic side chains of the ketone were expanded with methyl groups in **1b**, a markedly lower yield was observed. Given these results, we were surprised to see no further drop of yield when using more sterically hindered dibenzyl ketone for **1c**. No desired product was formed when $\upalpha$-branched diisopropyl ketone was used as a substrate.^1^

**Fig. 3:**
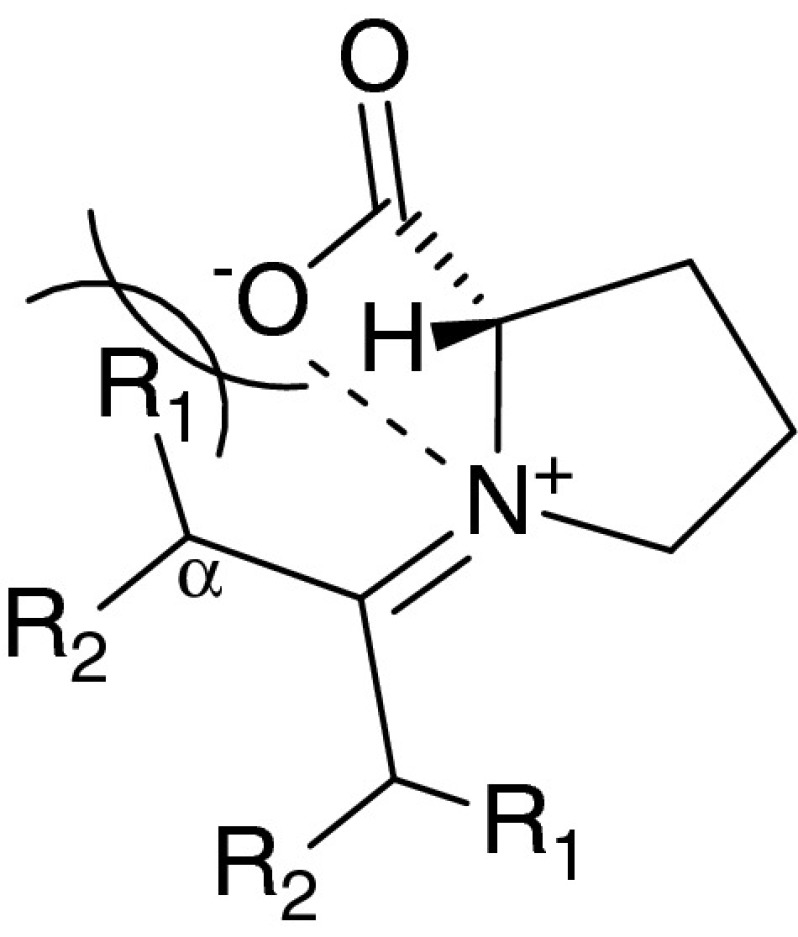
Possible unfavourable steric interaction within the U-5C-4CR zwitterionic intermediate

The results obtained for **1a**–**d** indicated that the outcome of U-5C-4CR is determined by the degree of steric hindrance around the $\upalpha$-carbon of the ketone. This finding could be explained by unfavourable steric interactions of the alkyl groups of the carbonyl component with the carboxylate in the zwitterionic imine intermediate (Fig. 3). These interactions were markedly more pronounced for $\upalpha$ branched ketones. In agreement with this elucidation, less conformationally strained cyclic ketones gave adducts **1e**–**h** with chemical yields comparable to those obtained for acetone.

Aware of the possible unfavourable steric interactions in the U-5C-4CR intermediate, we next investigated the influence of the steric hindrance of the isocyanide component on the outcome of the condensation (Fig. 2, adducts **1i**–**l**). A decrease in the steric hindrance of the isocyanide had an enhancing effect only when bulky ketones were employed as substrates. Thus, no significant enhancement was observed when *tert*-butyl isocyanide was replaced by tosylmethyl (product **1i**) or $n$-butyl (product **1j**) isocyanide as a coupling partner for unbulky cyclopentanone. On the contrary, when the more bulky and flexible dibenzyl ketone was reacted with $n$-butyl isocyanide (product **1k**), the chemical yield increased more than twofold compared with the analogous reaction for **1c**. Again, no adduct was formed when diisopropyl ketone was used as a substrate.

**Fig. 4:**
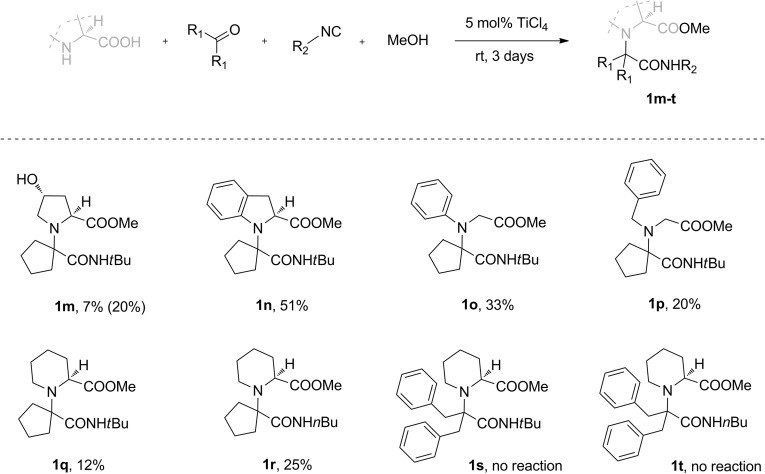
U-5C-4CR of various secondary amino acids, symmetrical ketones, isocyanides and methanol. Reactions were carried out in 0.2 M solutions on a 4.2–8.4 mmol scale. All yields are isolated yields

Other secondary amino acids were coupled with symmetrical ketones producing adducts **1m**–**t**, with chemical yields ranging from 0 to 51 % (Fig. 4). *Trans*-hydroxy-L-proline condensed with cyclopentanone, *tert-*butyl isocyanide and methanol to form **1m** with significantly lower yield than an analogous reaction for L-proline. This was due to the poor solubility of the amino acid in methanol. Higher yield was obtained when the solvent was changed to DMF/MeOH 1:1 (v/v). The amino acids containing aromatic amino groups, $(S)$-indolinecarboxylic acid and *N*-phenylglycine gave products **1n** and **1o**, respectively, with good yield. Surprisingly, the efficiency of the U-5C-4CR of L-pipecolic acid with cyclopentanone and *tert*-butyl isocyanide (product **1q**) was significantly lower than that for homologous L-proline (Fig. 2, product **1f**). When the hindered isocyanide was replaced with its linear isomer, a twofold increase in yield of coupling adduct **1r** was observed. Contrary to what had been observed with the formation of L-proline derivatives **1c** and **1k** (Fig. 2), the reactions of L-pipecolic acid with bulky dibenzyl ketone and either *tert*-butyl or $n$-butyl isocyanide failed to proceed. This significant loss of reactivity could be ascribed to the greater steric bulk or increased conformational flexibility of this amino acid when compared with homologous L-proline.

**Fig. 5:**
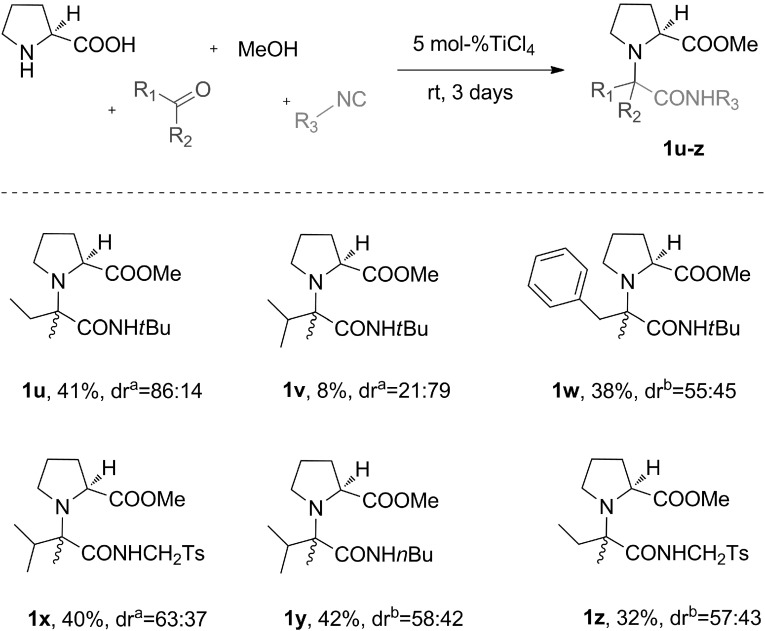
U-5C-4CR of L-proline, various unsymmetrical ketones, isocyanides and methanol. Reactions were carried out in 0.2 M MeOH solutions on a 4.2–8.4 mmol scale. With the exception of **1u**, the diastereomeric mixtures were resolved by column chromatography. All yields are isolated yields. $^\mathrm{a}$The diastereomeric ratios of $(2S,1R)/(2S,1S)$, estimated by LC/MS of the crude reaction mixtures. $^\mathrm{b}$The diastereomeric ratios of major/minor, estimated by LC/MS of the crude reaction mixtures

Once the substrate scope and limitations of U-5C-4CR of secondary amino acids and symmetrical ketones had been determined, we turned our attention to analogous condensations of prochiral, unsymmetrical ketones (Fig. 5). Initially, L-proline and *tert*-butyl isocyanide were used as coupling partners for 2-butanone, 3-methyl-2-butanone and 1-phenyl-2-propanone. As expected, the chemical yields of the corresponding adducts **1u**, **1v** and **1w** were markedly higher than those for the derivatives of similar symmetrical ketones (Fig. 2, compounds **1b**, **1d** and **1c**, respectively). Nonetheless, the same trend in reactivity was observed. The lowest yield was obtained for the 3-methyl-2-butanone adduct **1v**, incorporating branched isopropyl chain. The yields of the condensation products of the remaining two ketones, 2-butanone and 1-phenyl-2-propanone (compounds **1u** and **1w**, respectively), were roughly equal. Similar to what had been observed for symmetrical ketones, changing *tert*-butyl isocyanide to a less bulky tosylmethyl or $n$-butyl isocyanide greatly enhanced formation of derivatives of the hindered 3-methyl-2-butanone (Fig. 5, compounds **1x** and **1v)**.

The degree of diastereoinduction obtained for adducts **1u**–**z** depended largely on the structure of the isocyanide input. The relatively high diastereoselectivity was observed for derivatives of hindered *tert*-butyl isocyanide **1u** and **1v**. Surprisingly, almost equal amounts of diastereoisomers were formed for **1w**. When the isocyanide was changed to a less bulky tosylmethyl or $n$-butyl isocyanide, adducts **1x**, **1y** and **1z** were formed with only modest or trace diastereoinduction.

We subsequently investigated the sense of diastereoinduction for products **1u**, **1v** and **1x**. In accordance with the Ugi’s pioneering report, the sense of diastereoinduction of U-5C-4CR of amino acids and aldehydes is $(S,S)$ [19], which was also confirmed by us and others [20, 28]. However, some reports have observed opposite diastereoselectivity [22, 24, 26, 27], indicating that the stereochemical outcomes of U-5C-4CR may be highly sensitive to the nature of coupling reagents and reaction conditions.

**Scheme 2:**
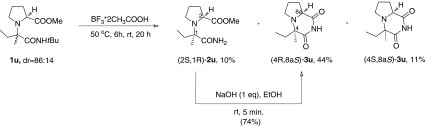
Synthesis of cyclic derivatives $(4R,8\text {a}S)$-**3u** and $(4S,8\text {a}S)$-**3u**

**Scheme 3:**
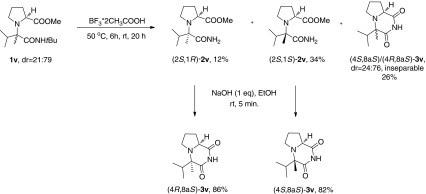
Synthesis of cyclic derivatives $(4R,8\text {a}S)$-**3v** and $(4S,8\text {a}S)$-**3v**

**Scheme 4:**
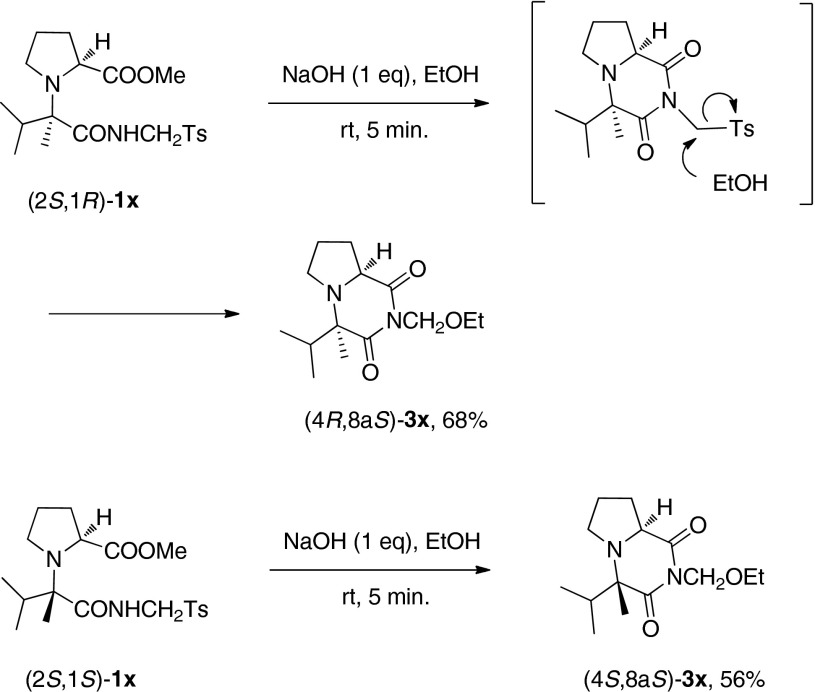
Synthesis of cyclic derivatives $(4R,8\text {a}S)$-**3x** and $(4S,8\text {a}S)$-**3x**

Since the rotation around the N-C$^{1}$ bond of the products **1** was unrestricted, the direct NOE measurements could not be considered diagnostic for determination of the relative configurations. Therefore, **1u**, **1v** and **1x** 2 were converted into the corresponding rigid, cyclic derivatives **3u**, **3v **and **3x** (Schemes 2, 3 and 4, respectively).

**Fig. 6:**
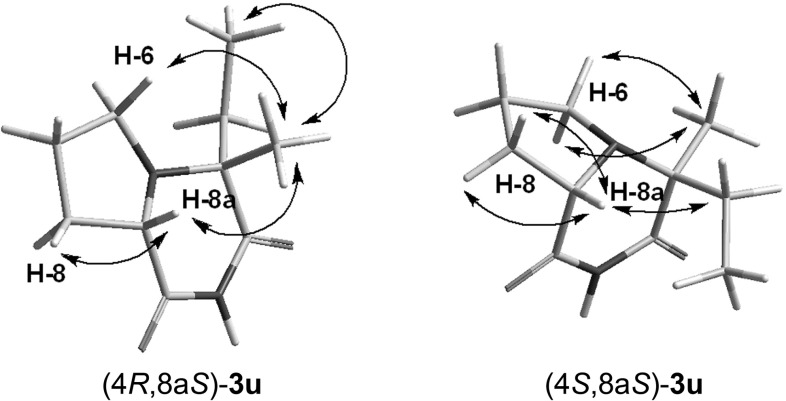
Selected NOESY correlations in $(4R,8\text {a}S)$-**3u** and $(4S,8\text {a}S)$-**3u**

Direct base-induced cyclocondensation of **1u** and **1v** to their corresponding N-*tert*-butylated imides failed. Therefore, N-de*tert*-butylation/cyclocondensation sequence leading to N-unsubstituted cyclic derivatives was chosen as an alternative pathway (Schemes 2 and 3, respectively). The diastereomeric mixture of **1u** was treated with $\text {BF}_{3}*2\text {CH}_{3}\text {COOH}$ at $50\,^{\circ }\text {C}$ to afford a chromatographically separable mixture of N-de*tert*-butylated amidoester $(2S,1R)$-**2u** 3 (10 %) and cyclic imides $(4R,8\text {a}S)$-**3u **(44 %), $(4S,8\text {a}S)$-**3u **(11 %). Subsequently, $(2S,1R)$-**2u** was converted to $(4R,8\text {a}S)$-**3u** upon treatment with base. The stereochemistries of $(4R,8\text {a}S)$-**3u** and $(4S,8\text {a}S)$-**3u** were determined by the observation of characteristic correlations in their NOESY spectra (Fig. 6). Another spectroscopic feature which was useful for distinguishing between the respective diastereoisomers of **3u** was the characteristic difference in the chemical shifts and shapes of multiplets of protons H-8a, H-6 and H$'$-6. The above results indicated that the sense of diastereoinduction for the formation of **1u** was $(2S,1R)$.

Unlike **1u**, which produced the desired, separable cyclic derivatives **3u** directly upon treatment with $\text {BF}_{3}*2\text {CH}_{3}\text {COOH}$ at $50\,^{\circ }\text {C}$, the diastereomeric mixture of **1v** gave $(2S,1R)$-**2v** (12 %), $(2S,1S)$-**2v** (34 %) and a chromatographically inseparable diastereomeric mixture of $(4R,8\text {a}S)$-**3v** and $(4S,8\text {a}S)$-**3v** (dr=30/70, $^{1}\text {H}$ NMR). However, the resulting amidoesters $(2S,1R)$-**2v** and $(2S,1S)$-**2v** could be chromatographically separated. They were subsequently treated with 1 eq. of NaOH to afford $(4R,8\text {a}S)$-**3v **and $(4S,8\text {a}S)$-**3v**, with chemical yields of 86 and 82 %, respectively. Relative configurations of the diastereomers of **3v** were assigned by comparing their $^{1}\text {H}$ NMR spectra to those of $(4R,8\text {a}S)$-**3u** and $(4S,8\text {a}S)$-**3u**. Surprisingly, the sense of diastereoinduction for the formation of **1v** was reversed compared to **1u**.

**Scheme 5:**
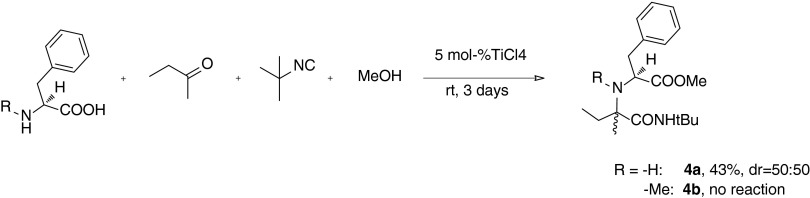
Synthesis of L-phenylalanine derivatives **4a** and **4b**. The diastereomeric ratios of major/minor were estimated by LC/MS analysis of the crude reaction mixture

In contrast to **1u** and **1v**, for which the base-induced cyclization failed, the respective diastereomers of **1x** could be converted to the corresponding cyclic imides $(4R,8\text {a}S)$-**3x** and $(4S,8\text {a}S)$-**3x** directly upon treatment with 1 eq. NaOH in EtOH. The reaction was accompanied by the tosyl-ethoxyl exchange, which was in agreement with earlier observations made by van Leusen et al during their investigations of tosylmethyl isocyanide derivatives [29]. The configurations of $(4R,8\text {a}S)$-**3x** and $(4S,8\text {a}S)$-**3x** were assigned by analysis of characteristic patterns of their $^{1}\text {H}$ NMR spectra. In view of the above, $(2S,1R)$-**1x** was the major isomer in the post reaction mixture, contrary to what had been assigned for **1v**.

The observed degree and sense of diastereoinduction observed for the formation of compounds **1u**, **1v**, **1w** and **1x** was not always consistent and could not be easily rationalized. In general, the stereochemical outcomes depended on the structure of both the isocyanide and the ketone inputs. Furthermore, the interactions of these two components with the rigid L-proline framework cannot be neglected. This was evaluated by the results of an additional experiment in which no diastereoselectivity was observed in the U-5C-4CR of bulky *tert*-butyl isocyanide, 2-butanone and conformationally flexible primary amino acid, L-phenylalanine (Scheme 5). Surprisingly, its secondary analogue, *N*-methyl-L-phenylalanine, failed to react under the same conditions.

## Conclusions

Experimental results show that various symmetrical and unsymmetrical ketones can be successfully used as coupling partners for secondary amino acids in the course of the U-5-C-4CR. Outcomes of these condensations seemed to depend on the hindrance and the components employed. Even very subtle differences in coupling reagents caused significant changes of both yields and stereochemical outcomes. In most cases, the chemical yields were satisfactory in view of possible steric interactions in the U-5C-4CR zwitterionic intermediate. When unsymmetrical ketones were used as prochiral carbonyl inputs for coupling with bulky *tert*-butyl isocyanide, high diastereoselectivities were usually observed. Although the sense of diastereoinduction was not consistent and could not be easily rationalized, we believe that some more general conclusions could be drawn from adequately designed experiments combined with computational methods. Therefore, in our opinion the presented variation of components for U-5C-4CR is not only capable of expanding the molecular diversity generated by the reaction, but can also serve as a potential basis for investigations of its mechanism and diastereoselectivity, which still remain a subject of a debate.

## Experimental section

### General

The NMR spectra were obtained on a Varian VNMRS 300 MHz or Varian Inova 500 MHz spectrometer at room temperature. Chemical shifts ($\delta$) were expressed in parts per million (ppm) relative to tetramethylsilane (TMS) or residual solvent peaks used as the internal references. The following abbreviations were used to describe the signal patterns: s (singlet), d (doublet), t (triplet), q (quartet), m (multiplet), p (pseudo-) and b (broad-). Coupling constants $(J)$ were in hertz (Hz). The FT-IR spectra (thin film on KBr pellets) were recorded on a Shimadzu FT-IR8300 instrument. High-resolution mass spectra (HRMS) were obtained using a LCT-TOF (Micromass) spectrometer with electrospray ionization (ESI). Optical rotations were measured with a Perkin-Elmer 241 polarimeter using a sodium lamp (589 nm). Melting points were determined on an Electrothermal 9100 apparatus in open capillary tubes and are uncorrected. LC/MS analyses were performed using a Shimadzu Nexera UHPLC system with LCMS-2020 single quadrupole spectrometer equipped with an ESI ion source. Supelcosil LC-18-DB column (length: 25 mm, internal diameter: 4.6 mm, particle size: 5 $\upmu$m) was used. The analyte concentration: approximately 50 ng/mL, injection: 5 $\upmu$L, flow rate 0.4 mL/min, temperature: $60\,^{\circ }\text {C}$, mobile phase: acetonitrile/water 60:40 (v/v). Thin-layer chromatography (TLC) was run on Merck silica gel (60-$\text {F}_{254})$ plates. The spots were visualized by ultraviolet light (254 nm) or iodine vapors. Flash column chromatography (FC) was carried out on silica gel 60 (particle size: 0.040–0.063 mm).

### General procedure for the U-5C-4CR condensation

To a stirred solution of an $\upalpha$-amino acid (1.2 eq.) and ketone (1.0 eq.) in MeOH (5 mL per 1 mmol of ketone) titanium (IV) chloride (5 mol%) was added, followed by isocyanide (1.0 eq.). The mixture was stirred at room temperature for 3 days and the volatiles were removed under reduced pressure. The resulting crude products were purified by FC.

#### Methyl $(2S)$-1-(1-(*tert*-butylcarbamoyl)-1-methyl-1-ethyl)-pyrrolidine-2-carboxylate **1a**

From L-proline (1.16 g, 10.08 mmol), acetone (0.62 mL, 8.40 mmol) and *tert-*butyl isocyanide (0.95 mL, 8.40 mmol); FC (gradient: PE/AcOEt 9:1 to 3:1): yield 1.39 g (61 %). White wax, M.p. 45–$46\,^{\circ }\text {C}$; TLC (PE/AcOEt 5:1): $\text {R}_\mathrm{f}=0.29$; $[\upalpha ]_\mathrm{D}=-34.7\,(c 1, \text {CHCl}_{3})$; IR (KBr): 1200, 1454, 1516, 1678, 1744, 2970, 3348; $^{1}\text {H}$ NMR $(\text {CDCl}_{3}$, 500 MHz): $\delta$ 1.16 (s, 3H, $\text {C}H_{3})$, 1.21 (s, 3H, $\text {C}H_{3})$, 1.32 (s, 9H, C$(\text {C}H_{3})_{3})$, 1.74–1.85 (m, 2H, H-4, H$'$-4), 1.87 (m, 1H, H-3), 1.99 (m, 1H, H$'$-3), 2.69 (m, 1H, H-5), 2.97 (m, 1H, H$'$-5), 3.61 (dd, $^{3}J_{1}=9.5,^{3}J_{2}=2.5$, 1H, H-2), 3.71 (s, 3H, $\text {OC}H_{3})$, 7.44 (bs, 1H, $\text {CON}H)$; $^{13}\text {C}$ NMR $(\text {CDCl}_{3}$, 125 MHz): $\delta$ 21.7 $(C\text {H}_{3})$, 22.3 $(C\text {H}_{3})$, 24.5 (C-4), 29.0 $(\text {C}(C\text {H}_{3})_{3})$, 32.3 (C-3), 48.6 (C-5), 50.6 $(C(\text {CH}_{3})_{3})$, 52.4 $(\text {O}C\text {H}_{3})$, 60.1 (C-2), 63.2 (C-1), 176.4 $(C\text {ONH})$, 177.7 $(C\text {OOCH}_{3})$; HRMS (ESI+) calcd for $\text {C}_{14}\text {H}_{26}\text {N}_{2}\text {O}_{3}$Na: 293.1841 (M+Na)$^{+}$ found 293.1823.

#### Methyl $(2S)$-1-(1-(*tert*-butylcarbamoyl)-1-ethyl-1-propyl)-pyrrolidine-2-carboxylate **1b**

From L-proline (1.16 g, 10.08 mmol), 3-pentanone (0.89 mL, 8.40 mmol) and *tert-*butyl isocyanide (0.95 mL, 8.40 mmol); FC (gradient: PE/AcOEt 10:1 to 4:1): yield 0.40 g (16 %). White wax; M.p. 47–$48\,^{\circ }\text {C}$; TLC (PE/AcOEt 5:1): $R_\mathrm{f}=0.42$; $[\alpha ]_\mathrm{D}=-2.5$ ($c$ 0.950, $\text {CHCl}_{3})$; IR (KBr): 667, 769, 1198, 1455, 1520, 1674, 1735, 2880, 2966, 3316; $^{1}\text {H}$ NMR $(\text {CDCl}_{3}$, 300 MHz): $\delta$ 0.74 (t, $^{3}J=7.5$, 3H, $\text {CH}_{2}\text {C}H_{3})$, $\delta$ 0.83 (t, $^{3}J=7.5$, 3H, $\text {CH}_{2}\text {C}\text {H}_{3}^{{\prime }})$, 1.35 (s, 9H, C$(\text {C}H_{3})_{3})$, 1.47–1.94 (m, 8H, 2xC$H_{2}$, H-3, H$'$-3, H-4, H$'$-4), 2.10 (m, 1H, H-5), 2.74 (m, 1H, H$'$-5), 3.73 (s, 3H, $\text {OC}H_{3})$, 4.09 (dd, $^{3}J_{1}=9.0$,$^{3}J_{2}=1.0$, 1H, H-2), 8.14 (bs, 1H, $\text {CON}H)$; $^{13}\text {C}$ NMR $(\text {CDCl}_{3}$, 75 MHz): $\delta$ 7.3, 9.3 (2x$C\text {H}_{3})$, 20.9, 24.3, 26.9, 28.9 $(\text {C}(C\text {H}_{3})_{3})$, 31.8 (C-3), 47.4 (C-5), 50.2 $(C(\text {CH}_{3})_{3})$, 52.3 $(\text {O}C\text {H}_{3})$, 59.2 (C-2), 66.7 (C-1), 175.7 $(C\text {ONH})$, 179.1 $(C\text {OOCH}_{3})$; HRMS (ESI+) calcd for $\text {C}_{16}\text {H}_{30}\text {N}_{2}\text {O}_{3}\text {Na}$: 321.2154 (M+Na)$^{+}$ found 321.2151.

#### Methyl $(2S)$-1-(1-(*tert*-butylcarbamoyl)-1-benzyl-3-phenyl-1-ethyl)-pyrrolidine-2-carboxylate **1c**

From L-proline (1.16 g, 10.08 mmol), diphenyl-2-propanone (1.77 g, 8.40 mmol) and *tert-*butyl isocyanide (0.95 mL, 8.40 mmol); FC (gradient: PE/AcOEt 12:1 to 8:1): yield 0.60 g (17 %). Yellow oil; TLC (PE/AcOEt 5:1): $R_\mathrm{f}=0.48$; $[\alpha ]_\mathrm{D}=-46.2$ ($c$ 0.792, $\text {CHCl}_{3})$; IR (KBr): 668, 669, 745, 1212, 1455, 1514, 1679, 1734, 2872, 2963, 3313; $^{1}\text {H}$ NMR $(\text {CDCl}_{3}$, 300 MHz): $\delta$ 1.30 (s, 9H, $\text {C}(\text {C}H_{3})_{3})$, 1.70–1.86 (m, 4H, H-3, H$'$-3, H-4, H$'$-4), 2.87 (d, $^{2}J=13.5$, 1H, $\text {C}H_{2})$, 3.07 (m, 2H, H-5, $\text {C}H_{2})$, 3.09–3.22 (m, 3H, H$'$-5, 2x$\text {C}H_{2}^{{\prime }})$, 3.64 (s, 3H, $\text {OC}H_{3})$, 3.67 (m, 1H, H-2), 7.13–7.38 (m, 10H, H-Ar), 7.66 (bs, 1H, $\text {CON}H)$; $^{13}\text {C}$ NMR $(\text {CDCl}_{3}$, 75 MHz): $\delta$ 23.8 (C-4), 28.5 $(\text {C}(C\text {H}_{3})_{3})$, 31.7 (C-3), 37.1 $(C\text {H}_{2})$, 40.6 $(C\text {H}_{2}^{{\prime }})$, 47.9 (C-5), 50.5 $(C(\text {CH}_{3})_{3})$, 52.0 $(\text {O}C\text {H}_{3})$, 59.9 (C-2), 68.9 (C-1), 126.5, 126.7, 127.9, 128.0, 130.79, 130.83, 136.9, 138.2 (C-Ar), 173.5 $(C\text {ONH})$, 178.7 $(C\text {OOCH}_{3})$; HRMS (ESI+) calcd for $\text {C}_{26}\text {H}_{34}\text {N}_{2}\text {O}_{3}\text {Na}$: 445.2467 (M+Na)$^{+}$ found 445.2451.

#### Methyl $(2S)$-1-(1-(*tert*-butylcarbamoyl)-cyclohexyl)-pyrrolidine-2-carboxylate **1e**

From L-proline (1.16 g, 10.08 mmol), cyclohexanone (0.87 mL, 8.40 mmol) and *tert-*butyl isocyanide (0.95 mL, 8.40 mmol); FC (gradient: PE/AcOEt 10:1 to 3:1): yield 1.23 g (47 %). Pale-yellow oil; TLC (PE/AcOEt 5:1): $R_\mathrm{f}=0.49$; $[\alpha ]_\mathrm{D}=-15.0$ ($c$ 0.613, $\text {CHCl}_{3})$; IR (KBr): 668, 766, 1199, 1455, 1510, 1679, 1738, 2863, 2933, 3331; $^{1}\text {H}$ NMR $(\text {CDCl}_{3}$, 300 MHz): $\delta$ 1.34 (s, 9H, $\text {C}(\text {C}H_{3})_{3})$, 1.44–1.68 (m, 6H), 1.68–1.91 (m, 6H), 1.91–2.06 (m, 2H), 2.81 (m, $^{2}J=^{3}J_{1}=8.5$,$^{3}J_{2}=6.0$, 1H, H-5), 2.91 (m, 1H, H$'$-5), 3.70 (s, 3H, $\text {OC}H_{3})$, 3.78 (dd, $^{3}J_{1}=9.0$,$^{3}J_{2}=2.5$, 1H, H-2), 7.13 (bs, 1H, $\text {CON}H)$; $^{13}\text {C}$ NMR $(\text {CDCl}_{3}$, 75 MHz): $\delta$ 23.4, 23.6, 25.1, 26.3, 29.1 $(\text {C}(C\text {H}_{3})_{3})$, 30.3, 32.3, 33.6, 47.7 (C-5), 50.6 $(C(\text {CH}_{3})_{3})$, 52.4 $(\text {O}C\text {H}_{3})$, 59.1 (C-2), 64.8 (C-1), 175.8 $(C\text {ONH})$, 178.2 $(C\text {OOCH}_{3})$; HRMS (ESI+) calcd for $\text {C}_{17}\text {H}_{30}\text {N}_{2}\text {O}_{3}\text {Na}$: 333.2154 (M+Na)$^{+}$ found 333.2148.

#### Methyl $(2S)$-1-(1-(*tert*-butylcarbamoyl)-cyclopentyl)-pyrrolidine-2-carboxylate **1f**

From L-proline (1.16 g, 10.08 mmol), cyclopentanone (0.74 mL, 8.40 mmol) and *tert-*butyl isocyanide (0.95 mL, 8.40 mmol); FC (gradient: PE/AcOEt 10:1 to 3:1): yield 1.50 g (60 %). Colorless oil; TLC (PE/AcOEt 5:1): $R_\mathrm{f}=0.41$; $[\alpha ]_\mathrm{D}=-12.0$ ($c$ 0.325, $\text {CHCl}_{3})$; IR (KBr): 768, 1199, 1456, 1509, 1681, 1737, 2875, 2966, 3368; $^{1}\text {H}$ NMR $(\text {CDCl}_{3}$, 300 MHz): $\delta$ 1.34 (s, 9H, $\text {C}(\text {C}H_{3})_{3})$, 1.45–2.22 (m, 12H), 2.66 (m, $^{2}J=^{3}J_{1}=8.5$,$^{3}J_{2}=7.0$, 1H, H-5), 3.03 (m, 1H, H$'$-5), 3.61 (dd, $^{3}J_{1}=$9.5,$^{3}J_{2}=2.5$, 1H, H-2), 3.73 (s, 3H, $\text {OC}H_{3})$, 7.58 (bs, 1H, $\text {CON}H)$; $^{13}\text {C}$ NMR $(\text {CDCl}_{3}$, 75 MHz): $\delta$ 24.8, 25.5, 25.7, 28.8 $(\text {C}(C\text {H}_{3})_{3})$, 32.0, 32.4, 34.9, 49.3 (C-5), 50.2 $(C(\text {CH}_{3})_{3})$, 52.2 $(\text {O}C\text {H}_{3})$, 60.5 (C-2), 73.8 (C-1), 176.3 $(C\text {ONH})$, 177.5 $(C\text {OOCH}_{3})$; HRMS (ESI+) calcd for $\text {C}_{16}\text {H}_{28}\text {N}_{2}\text {O}_{3}\text {Na}$: 319.1998 (M+Na)$^{+}$ found 319.1995.

#### Methyl $(2S)$-1-(1-(*tert*-butylcarbamoyl)-cyclobutyl)-pyrrolidine-2-carboxylate **1g**

From L-proline (1.16 g, 10.08 mmol), cyclobutanone (0.63 mL, 8.40 mmol) and *tert-*butyl isocyanide (0.95 mL, 8.40 mmol); FC (gradient: PE/AcOEt 10:1 to 3:1): yield 1.12 g (47 %). Yellow oil; TLC (PE/AcOEt 5:1): $R_\mathrm{f}=0.33$; $[\alpha ]_\mathrm{D}=+4.4$ ($c$ 0.607, $\text {CHCl}_{3})$; IR (KBr): 765, 1202, 1457, 1514, 1680, 1739, 2871, 2959, 3335; $^{1}\text {H}$ NMR $(\text {CDCl}_{3}$, 300 MHz): $\delta$ 1.35 (s, 9H, $\text {C}(\text {C}H_{3})_{3})$, 1.66 (m, 1H), 1.74–2.24 (m, 8H), 2.47 (m, 1H), 2.76 (m, $^{2}J=^{3}J_{1}=8.5$,$^{3}J_{2}=7.0$, 1H, H-5), 2.90 (m, 1H, H$'$-5), 3.71 (m, 4H, H-2, $\text {OC}H_{3})$, 7.42 (bs, 1H, $\text {CON}H)$; $^{13}\text {C}$ NMR $(\text {CDCl}_{3}$, 75 MHz): $\delta$ 14.4, 24.9, 25.8, 28.9 $(\text {C}(C\text {H}_{3})_{3})$, 30.2, 31.5, 48.3 (C-5), 50.2 $(C(\text {CH}_{3})_{3})$, 52.2 $(\text {O}C\text {H}_{3})$, 60.2 (C-2), 66.6 (C-1), 175.2 $(C\text {ONH})$, 176.8 $(C\text {OOCH}_{3})$; HRMS (ESI+) calcd for $\text {C}_{19}\text {H}_{28}\text {N}_{2}\text {O}_{3}\text {Na}$: 305.1847 (M+Na)$^{+}$ found 305.1841.

#### Methyl $(2S)$-1-(4-(*tert*-butylcarbamoyl)-4-tetrahydropyranyl)-pyrrolidine-2-carboxylate **1h**

From L-proline (1.16 g, 10.08 mmol), 4-oxotetrahydropyran (0.78 mL, 8.40 mmol) and *tert-*butyl isocyanide (0.95 mL, 8.40 mmol); FC (gradient: PE/AcOEt 10:1 to 3:1): yield 0.94 g (36 %). White powder; M.p. 97–$99\,^{\circ }\text {C}$; TLC (PE/AcOEt 5:1): $R_\mathrm{f}=0.11$; $[\alpha ]_\mathrm{D}=-15.3$ ($c$ 0.945, $\text {CHCl}_{3})$; IR (KBr): 668, 760, 1202, 1455, 1512, 1677, 1739, 2869, 2959, 3360; $^{1}\text {H}$ NMR $(\text {CDCl}_{3}$, 300 MHz): $\delta$ 1.34 (s, 9H, $\text {C}(\text {C}H_{3})_{3})$, 1.56 (m,$^{2}J=13.5$, $^{3}J_{1}=11.5$,$^{3}J_{2}=6.0$, 1H), 1.70 (m, 2H), 1.78 (m, 2H), 1.87 (m, 1H), 1.92–2.07 (m, 2H), 2.79 (m, $^{2}J=^{3}J_{1}=8.5$,$^{3}J_{2}=7.5$, 1H, H-5), 2.91 (m, 1H, H$'$-5), 3.64 (m, 1H), 3.70 (s, 3H, $\text {OC}H_{3})$, 3.72–3.90 (m, 3H), 3.78 (td, $^{2}J=^{3}J_{1}=11.5$,$^{3}J_{2}=2.5$, 1H), 7.23 (bs, 1H, $\text {CON}H)$; $^{13}\text {C}$ NMR $(\text {CDCl}_{3}$, 75 MHz): $\delta$ 25.0, 29.1 $(\text {C}(C\text {H}_{3})_{3})$, 30.6, 32.1, 33.6, 47.4 (C-5), 50.7 $(C(\text {CH}_{3})_{3})$, 52.5 $(\text {O}C\text {H}_{3})$, 58.8 (C-2), 62.3 (C-1), 65.1, 66.1, 175.2 $(C\text {ONH})$, 177.8 $(C\text {OOCH}_{3})$; HRMS (ESI+) calcd for $\text {C}_{16}\text {H}_{28}\text {N}_{2}\text {O}_{4}\text {Na}$: 355.1947 (M+Na)$^{+}$ found 355.1952.

#### Methyl $(2S)$-1-(1-(tosylmethylcarbamoyl)-cyclopentyl)-pyrrolidine-2-carboxylate **1i**

From L-proline (1.16 g, 10.08 mmol), cyclopentanone (0.74 mL, 8.40 mmol) and tosylmethyl isocyanide (1.64 g, 8.40 mmol); FC (gradient: PE/AcOEt 5:1 to 1:1): yield 1.90 g (55 %). Pale-yellow oil; TLC (PE/AcOEt 3:1): $R_\mathrm{f}$=0.13; $[\alpha ]_\mathrm{D}=+4.1$ ($c$ 1.310, $\text {CHCl}_{3})$; IR (KBr): 566, 758, 1142, 1321, 1455, 1495, 1692, 1733, 2873, 2953, 3339; $^{1}\text {H}$ NMR $(\text {CDCl}_{3}$, 300 MHz): $\delta$ 1.36–1.69 (m, 6H), 1.70–1.97 (m, 4H), 2.04 (m, 2H), 2.43 (s, 3H, $\text {C}H_{3})$, 2.57 (m, $^{2}J=^{3}J_{1}=9.0$,$^{3}J_{2}=6.5$, 1H, H-5), 2.90 (m, 1H, H$'$-5), 3.61 (dd, $^{3}J_{1}=9.5$,$^{3}J_{2}=3.0$, 1H, H-2), 3.76 (s, 3H, $\text {OC}H_{3})$, 4.71 (d, $^{2}J=^{3}J=7.0$, 2H, $\text {C}H_{2})$, 7.32 (d, $^{3}J=8.0$, 2H, H-Ar), 7.80 (d, $^{3}J=8.0$, 2H, H-Ar), 8.63 (pt, $^{3}J=7.0$, 1H, $\text {CON}H)$; $^{13}\text {C}$ NMR $(\text {CDCl}_{3}$, 75 MHz): $\delta$ 21.9, 24.7, 24.9, 31.2, 31.7, 36.2, 49.3 (C-5), 52.5 $(\text {O}C\text {H}_{3})$, 59.9, 60.9, 72.9 (C-1), 129.0, 129.9, 134.8, 145.2 (4xC-Ar), 177.0 $(C\text {ONH})$, 177.4 $(C\text {OOCH}_{3})$; HRMS (ESI+) calcd for $\text {C}_{20}\text {H}_{28}\text {N}_{2}\text {O}_{5}\text {SNa}$: 431.1617 (M+Na)$^{+}$ found 431.1601.

#### Methyl $(2S)$-1-(1-($n$-butylcarbamoyl)-cyclopentyl)-pyrrolidine-2-carboxylate **1j**

From L-proline (0.58 g, 5.04 mmol), cyclopentanone (0.37 mL, 4.20 mmol) and $n$-butyl isocyanide (0.44 mL, 4.20 mmol); FC (gradient: PE/AcOEt 7:1 to 3:1): yield 0.83 g (67 %). Pale-yellow oil; TLC (PE/AcOEt 5:1): $R_\mathrm{f}=0.26$; $[\alpha ]_\mathrm{D}=+3.6$ ($c$ 0.875, $\text {CHCl}_{3})$; IR (KBr): 668, 760, 1199, 1457, 1518, 1676, 1737, 2871, 2956, 3339; $^{1}\text {H}$ NMR $(\text {CDCl}_{3}$, 300 MHz): $\delta$ 0.93 (t, $^{3}J=7.0$, 3H, $\text {C}H_{3})$, 1.36 (m, 2H), 1.43–1.68 (m, 6H), 1.68–2.13 (m, 7H), 2.21 (m, 1H), 2.65 (m, $^{2}J=^{3}J_{1}=9.0$,$^{3}J_{2}=7.0$, 1H, H-5), 2.99 (m, 2H, H$'$-5), 3.16 (m, 1H), 3.28 (m, 1H), 3.62 (dd, $^{3}J_{1}=9.5$,$^{3}J_{2}=2.5$, 1H, H-2), 3.71 (s, 3H, $\text {OC}H_{3})$, 7.69 (pt, $^{3}J=5.5$, 1H, $\text {CON}H)$; $^{13}\text {C}$ NMR $(\text {CDCl}_{3}$, 75 MHz): $\delta$ 14.3, 20.6, 25.1, 25.6, 25.8, 32.2, 32.2, 32.4, 35.9, 39.4, 49.6 (C-5), 52.5 $(\text {O}C\text {H}_{3})$, 60.5, (C-2), 73.5 (C-1), 177.2 $(C\text {ONH})$, 177.9 $(C\text {OOCH}_{3})$; HRMS (ESI+) calcd for $\text {C}_{16}\text {H}_{28}\text {N}_{2}\text {O}_{3}\text {Na}$: 319.1998 (M+Na)$^{+}$ found 319.2012.

#### Methyl $(2S)$-1-(1-($n$-butylcarbamoyl)-1-benzyl-3-phenyl-1-ethyl)-pyrrolidine-2-carboxylate **1k**

From L-proline (0.58 g, 5.04 mmol), diphenyl-2-propanone (0.88 g, 4.20 mmol) and $n$-butyl isocyanide (0.44 mL, 4.20 mmol); FC (gradient: PE/AcOEt 10:1 to 4:1): yield 0.69 g (39 %). Pale-yellow oil; TLC (PE/AcOEt 5:1): $R_\mathrm{f}=0.27$; $[\alpha ]_\mathrm{D}=-50.5$ ($c$ 1.340, $\text {CHCl}_{3})$; IR (KBr): 669, 700, 750, 1169, 1210, 1454, 1522, 1672, 1730, 2870, 2954, 3325; $^{1}\text {H}$ NMR $(\text {CDCl}_{3}$, 500 MHz): $\delta$ 0.93 (t, $^{3}J=7.5$, 3H, $\text {CH}_{2}\text {CH}_{2}\text {CH}_{2}\text {C}H_{3})$, 1.35 (m, 2H, $\text {CH}_{2}\text {CH}_{2}\text {CH}_{2}\text {CH}_{3})$, 1.46 (m 2H, $\text {CH}_{2}\text {C}H_{2}\text {CH}_{2}\text {CH}_{3})$, 1.72–1.87 (m, 4H, H-3, H$'$-3, H-4, H$'$-4), 2.86 (d, $^{2}J=13.5$, 1H, $\text {CH}_{2})$, 3.07–3.21 (m, 6H, H-5, H$'$-5, $\text {CH}_{2}$, 2x$\text {C}H_{2}'$,$\text {C}H_{2}\text {CH}_{2}\text {CH}_{2}\text {CH}_{3})$, 3.27 (m, 1H, $\text {C}H_{2}\text {CH}_{2}\text {CH}_{2}\text {CH}_{3})$, 3.61 (s, 3H, $\text {OC}H_{3})$, 3.71 (m, 1H, H-2), 7.18–7.37 (m, 10H, H-Ar), 7.86 (bpt, $^{3}J=5.5$, 1H, $\text {CON}H)$; $^{13}\text {C}$ NMR $(\text {CDCl}_{3}$, 125 MHz): $\delta$ 14.1 $(\text {CH}_{2}\text {CH}_{2}\text {CH}_{2}C\text {H}_{3})$, 20.6 $(\text {CH}_{2}\text {CH}_{2}C\text {H}_{2}\text {CH}_{3})$, 24.1 (C-4), 31.7 (C-3), 31.9 $(\text {CH}_{2}C\text {H}_{2}\text {CH}_{2}\text {CH}_{3})$, 37.8 $(C\text {H}_{2}\text {CH}_{2}\text {CH}_{2}\text {CH}_{3})$, 39.6 $(C\text {H}_{2})$, 40.9 $(C\text {H}_{2}^{{\prime }})$, 48.1 (C-5), 52.3 $(\text {O}C\text {H}_{3})$, 60.2 (C-2), 69.3 (C-1), 126.8, 127.0, 128.28, 128.32, 130.95, 130.99, 137.1, 138.2 (C-Ar), 174.6 $(C\text {ONH})$, 179.1 $(C\text {OOCH}_{3})$; HRMS (ESI+) calcd for $\text {C}_{26}\text {H}_{34}\text {N}_{2}\text {O}_{3}\text {Na}$: 445.2467 (M+Na)$^{+}$ found 445.2468.

#### Methyl $(2S,4R)$-1-(1-(*tert*-butylcarbamoyl)-cyclopentyl)-4-hydroxypyrrolidine-2-carboxylate **1m**

From *trans*-4-hydroxy-L-proline (1.32 g, 10.08 mmol), cyclopentanone (0.74 mL, 8.40 mmol) and *tert-*butyl isocyanide (0.95 mL, 8.40 mmol); FC (gradient: PE/AcOEt 5:1 to 1:2): yield 0.18 g (7 %). Pale-yellow wax; M.p. 56–$$63\,^{\circ }\text {C}$$; TLC (PE/AcOEt 3:1): $$R_\mathrm{f}=0.09$$; $$[\alpha ]_\mathrm{D}=+0.5$$ ($$c$$ 0.840, $$\text {CHCl}_{3})$$; IR (KBr): 768, 1202, 1455, 1507, 1652, 1734, 2873, 2960, 3311; $$^{1}\text {H}$$ NMR $$(\text {CDCl}_{3}$$, 300 MHz): $$\delta $$ 1.34 (s, 9H, $$\text {C}(\text {C}H_{3})_{3})$$, 1.54–1.90 (m, 7H), 2.00 (m, 1H), 2.09–2.24 (m, 2H), 2.58 (bs, 1H O$$H)$$, 2.67 (4d, $$^{2}J=10.0$$, $$^{3}J_{1}=4.5$$, $$^{3}J_{2}=1.0$$, 1H, H-5), 3.26 (dd, $$^{2}J=^{3}J_{1}=10.0$$, $$^{3}J_{2}=5.0$$, 1H, H$$'$$-5), 3.73 (s, 3H, $$\text {OC}H_{3})$$, 3.86 (dd, $$^{3}J_{1}=8.0$$, $$^{3}J_{2}=6.5$$, 1H, H-2), 4.39 (m, 1H, H-4), 7.36 (bs, 1H, $$\text {CON}H)$$; $$^{13}\text {C}$$ NMR $$(\text {CDCl}_{3}$$, 75 MHz): $$\delta $$ 25.2, 25.5, 29.1 $$(\text {C}(C\text {H}_{3})_{3})$$, 29.4, 36.6, 40.5, 50.7, 52.6, 57.7, 59.9, 70.6, 74.1, 176.5 $$(C\text {ONH})$$, 177.7 $$(C\text {OOCH}_{3})$$; HRMS (ESI+) calcd for $$\text {C}_{16}\text {H}_{28}\text {N}_{2}\text {O}_{4}\text {Na}$$: 335.1947 (M+Na)$$^{+}$$ found 335.1948.

#### Methyl $(2S)$-1-(1-(*tert*-butylcarbamoyl)-cyclopentyl)-indoline-2-carboxylate **1n**

From ($$S)$$-(-)-indoline-2-carboxylic acid (1.64 g, 10.08 mmol), cyclopentanone (0.74 mL, 8.40 mmol) and *tert-*butyl isocyanide (0.95 mL, 8.40 mmol); FC (gradient: PE/AcOEt 10:1 to 7:1): yield 1.47 g (51 %). Pale-yellow wax; M.p. 93–$$95\,^{\circ }\text {C}$$; TLC (PE/AcOEt 5:1): $$R_\mathrm{f}=0.41$$; $$[\alpha ]_\mathrm{D}=+13.7$$ ($$c$$ 0.913, $$\text {CHCl}_{3})$$; IR (KBr): 745, 1202, 1457, 1515, 1539, 1680, 1738, 2874, 2960, 3334; $$^{1}\text {H}$$ NMR $$(\text {CDCl}_{3}$$, 300 MHz): $$\delta $$ 1.34 (s, 9H, $$\text {C}(\text {C}H_{3})_{3})$$, 1.50–166 (m, 3H), 1.67–1.98 (m, 4H), 2.68 (m, 1H), 3.04 (dd, $$^{2}J=16.5$$, $$^{3}J=6.0$$, 1H, H-3), 3.57 (dd, $$^{2}J=16.5$$, $$^{3}J=12.0$$, 1H, H$$'$$-3), 3.82 (s, 3H, $$\text {OC}H_{3})$$, 4.48 (dd, $$^{3}J_{1}=12.0$$, $$^{3}J_{2}=6.0$$, 1H, H-2), 6.41 (d, $$^{3}J=8.0$$, 1H, H-Ar), 6.68 (dt, $$^{3}J$$=7.5, $$^{4}J$$=0.5, 1H, H-Ar), 7.00 (m, 2H, H-Ar), 8.16 (bs, 1H, $$\text {CON}H)$$; $$^{13}\text {C}$$ NMR $$(\text {CDCl}_{3}$$, 75 MHz): $$\delta $$ 25.3, 25.5, 28.9 $$(\text {C}(C\text {H}_{3})_{3})$$, 32.1, 34.7, 38.0, 50.9, 53.1, 61.4, 72.4, 111.0, 118.9, 124.3, 127.5, 127.9, 148.8 (C-Ar), 174.9 $$(C\text {ONH})$$, 176.8 $$(C\text {OOCH}_{3})$$; HRMS (ESI+) calcd for $$\text {C}_{20}\text {H}_{28}\text {N}_{2}\text {O}_{3}\text {Na}$$: 367.1998 (M+Na)$$^{+}$$ found 367.1995.

#### Methyl ($N$-(1-(*tert*-butylcarbamoyl)-cyclopentyl)-$N$-phenyl)-aminoacetate **1o**

From $N$-phenylglycine (1.52 g, 10.08 mmol), cyclopentanone (0.74 mL, 8.40 mmol) and *tert-*butyl isocyanide (0.95 mL, 8.40 mmol); FC (gradient: PE/AcOEt 10:1 to 6:1): yield 0.92 g (33 %). White wax; M.p. 88–$90\,^{\circ }\text {C}$; TLC (PE/AcOEt 5:1): $R_\mathrm{f}=0.40$; IR (KBr): 693, 751, 1215, 1456, 1506, 1671, 1740, 2874, 2960, 3336; $^{1}\text {H}$ NMR $(\text {CDCl}_{3}$, 300 MHz): $\delta$ 1.18 (s, 9H, $\text {C}(\text {C}H_{3})_{3})$, 1.52–1.72 (m, 2H), 1.72–1.96 (m, 4H), 2.45 (m, 2H), 3.83 (s, 3H, $\text {OC}H_{3})$, 4.12 (s, 2H, $\text {C}H_{2})$, 6.63 (m, 2H, H-Ar), 6.78 (m, 1H, H-Ar), 7.18 (m, 2H, H-Ar), 7.86 (bs, 1H, $\text {CON}H)$; $^{13}\text {C}$ NMR $(\text {CDCl}_{3}$, 75 MHz): $\delta$ 25.8, 28.7 $(\text {C}(C\text {H}_{3})_{3})$, 37.1, 49.4, 50.9, 53.0, 74.5, 115.5, 119.0, 129.3, 146.7 (C-Ar), 174.7 $(C\text {ONH})$, 175.3 $(C\text {OOCH}_{3})$; HRMS (ESI+) calcd for $\text {C}_{19}\text {H}_{28}\text {N}_{2}\text {O}_{3}\text {Na}$: 355.1998 (M+Na)$^{+}$ found 355.1999.

#### Methyl ($N$-benzyl-$N$-(1-(*tert*-butylcarbamoyl)-cyclopentyl)-aminoacetate **1p**

From $N$-benzylglycine (generated from $N$-benzylglycine hydrochloride (1.69 g, 10.08 mmol) and triethylamine (1.40 mL, 10.08 mmol)), cyclopentanone (0.74 mL, 8.40 mmol) and *tert-*butyl isocyanide (0.95 mL, 8.40 mmol); FC (gradient: PE/AcOEt 10:1 to 6:1): yield 0.58 g (20 %). White wax; M.p. 36–$37\,^{\circ }\text {C}$; TLC (PE/AcOEt 5:1): $R_\mathrm{f}=0.45$; IR (KBr): 668, 745, 1201, 1455, 1510, 1677, 1744, 2872, 2959, 3347; $^{1}\text {H}$ NMR $(\text {CDCl}_{3}$, 300 MHz): $\delta$ 1.36 (s, 9H, $\text {C}(\text {C}H_{3})_{3})$, 1.57–1.82 (m, 6H), 2.06 (m, 2H), 3.20 (s, 2H), 3.50 (s, 3H, $\text {OC}H_{3})$, 3.65 (s, 2H), 7.21-7.39 (m, 5H, H-Ar), 7.83 (bs, 1H, $\text {CON}H)$; $^{13}\text {C}$ NMR $(\text {CDCl}_{3}$, 75 MHz): $\delta$ 25.9, 29.1 $(\text {C}(C\text {H}_{3})_{3})$, 33.0, 50.6, 52.1, 56.8, 76.8, 127.7, 128.8, 129.0, 139.2 (C-Ar), 173.6 $(C\text {ONH})$, 176.8 $(C\text {OOCH}_{3})$; HRMS (ESI+) calcd for $\text {C}_{20}\text {H}_{30}\text {N}_{2}\text {O}_{3}\text {Na}$: 369.2154 (M+Na)$^{+}$ found 369.2141.

#### Methyl $(2S)$-1-(1-(*tert*-butylcarbamoyl)-cyclopentyl)-piperidine-2-carboxylate **1q**

From L-pipecolic acid (1.30 g, 10.08 mmol), cyclopentanone (0.74 mL, 8.40 mmol) and *tert-*butyl isocyanide (0.95 mL, 8.40 mmol); FC (gradient: PE/AcOEt 10:1 to 7:1): yield 0.31 g (12 %). Pale-yellow oil; TLC (PE/AcOEt 5:1): $R_\mathrm{f}=0.51$; $[\alpha ]_\mathrm{D}=+10.1$ ($c$ 0.933, $\text {CHCl}_{3})$; IR (KBr): 668, 772, 1159, 1456, 1508, 1518, 1539, 1680, 1736, 2869, 2954, 3316; $^{1}\text {H}$ NMR $(\text {CDCl}_{3}$, 300 MHz): $\delta$ 1.34 (s, 9H, $\text {C}(\text {C}H_{3})_{3})$, 1.37–1.92 (m, 12H), 1.98 (m, 2H), 2.49 (dt, $^{2}J=12.5$, $^{3}J_{1}=^{3}J_{2}=$4.0, 1H, H-6), 3.14 (td, $^{2}J=12.5$, $^{3}J_{1}=^{3}J_{2}=3.5$, 1H, H$'$-6), 3.69 (s, 3H, $\text {OC}H_{3})$, 3.78 (m, 1H, H-2), 7.54 (bs, 1H, $\text {CON}H)$; $^{13}\text {C}$ NMR $(\text {CDCl}_{3}$, 75 MHz): $\delta$ 21.0, 25.1, 25.3, 26.5, 29.1 $(\text {C}(C\text {H}_{3})_{3})$, 30.0, 32.2, 34.2, 44.5, 50.4 $(C(\text {CH}_{3})_{3})$, 51.9 $(\text {O}C\text {H}_{3})$, 56.3 (C-2), 76.8 (C-1), 175.9 $(C\text {ONH})$, 177.3 $(C\text {OOCH}_{3})$; HRMS (ESI+) calcd for $\text {C}_{17}\text {H}_{30}\text {N}_{2}\text {O}_{3}\text {Na}$: 333.2154 (M+Na)$^{+}$ found 333.2165.

#### Methyl $(2S)$-1-(1-($n$-butylcarbamoyl)-cyclopentyl)-piperidine-2-carboxylate **1r**

From L-pipecolic acid (0.65 g, 5.04 mmol), cyclopentanone (0.37 mL, 4.20 mmol) and $n$-butyl isocyanide (0.44 mL, 4.20 mmol); FC (gradient: PE/AcOEt 10:1 to 3:1): yield 0.33 g (25 %). Pale-yellow oil; TLC (PE/AcOEt 5:1): $R_\mathrm{f}=0.21$; $[\alpha ]_\mathrm{D}=-2.0$ ($c$ 0.700, $\text {CHCl}_{3})$; IR (KBr): 758, 1157, 1447, 1506, 1655, 1736, 2866, 2934, 3368; $^{1}\text {H}$ NMR $(\text {CDCl}_{3}$, 500 MHz): $\delta$ 0.93 (t, 3H, $^{3}J=7.5$, $\text {CH}_{2}\text {CH}_{2}\text {CH}_{2}\text {C}H_{3})$, 1.31–1.53 (m, 6H), 1.54–1.66 (m, 3H), 1.67–1.88 (m, 6H), 1.90–2.02 (m, 3H), 2.51 (dt, $^{2}J=12.5$, $^{3}J_{1}=^{3}J_{2}=4.0$, 1H, H-6), 3.12 (td, $^{2}J=12.5$, $^{3}J_{1}=^{3}J_{2}=2.5$, 1H, H$'$-6), 3.23 (m, 2H, $\text {C}H_{2}\text {CH}_{2}\text {CH}_{2}\text {CH}_{3})$, 3.70 (s, 3H, $\text {OC}H_{3})$, 3.79 (m, 1H, H-2), 7.69 (bpt,$^{3}J=$5.5, 1H, $\text {CON}H)$; $^{13}\text {C}$ NMR $(\text {CDCl}_{3}$, 125 MHz): $\delta$ 14.1 $(\text {CH}_{2}\text {CH}_{2}\text {CH}_{2}C\text {H}_{3})$, 20.5 $(\text {CH}_{2}\text {CH}_{2}C\text {H}_{2}\text {CH}_{3})$, 21.0, 24.7, 24.9, 26.3, 29.7, 32.2, 32.7 $(\text {CH}_{2}C\text {H}_{2}\text {CH}_{2}\text {CH}_{3})$, 34.2, 39.3 $(C\text {H}_{2}\text {CH}_{2}\text {CH}_{2}\text {CH}_{3})$, 44.5, 51.8 $(\text {O}C\text {H}_{3})$, 56.3 (C-2), 76.3 (C-1), 176.0 $(C\text {ONH})$, 177.6 $(C\text {OOCH}_{3})$; HRMS (ESI+) calcd for $\text {C}_{17}\text {H}_{30}\text {N}_{2}\text {O}_{3}\text {Na}$: 333.2154 (M+Na)$^{+}$ found 333.2167.

#### Methyl $\varvec{(2S,1R)}$- and $\varvec{(2S,1S)}$-1-(1-(*tert*-butylcarbamoyl)-1-methyl-1-propyl)-pyrrolidine-2-carboxylate $\varvec{(2S,1R)}$-**1u** and $\varvec{(2S,1S)}$-**1u**

From L-proline (1.16 g, 10.08 mmol), 2-butanone (0.75 mL, 8.40 mmol) and *tert-*butyl isocyanide (0.95 mL, 8.40 mmol); FC (gradient: PE/AcOEt 9:1 to 3:1): yield 0.98 g (41 %) of chromatographically inseparable diastereomeric mixture (dr = 86:14, LC/MS). A sample of diastereomerically pure $\varvec{(2S,1R)}$-**1u** (major isomer) was obtained by recrystallization from PE. $\varvec{(2S,1R)}$-**1u**: White wax, M.p. 53–$54\,^{\circ }\text {C}$; TLC (PE/AcOEt 5:1): $R_\mathrm{f}=0.35$; [$\alpha ]_\mathrm{D}=-19.0$ ($c$ 1, $\text {CHCl}_{3})$; IR (KBr): 1200, 1454, 1508, 1678, 1744, 2962, 3360; $^{1}\text {H}$ NMR $(\text {CDCl}_{3}$, 500 MHz): $\delta$ 0.85 (t, $^{3}J=7.5$, 3H, $\text {CH}_{2}\text {C}H_{3})$, 1.12 (s, 3H, $\text {C}H_{3})$, 1.30 (s, 9H, $\text {C}(\text {C}H_{3})_{3})$, 1.66–2.02 (m, 6H, H-3, H$'$-3, H-4, H$'$-4, $\text {C}H_{2})$, 2.70 (m, 1H, H-5), 3.00 (m, 1H, H$'$-5), 3.72 (m, 4H, H-2, $\text {OC}H_{3})$, 7.27 (bs, 1H, $\text {CON}H)$; $^{13}\text {C}$ NMR (from diastereomeric mixture, $\text {CDCl}_{3}$, 125 MHz): $\delta$ 8.5 $(C\text {H}_{3})$, 15.8 $(C\text {H}_{3}'$), 24.4 (C-4), 28.5 $(\text {C}(C\text {H}_{3})_{3})$, 30.6 $(C\text {H}_{2})$, 31.5 (C-3), 47.2 (C-5), 50.2 $(C(\text {CH}_{3})_{3})$, 51.9 $(\text {O}C\text {H}_{3})$, 60.7 (C-2), 65.7 (C-1), 174.9 $(C\text {ONH})$, 177.4 $(C\text {OOCH}_{3})$; LC/MS: 285 [M+H]$^{+}$, retention time: 14.4 min; HRMS (ESI+) calcd for $\text {C}_{15}\text {H}_{18}\text {N}_{2}\text {O}_{3}\text {Na}$: 307.1998 (M+Na)$^{+}$ for 307.1969. $\varvec{(2S,1S)}$-**1u**: $^{1}\text {H}$ NMR (from diastereomeric mixture, $\text {CDCl}_{3}$, 500 MHz): $\delta$ 0.82 (t, $^{3}J=7.5$, 3H, $\text {CH}_{2}\text {C}H_{3})$, 1.08 (s, 3H, $\text {C}H_{3})$, 1.35 (s, 9H, $\text {C}(\text {C}H_{3})_{3})$, 1.66–1.90 (m, 5H, H-3, H-4, H$'$-4, $\text {C}H_{2})$, 2.07 (m, 1H, H$'$-3), 2.70 (m, 1H, H-5), 2.89 (m, 1H, H$'$-5), 3.66 (dd, $^{3}J_{1}=10.0$,$^{3}J_{2}=4.0$, 1H, H-2), 3.72 (s, 3H, $\text {OC}H_{3})$, 7.36 (bs, 1H, $\text {CON}H)$; $^{13}\text {C}$ NMR (from diastereomeric mixture, $\text {CDCl}_{3}$, 125 MHz): $\delta$ 9.2 $(C\text {H}_{3})$, 13.8 $(C\text {H}_{3}'$), 24.8 (C-4), 28.7 $(\text {C}(C\text {H}_{3})_{3})$, 32.1 (C-3), 31.5 $(C\text {H}_{2})$, 48.7 (C-5), 50.1 $(C(\text {CH}_{3})_{3})$, 51.9 $(\text {O}C\text {H}_{3})$, 58.5 (C-2), 65.9 (C-1), 174.3 $(C\text {ONH})$, 177.6 $(C\text {OOCH}_{3})$; LC/MS: 285 [M+H]$^{+}$, retention time: 13.6 min.

#### Methyl $\varvec{(2S,1R)}$- and $\varvec{(2S,1S)}$-1-(1-(*tert*-butylcarbamoyl)-1,2-dimethyl-1-propyl)-pyrrolidine-2-carboxylate $\varvec{(2S,1R)}$-**1v** and $\varvec{(2S,1S)}$-**1v**

From L-proline (1.16 g, 10.08 mmol), 3-methyl-2-butanone (0.90 mL, 8.40 mmol) and *tert-*butyl isocyanide (0.95 mL, 8.40 mmol); FC (gradient: PE/AcOEt 10:1 to 5:1): yield 0.20 g (8 %): 0.03 g (1 %) of $\varvec{(2S,1R)}$-**1v**, 0.10 g (4 %) of $\varvec{(2S,1S)}$-**1v**, and 0.07 g (3 %) of diastereomeric mixture. $\varvec{(2S,1R)}$-**1v**: White wax; 49–$57\,^{\circ }\text {C}$; TLC (PE/AcOEt 5:1): $R_\mathrm{f}=0.41$; $[\alpha ]_\mathrm{D}=-21.3$ ($c$ 0.800, $\text {CHCl}_{3})$; IR (KBr): 766, 1161, 1202, 1456, 1509, 1668, 1742, 2844, 2962, 3354; $^{1}\text {H}$ NMR $(\text {CDCl}_{3}$, 300 MHz): $\delta$ 0.88 (d, $^{3}J=6.5$, 3H, $(\text {CH}(\text {C}H_{3})_{2})$, 1.10 (d, $^{3}J=6.5$, 3H, $(C(\text {C}H_{3})_{2}^{{\prime }})$, 1.14 (s, 3H, $\text {C}H_{3})$, 1.28 (s, 9H, $\text {C}(\text {C}H_{3})_{3})$, 1.69–1.96 (m, 5H, H-3, H$'$-3, H-4, H$'$-4, $\text {C}H(\text {CH}_{3})_{2})$, 2.73 (m, $^{2}J=^{3}J_{1}=9.0$, $^{3}J=6.5$, 1H, H-5), 3.03 (m, $^{2}J=^{3}J_{1}=9.0$, $^{3}J=1.5$, 1H, H$'$-5), 3.62 (dd,$^{3}J_{1}=7.0$, $^{3}J=2.5$, 1H, H-2), 3.70 (s, 3H, $\text {OC}H_{3})$, 6.92 (bs, 1H, $\text {CON}H)$; $^{13}\text {C}$ NMR $(\text {CDCl}_{3}$, 75 MHz): $\delta$ 14.0 $(C\text {H}_{3})$, 18.7, 18.8 (2xCH$(C\text {H}_{3})_{2})$, 24.9 (C-4), 29.0 $(\text {C}(C\text {H}_{3})_{3})$, 32.2 (C-3), 35.8 $(C\text {H}(\text {CH}_{3})_{2})$, 47.7 (C-5), 51.0 $(C(\text {CH}_{3})_{3})$, 52.2 $(\text {O}C\text {H}_{3})$, 62.5 (C-2), 70.0 (C-1), 172.5 $(C\text {ONH})$, 177.7 $(C\text {OOCH}_{3})$; LC/MS: 299 [M+H]$^{+}$, retention time: 17.9 min; HRMS (ESI+) calcd for $\text {C}_{16}\text {H}_{30}\text {N}_{2}\text {O}_{3}\text {Na}$: 321.2154 (M+Na)$^{+}$ found 321.2157.$\varvec{(2S,1S)}$-**1v**: Colorless oil; TLC (PE/AcOEt 5:1): $R_\mathrm{f}=0.37$; $[\upalpha ]_\mathrm{D}=-39.8$($c$ 0.733, $\text {CHCl}_{3})$; IR (KBr): 758, 1170, 1199, 1453, 1507, 1670, 1734, 2876, 2965, 3365; $^{1}\text {H}$ NMR $(\text {CDCl}_{3}$, 300 MHz): $\delta$ 0.86 (d, $^{3}J=6.5$, 3H, $(\text {CH}(\text {C}H_{3})_{2})$, 0.93 (d, $^{3}J=6.5$, 3H, (C$(\text {C}H_{3})_{2}^{{\prime }})$, 1.10 (s, 3H, $\text {C}H_{3})$, 1.36 (s, 9H, $\text {C}(\text {C}H_{3})_{3})$, 1.72 (m, 1H, H-4), 1.86 (m, 3H, H-3, H$'$-4, $\text {C}H(\text {CH}_{3})_{2})$, 2.02 (m, 1H, H$'$-3) 2.87 (m, 1H, H-5), 3.05 (m, 1H, H$'$-5), 3.70 (m, 4H, H-2, $\text {OC}H_{3})$, 6.63 (bs, 1H, $\text {CON}H)$; $^{13}\text {C}$ NMR $(\text {CDCl}_{3}$, 75 MHz): $\delta$ 14.3 $(C\text {H}_{3})$, 18.91, 18.93 (2xCH$(C\text {H}_{3})_{2})$, 25.3 (C-4), 29.3 $(\text {C}(C\text {H}_{3})_{3})$, 32.8 (C-3), 35.4 $(C\text {H}(\text {CH}_{3})_{2})$, 50.6 (C-5), 51.1 $(C(\text {CH}_{3})_{3})$, 52.3 $(\text {O}C\text {H}_{3})$, 60.2 (C-2), 69.9 (C-1), 172.5 $(C\text {ONH})$, 177.7 $(C\text {OOCH}_{3})$; LC/MS: 299 [M+H]$^{+}$, retention time: 15.2 min; HRMS (ESI+) calcd for $\text {C}_{16}\text {H}_{30}\text {N}_{2}\text {O}_{3}\text {Na}$: 321.2154 (M+Na)$^{+}$ found 321.2149.

#### Methyl $\varvec{(2S,1R)}$- and $\varvec{(2S,1S)}$-1-(1-(*tert*-butylcarbamoyl)-1-methyl-3-phenyl-1-ethyl)-pyrrolidine-2-carboxylate $\varvec{(2S,1R)}$-**1w** and $\varvec{(2S,1S)}$-**1w**

From L-proline (1.16 g, 10.08 mmol), phenyl-2-propanone (1.12 mL, 8.40 mmol) and *tert-*butyl isocyanide (0.95 mL, 8.40 mmol); FC (gradient: PE/AcOEt 12:1 to 7:1): yield 1.10 g (38 %): 0.38 g (13 %) of **major-1w**, 0.39 g (13 %) of **minor-1w** and 0.33 g (12 %) of diastereomeric mixture. **Major-1w**: Pale-yellow wax; M.p. 61–$68\,^{\circ }\text {C}$; TLC (PE/AcOEt 5:1): $R_\mathrm{f}=0.39$; $[\upalpha ]_\mathrm{D}=-27.5$ ($c$ 0.613, $\text {CHCl}_{3})$; IR (KBr): 703, 764, 1205, 1454, 1509, 1676, 1734, 2874, 2967, 3314; $^{1}\text {H}$ NMR $(\text {CDCl}_{3}$, 300 MHz): $\delta$ 1.16 (s, 3H, $\text {C}H_{3})$, 1.32 (s, 9H, $\text {C}(\text {C}H_{3})_{3})$, 1.75–2.00 (m, 4H, H-3, H$'$-3, H-4, H$'$-4), 3.02–3.11 (m, 3H, H-5, 2x$\text {C}H_{2})$, 3.15 (m, 1H, H$'$-5), 3.39 (dd, $^{3}J_{1}=9.0$,$^{3}J_{2}=1.0$, 1H, H-2), 3.64 (s, 3H, $\text {OC}H_{3})$, 7.16–7.28 (m, 5H, H-Ar), 7.80 (bs, 1H, $\text {CON}H)$; $^{13}\text {C}$ NMR $(\text {CDCl}_{3}$, 75 MHz): $\delta$ 21.8 $(C\text {H}_{3})$, 24.9 (C-4), 29.0 $(\text {C}(C\text {H}_{3})_{3})$, 32.0 (C-3), 39.2 $(C\text {H}_{2})$, 48.1 (C-5), 50.7 $(C(\text {CH}_{3})_{3})$, 52.4 $(\text {O}C\text {H}_{3})$, 60.1 (C-2), 65.6 (C-1), 126.8, 128.4, 131.1, 138.6 (C-Ar), 175.7 $(C\text {ONH})$, 178.8 $(C\text {OOCH}_{3})$; LC/MS: 347 [M+H]$^{+}$, retention time: 20.3 min; HRMS (ESI+) calcd for $\text {C}_{20}\text {H}_{30}\text {N}_{2}\text {O}_{3}\text {Na}$: 369.2154 (M+Na)$^{+}$ found 369.2136. **Minor-1w**: Pale-yellow wax; M.p. 42–$49\,^{\circ }\text {C}$; TLC (PE/AcOEt 5:1): $R_\mathrm{f}=0.30$; $[\alpha ]_\mathrm{D}=-11.4$ ($c$ 0.733, $\text {CHCl}_{3})$; IR (KBr): 704, 768, 1213, 1455, 1514, 1683, 1735, 2872, 2966, 3314; $^{1}\text {H}$ NMR $(\text {CDCl}_{3}$, 300 MHz): $\delta$ 1.10 (s, 3H, $\text {C}H_{3})$, 1.37 (s, 9H, $\text {C}(\text {C}H_{3})_{3})$, 1.68–1.94 (m, 3H, H-3, H-4, H$'$-4), 2.11 (m, 1H, H$'$-3), 2.67 (d,$^{2}J=12.5$, 1H, $\text {C}H_{2})$, 2.76 (m, $^{2}J=^{3}J_{1}=8.5$,$^{3}J_{2}=6.5$, 1H, H-5), 2.99 (m, 2H, H$'$-5, $\text {C}H_{2})$, 3.72 (s, 3H, $\text {OC}H_{3})$, 3.82 (dd, $^{3}J_{1}=9.5$,$^{3}J_{2}=3.0$, 1H, H-2), 7.13–7.25 (m, 5H, H-Ar), 7.30 (bs, 1H, $\text {CON}H)$; $^{13}\text {C}$ NMR $(\text {CDCl}_{3}$, 75 MHz): $\delta$ 16.2 $(C\text {H}_{3})$, 25.2 (C-4), 29.2 $(\text {C}(C\text {H}_{3})_{3})$, 32.6 (C-3), 44.8 $(C\text {H}_{2})$, 49.3 (C-5), 50.9 $(C(\text {CH}_{3})_{3})$, 52.5 $(\text {O}C\text {H}_{3})$, 55.6 (C-2), 67.0 (C-1), 127.1, 128.3, 131.0, 137.4 (C-Ar), 174.2 $(C\text {ONH})$, 177.9 $(C\text {OOCH}_{3})$; LC/MS: 347 [M+H]$^{+}$, retention time: 17.7 min; HRMS (ESI+) calcd for $\text {C}_{20}\text {H}_{30}\text {N}_{2}\text {O}_{3}\text {Na}$: 369.2154 (M+Na)$^{+}$ found 369.2171.

#### Methyl $\varvec{(2S,1R)}$- and $\varvec{(2S,1S)}$-1-(1-(tosylmethylcarbamoyl)-1,2-dimethyl-1-propyl)-pyrrolidine-2-carboxylate $\varvec{(2S,1R)}$-**1x** and $\varvec{(2S,1S)}$-**1x**

From L-proline (1.16 g, 10.08 mmol), 3-methyl-2-butanone (0.90 mL, 8.40 mmol) and tosylmethyl isocyanide (1.64 g, 8.40 mmol); FC (gradient: PE/AcOEt 5:1 to 1:1): yield 1.38 g (40 %): 0.91 g (26 %) of $\varvec{(2S,1R)}$-**1x**, 0.12 g (4 %) of $\varvec{(2S,1S)}$-**1x** and 0.32 g (10 %) of diastereomeric mixture. $\varvec{(2S,1R)}$-**1x**: Colorless oil; TLC (PE/AcOEt 3:1): $R_\mathrm{f}=0.19$; $[\alpha ]_\mathrm{D}=-16.1$ ($c$ 1.065, $\text {CHCl}_{3})$; IR (KBr): 565, 669, 745, 1142, 1205, 1323, 1506, 1685, 1733, 2876, 2956, 3310; $^{1}\text {H}$ NMR $(\text {CDCl}_{3}$, 300 MHz): $\delta$ 0.70 (d, $^{3}J=7.0$, 3H, $(\text {CH}(\text {C}H_{3})_{2})$, 1.00 (d, $^{3}J=7.0$, 3H, $\text {CH}(\text {C}H_{3})_{2}^{{\prime }})$, 1.06 (s, 3H, $\text {C}H_{3})$, 1.66–1.89 (m, 2H, H-4, H$'$-4), 1.90–2.06 (m, 3H, $\text {C}H(\text {CH}_{3})_{2}$, H-3, H$'$-3), 2.42 (s, 3H, Ar-$\text {C}H_{3})$, 2.72 (m, 1H, H-5), 2.91 (m, 1H, H$'$-5), 3.75 (m, 4H, H-2, $\text {OC}H_{3})$, 4.51 (dd, $^{2}J=14.0$, $^{3}J=6.5$, 1H, $\text {C}H_{2})$, 4.80 (dd, $^{2}J=14.0$, $^{3}J=7.5$, 1H, $\text {C}H_{2}'$), 7.33 (d, $^{3}J=8.0$, 2H, H-Ar), 7.81 (d, $^{3}J=8.0$, 2H, H-Ar), 8.46 (pt, $^{3}J=7.0$, 1H, $\text {CON}H)$; $^{13}\text {C}$ NMR $(\text {CDCl}_{3}$, 75 MHz): $\delta$ 15.9, 17.8, 18.4, 21.8, 24.5, 31.6, 33.9, 47.2 (C-5), 52.6 $(\text {O}C\text {H}_{3})$, 61.0, 61.3, 67.9 (C-1), 129.0, 129.9, 135.2, 145.1 (C-Ar), 175.1 $(C\text {ONH})$, 178.0 $(C\text {OOCH}_{3})$; LC/MS: 411 [M+H]$^{+}$, retention time: 13.9 min; HRMS (ESI+) calcd for $\text {C}_{20}\text {H}_{30}\text {N}_{2}\text {O}_{5}\text {SNa}$: 433.1773 (M+Na)$^{+}$ found 433.1791.$\varvec{(2S,1S)}$-**1x**: Colorless oil; TLC (PE/AcOEt 3:1): $R_\mathrm{f}=0.13$; $[\alpha ]_\mathrm{D}=-17.5$ ($c$ 0.558, $\text {CHCl}_{3})$; IR (KBr): 570, 668, 761, 1142, 1320, 1506, 1699, 1733, 2876, 2965, 3418; $^{1}\text {H}$ NMR $(\text {CDCl}_{3}$, 300 MHz): $\delta$ 0.73 (d, $^{3}J=6.5$, 3H, $\text {C}H(\text {C}H_{3})_{2})$, 0.80 (d, $^{3}J=6.5$, 3H, $(\text {C}(\text {C}H_{3})_{2}^{{\prime }})$, 1.06 (s, 3H, $\text {C}H_{3})$, 1.66–2.03 (m, 5H, $\text {C}H(\text {CH}_{3})_{2}$, H-3, H$'$-3, H-4, H$'$-4), 2.43 (s, 3H, Ar-$\text {C}H_{3})$, 2.75 (m, 1H, H-5), 3.05 (m, 1H, H$'$-5), 3.66 (dd, $^{3}J_{1}=8.5$,$^{3}J_{2}=3.5$, 1H, H-2), 3.71 (s, 3H, $\text {OC}H_{3})$, 4.67 (dd, $^{2}J=14.0$, $^{3}J=6.5$, 1H, $\text {C}H_{2})$, 4.82 (dd, $^{2}J=14.0$, $^{3}J=7.5$, 1H, $\text {C}H_{2}'$), 7.35 (d, $^{3}J=8.5$, 2H, H-Ar), 7.62 (pt, $^{3}J=6.5$, 1H, $\text {CON}H)$, 7.81 (d, $^{3}J=8.5$, 2H, H-Ar); $^{13}\text {C}$ NMR $(\text {CDCl}_{3}$, 75 MHz): $\delta$ 14.8, 18.5, 18.7, 22.2, 25.2, 32.5, 34.9, 50.2 (C-5), 52.5 $(\text {O}C\text {H}_{3})$, 60.2, 60.9, 69.7 (C-1), 129.2, 130.3, 135.1, 145.7 (C-Ar), 174.0 $(C\text {ONH})$, 177.5 $(C\text {OOCH}_{3})$; LC/MS: 411 [M+H]$^{+}$, retention time: 11.9 min; HRMS (ESI+) calcd for $\text {C}_{20}\text {H}_{30}\text {N}_{2}\text {O}_{5}\text {SNa}$: 433.1773 (M+Na)$^{+}$ found 433.1775.

#### Methyl $\varvec{(2S,1R)}$- and $\varvec{(2S,1S)}$-1-(1-($n$-butylcarbamoyl)-1,2-dimethyl-1-propyl)-pyrrolidine-2-carboxylate $\varvec{(2S,1R)}$-**1y** and $\varvec{(2S,1S)}$-**1y**

From L-proline (0.58 g, 5.04 mmol), 3-methyl-2-butanone (0.45 mL, 4.20 mmol) and $n$-butyl isocyanide (0.44 mL, 4.20 mmol); FC (gradient: PE/AcOEt 7:1 to 3:1): yield 0.52 g (42 %): 0.14 g (11 %) of **major-1y**, 0.21 g (17 %) of **minor-1y** and 0.18 g (14 %) of diastereomeric mixture. **Major-1y**: Colorless oil; TLC (PE/AcOEt 5:1): $R_\mathrm{f}=0.26$; $[\alpha ]_\mathrm{D}=-38.3$ ($c$ 0.853, $\text {CHCl}_{3})$; IR (KBr): 668, 1162, 1205, 1457, 1520, 1671, 1736, 2872, 2958, 3380; $^{1}\text {H}$ NMR $(\text {CDCl}_{3}$, 300 MHz): $\delta$ 0.90 (m, 6H), 1.07 (d, $^{3}J=6.5$, 3H, $(\text {C}(\text {C}H_{3})_{2})$, 1.18 (s, 3H, $\text {C}H_{3})$, 1.23–1.50 (m, 4H), 1.68-2.00 (m, 5H), 2.74 (m, $^{2}J=^{3}J_{1}=10.0$,$^{3}J_{2}=6.5$, 1H, H-5), 2.92–3.07 (m, 2H), 3.29 (m, 1H), 3.63 (dd, $^{3}J_{1}=6.5$,$^{3}J_{2}=3.5$, 1H, H-2), 3.67 (s, 3H, $\text {OC}H_{3})$, 7.17 (pt, $^{3}J=5.5$, 1H, $\text {CON}H)$; $^{13}\text {C}$ NMR $(\text {CDCl}_{3}$, 75 MHz): $\delta$ 14.2, 14.5, 18.7, 18.8, 20.8, 24.9, 32.1, 32.2, 35.5, 39.3, 47.7 (C-5), 52.3 $(\text {O}C\text {H}_{3})$, 62.2 (C-2), 69.4 (C-1), 173.7 $(C\text {ONH})$, 178.0 $(C\text {OOCH}_{3})$; LC/MS: 299 [M+H]$^{+}$, retention time: 16.4 min; HRMS (ESI+) calcd for $\text {C}_{16}\text {H}_{30}\text {N}_{2}\text {O}_{3}\text {Na}$: 321.2154 (M+Na)$^{+}$ found 321.2155. **Minor-1y**: Pale-yellow oil; TLC (PE/AcOEt 5:1): $R_\mathrm{f}$=0.21; $[\alpha ]_\mathrm{D}=-30.9$ ($c$ 0.840, $\text {CHCl}_{3})$; IR (KBr): 758, 1157, 1202, 1457, 1520, 1652, 1734, 2873, 2960, 3366; $^{1}\text {H}$ NMR $(\text {CDCl}_{3}$, 300 MHz): $\delta$ 0.83 (d, $^{3}J=6.5$, 3H, $(\text {C}(\text {C}H_{3})_{2})$, 0.94 (m, 6H), 1.14 (s, 3H, $\text {C}H_{3})$, 1.36 (m, 2H), 1.49 (m, 2H), 1.80–1.95 (m, 4H), 2.03 (m, 1H), 2.87 (m, $^{2}J=^{3}J_{1}=9.5$,$^{3}J_{2}=6.5$, 1H, H-5), 3.04 (m, 1H), 3.25 (m, 2H), 3.70 (m, 4H, H-2, $\text {OC}H_{3})$, 6.74 (pt, $^{3}J=5.5$, 1H, $\text {CON}H)$; $^{13}\text {C}$ NMR $(\text {CDCl}_{3}$, 75 MHz): $\delta$ 14.2, 14.5, 18.8, 18.9, 20.8, 25.3, 32.4, 32.7, 35.3, 39.4, 50.5 (C-5), 52.3 $(\text {O}C\text {H}_{3})$, 60.2 (C-2), 69.8 (C-1), 173.4 $(C\text {ONH})$, 177.8 $(C\text {OOCH}_{3})$; LC/MS: 299 [M+H]$^{+}$, retention time: 13.2 min; HRMS (ESI+) calcd for $\text {C}_{16}\text {H}_{30}\text {N}_{2}\text {O}_{3}\text {Na}$: 321.2154 (M+Na)$^{+}$ found 321.2163.

#### Methyl $\varvec{(2S,1R)}$- and $\varvec{(2S,1S)}$-1-(1-(tosylmethylcarbamoyl)-1-methyl-1-propyl)-pyrrolidine-2-carboxylate $\varvec{(2S,1R)}$-**1z** and $\varvec{(2S,1S)}$-**1z**

From L-proline (1.16 g, 10.08 mmol), 2-butanone (0.75 mL, 8.40 mmol) and tosylmethyl isocyanide (1.64 g, 8.40 mmol); FC (gradient: PE/AcOEt 4:1 to 1:1): yield 1.06 g (32 %): 0.32 g (10 %) of **major-1z**, 0.29 g (9 %) of **minor-1z** and 0.45 g (13 %) of diastereomeric mixture. **Major-1z**: Colorless oil; TLC (PE/AcOEt 3:1): $R_\mathrm{f}=0.08$; $[\alpha ]_\mathrm{D}=-6.7$ ($c$ 0.915, $\text {CHCl}_{3})$; IR (KBr): 557, 671, 766, 1142, 1211, 1321, 1506, 1686, 1734, 2851, 2880, 2928, 3290; $^{1}\text {H}$ NMR $(\text {CDCl}_{3}$, 500 MHz): $\delta$ 0.72 (t, $^{3}J=7.5$, 3H, $\text {CH}_{2}\text {C}H_{3})$, 1.07 (s, 3H, $\text {C}H_{3})$, 1.67 (q,$^{3}J=7.5$, 2H, $\text {C}H_{2}\text {CH}_{3})$, 1.76–1.87 (m, 2H, H-4, H$'$-4), 1.93–2.09 (m, 2H, H-3, H$'$-3), 2.43 (s, 3H, Ar-$\text {C}H_{3})$, 2.67 (m, 1H, H-5), 2.87 (m, 1H, H$'$-5), 3.76 (s, 3H, $\text {OC}H_{3})$, 3.85 (dd, $^{3}J_{1}=9.5$, $^{3}J_{2}=1.5$, 1H, H-2), 4.59 (dd, $^{2}J=14.0$, $^{3}J=6.5$, 1H, $\text {C}H_{2})$, 4.77 (dd, $^{2}J=14.0$, $^{3}J=7.5$, 1H, $\text {C}H_{2}'$), 7.33 (d, $^{3}J=8.0$, 2H, H-Ar), 7.79 (d, $^{3}J=8.0$, 2H, H-Ar), 8.85 (pt, $^{3}J=6.5$, 1H, $\text {CON}H)$; $^{13}\text {C}$ NMR $(\text {CDCl}_{3}$, 125 MHz): $\delta$ 7.7, 19.0, 21.8, 24.4, 27.8, 31.3, 47.2 (C-5), 52.4 $(\text {O}C\text {H}_{3})$, 60.1, 60.8, 64.2 (C-1), 128.8, 129.7, 134.8, 145.0 (C-Ar), 177.0 $(C\text {ONH})$, 177.9 $(C\text {OOCH}_{3})$; LC/MS: 411 [M+H]$^{+}$, retention time: 13.9 min; HRMS (ESI+) calcd for $\text {C}_{19}\text {H}_{28}\text {N}_{2}\text {O}_{5}\text {SNa}$: 419.1617 (M+Na)$^{+}$ found 419.1605. **Minor-1z**: Colorless oil; TLC (PE/AcOEt 3:1): $R_\mathrm{f}=0.05$; $[\alpha ]_\mathrm{D}=-4.6$ ($c$ 1.150, $\text {CHCl}_{3})$; IR (KBr): 760, 1140, 1215, 1321, 1501, 1693, 1732, 2876, 2937, 3337; $^{1}\text {H}$ NMR $(\text {CDCl}_{3}$, 500 MHz): $\delta$ 0.58 (t, $^{3}J=7.5$, 3H, $\text {CH}_{2}\text {C}H_{3})$, 1.05 (s, 3H, $\text {C}H_{3})$, 1.42 (m,$^{3}J=7.5$, 2H, $\text {C}H_{2}\text {CH}_{3})$, 1.72 (m, 1H, H-4), 1.78–1.92 (m, 2H, H-3, H$'$-4), 2.06 (m, 1H, H$'$-3), 2.43 (s, 3H, Ar-$\text {C}H_{3})$, 2.66 (m, 1H, H-5), 2.87 (m, 1H, H$'$-5), 3.68 (dd, $^{3}J_{1}=9.5$,$^{3}J_{2}=3.5$, 1H, H-2), 3.75 (s, 3H, $\text {OC}H_{3})$, 4.70 (dd, $^{2}J=14.0$, $^{3}J=6.5$, 1H, $\text {C}H_{2})$, 4.75 (dd, $^{2}J=14.0$, $^{3}J=6.5$, 1H, $\text {C}H_{2}'$), 7.34 (d, $^{3}J=8.0$, 2H, H-Ar), 7.80 (d, $^{3}J=8.0$, 2H, H-Ar), 8.35 (pt, $^{3}J=6.5$, 1H, $\text {CON}H)$; $^{13}\text {C}$ NMR $(\text {CDCl}_{3}$, 125 MHz): $\delta$ 9.1, 13.6, 21.7, 24.8, 31.9, 32.0, 48.7 (C-5), 52.3 $(\text {O}C\text {H}_{3})$, 58.5, 60.6, 65.7 (C-1), 128.7, 129.9, 134.7, 145.1 (C-Ar), 175.3 $(C\text {ONH})$, 177.4 $(C\text {OOCH}_{3})$; LC/MS: 411 [M+H]$^{+}$, retention time: 11.9 min; HRMS (ESI+) calcd for $\text {C}_{19}\text {H}_{28}\text {N}_{2}\text {O}_{5}\text {SNa}$: 419.1617 (M+Na)$^{+}$ found 419.1597

#### Methyl $\varvec{(2S,1R)}$- and $\varvec{(2S,1S)}$-1-(1-(*tert*-butylcarbamoyl)-1-methyl-1-propylamino)-3-phenylpropionate $\varvec{(2S,1R)}$-**4a** and $\varvec{(2S,1S)}$-**4a**

From L-phenylalanine (1.67 g, 10.08 mmol), 2-butanone (0.75 mL, 8.40 mmol) and *tert-*butyl isocyanide (0.95 mL, 8.40 mmol); FC (gradient: PE/AcOEt 6:1 to 4:1): yield 1.21 g (43 %) of chromatographically inseparable diastereomeric mixture (dr = 50:50, LC/MS). Pale-yellow oil; TLC (PE/AcOEt 3:1): $R_\mathrm{f}=0.38$; IR (KBr): 700, 760, 1196, 1454, 1508, 1674, 1740, 2968, 3358; $^{1}\text {H}$ NMR (diastereomeric mixture, $\text {CDCl}_{3}$, 500 MHz): $\delta$ 0.58 (t, $^{3}J=7.5$, 3H, $\text {C}H_\mathrm{3I})$, 0.85 (t, $^{3}J=7.5$, 3H, $\text {C}H_\mathrm{3II})$, 1.10 (s, 6H, $\text {C}H_\mathrm{3I },\text {C}H_\mathrm{3II})$, 1.19, 1.29 (2xs, 18H, C$(\text {C}H_{3})_\mathrm{3I}$, C$(\text {C}H_{3})_\mathrm{3II})$, 1.37–1.55 (m, 2H, $\text {C}H_{2}\text {CH}_\mathrm{3II})$, 1.64 (m, 2H, $\text {C}H_{2}\text {CH}_\mathrm{3I)})$, 1.68 (bs, 1H, N$H_\mathrm{I})$, 1.94 (bs, 1H, N$H_\mathrm{II})$, 2.78-2.79 (m,3H, $\text {C}H_\mathrm{2II}$, $\text {C}H_{2}$’$_\mathrm{II}$, $\text {C}H_\mathrm{2I})$, 2.97 (dd,$^{2}J=13.5$,$^{3}J$=6.0, 1H, $\text {C}H_{2}$’$_\mathrm{I})$, 3.42 (pt, $^{3}J=7.0$, 1H, H-2$_\mathrm{II})$, 3.55 (dd, $^{3}J_{1}=8.0$,$^{3}J_{2}=6.0$, 1H, H-2$_\mathrm{I})$, 3.62 (s, 3H, O$\text {C}H_\mathrm{3I})$, 3.67 (s, 3H, O$\text {C}H_\mathrm{3II})$, 6.95 (bs, 1H, CON$H_\mathrm{II})$, 7.16 (m, 4H, H-Ar), 7.20 (bs, 1H, CON$H_\mathrm{I})$, 7.25 (m, 2H, H-Ar), 7.30 (m, 4H, H-Ar); $^{13}\text {C}$ NMR (diastereomeric mixture, $\text {CDCl}_{3})$, 125 MHz): $\delta$ 7.9 $(\text {CH}_{2}C\text {H}_\mathrm{3I})$, 8.3 $(\text {CH}_{2}C\text {H}_\mathrm{3II})$, 20.7 $(C\text {H}_\mathrm{3II})$, 22.4 $(C\text {H}_\mathrm{3I})$, 28.59, 28.63 $(\text {C}(C\text {H}_{3})_\mathrm{3II}$, $\text {C}(C\text {H}_{3})_\mathrm{3I})$, 31.2 $(C\text {H}_{2}\text {CH}_\mathrm{3I})$, 34.2 $(C\text {H}_{2}\text {CH}_\mathrm{3II})$, 41.2 $(C\text {H}_\mathrm{2I})$, 41.9 $(C\text {H}_\mathrm{2II})$, 50.1, 50.3 $(C(\text {CH}_{3})_\mathrm{3II}$, $C(\text {CH}_{3})_\mathrm{3I})$, 51.92, 51.93 $(\text {O}C\text {H}_\mathrm{3II}$, $\text {O}C\text {H}_\mathrm{3I})$, 57.2 (C-2$_\mathrm{I})$, 58.2 (C-2$_\mathrm{II})$, 62.4 (C-1$_\mathrm{II})$, 62.5 (C-1$_\mathrm{I})$, 126.9, 127.0, 128.55, 128.59, 129.33, 129.29, 137.0, 137.1 (C-Ar$_\mathrm{I}$, C-Ar$_\mathrm{II})$, 174.5, 174.8 $(C\text {ONH}_\mathrm{2I}$, $C\text {ONH}_\mathrm{2II})$, 176.0, 176.8 ($C\text {OOCH}_\mathrm{3I}$, $C\text {OOCH}_\mathrm{3II})$; LC/MS: 335 [M+H]$^{+}$, retention time: 30.7 and 33.1 min; HRMS (ESI+) calcd for $\text {C}_{19}\text {H}_{30}\text {N}_{2}\text {O}_{3}\text {Na}$: 357.2154 (M+Na)$^{+}$ found 357.2154.

#### General procedure for $\text {BF}{\bullet }_{3}2\text {CH}_{3}$COOH-mediated N-de*tert*-butylation/cyclocondensation of *tert*-butylamidoesters **1u** and **1v**

The diastereomeric mixture of the appropriate *tert*-butylamidoester was dissolved in $\text {BF}{\bullet }_{3}2\text {CH}_{3}\text {COOH}$ ($\sim$36 % $\text {BF}_{3}$ basis, 10 mL per 1.5 mmol of the substrate), at $50\,^{\circ }\text {C}$. The mixture was stirred for 6 h at $50\,^{\circ }\text {C}$ and subsequently at rt for 20 h. The resulting solution was slowly poured onto excess of crushed ice and 25 % aqueous solution of ammonia. The mixture was extracted with DCM (3$\times$40 mL) and DCM/MeOH 3:1 (1$\times$30 mL). The combined organic phase was washed with water (30 mL), dried over anhydrous $\text {MgSO}_{4}$, filtered and concentrated under reduced pressure. The residue was purified by FC.

From the diastereomeric mixture of $\varvec{(2S,1R)}$-**1u** and $\varvec{(2S,1S)}$-**1u** (490 mg, 1.72 mmol, dr = 86:14, LC/MS) and $\text {BF}{\bullet }_{3}2\text {CH}_{3}\text {COOH}$ (11 mL); FC (gradient: PE/AcOEt 5:1 to 0:1, then AcOEt/MeOH 9:1 to 5:1): yield 65 %: 105 mg (31 %) of $\varvec{(4R,8}\mathbf{a}\varvec{S)}$-**3u**, 24 mg (7 %) of $\varvec{(4S,8}\mathbf{a}\varvec{S)}$-**3u**, 56 mg (17 %) of diastereomeric mixture of $\varvec{(4R,8}\mathbf{a}\varvec{S)}$-**3u** and $\varvec{(4S,8}\mathbf{a}\varvec{S)}$-**3u** (dr$\approx$80/20, $^{1}\text {H}$ NMR) and 41 mg (10 %) of $\varvec{(2S,1R)}$-**2u**.^4^

#### Methyl $\varvec{(2S,1R)}$-1-(1-carbamoyl-1-methyl-1-propyl)-pyrrolidine-2-carboxylate $\varvec{(2S,1R)}$-**2u**

White wax; M.p. 69–$$72\,^{\circ }\text {C}$$; TLC (AcOEt): $$R_\mathrm{f}=0.48$$; $$[\alpha ]_\mathrm{D}=-9.4$$ ($$c $$0.583, $$\text {CHCl}_{3})$$; IR (KBr): 759, 802, 1201, 1456, 1578, 1680, 1731, 2852, 2880, 2936, 2969, 3212, 3340, 3436; $$^{1}\text {H}$$ NMR $$(\text {CDCl}_{3}$$, 500 MHz): $$\delta $$ 0.87 (t, 3H, $$\text {CH}_{2}\text {C}H_{3})$$, 1.20 (s, 3H, $$\text {C}H_{3})$$, 1.73–1.86 (m, 4H, $$\text {C}H_{2}\text {CH}_{3}$$, H-4, H$$'$$-4), 1.92 (m, 1H, H-3), 2.01 (m, 1H, H$$'$$-3), 2.74 (m, $$^{2}J=^{3}J_{1}=6.5$$,$$^{3}J_{2}=2.0$$, 1H, H-5), 3.02 (m, $$^{2}J=^{3}J_{1}=6.5$$, $$^{3}J_{2}=2.0$$, 1H, H$$'$$-5), 3.70 (s, 3H, $$\text {OC}H_{3})$$, 3.83 (dd, $$^{3}J_{1}=8.0$$, $$^{3}J_{2}=1.5$$, 1H, H-2), 5.32 (bs, 1H, $$\text {CON}H)$$, 7.65 (bs, 1H, CON$$H'$$);$$^{13}\text {C}$$ NMR $$(\text {CDCl}_{3}$$, 125 MHz): $$\delta $$ 8.3 $$(\text {CH}_{2}C\text {H}_{3})$$, 17.6 $$(C\text {H}_{3})$$, 24.5 (C-4), 29.2 $$(C\text {H}_{2}\text {CH}_{3})$$, 31.4 (C-3), 47.3 (C-5), 52.1 $$(\text {O}C\text {H}_{3})$$, 60.4 (C-2), 64.8 (C-$$\alpha )$$, 178.0 $$(C\text {OOCH}_{3})$$, 179.4 $$(C\text {ONH})$$; HRMS (ESI+) calcd for $$\text {C}_{11}\text {H}_{20}\text {N}_{2}\text {O}_{3}\text {Na}$$: 251.1372 (M+Na)$$^{+}$$ found 251.1344.

#### $\varvec{(4R,8}\mathbf{a}\varvec{S)}$-4-Ethyl-4-methylperhydropyrrolo[1,2-$a$]pyrazine-1,3-dione $\varvec{(4R,8}\mathbf{a}\varvec{S)}$-**3u**

White powder; M.p. 71–$$74\,^{\circ }\text {C}$$; TLC (PE/AcOEt 3:1): $$R_\mathrm{f}=0.33$$; $$[\alpha ]_\mathrm{D}=+25.5$$ ($$c $$1.373, $$\text {CHCl}_{3})$$; IR (KBr): 760, 818, 1234, 1459, 1710, 2826, 2880, 2940, 2974, 3098, 3223; $$^{1}\text {H}$$ NMR (500 MHz, CDCl$$_{3}$$: $$\delta $$ 0.91 (t, $$^{3}J=7.5$$, 3H, $$\text {CH}_{2}\text {C}H_{3})$$, 1.31 (s, 3H, $$\text {C}H_{3})$$, 1.68–1.88 (m, 3H, H-7, H-7$$'$$, $$\text {C}H_{2}\text {CH}_{3})$$, 1.94 (m, $$^{3}J=7.5$$, 1H, $$\text {C}H_{2}\text {CH}_{3}^{{\prime }})$$, 2.20 (m, 2H, H-8, H$$'$$-8), 2.65 (pq, $$^{2}J=^{3}J_{1}=^{3}J_{2}=7.5$$, 1H, H-6), 2.76 (pq, $$^{2}J=^{3}J_{1}=^{3}J_{2}=7.5$$, 1H, H$$'$$-6), 3.69 (pt, $$^{3}J=7.0$$, 1H, H-8a), 7.99 (bs, 1H, $$\text {N}H$$); $$^{13}\text {C}$$ NMR $$(\text {CDCl}_{3}$$, 125 MHz): $$\delta $$ 7.4 $$(\text {CH}_{2}C\text {H}_{3})$$, 17.9 $$(C\text {H}_{3})$$, 21.8 (C-7), 26.3 (C-8), 29.1 $$(C\text {H}_{2}\text {CH}_{3})$$, 45.5 (C-6), 59.0 (C-8a), 62.3 (C-4), 173.3 (C-3), 176.7 (C-1).

#### $\varvec{(4S,8}\mathbf{a}\varvec{S)}$-4-Ethyl-4-methylperhydropyrrolo[1,2-$a$]pyrazine-1,3-dione $\varvec{(4S,8}\mathbf{a}\varvec{S)}$-**3u**

White powder; M.p. 107–$$109\,^{\circ }\text {C}$$; TLC (PE/AcOEt 3:1): $$R_\mathrm{f}=0.23$$; $$[\alpha ]_\mathrm{D}=+1.3$$ ($$c $$0.613, $$\text {CHCl}_{3})$$; IR (KBr): 758, 1251, 1463, 1693, 2814, 2880, 2943, 2943, 2974, 3094, 3219; $$^{1}\text {H}$$ NMR (500 MHz, CDCl$$_{3})$$: $$\delta $$ 0.98 (t, $$^{3}J=7.5$$, 3H, $$\text {CH}_{2}\text {C}H_{3})$$, 1.38 (s, 3H, $$\text {C}H_{3})$$, 1.67–1.91 (m, 4H, H-7, H$$'$$-7, $$\text {C}H_{2}\text {CH}_{3})$$, 2.16–2.31 (m, 2H, H-8, H$$'$$-8), 2.46 (pq, $$^{2}J=^{3}J_{1}=^{3}J_{2}=8.5$$, 1H, H-6), 3.11 (m, $$^{2}J=^{3}J_{1}=8.5$$, $$^{3}J_{2}=3.5$$, 1H, H$$'$$-6), 3.95 (dd, $$^{3}J_{1}=8.5$$, $$^{3}J_{2}=3.0$$, 1H, H-8a), 7.89 (bs, 1H, $$\text {N}H$$); $$^{13}\text {C}$$ NMR $$(\text {CDCl}_{3}$$, 125 MHz): $$\delta $$ 7.7 $$(\text {CH}_{2}C\text {H}_{3})$$, 19.5 $$(C\text {H}_{3})$$, 22.2 (C-7), 27.6 (C-8), 30.4 $$(C\text {H}_{2}\text {CH}_{3})$$, 47.8 (C-6), 59.0 (C-8a), 62.2 (C-4), 174.4 (C-3), 176.2 (C-1); HRMS (ESI-) calcd for $$\text {C}_{10}\text {H}_{15}\text {N}_{2}\text {O}_{2}$$: 195.1134 (M-H)$$^{-}$$ found 195.1130.

From the diastereomeric mixture of $\varvec{(2S,1R)}$-**1v** and $\varvec{(2S,1S)}$-**1v** (338 mg, 1.13 mmol, dr = 21:79, LC/MS) and $\text {BF}_{3}2\text {CH}_{3}\text {COOH}$ (6 mL); FC (gradient: PE/AcOEt 6:1 to 0:1): yield 72 %: 62 mg (26 %) of inseparable diastereomeric mixture of $\varvec{(4R,8}\mathbf{a}\varvec{S)}$-**3v** and $\varvec{(4S,8}\mathbf{a}\varvec{S)}$-**3v**(dr = 30:70, $^{1}\text {H}$ NMR), 33 mg (12 %) of $\varvec{(2S,1R)}$-**2v** and 93 mg (34 %) of $\varvec{(2S,1S)}$-**2v**.

#### Methyl $\varvec{(2S,1R)}$-1-(1-carbamoyl-1,2-dimethyl-1-propyl)-pyrrolidine-2-carboxylate and $\varvec{(2S,1R)}$-**2v**

Yellow oil; TLC (AcOEt): $R_\mathrm{f}=0.52$; $[\alpha ]_\mathrm{D}=-26.0$ ($c$ 0.845, $\text {CHCl}_{3})$; IR (KBr): 760, 1167, 1211, 1369, 1458, 1509, 1655, 1736, 2852, 296, 3340, 3449; $^{1}\text {H}$ NMR $(\text {CDCl}_{3}$, 300 MHz): $\delta$ 0.90 (d, $^{3}J=6.5$, 3H, $(\text {CH}(\text {C}H_{3})_{2})$, 1.11 (d, $^{3}J=6.5$, 3H, $(\text {C}(\text {C}H_{3})_{2}^{{\prime }})$, 1.19 (s, 3H, $\text {C}H_{3})$, 1.72–2.07 (m, 5H, H-3, H$'$-3, H-4, H$'$-4, $\text {C}H(\text {CH}_{3})_{2})$, 2.77 (m, $^{2}J=^{3}J_{1}=9.5$, $^{3}J=6.5$, 1H, H-5), 3.05 (m, 1H, H$'$-5), 3.68 (s, 3H, $\text {OC}H_{3})$, 3.72 (m, 1H, H-2), 5.47 (bs, 1H, $\text {CON}H)$ 7.11 (bs, 1H, $\text {CON}H'$); $^{13}\text {C}$ NMR $(\text {CDCl}_{3}$, 75 MHz): $\delta$ 14.3 $(C\text {H}_{3})$, 17.9, 18.3 (2xCH$(C\text {H}_{3})_{2})$, 24.4 (C-4), 31.6 (C-3), 34.5 $(C\text {H}(\text {CH}_{3})_{2})$, 47.3 (C-5), 52.0 $(\text {O}C\text {H}_{3})$, 61.6 (C-2), 68.7 (C-1), 176.7 $(C\text {ONH})$, 177.7 $(C\text {OOCH}_{3})$; HRMS (ESI+) calcd for $\text {C}_{12}\text {H}_{22}\text {N}_{2}\text {O}_{3}\text {Na}$: 265.1528 (M+Na)$^{+}$ found 265.1532.

#### Methyl $\varvec{(2S,1S)}$-1-(1-carbamoyl-1,2-dimethyl-1-propyl)-pyrrolidine-2-carboxylate $\varvec{(2S,1S)}$-**2v**

Pale-yellow wax; M.p. 92–$96\,^{\circ }\text {C}$; TLC (AcOEt): $R_\mathrm{f}$=0.42; $[\alpha ]_\mathrm{D}=-35.4$ ($c$ 0.813, $\text {CHCl}_{3})$; IR (KBr): 669, 766, 1155, 1202, 1458, 1508, 1541, 1653, 1738, 2876, 2964, 3200; $^{1}\text {H}$ NMR $(\text {CDCl}_{3}$, 300 MHz): $\delta$ 0.90 (d, $^{3}J=6.5$, 3H, $(\text {CH}(\text {C}H_{3})_{2})$, 0.96 (d, $^{3}J=6.5$, 3H, $(\text {C}(\text {C}H_{3})_{2}^{{\prime }})$, 1.14 (s, 3H, $\text {C}H_{3})$, 1.74 (m, 1H, H-4), 1.86 (m, 2H, H-3, H$'$-4), 2.02 (m, 2H, H$'$-3, $\text {C}H(\text {CH}_{3})_{2})$ 2.93 (m,$^{2}J=^{3}J_{1}=9.5$,$^{3}J_{2}=7.0$, 1H, H-5), 3.12 (m, $^{2}J=9.5$,$^{3}J_{1}=6.5$, $^{3}J_{2}=4.5$, 1H, H$'$-5), 3.69 (s, 3H, $\text {OC}H_{3})$, 3.74 (dd, $^{3}J_{1}=9.5$,$^{3}J_{2}=3.5$, 1H, H-2), 5.33 (bs, 1H, $\text {CON}H)$, 6.55 (bs, 1H, $\text {CON}H'$); $^{13}\text {C}$ NMR $(\text {CDCl}_{3}$, 75 MHz): $\delta$ 14.1 $(C\text {H}_{3})$, 18.0, 18.4 (2xCH$(C\text {H}_{3})_{2})$, 24.8 (C-4), 32.0 (C-3), 34.2 $(C\text {H}(\text {CH}_{3})_{2})$, 49.7 (C-5), 51.8 $(\text {O}C\text {H}_{3})$, 59.8 (C-2), 69.1 (C-1), 175.8 $(C\text {ONH})$, 177.3 $(C\text {OOCH}_{3})$; HRMS (ESI+) calcd for $\text {C}_{12}\text {H}_{22}\text {N}_{2}\text {O}_{3}\text {Na}$: 265.1528 (M+Na)$^{+}$ found 265.1533.

#### General procedure for NaOH-mediated cyclocondensation of amidoesters **2v** and **2x**

To a stirred solution of the appropriate amidoester in absolute EtOH (5 mL per 0.1 mmol of substrate), NaOH (1 eq.) was added at room temperature. After dissolution of the hydroxide, the mixture was stirred for additional 10 min and quenched with saturated aqueous solution of ammonium chloride (80 mL). The resulting solution was extracted with DCM (3$\times$25 mL). The combined organic phase was washed with water (20 mL), dried over anhydrous $\text {MgSO}_{4}$, filtered and concentrated under reduced pressure. The residue was purified by FC.

#### $\varvec{(4R,8}\mathbf{a}\varvec{S)}$-4-Isopropyl-4-methylperhydropyrrolo[1,2-$a$]pyrazine-1,3-dione $\varvec{(4R,8}\mathbf{a}\varvec{S)}$-**3v**

From $$\varvec{(2S,1R)}$$-**2v** (22 mg, 0.09 mmol) and NaOH (4 mg, 1eq.); FC (gradient: PE/AcOEt 4:1 to 2:1): yield 16 mg (86 %). Pale-yellow wax, M.p. $$65=-70\,^{\circ }\text {C}$$; TLC (PE/AcOEt 3:1): $$R_\mathrm{f}=0.35$$; $$[\alpha ]_\mathrm{D}=+3.6$$ ($$c $$0.893, $$\text {CHCl}_{3})$$; IR (KBr): 1246, 1373, 1460, 1701, 2851, 2924, 2964, 3101, 3234; $$^{1}\text {H}$$ NMR (500 MHz, CDCl$$_{3})$$: $$\delta $$ 1.08 (d, $$^{3}J=6.5$$, 3H, $$\text {CH}(\text {C}H_{3})_{2})$$, 1.09 (d, $$^{3}J=6.5$$, 3H, $$\text {CH}(\text {C}H_{3})_{2}'$$), 1.30 (s, 3H, $$\text {C}H_{3})$$, 1.64–1.91 (m, 3H, H-7, H-7$$'$$, H-8), 2.02–2.25 (m, 2H, H$$'$$-8, $$(\text {C}H(\text {CH}_{3})_{2})$$, 2.69 (m, $$^{2}J=^{3}J_{1}=8.5$$, $$^{3}J_{2}=7.0$$, 1H, H-6), 2.77 (m, $$^{2}J=^{3}J_{1}=8.5$$, $$^{3}J_{2}$$=5.5, 1H, H$$'$$-6), 3.61 (pt, $$^{3}J=7.5$$, 1H, H-8a), 7.26 (bs, 1H, $$\text {N}H$$); $$^{13}\text {C}$$ NMR $$(\text {CDCl}_{3}$$, 125 MHz): $$\delta $$ 13.8 $$(C\text {H}_{3})$$, 17.7 (2xCH$$(C\text {H}_{3})_{2})$$, 21.9 (C-7), 25.7 (C-8), 34.4 $$(C\text {H}(\text {CH}_{3})_{2})$$, 45.3 (C-6), 59.1 (C-8a), 65.1 (C-4), 173.1 (C-3), 176.5 (C-1); HRMS (ESI-) calcd for $$\text {C}_{11}\text {H}_{17}\text {N}_{2}\text {O}_{2}$$: 209.1290 (M-H)$$^{-}$$ found 209.1281.

#### $\varvec{(4S,8}\mathbf{a}\varvec{S)}$-4-Isopropyl-4-methylperhydropyrrolo[1,2-$a$]pyrazine-1,3-dione $\varvec{(4S,8}\mathbf{a}\varvec{S)}$-**3v**

From $$\varvec{(2S,1S)}$$-**2v** (85 mg, 0.35 mmol) and NaOH (14 mg, 1eq.); FC (gradient: PE/AcOEt 4:1 to 2:1): yield 60 mg (82 %). White wax; M.p. 107–$$110\,^{\circ }\text {C}$$; TLC (PE/AcOEt 3:1): $$R_\mathrm{f}=0.32$$; $$[\alpha ]_\mathrm{D}=-28.4$$ ($$c $$0.758, $$\text {CHCl}_{3})$$; IR (KBr): 1261, 392, 1458, 1508, 1541, 1705, 2820, 2920, 2974, 3157; $$^{1}\text {H}$$ NMR (500 MHz, CDCl$$_{3})$$: $$\delta $$ 0.91 (d, $$^{3}J=6.5$$, 3H, $$\text {CH}(\text {C}H_{3})_{2})$$, 1.01 (d, $$^{3}J=6.5$$, 3H, $$\text {CH}(\text {C}H_{3})_{2}'$$), 1.22 (s, 3H, $$\text {C}H_{3})$$, 1.75–1.87 (m, 2H, H-7, H$$'$$-7), 2.07 (m,$$^{3}J=6.5$$, 1H, $$\text {C}H(\text {CH}_{3})_{2})$$, 2.26 (m, 2H, H-8, H$$'$$-8), 2.41 (pq, $$^{2}J=^{3}J_{1}=^{3}J_{2}=9.0$$, 1H, H-6), 3.13 (m, $$^{2}J=9.0$$, $$^{3}J_{1}$$=7.5, $$^{3}J_{2}=3.5$$, 1H, H$$'$$-6), 3.95 (pt, $$^{3}J_{1}=^{3}J_{2}=5.5$$, 1H, H-8a), 7.84 (bs, 1H, $$\text {N}H$$); $$^{13}\text {C}$$ NMR $$(\text {CDCl}_{3}$$, 125 MHz): $$\delta $$ 13.9 $$(C\text {H}_{3})$$, 16.1, 17.5 (2xCH$$(C\text {H}_{3})_{2})$$, 22.3 (C-7), 28.2 (C-8), 32.6 $$(C\text {H}(\text {CH}_{3})_{2})$$, 48.2 (C-6), 58.5 (C-8a), 65.4 (C-4), 175.1 (C-3), 175.8 (C-1); HRMS (ESI-) calcd for $$\text {C}_{11}\text {H}_{17}\text {N}_{2}\text {O}_{2}$$: 209.1290 (M-H)$$^{-}$$ found 209.1297.

#### $\varvec{(4R,8}\mathbf{a}\varvec{S)}$-2-Ethoxymethyl-4-isopropyl-4-methylperhydropyrrolo[1,2-$a$]pyrazine-1,3-dione $\varvec{(4R,8}\mathbf{a}\varvec{S)}$-**3x**

From $$\varvec{(2S,1R)}$$-**1x** (107 mg, 0.26 mmol) and NaOH (10 mg, 1eq.); FC (gradient: PE/AcOEt 8:1 to 3:1): yield 50 mg (68 %). Pale-yellow oil; TLC (PE/AcOEt 5:1): $$R_\mathrm{f}=0.38$$; $$[\alpha ]_\mathrm{D}=+11.1$$ ($$c $$0.717, $$\text {CHCl}_{3})$$; IR (KBr): 1101, 1242, 1296, 1344, 1373, 1454, 1508, 1693, 1738, 2879, 2930, 2972, 3369; $$^{1}\text {H}$$ NMR (500 MHz, CDCl$$_{3})$$: $$\delta $$ 1.04 (d, $$^{3}J=7.0$$, 3H, $$\text {CH}(\text {C}H_{3})_{2})$$, 1.09 (d, $$^{3}J=7.0$$, 3H, $$\text {CH}(\text {C}H_{3})_{2}'$$), 1.17 (t,$$^{3}J=7.0$$, 3H, $$\text {CH}_{2}\text {O}\text {CH}_{2}\text {C}H_{3})$$, 1.29 (s, 3H, $$\text {C}H_{3})$$, 1.75–1.88 (m, 2H, H-7, H-7$$'$$), 2.10–2.24 (m, 3H, H-8, H$$'$$-8, $$(\text {C}H(\text {CH}_{3})_{2})$$, 2.67 (m, $$^{2}J=^{3}J_{1}=8.5$$, $$^{3}J_{2}=7.0$$, 1H, H-6), 2.81 (m, $$^{2}J=^{3}J_{1}=8.5$$, $$^{3}J_{2}=5.0$$, 1H, H$$'$$-6), 3.57 (m, 3H, H-8a, $$\text {CH}_{2}\text {O}\text {C}H_{2}\text {CH}_{3})$$, 5.19 (d, $$^{2}J=9.5$$, 1H, $$\text {C}H_{2}\text {OCH}_{2}\text {CH}_{3})$$, 5.23 (d, $$^{2}J=9.5$$, 1H, $$\text {C}H_{2}\text {OCH}_{2}\text {CH}_{3}'$$); $$^{13}\text {C}$$ NMR $$(\text {CDCl}_{3}$$, 125 MHz): $$\delta $$ 13.6 $$(C\text {H}_{3})$$, 15.1 $$(\text {CH}_{2}\text {OCH}_{2}C\text {H}_{3})$$, 17.6, 17.7 (2xCH$$(C\text {H}_{3})_{2})$$, 21.9 (C-7), 26.4 (C-8), 35.4 ($$C\text {H}(\text {CH}_{3})_{2})$$, 45.5 (C-6), 59.0 (C-8a), 65.3 (C-4), 65.8 $$(\text {CH}_{2}\text {O}C\text {H}_{2}\text {CH}_{3})$$, 69.1 $$(C\text {H}_{2}\text {OCH}_{2}\text {CH}_{3})$$, 173.1 (C-3), 176.4 (C-1); HRMS (ESI+) calcd for $$\text {C}_{14}\text {H}_{24}\text {N}_{2}\text {O}_{3}\text {Na}$$: 291.1685 (M+Na)$$^{+}$$ found 291.1682.

#### $\varvec{(4S,8}\mathbf{a}\varvec{S)}$-2-Ethoxymethyl-4-isopropyl-4-methylperhydropyrrolo[1,2-$a$]pyrazine-1,3-dione $\varvec{(4S,8}\mathbf{a}\varvec{S)}$-**3x**

From $$\varvec{(2S,1S)}$$-**1x** (85 mg, 0.21 mmol) and NaOH (9 mg, 1eq.); FC (gradient: PE/AcOEt 8:1 to 3:1): yield 31 mg (56 %). Pale-yellow oil; TLC (PE/AcOEt 5:1): $$R_\mathrm{f}=0.35$$; $$[\alpha ]_\mathrm{D}=-14.8$$ ($$c $$0.483, $$\text {CHCl}_{3})$$; IR (KBr): 1095, 1246, 1342, 1456, 1522, 1541, 1686, 1732, 2808, 2880, 2930, 2974, 3368; $$^{1}\text {H}$$ NMR (500 MHz, CDCl$$_{3})$$: $$\delta $$ 0.85 (d, $$^{3}J=6.5$$, 3H, $$\text {CH}(\text {C}H_{3})_{2})$$, 1.00 (d, $$^{3}J=6.5$$, 3H, $$\text {CH}(\text {C}H_{3})_{2}'$$), 1.17 (t,$$^{3}J=7.0$$, 3H, $$\text {CH}_{2}\text {O}\text {CH}_{2}\text {C}H_{3})$$, 1.23 (s, 3H, $$\text {C}H_{3})$$, 1.75–1.87 (m, 2H, H-7, H$$'$$-7), 2.02 (m,$$^{3}J=6.5$$, 1H, $$\text {C}H(\text {CH}_{3})_{2})$$, 2.16 (m, 1H, H-8), 2.37 (m, 1H, H$$'$$-8), 2.42 (pq, $$^{2}J=^{3}J_{1}=^{3}J_{2}=9.0$$, 1H, H-6), 3.12 (m, $$^{2}J=9.0$$, $$^{3}J_{1}=7.5$$, $$^{3}J_{2}=3.5$$, 1H, H$$'$$-6), 3.57 (m, 2H, $$\text {CH}_{2}\text {OC}H_{2}\text {CH}_{3})$$, 4.05 (dd, $$^{3}J_{1}=9.0$$, $$^{3}J_{2}=2.5$$, 1H, H-8a), 5.15 (d, $$^{2}J=9.5$$, 1H, $$\text {C}H_{2}\text {OCH}_{2}\text {CH}_{3})$$, 5.30 (d, $$^{2}J=9.5$$, 1H, $$\text {C}H_{2}\text {OCH}_{2}\text {CH}_{3}'$$); $$^{13}\text {C}$$ NMR $$(\text {CDCl}_{3})$$, 125 MHz): $$\delta $$ 14.5 $$(C\text {H}_{3})$$, 15.2 $$(\text {CH}_{2}\text {OCH}_{2}C\text {H}_{3})$$, 16.0, 17.3 (2xCH$$(C\text {H}_{3})_{2})$$, 22.7 (C-7), 29.8 (C-8), 32.7 ($$C\text {H}(\text {CH}_{3})_{2})$$, 48.7 (C-6), 58.8 (C-8a), 65.2 (C-4), 65.5 $$(\text {CH}_{2}\text {O}C\text {H}_{2}\text {CH}_{3})$$, 68.8 $$(C\text {H}_{2}\text {OCH}_{2}\text {CH}_{3})$$, 175.1 (C-3), 175.5 (C-1); HRMS (ESI+) calcd for $$\text {C}_{14}\text {H}_{24}\text {N}_{2}\text {O}_{3}\text {Na}$$: 291.1685 (M+Na)$$^{+}$$ found 291.1677.

## Electronic supplementary material

Below is the link to the electronic supplementary material.

Supplementary material 1 (pdf 3269 KB)

## Footnotes

1 In cases where either no U-5C-4CR products were formed or low yields were observed, the starting materials could be recovered from the post-condensation mixtures. No side products were detected.

2 The differences in the $^{1}\text {H}$ and $^{13}\text {C}$ NMR spectra of the respective diastereomers of **1** were not consistent and did not allow for easy differentiation. Therefore, the conversion to the corresponding cyclic forms was necessary in all cases. Determination of absolute chemistry of compounds **1w**, **1y**, **1z**, for which the diastereoinduction was negligible, was not performed.

3
$(2S,1S)$-**1u** could not be isolated from the post-reaction mixture, most probably due to its full conversion to $(4S,8\text {a}S)$-**3u**.

4
$\varvec{(2S,1S)}$-**2u** could not be isolated from the post-reaction mixture, most probably due to its full conversion to $\varvec{(4S,8}\mathbf{a}\varvec{S)}$-**3u**.
