# Supplementary material for: Expanding the substrate scope of ugi five-center, four-component reaction U-5C-4CR): ketones as coupling partners for secondary amino acids
Source: Mol Divers. 2013 Oct 24;18(1):61–77. doi: 10.1007/s11030-013-9488-0 (PMC3906574; doi:10.1007/s11030-013-9488-0)
Supplement: Supplementary file 1 — Supplementary material 1 (pdf 3269 KB) [file 11030_2013_9488_MOESM1_ESM.pdf]

NMR spectra of synthesized compounds

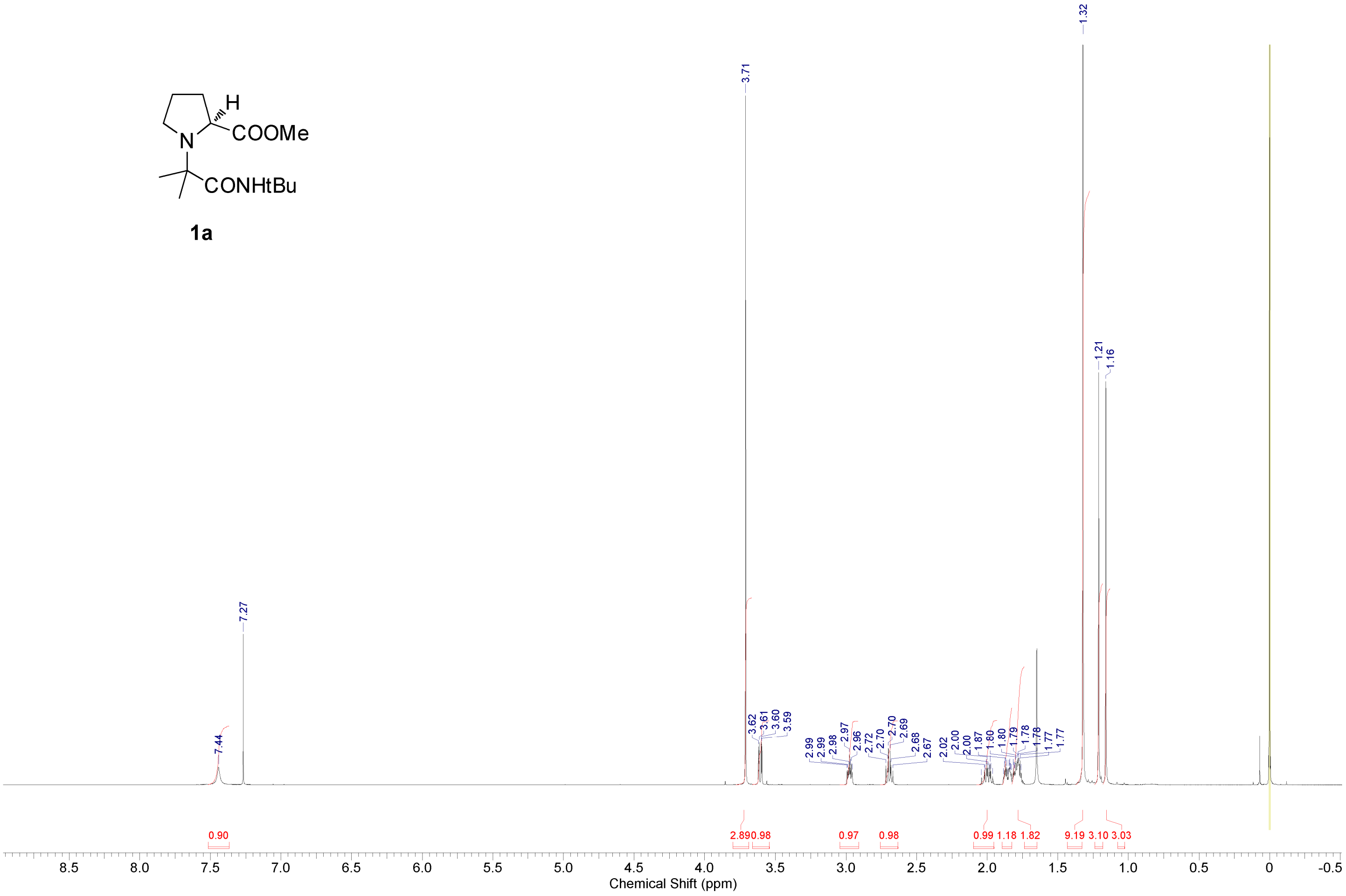
H
COOMe
N
CONHtBu
1a
7.44
7.27
3.71
3.62
3.61
3.60
3.59
2.99
2.99
2.98
2.97
2.96
2.72
2.70
2.70
2.69
2.68
2.67
2.02
2.00
2.00
1.87
1.80
1.80
1.79
1.78
1.78
1.77
1.77
1.32
1.21
1.16
0.90
2.89
0.98
0.97
0.98
0.99
1.18
1.82
9.19
3.10
3.03
8.5 8.0 7.5 7.0 6.5 6.0 5.5 5.0 4.5 4.0 3.5 3.0 2.5 2.0 1.5 1.0 0.5 0 -0.5
Chemical Shift (ppm)

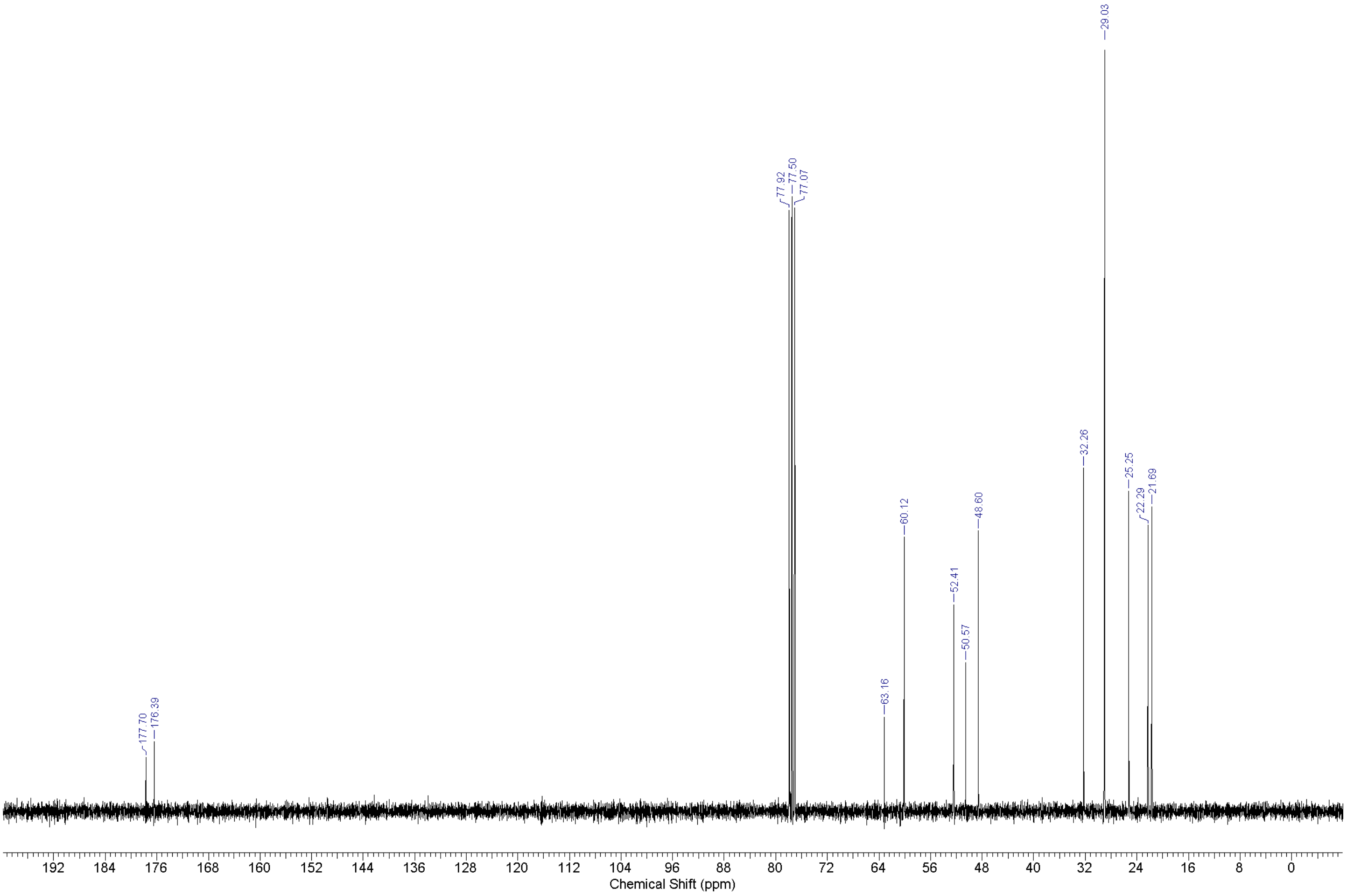
177.70
176.39
77.92
77.50
77.07
63.16
60.12
52.41
50.57
48.60
32.26
29.03
25.25
22.29
21.69
192 184 176 168 160 152 144 136 128 120 112 104 96 88 80 72 64 56 48 40 32 24 16 8 0
Chemical Shift (ppm)

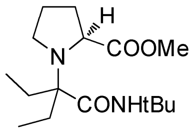
H
COOMe
N
CONHtBu

1b

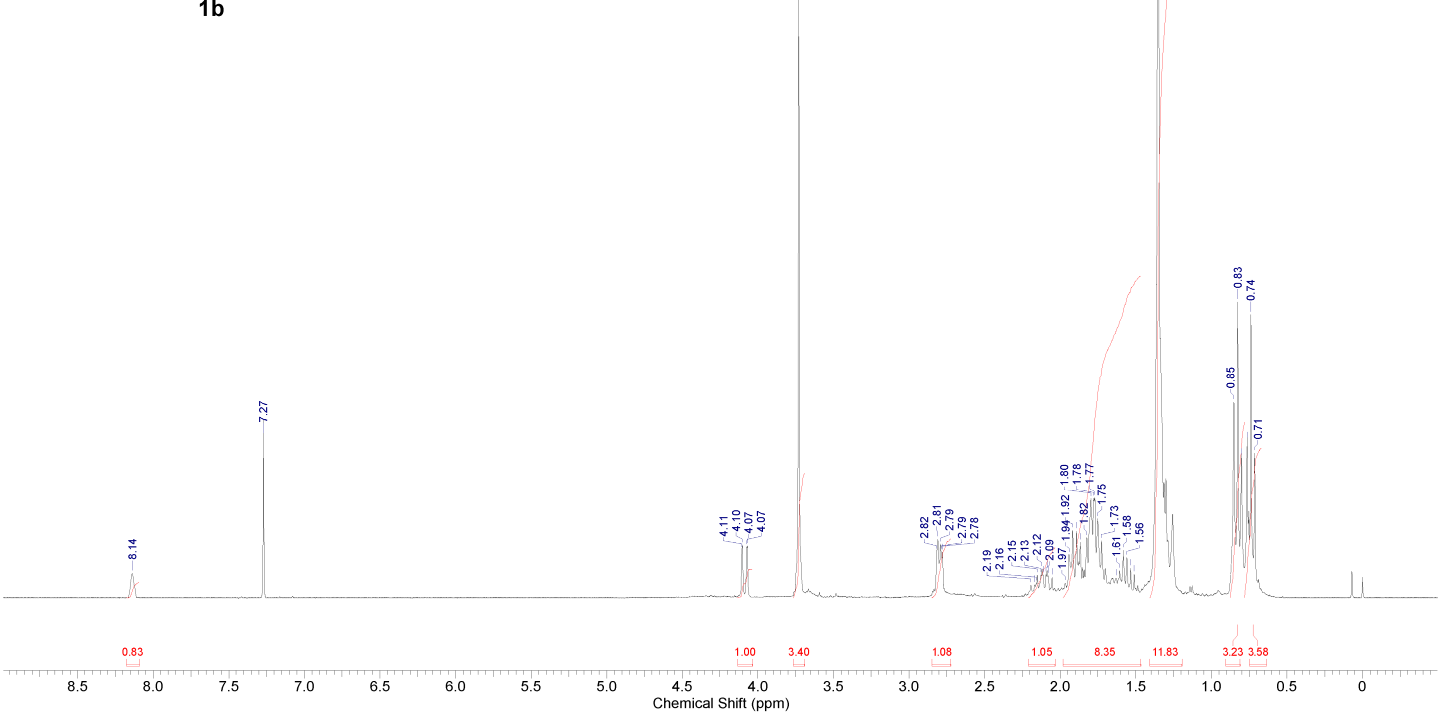
8.14
7.27
4.11 4.10 4.07 4.07
3.73
2.82 2.81 2.79 2.79 2.78
2.19 2.16 2.15 2.13 2.12 2.09
1.97 1.94 1.92 1.80 1.78 1.82 1.77 1.75 1.73 1.61 1.58 1.56
1.35
0.85 0.83 0.74 0.71
0.83 1.00 3.40 1.08 1.05 8.35 11.83 3.23 3.58
8.5 8.0 7.5 7.0 6.5 6.0 5.5 5.0 4.5 4.0 3.5 3.0 2.5 2.0 1.5 1.0 0.5 0
Chemical Shift (ppm)

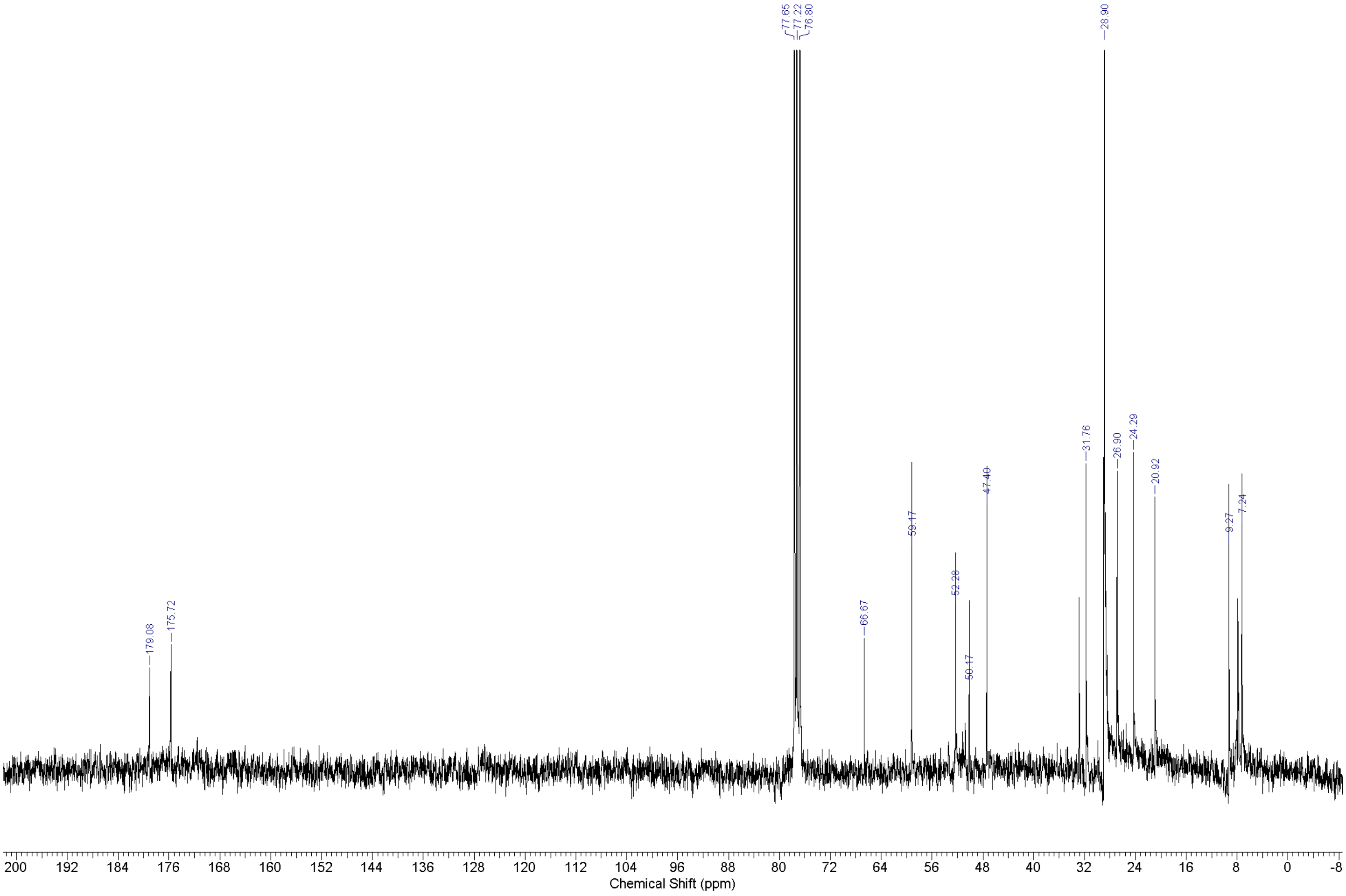
179.08
175.72
77.65 77.22 76.80
66.67
59.17
52.28
50.17
47.40
31.76
28.90
26.90
24.29
20.92
9.27
7.24
200 192 184 176 168 160 152 144 136 128 120 112 104 96 88 80 72 64 56 48 40 32 24 16 8 0 -8
Chemical Shift (ppm)

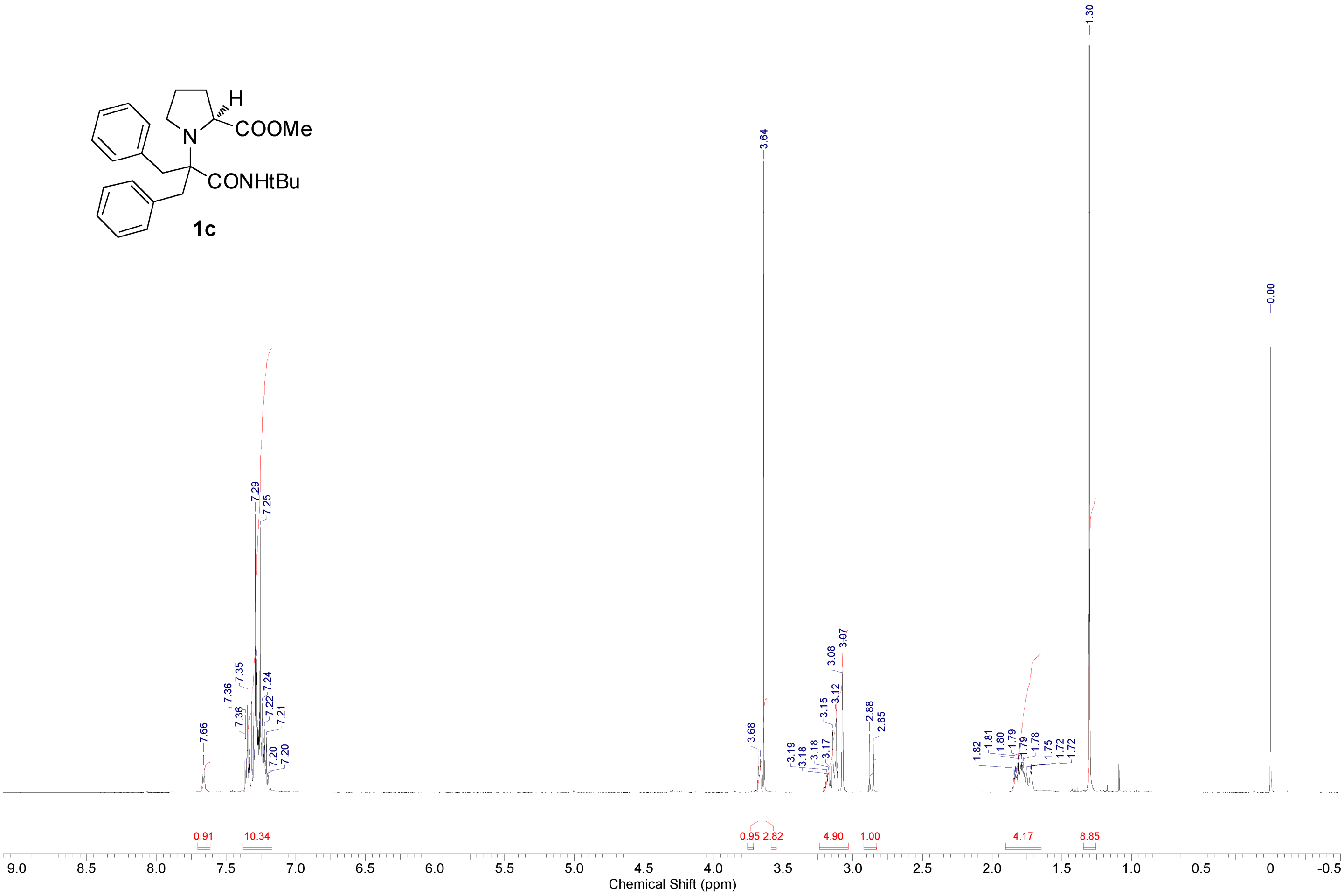

H
COOMe
N
CONHtBu
1c
7.66
7.36
7.36
7.35
7.29
7.25
7.24
7.22
7.21
7.20
7.20
3.68
3.64
3.19
3.18
3.18
3.17
3.15
3.12
3.08
3.07
2.88
2.85
1.82
1.81
1.80
1.79
1.79
1.78
1.75
1.72
1.72
1.30
0.00
0.91
10.34
0.95
2.82
4.90
1.00
4.17
8.85
Chemical Shift (ppm)

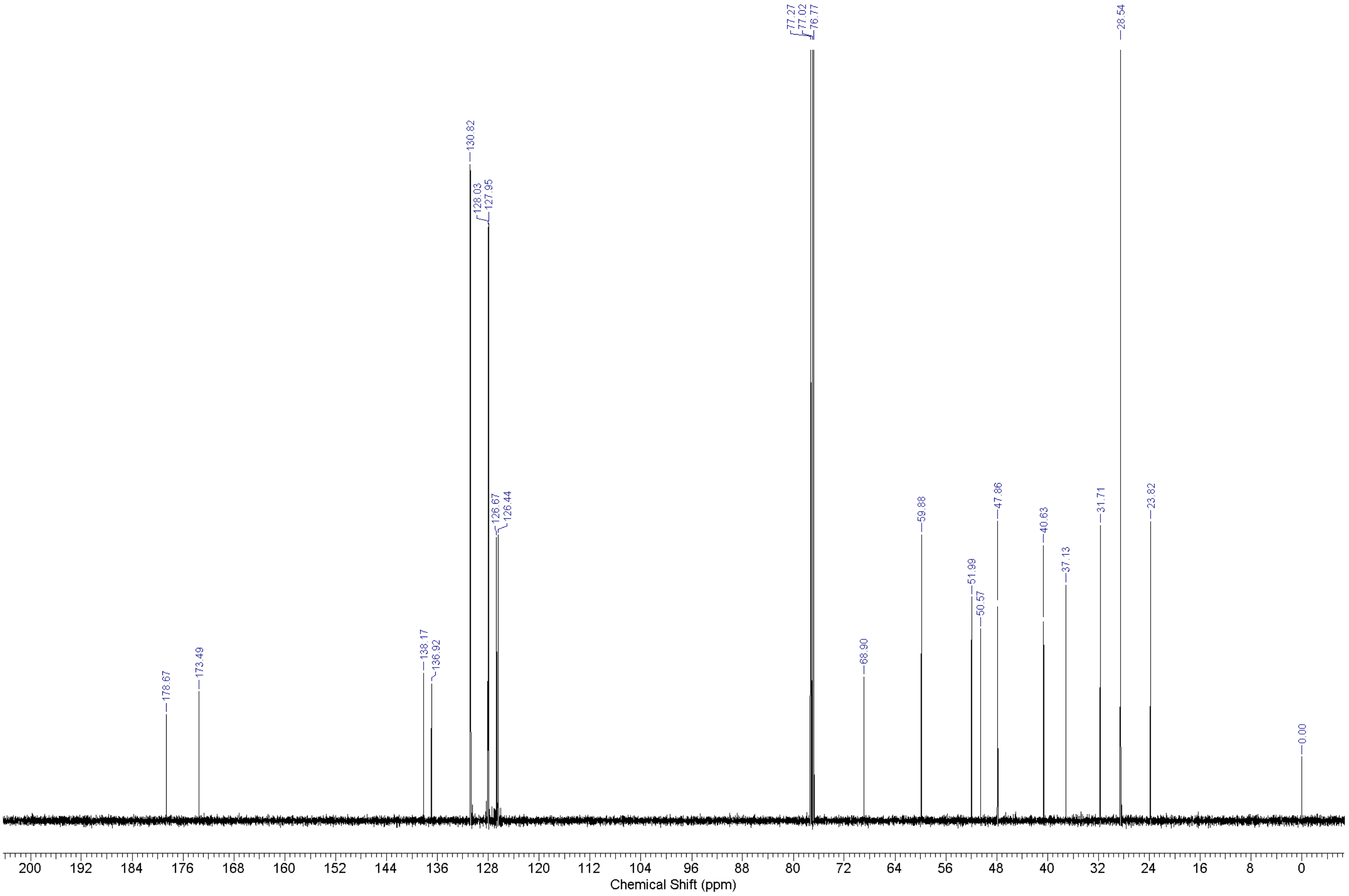

178.67
173.49
138.17
136.92
130.82
128.03
127.95
126.67
126.44
77.27
77.02
76.77
68.90
59.88
51.99
50.57
47.86
40.63
37.13
31.71
28.54
23.82
0.00
Chemical Shift (ppm)

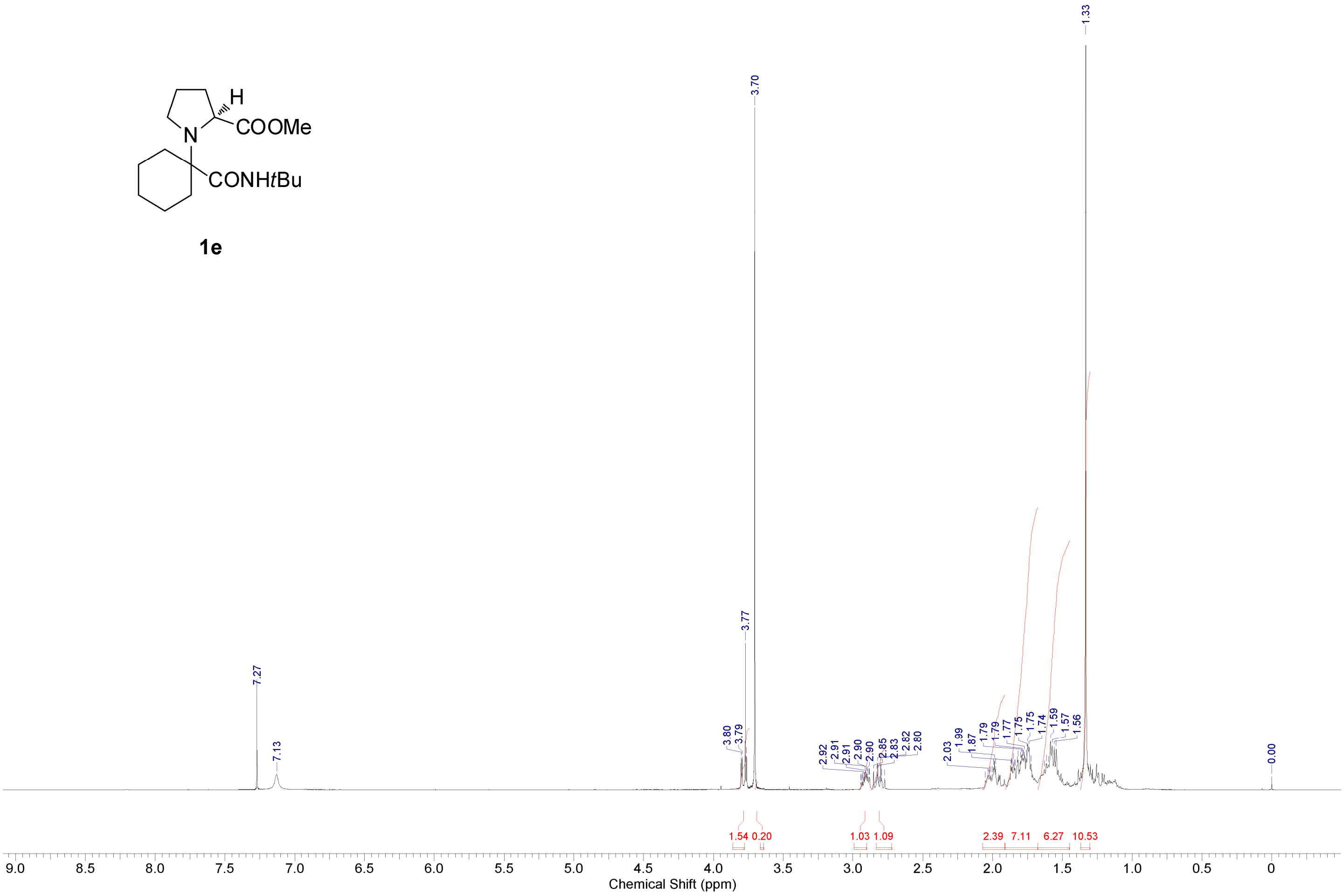
H
N
COOMe
CONHtBu

1e

1.33
3.70
3.77
7.27
7.13
3.80
3.79
2.92
2.91
2.91
2.90
2.90
2.85
2.83
2.82
2.80
2.03
1.99
1.87
1.79
1.79
1.77
1.75
1.74
1.59
1.57
1.56
0.00
1.54
0.20
1.03
1.09
2.39
7.11
6.27
10.53
Chemical Shift (ppm)

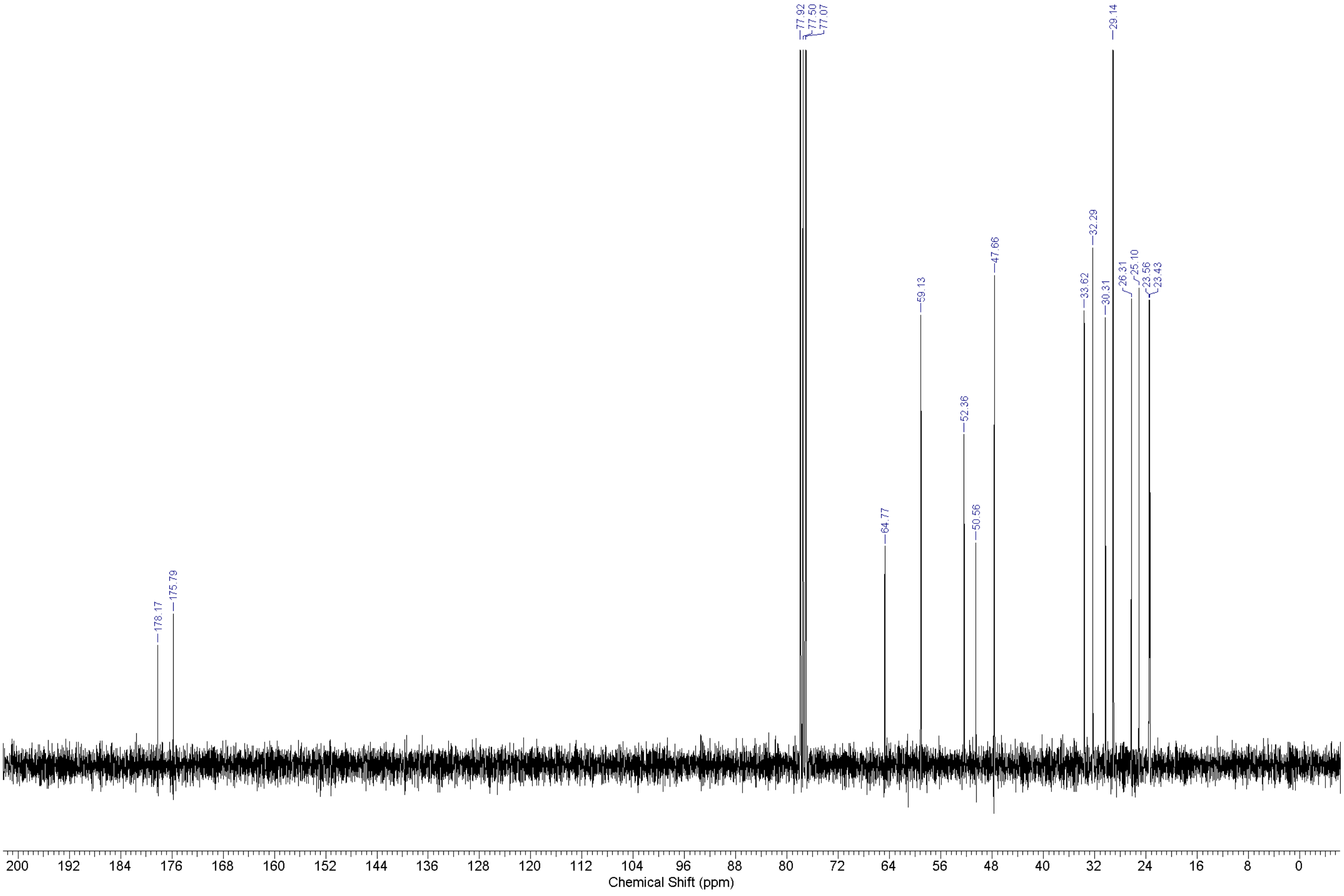
77.92
77.50
77.07
29.14
32.29
47.66
33.62
30.31
26.31
25.10
23.56
23.43
59.13
52.36
64.77
50.56
178.17
175.79
Chemical Shift (ppm)

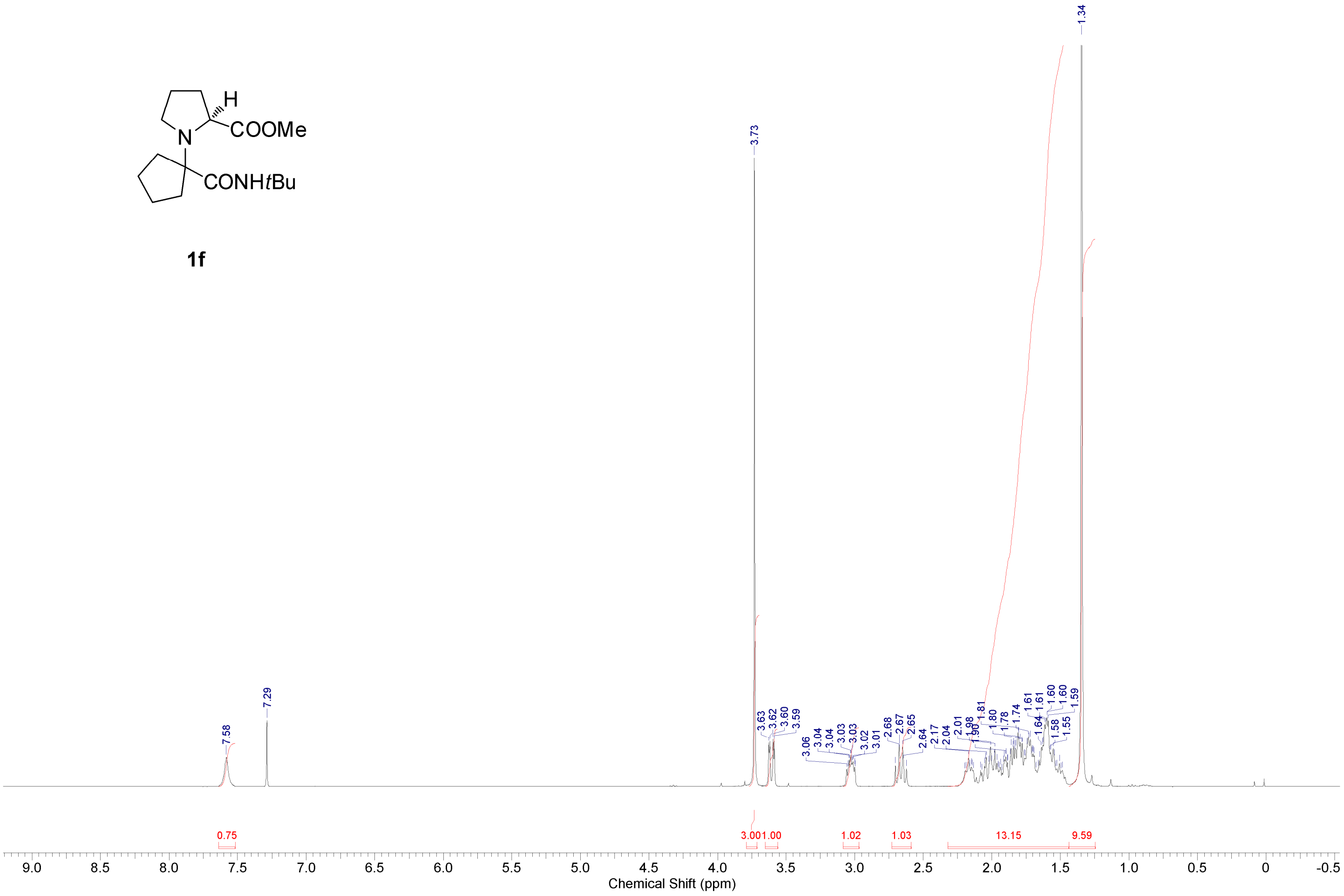
H
COOMe
N
CONHtBu
1f
7.58
7.29
3.73
3.63
3.62
3.60
3.59
3.06
3.04
3.03
3.03
3.02
3.01
2.68
2.67
2.65
2.64
2.17
2.04
2.01
1.98
1.96
1.81
1.80
1.78
1.74
1.64
1.61
1.61
1.60
1.60
1.59
1.58
1.55
1.34
0.75
3.00
1.00
1.02
1.03
13.15
9.59
Chemical Shift (ppm)

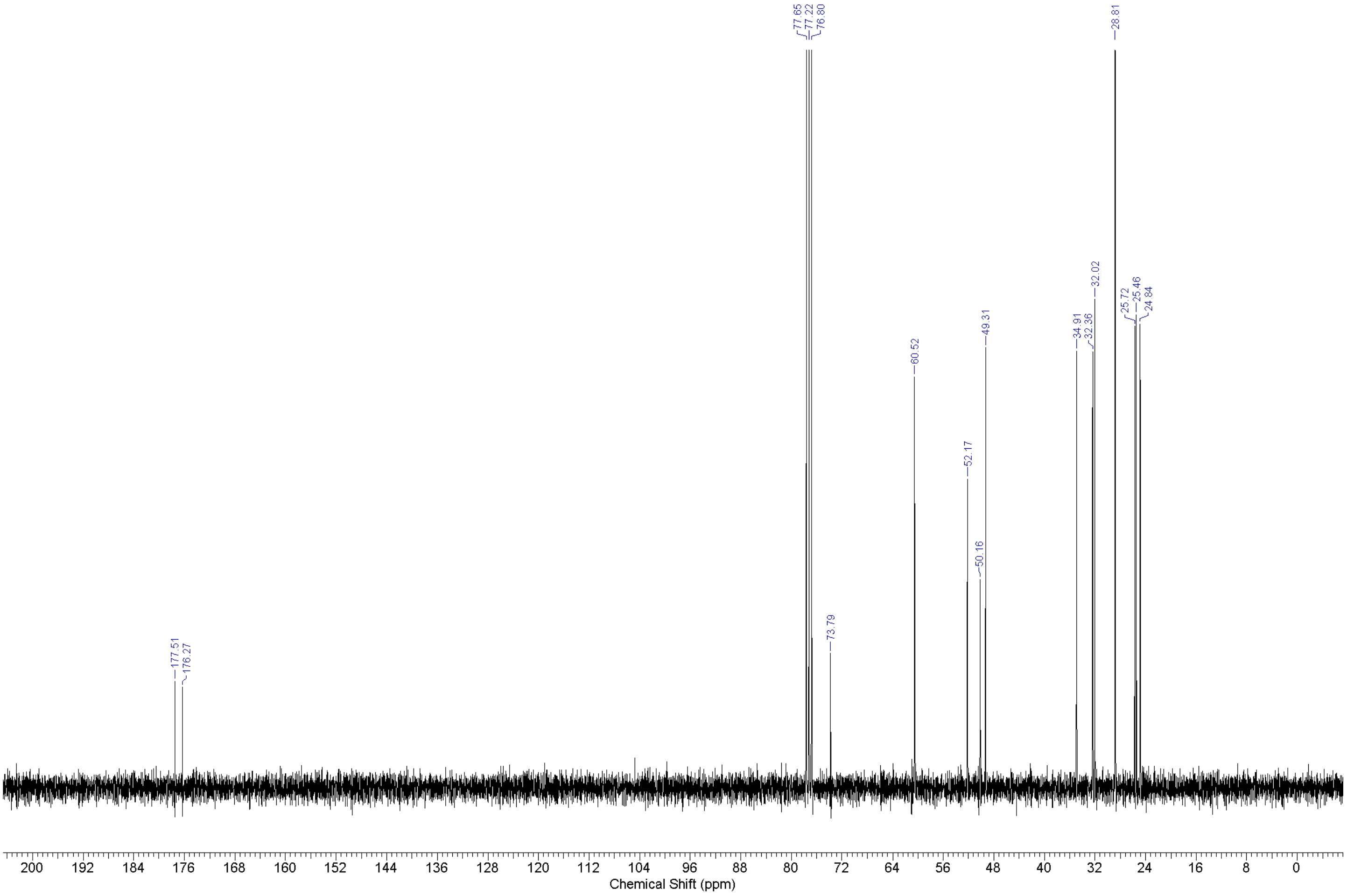
177.51
176.27
77.65
77.22
76.80
73.79
60.52
52.17
50.16
49.31
34.91
32.36
32.02
28.81
25.72
25.46
24.84
Chemical Shift (ppm)

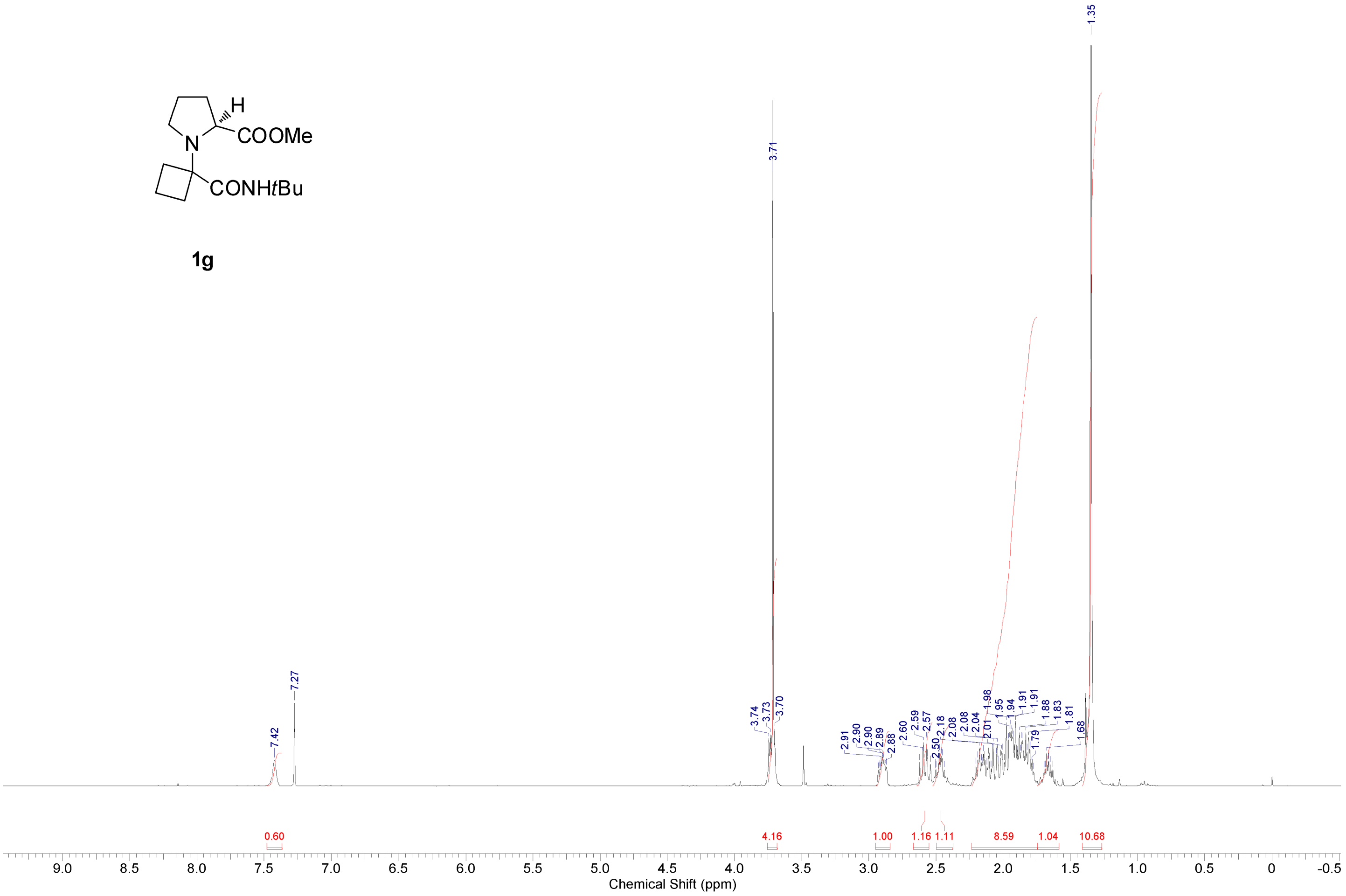
H
COOMe
N
CONHtBu
1g
1.35
3.71
7.27
7.42
3.74
3.73
3.70
0.60
4.16
1.00
1.16
1.11
8.59
1.04
10.68
Chemical Shift (ppm)

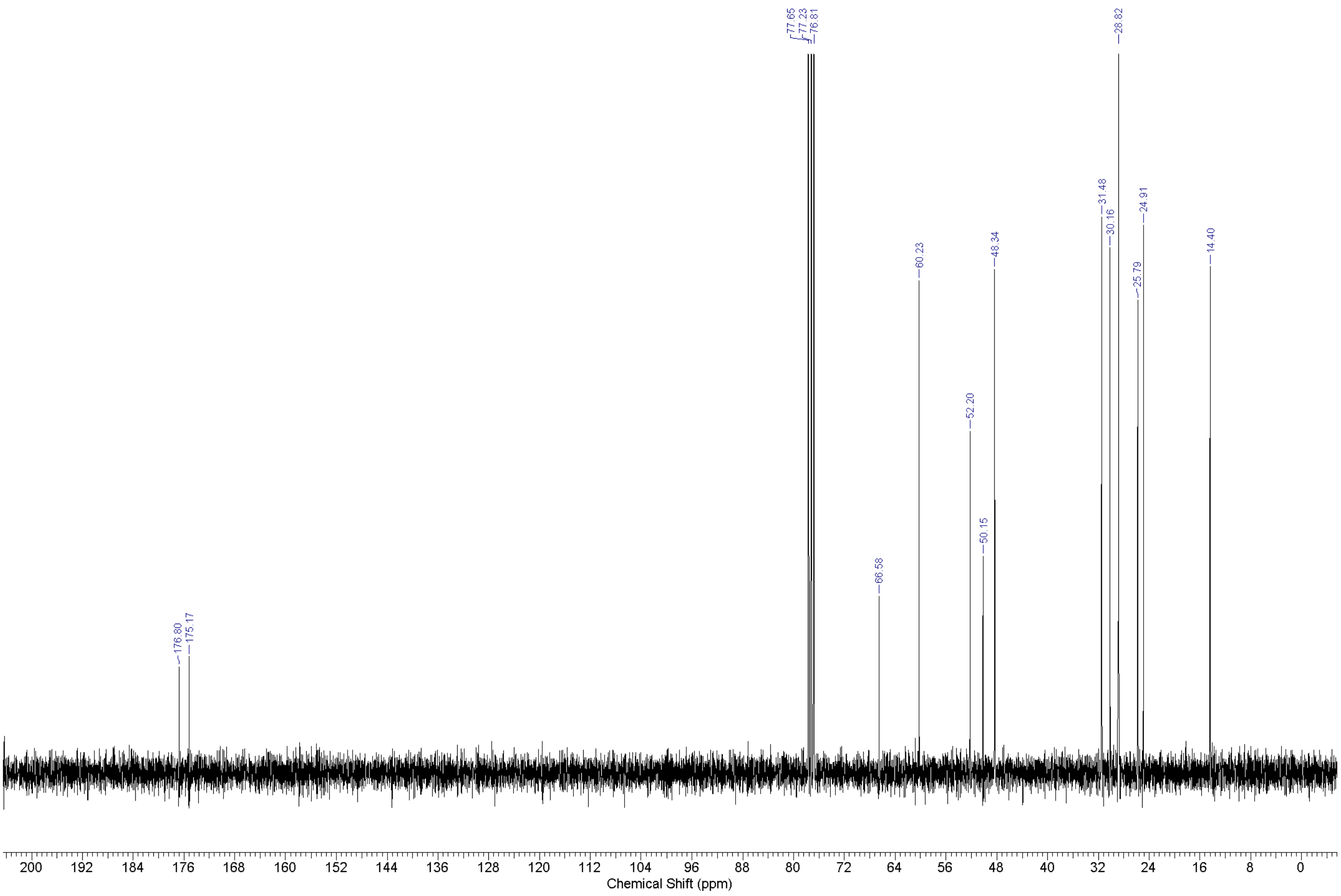
176.80
175.17
77.65
77.23
76.81
66.58
60.23
52.20
50.15
48.34
31.48
30.16
28.82
25.79
24.91
14.40
Chemical Shift (ppm)

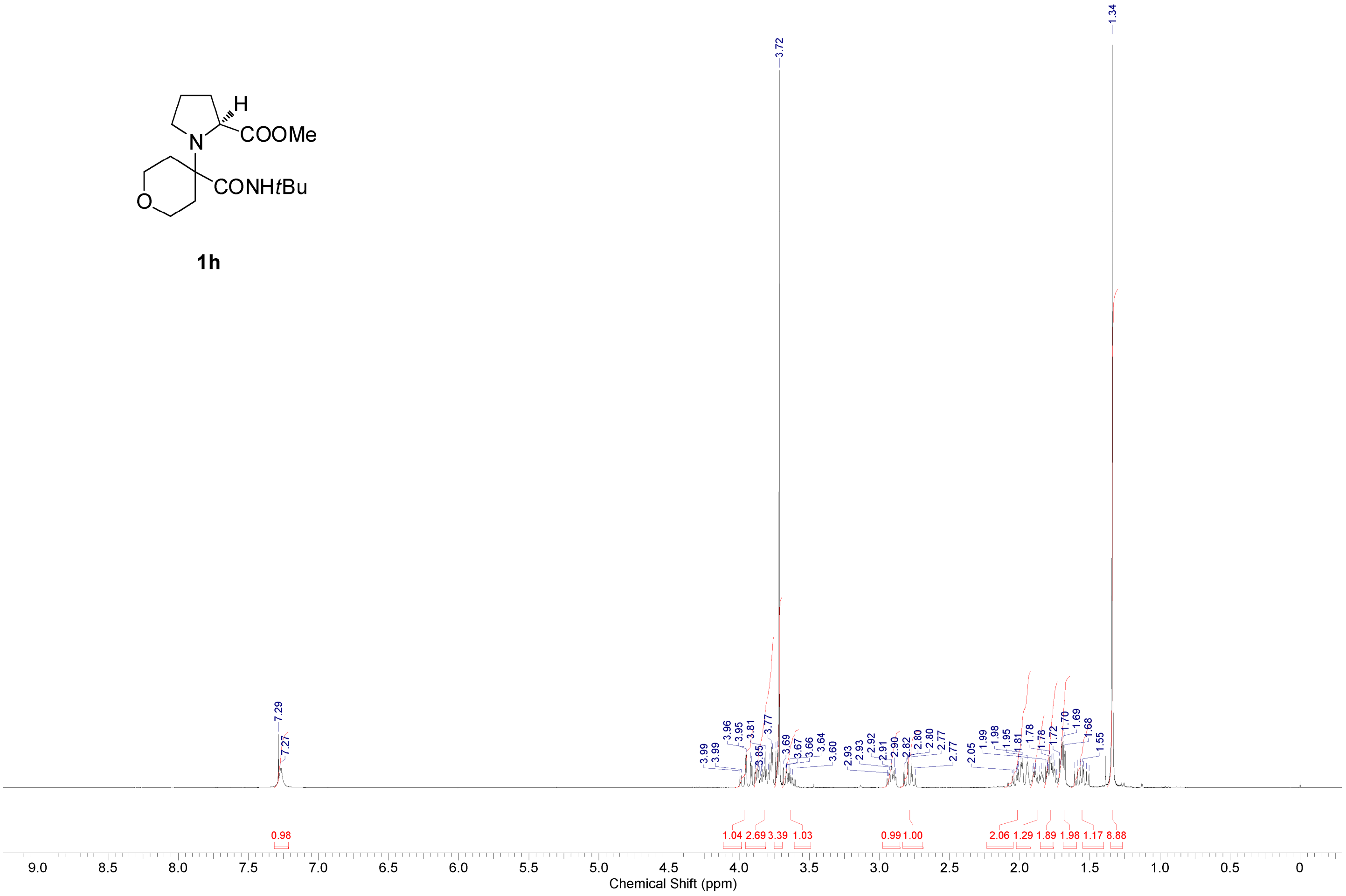
H
COOMe
N
O
CONHtBu
1h
7.29
7.27
3.99
3.99
3.96
3.95
3.95
3.85
3.81
3.77
3.72
3.69
3.67
3.66
3.64
3.60
2.93
2.93
2.92
2.91
2.90
2.82
2.80
2.80
2.77
2.77
2.05
1.99
1.98
1.95
1.81
1.78
1.72
1.70
1.69
1.68
1.55
1.34
0.98
1.04
2.69
3.39
1.03
0.99
1.00
2.06
1.29
1.89
1.98
1.17
8.88
Chemical Shift (ppm)

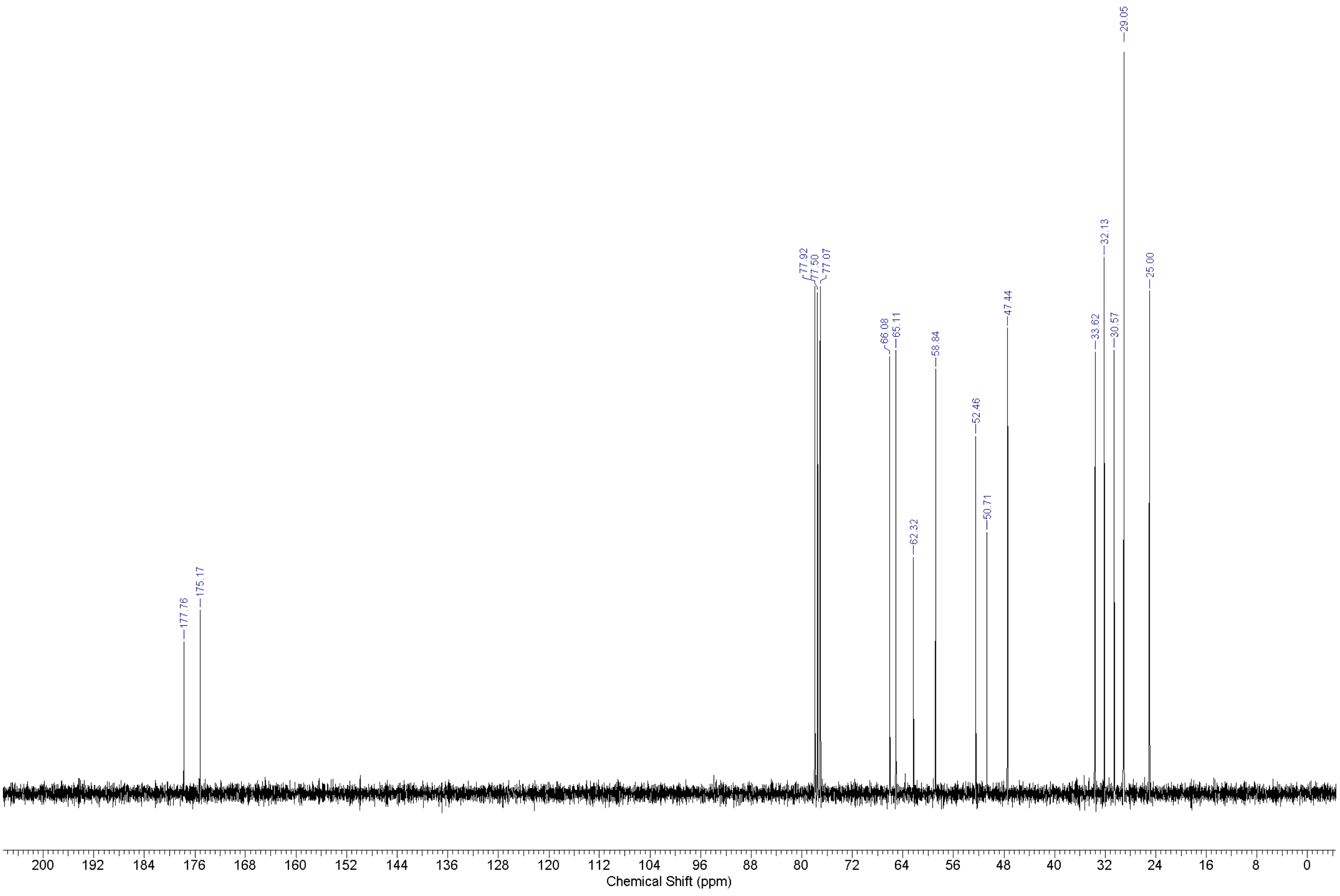
177.76
175.17
77.92
77.50
77.07
66.08
65.11
62.32
58.84
52.46
50.71
47.44
33.62
32.13
30.57
29.05
25.00
Chemical Shift (ppm)

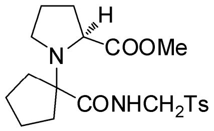
H
COOMe
N
CONHCH2Ts

1i

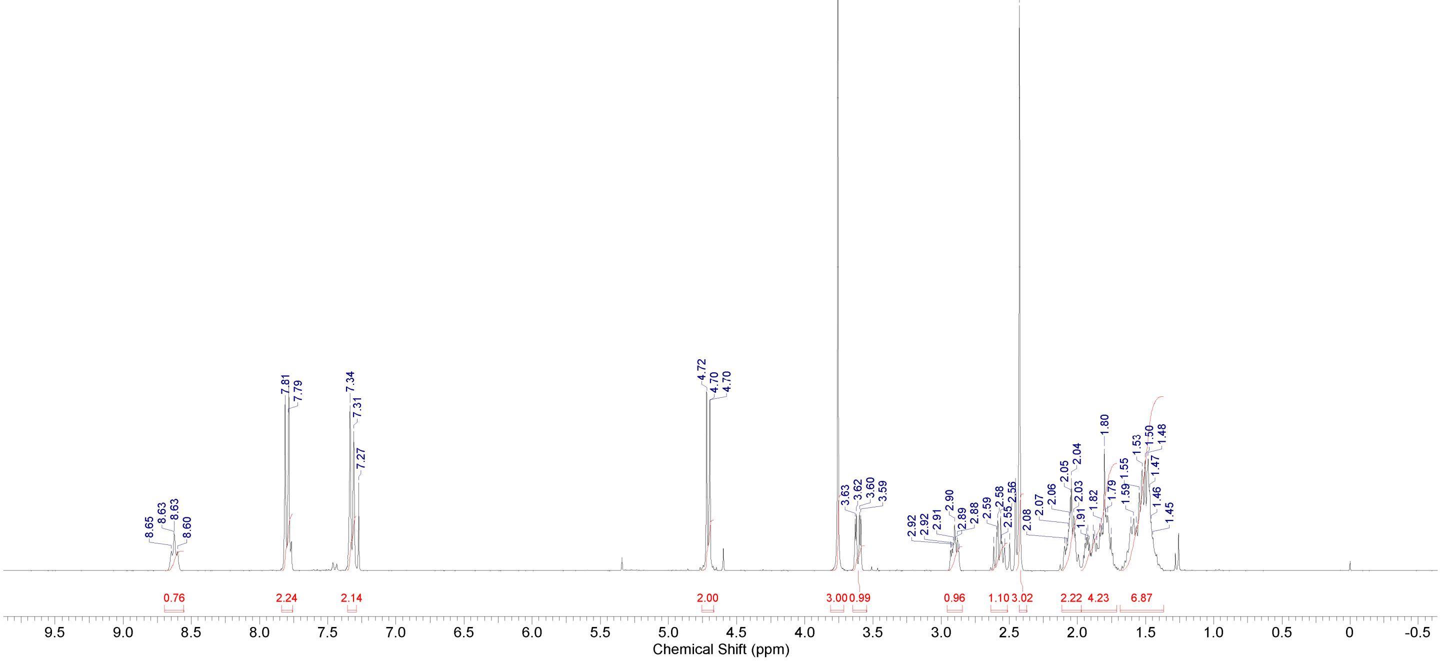
3.76
2.43
7.81
7.79
7.34
7.31
7.27
4.72
4.70
4.70
8.65
8.63
8.63
8.60
3.63
3.62
3.60
3.59
2.92
2.92
2.91
2.90
2.89
2.88
2.59
2.58
2.56
2.55
2.08
2.07
2.06
2.05
2.04
2.03
1.91
1.82
1.80
1.79
1.59
1.55
1.53
1.50
1.48
1.47
1.46
1.45
0.76
2.24
2.14
2.00
3.00
0.99
0.96
1.10
3.02
2.22
4.23
6.87
9.5 9.0 8.5 8.0 7.5 7.0 6.5 6.0 5.5 5.0 4.5 4.0 3.5 3.0 2.5 2.0 1.5 1.0 0.5 0 -0.5
Chemical Shift (ppm)

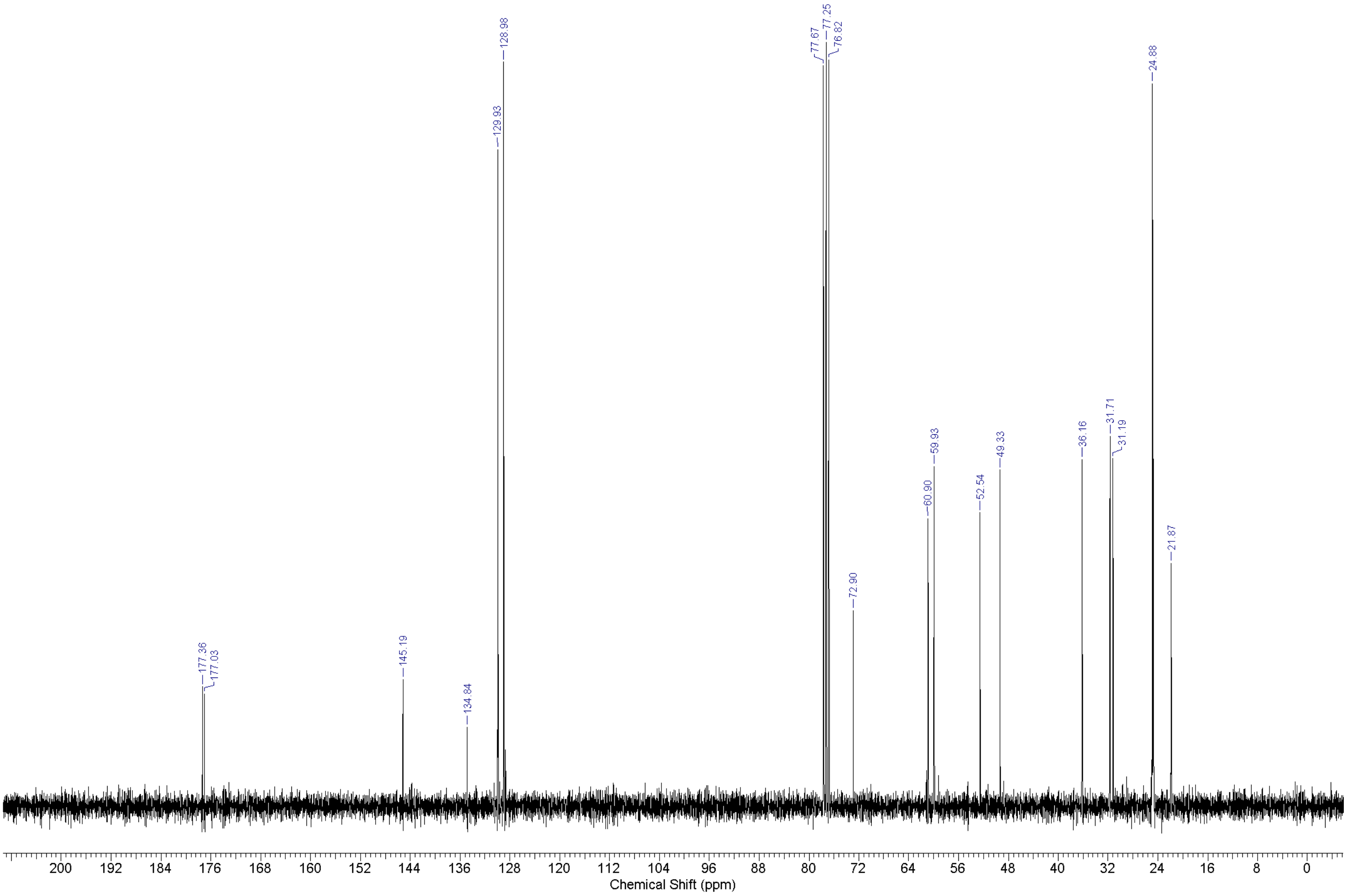
177.36
177.03
145.19
134.84
129.93
128.98
77.67
77.25
76.82
72.90
60.90
59.93
52.54
49.33
36.16
31.71
31.19
24.88
21.87
200 192 184 176 168 160 152 144 136 128 120 112 104 96 88 80 72 64 56 48 40 32 24 16 8 0
Chemical Shift (ppm)

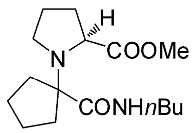
H
COOMe
N
CONH*n*Bu

1j

3.71
0.93
0.95
0.90
7.28
7.71 7.69 7.67
3.64 3.63 3.61 3.60 3.33 3.31 3.29 3.26 3.24 3.19 3.17 3.15 2.99 2.66 2.64 2.63 2.21 2.04 2.02 1.98 1.91 1.90 1.80 1.79 1.78 1.74 1.60 1.59 1.49 1.47 1.37 1.34 1.32
0.00
0.91 2.93 1.00 2.05 1.05 1.00 1.18 1.07 6.80 4.06 2.26 2.14 3.08
Chemical Shift (ppm)

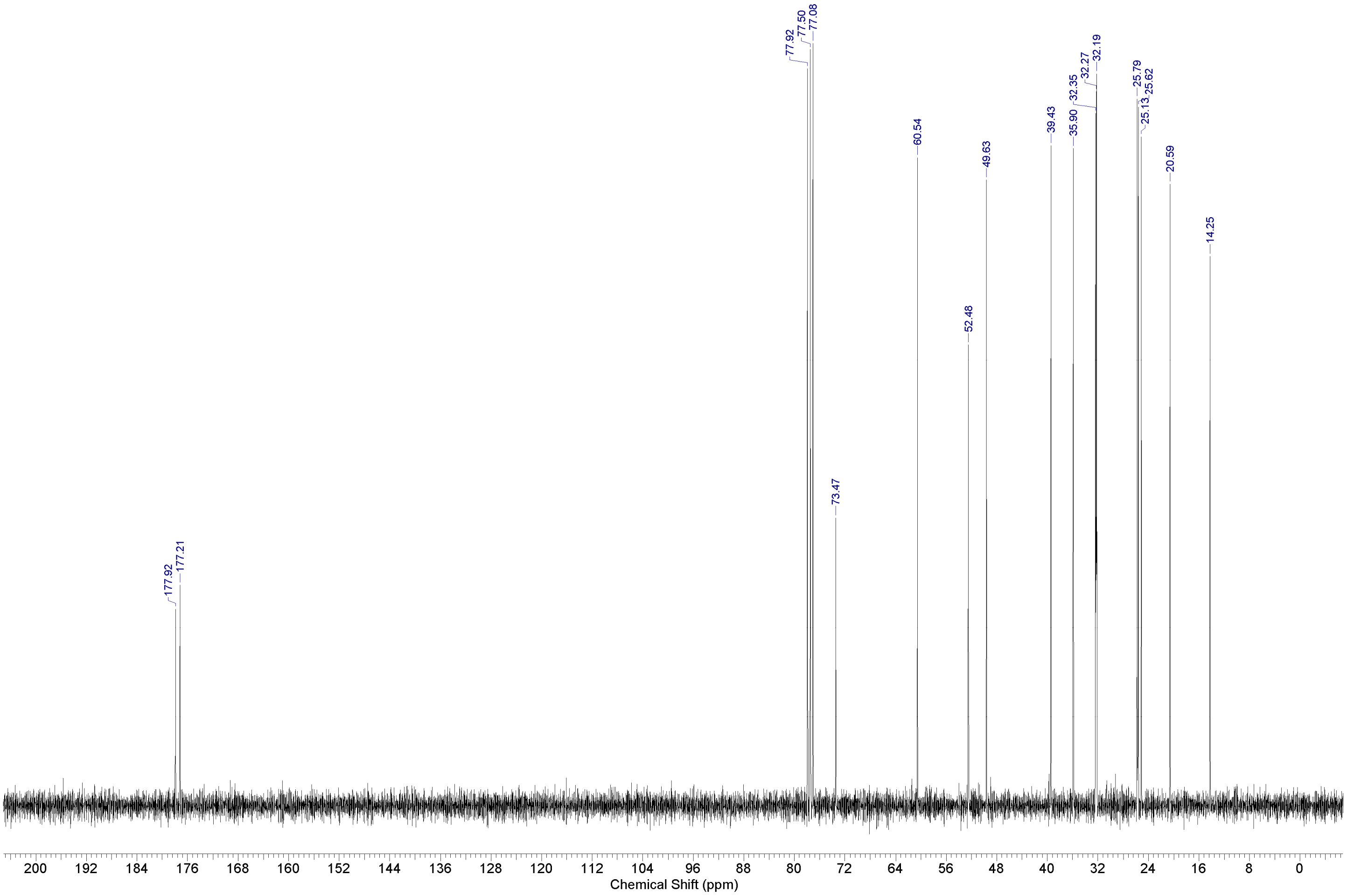
177.92 177.21
77.92 77.50 77.08
73.47
60.54
52.48
49.63
39.43
35.90 32.35 32.27 32.19
25.79 25.13 25.62
20.59
14.25
Chemical Shift (ppm)

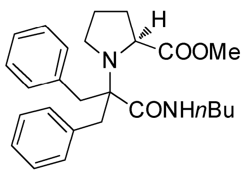

1k

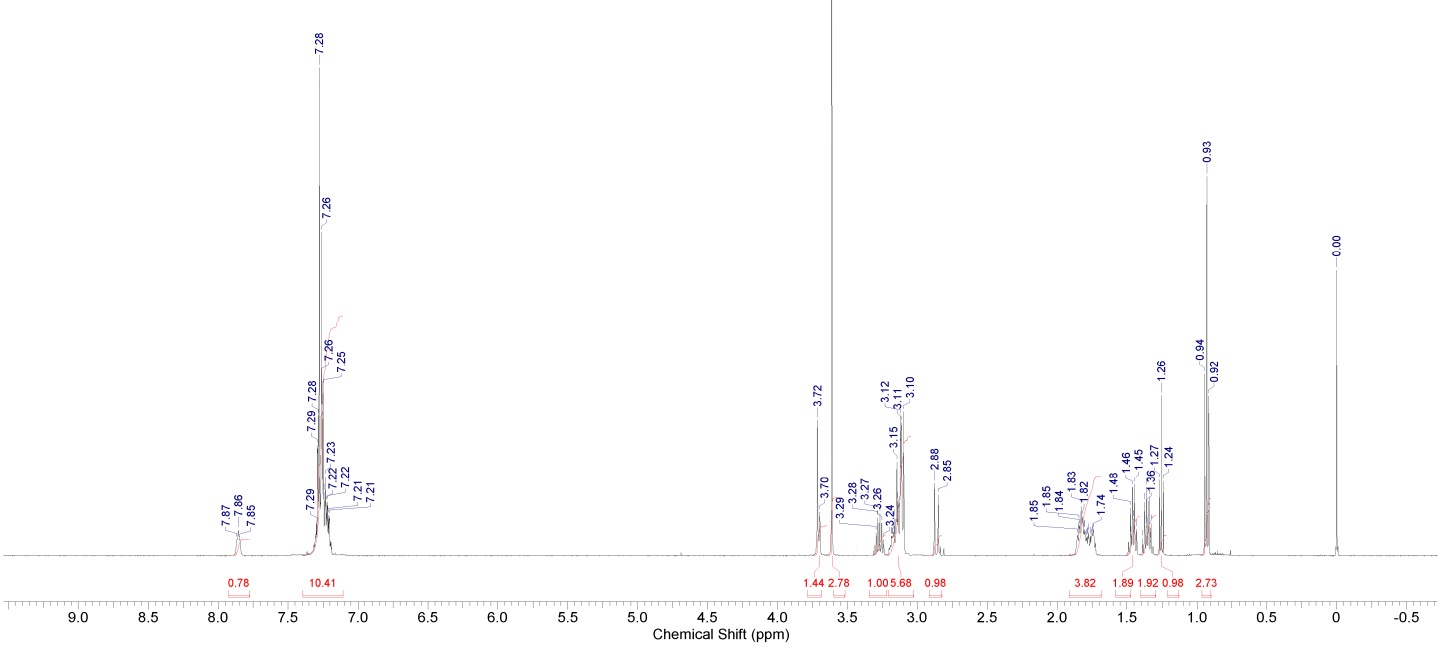

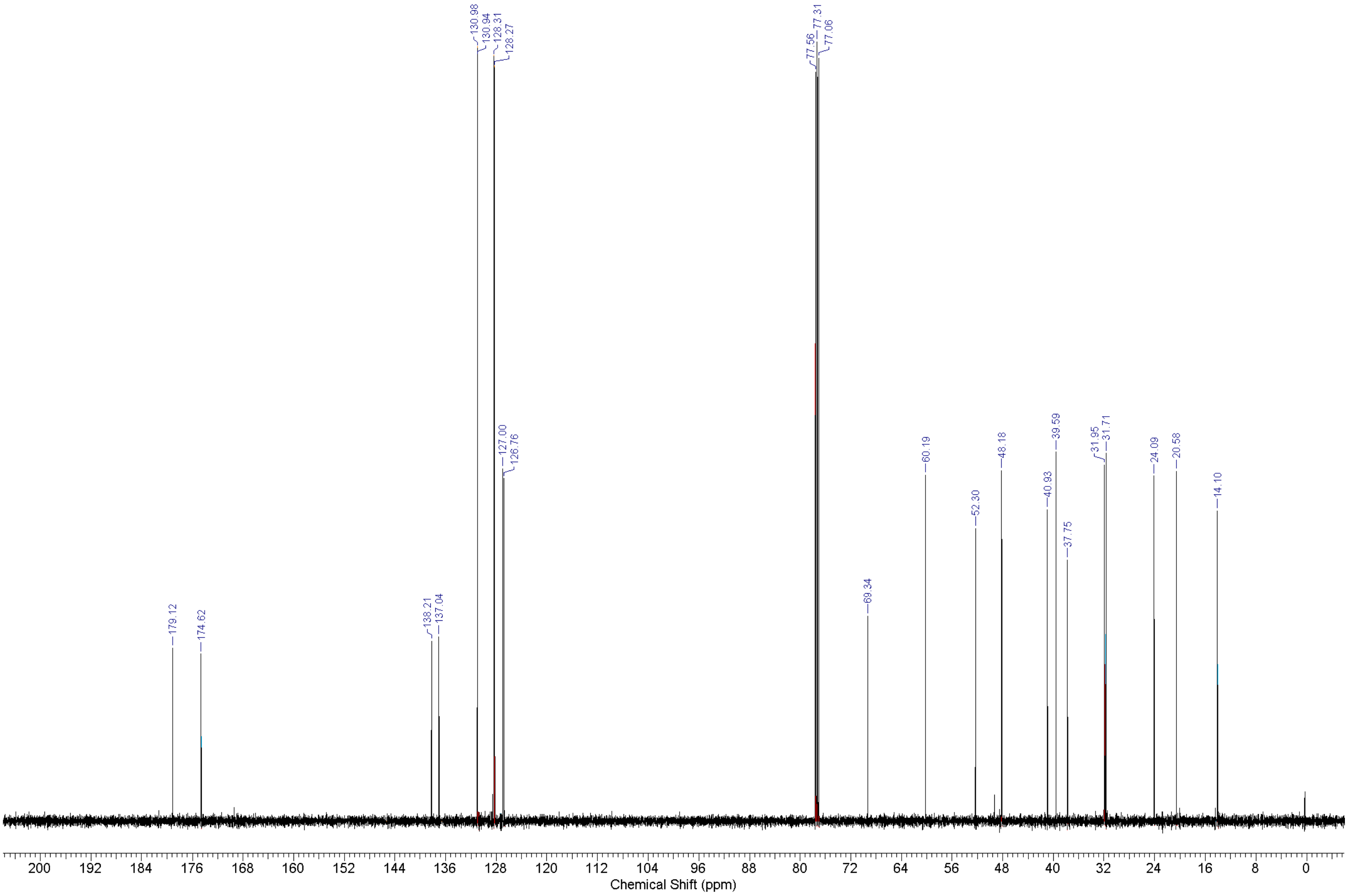

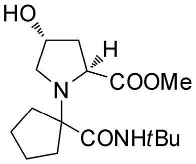
HO
H
COOMe
N
CONHtBu

1m

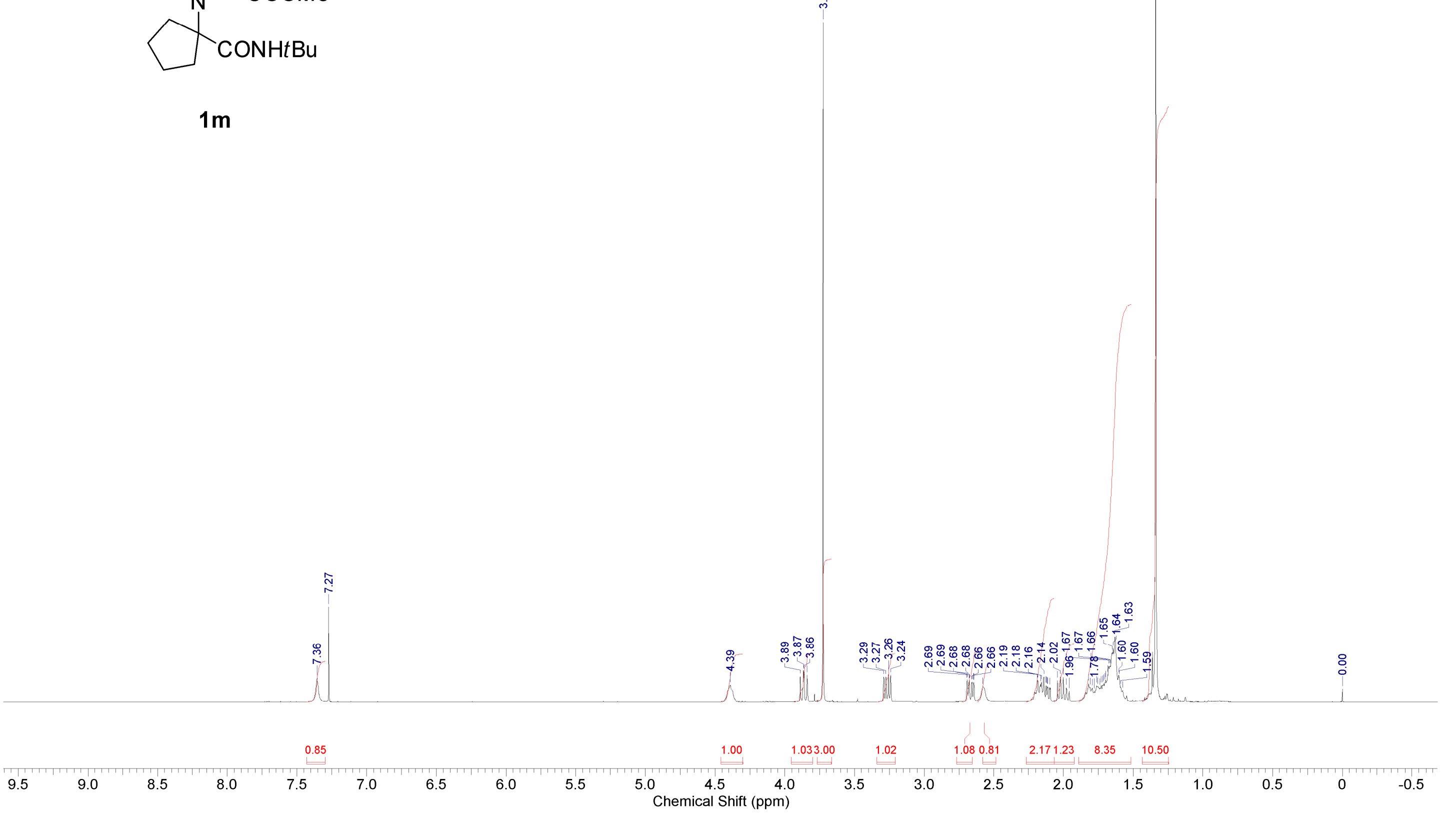
7.36
7.27
4.39
3.89
3.87
3.86
3.73
3.29
3.27
3.26
3.24
1.34
0.00
0.85
1.00
1.03
3.00
1.02
1.08
0.81
2.17
1.23
8.35
10.50
Chemical Shift (ppm)

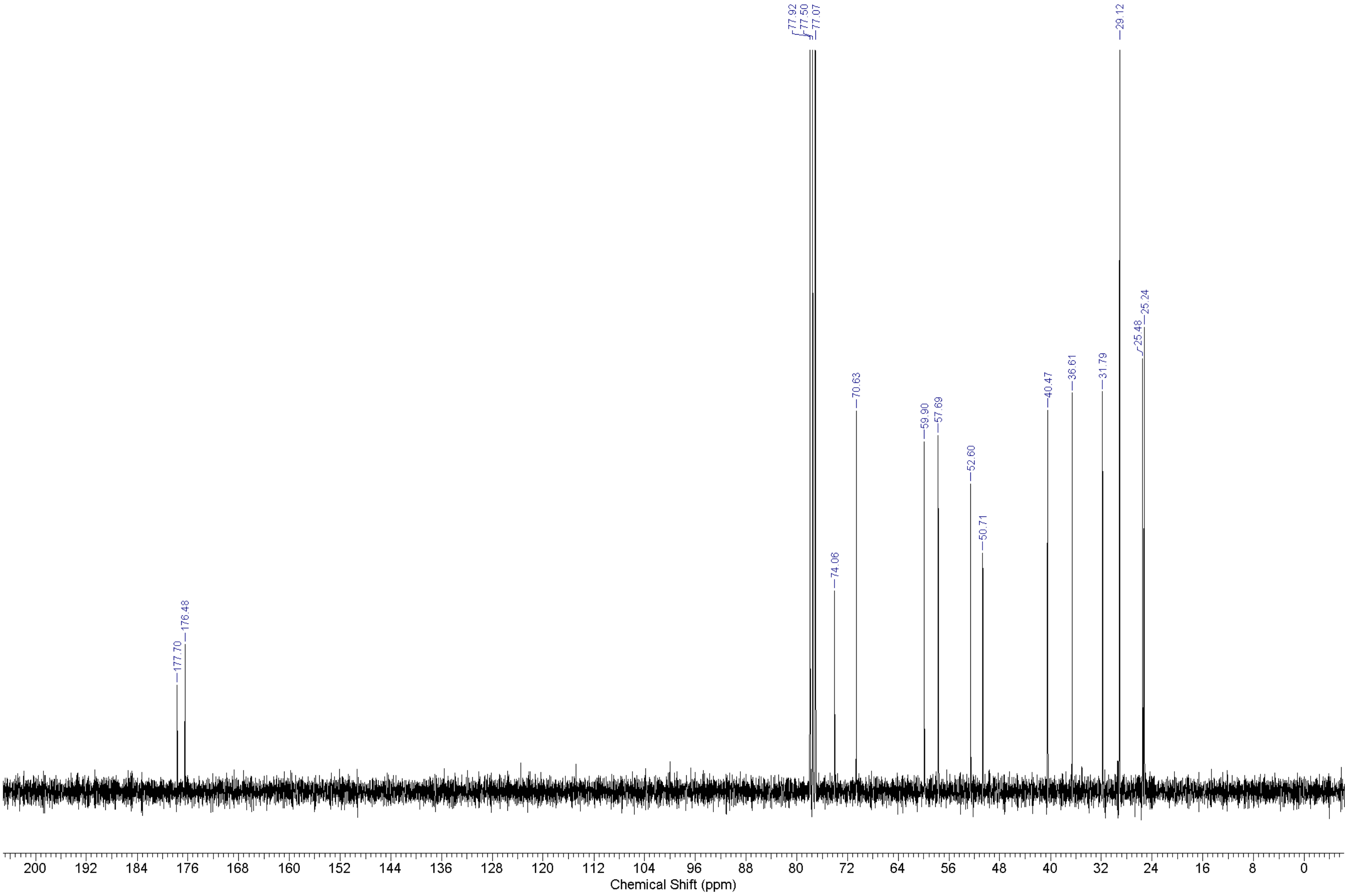
177.70
176.48
77.92
77.50
77.07
74.06
70.63
59.90
57.69
52.60
50.71
40.47
36.61
31.79
29.12
25.48
25.24
Chemical Shift (ppm)

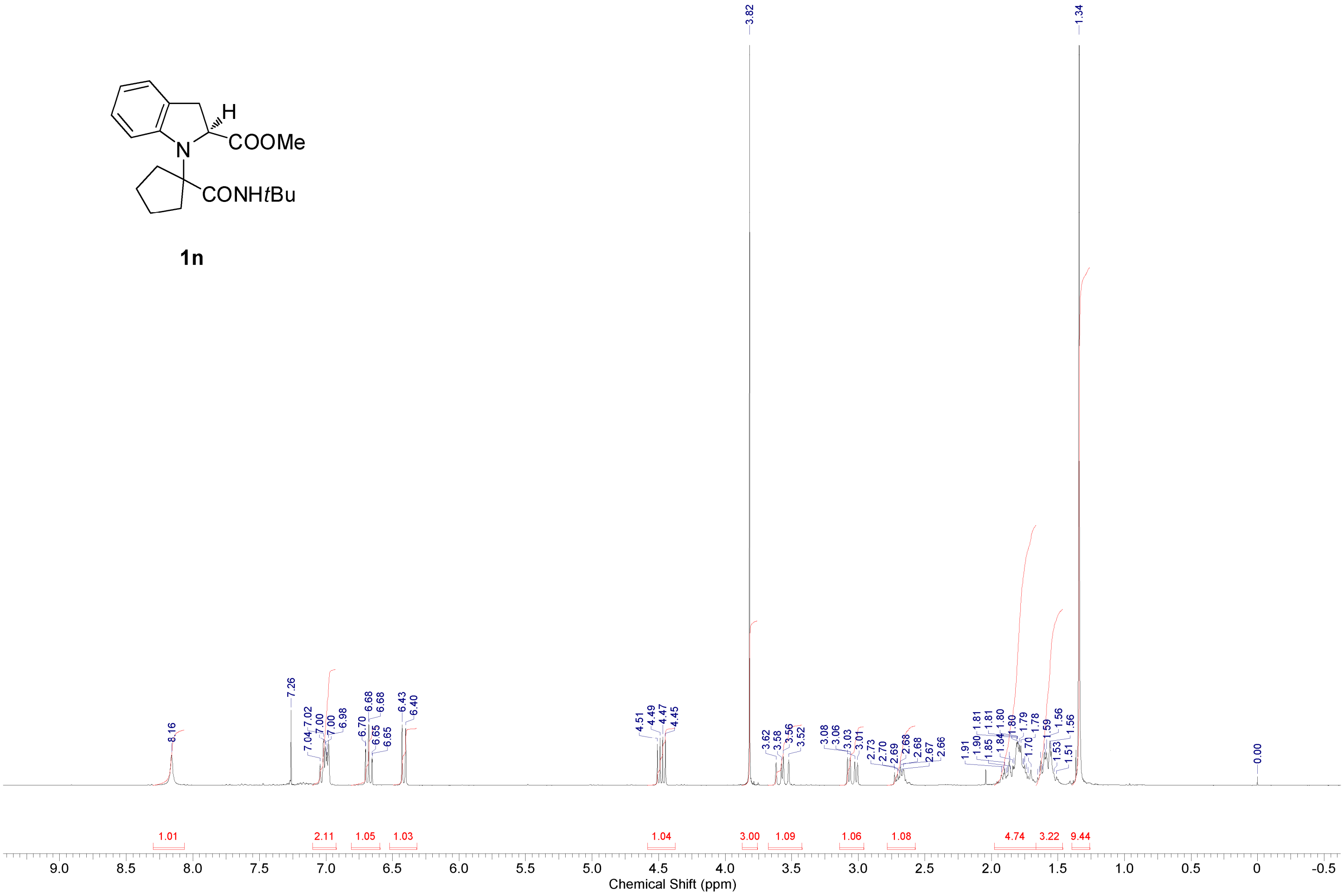
H
COOMe
N
CONHtBu
1n
3.82
1.34
8.16
7.26
7.04 7.02 7.00 7.00 6.98
6.70 6.68 6.68 6.65 6.65
6.43 6.40
4.51 4.49 4.47 4.45
3.62 3.58 3.56 3.52
3.08 3.06 3.03 3.01
2.73 2.70 2.69 2.68 2.68 2.67 2.66
1.91 1.90 1.85 1.84 1.81 1.81 1.80 1.80 1.79 1.78 1.70 1.59 1.56 1.56 1.53 1.51
0.00
1.01
2.11
1.05
1.03
1.04
3.00
1.09
1.06
1.08
4.74
3.22
9.44
9.0
8.5
8.0
7.5
7.0
6.5
6.0
5.5
5.0
4.5
4.0
3.5
3.0
2.5
2.0
1.5
1.0
0.5
0
-0.5
Chemical Shift (ppm)

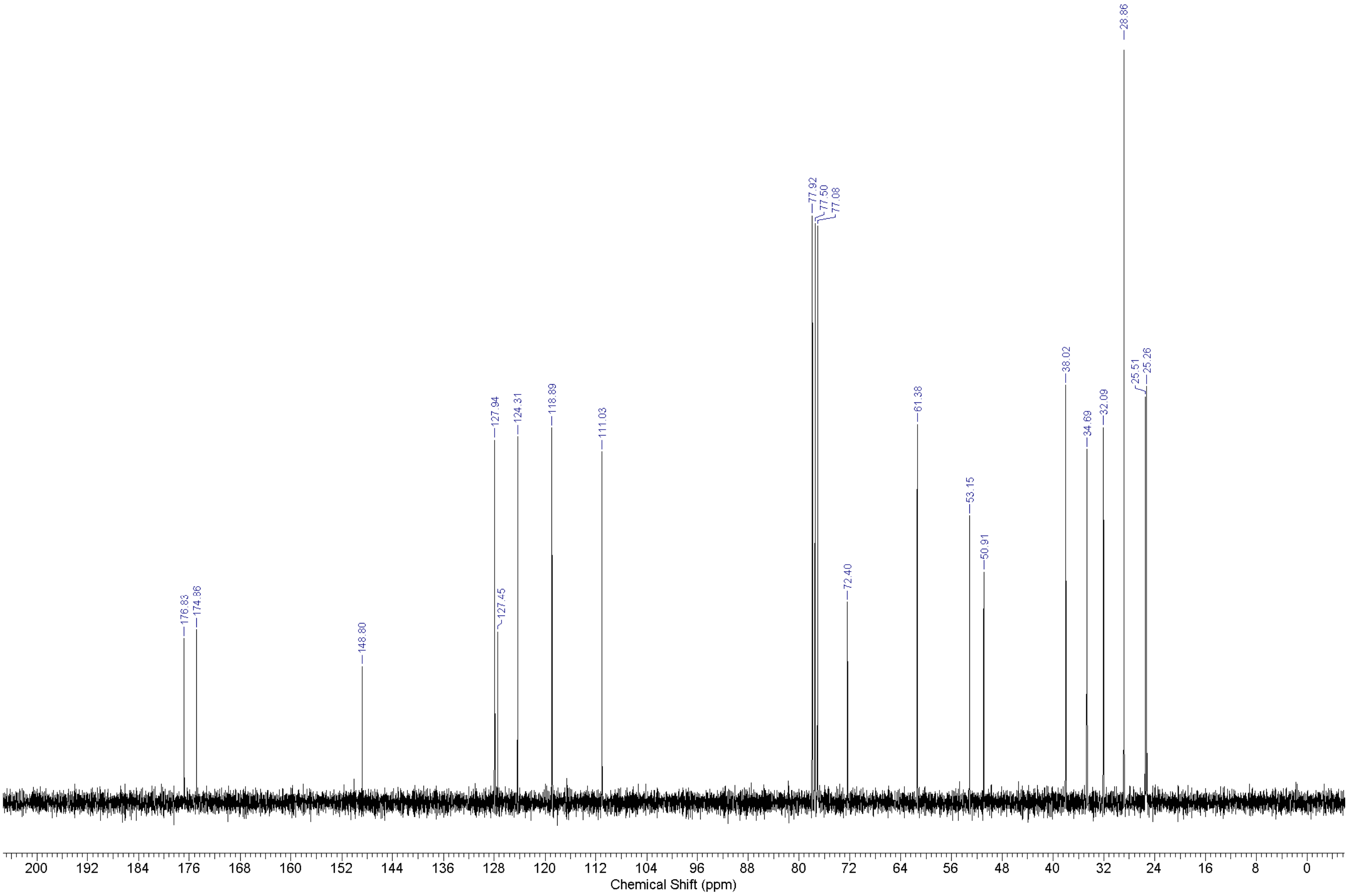
28.86
77.92 77.50 77.08
38.02
25.51 25.26
127.94
124.31
118.89
111.03
61.38
34.69
32.09
53.15
50.91
72.40
176.83 174.86
127.45
148.80
200
192
184
176
168
160
152
144
136
128
120
112
104
96
88
80
72
64
56
48
40
32
24
16
8
0
Chemical Shift (ppm)

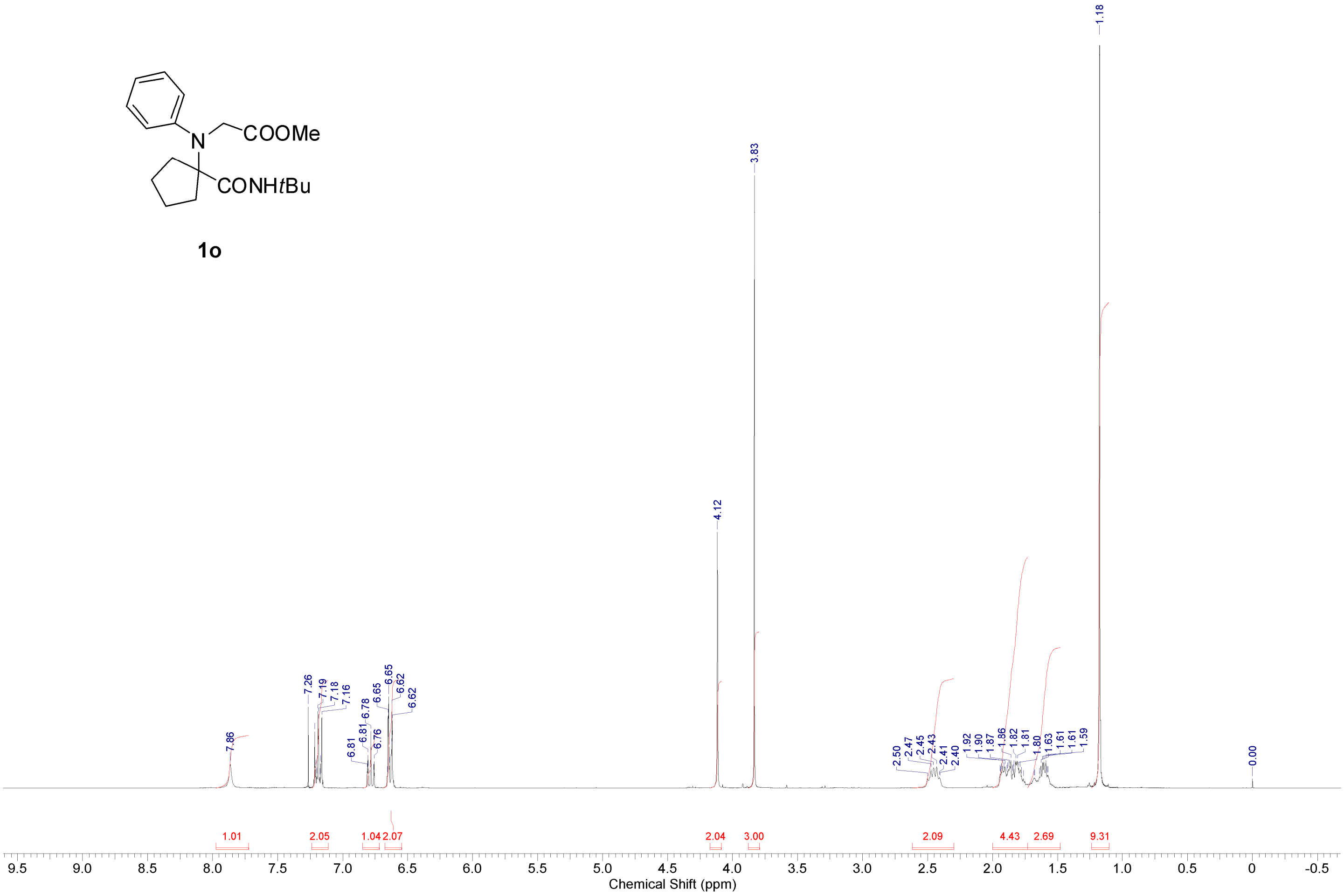
1o
Chemical Shift (ppm)

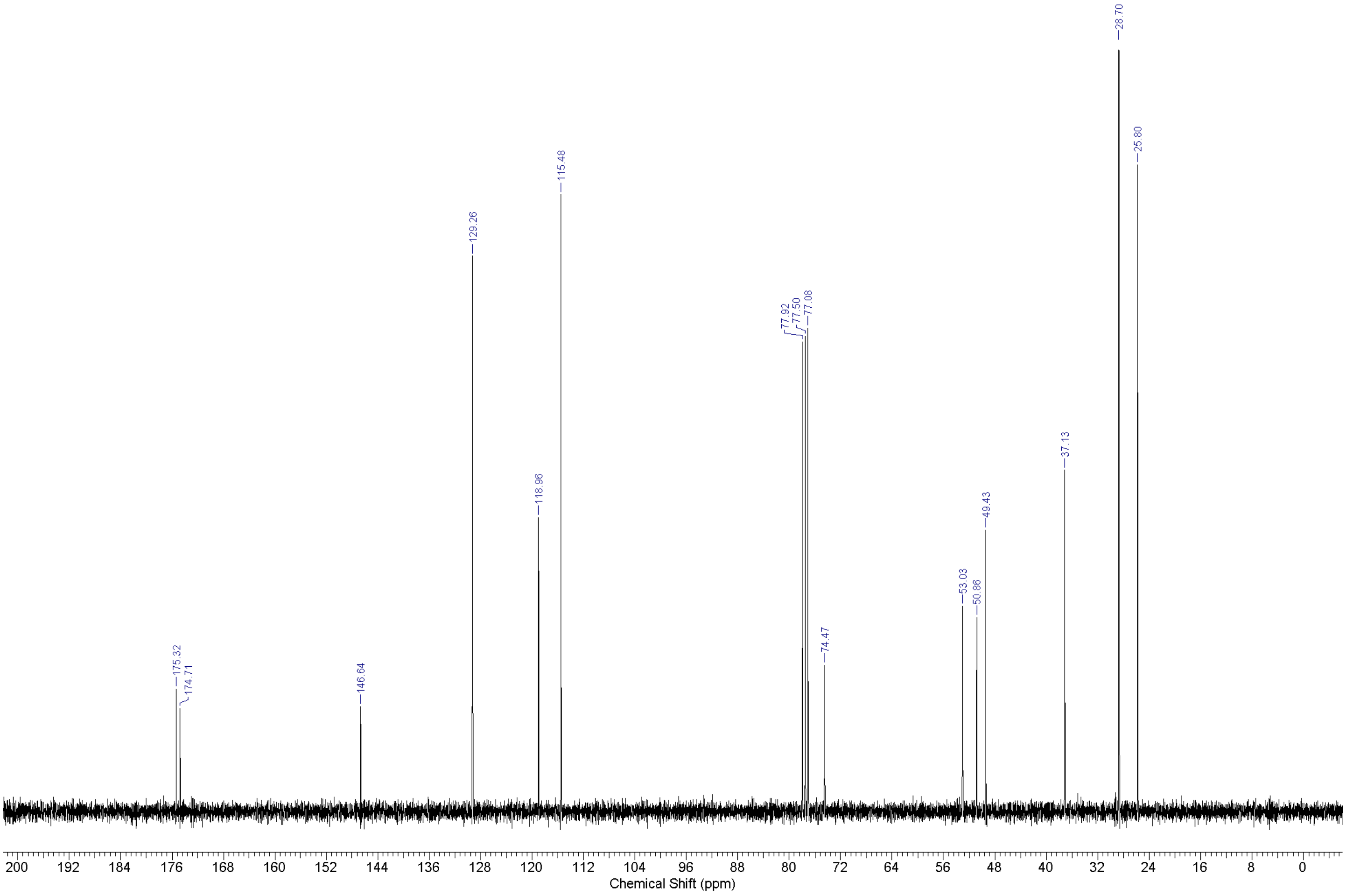
Chemical Shift (ppm)

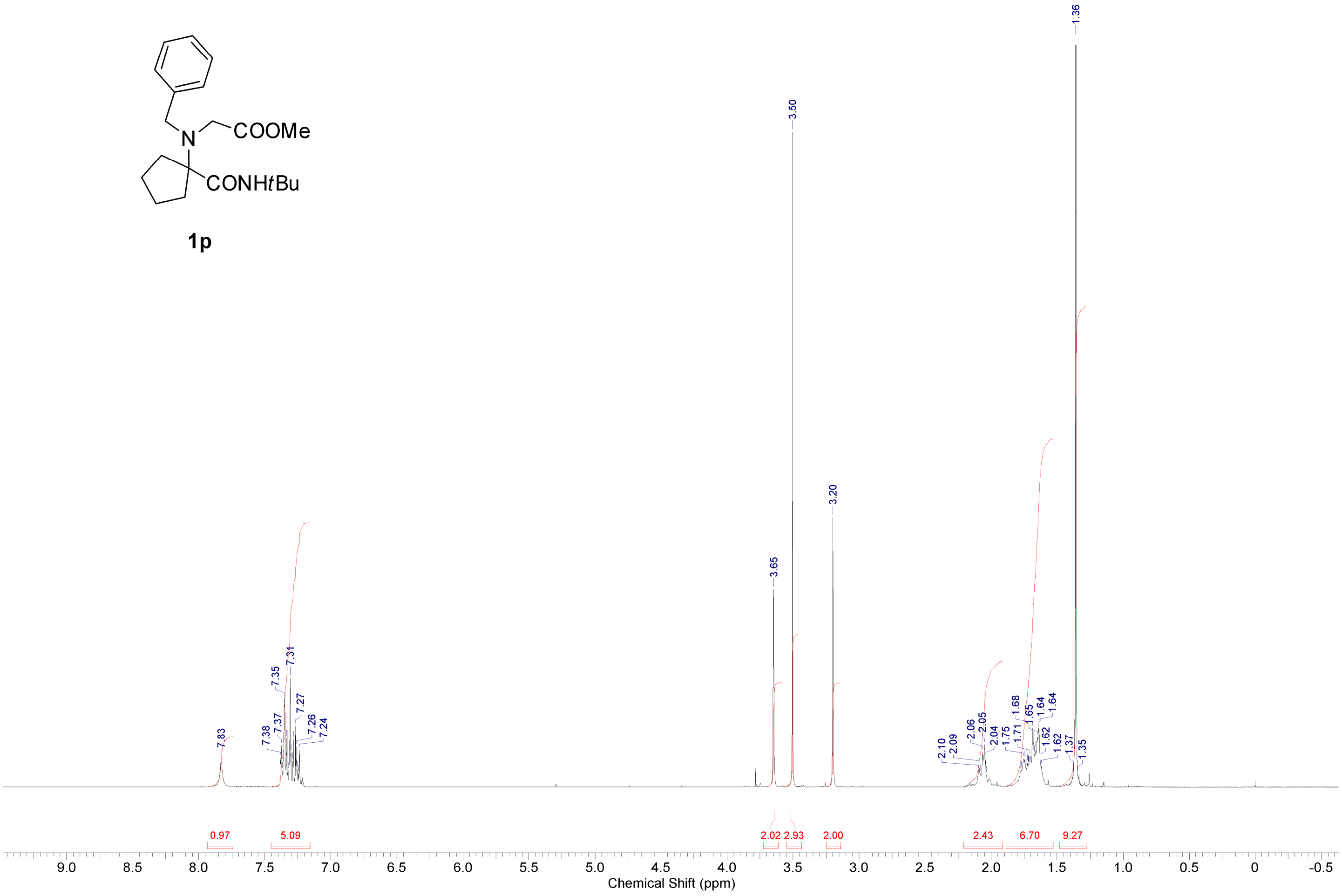
N
COOMe
CONHtBu
1p
7.83
7.38
7.37
7.35
7.31
7.27
7.26
7.24
3.65
3.50
3.20
2.10
2.09
2.06
2.05
2.04
1.75
1.71
1.68
1.65
1.64
1.64
1.62
1.37
1.36
1.35
0.97
5.09
2.02
2.93
2.00
2.43
6.70
9.27
9.0 8.5 8.0 7.5 7.0 6.5 6.0 5.5 5.0 4.5 4.0 3.5 3.0 2.5 2.0 1.5 1.0 0.5 0 -0.5
Chemical Shift (ppm)

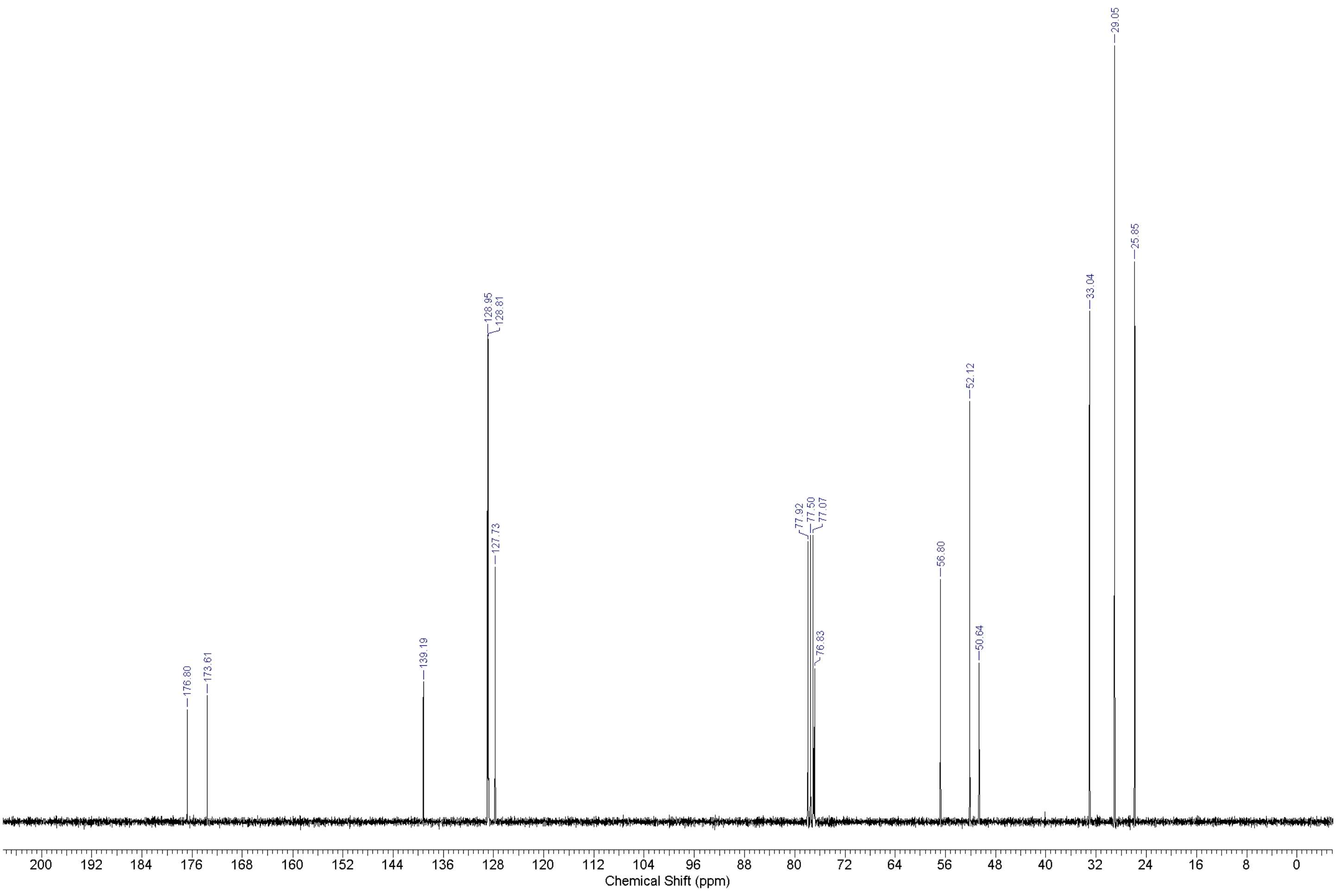
176.80
173.61
139.19
128.95
128.81
127.73
77.92
77.50
77.07
76.83
56.80
52.12
50.64
33.04
29.05
25.85
200 192 184 176 168 160 152 144 136 128 120 112 104 96 88 80 72 64 56 48 40 32 24 16 8 0
Chemical Shift (ppm)

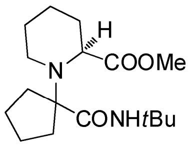
H
COOMe
N
CONHtBu

1q

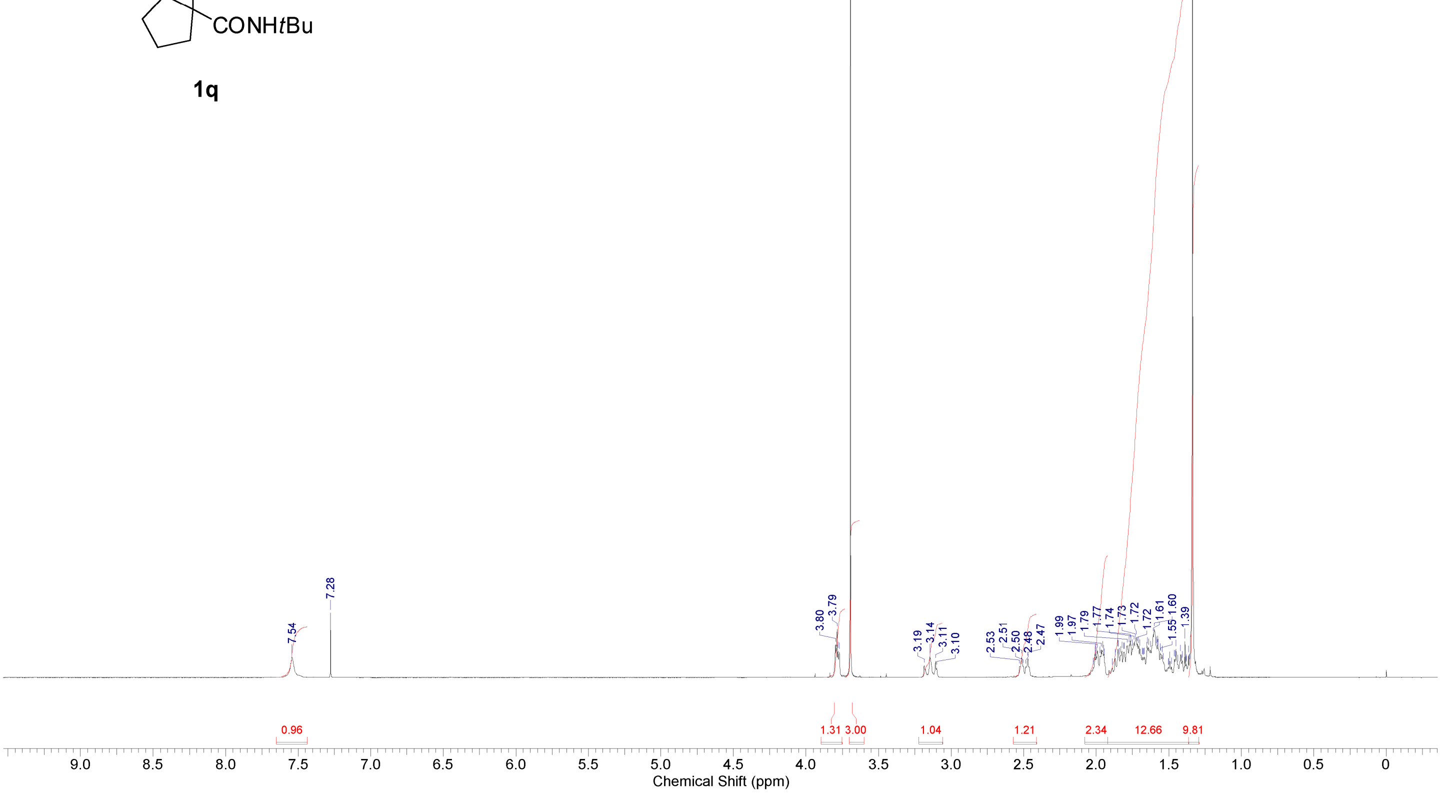
7.54
7.28
3.80
3.79
3.69
3.19
3.14
3.11
3.10
2.53
2.51
2.50
2.48
2.47
1.99
1.97
1.79
1.77
1.74
1.73
1.72
1.72
1.61
1.60
1.55
1.39
1.34
0.96
1.31
3.00
1.04
1.21
2.34
12.66
9.81
Chemical Shift (ppm)

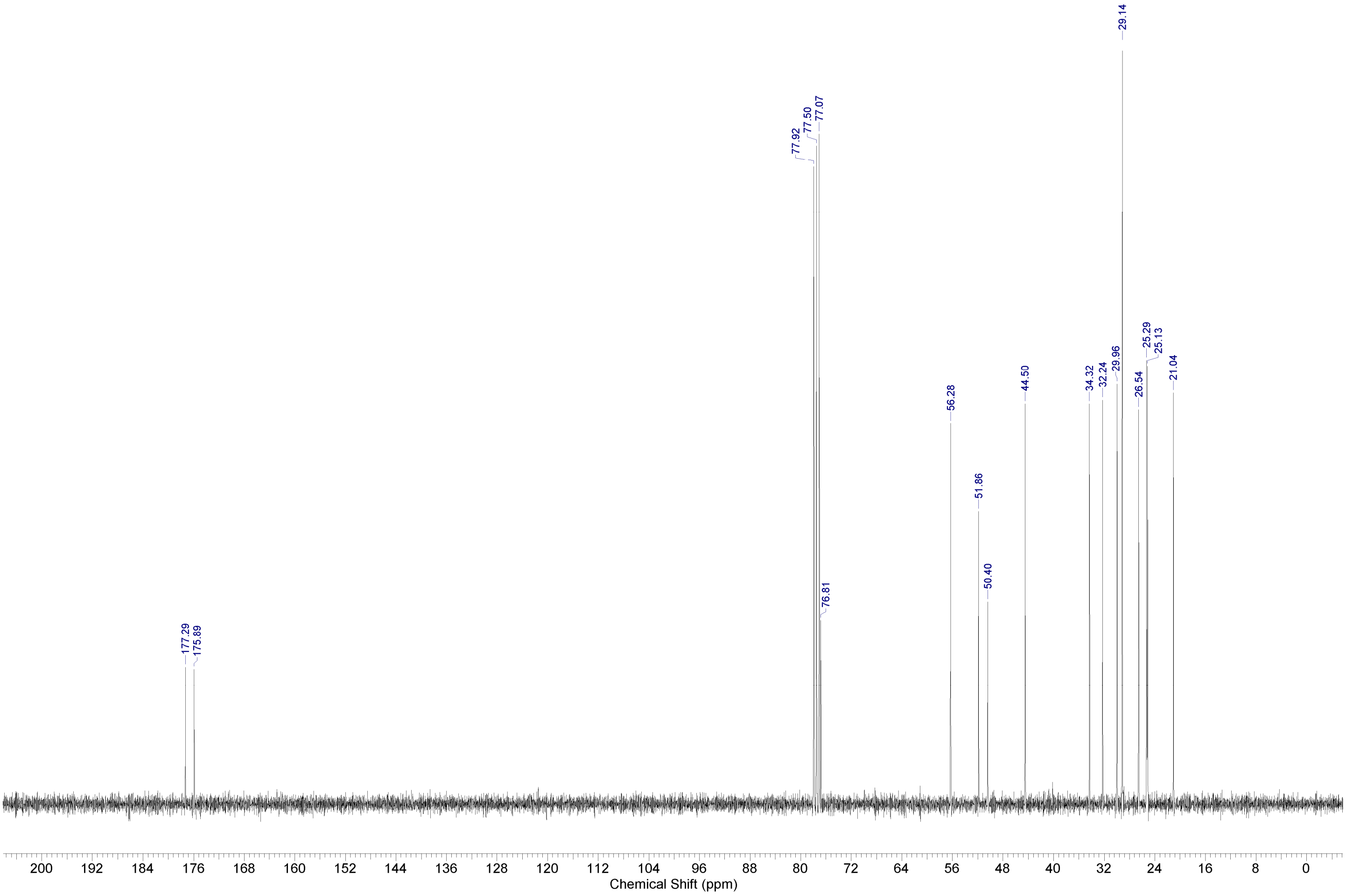
177.29
175.89
77.92
77.50
77.07
76.81
56.28
51.86
50.40
44.50
34.32
32.24
29.96
29.14
26.54
25.29
25.13
21.04
Chemical Shift (ppm)

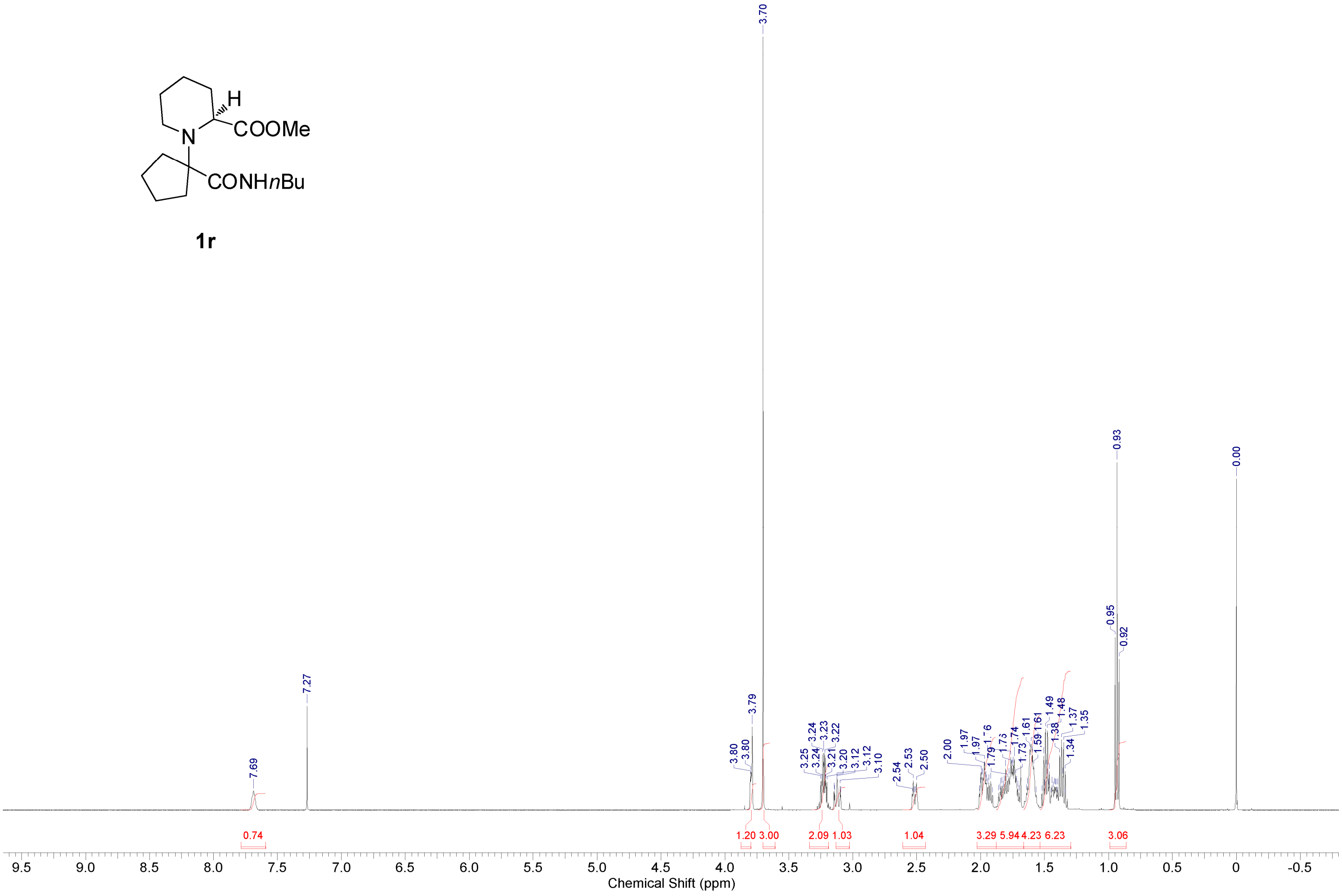
H
COOMe
N
CONHnBu
1r
3.70
7.27
7.69
0.93
0.95
0.92
0.00
0.74
1.20
3.00
2.09
1.03
1.04
3.29
5.94
4.23
6.23
3.06
Chemical Shift (ppm)

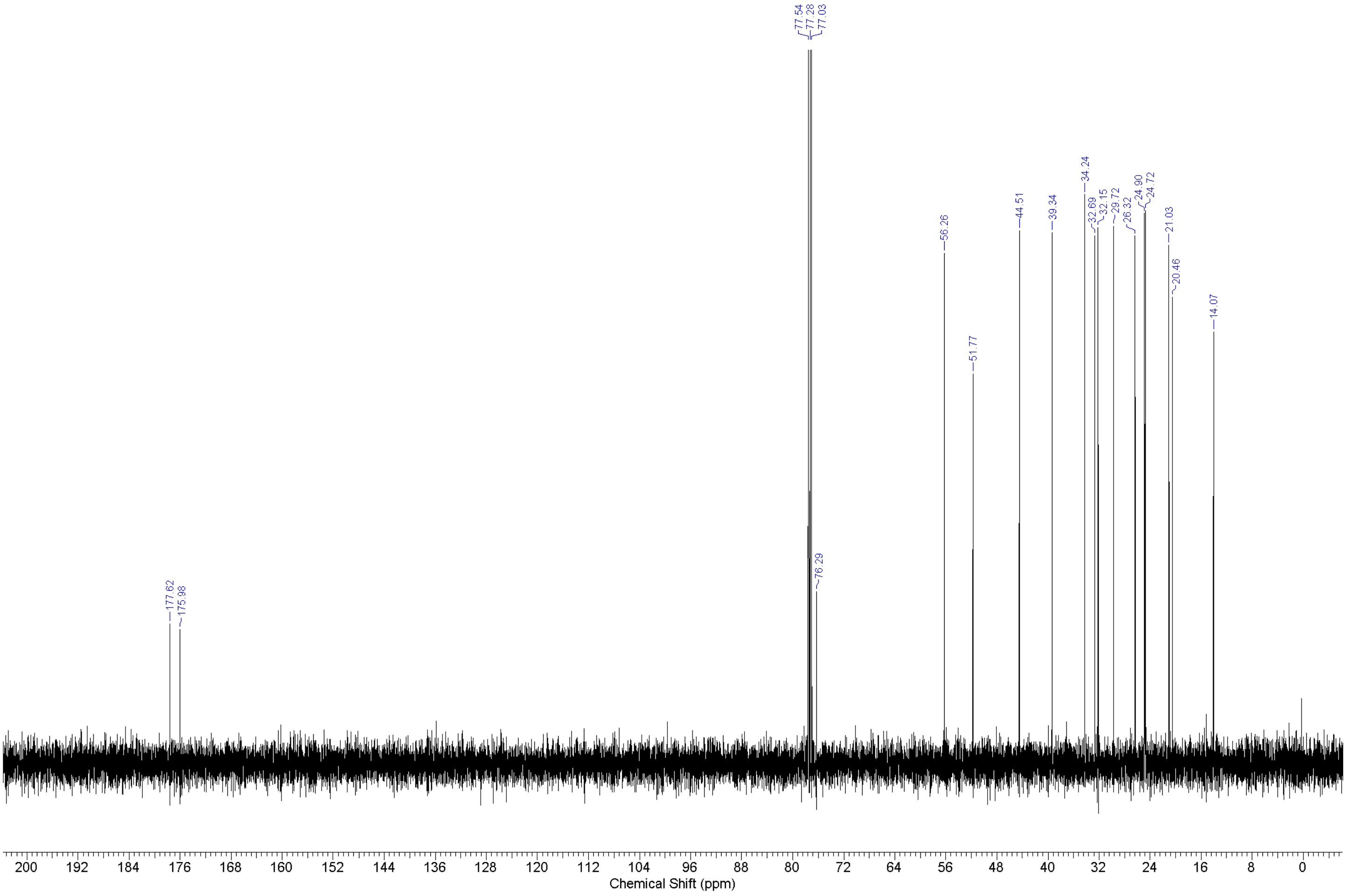
77.54
77.28
77.03
76.29
177.62
175.98
56.26
51.77
44.51
39.34
34.24
32.69
32.15
29.72
26.32
24.90
24.72
21.03
20.46
14.07
Chemical Shift (ppm)

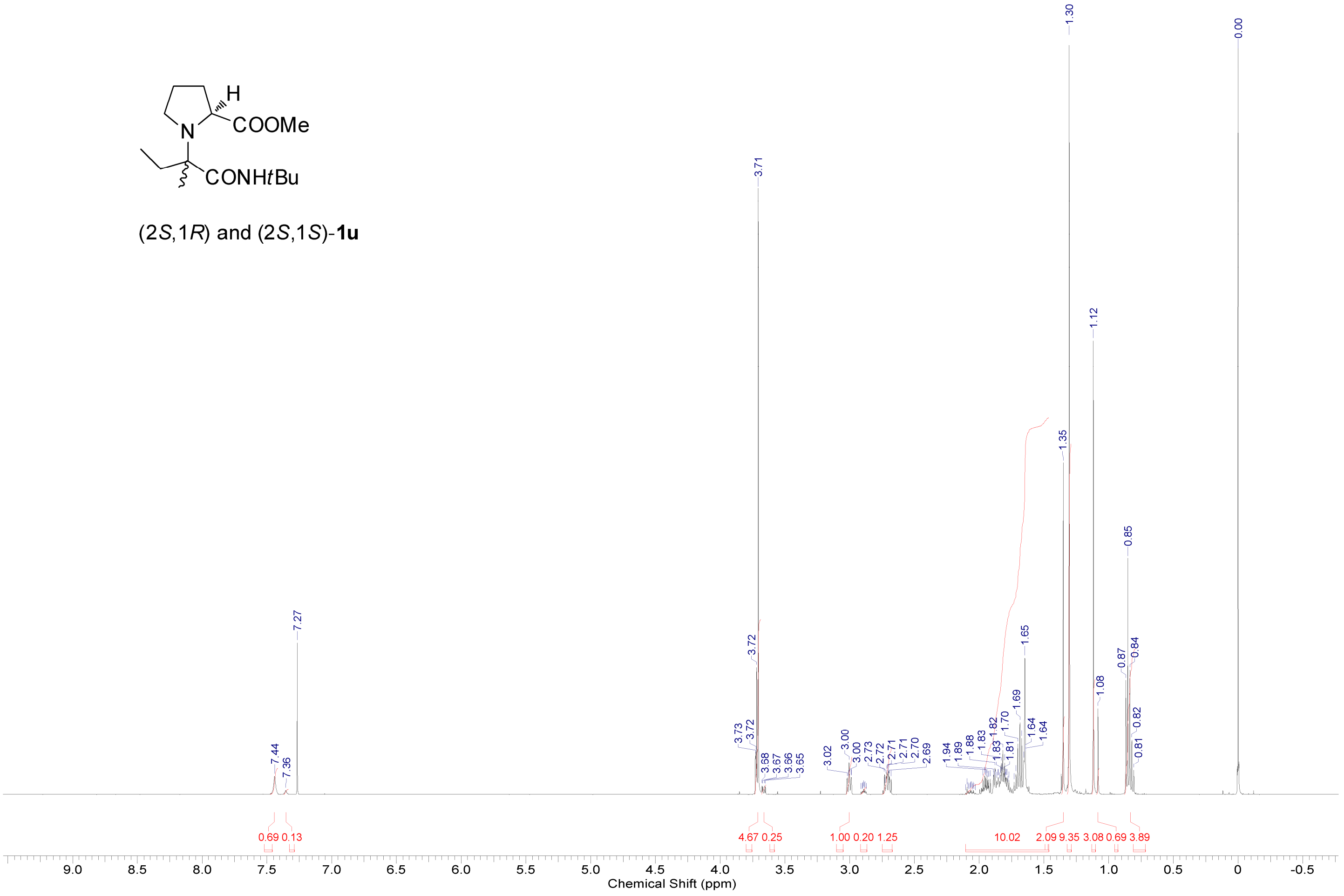

(2*S*,1*R*) and (2*S*,1*S*)-**1u**

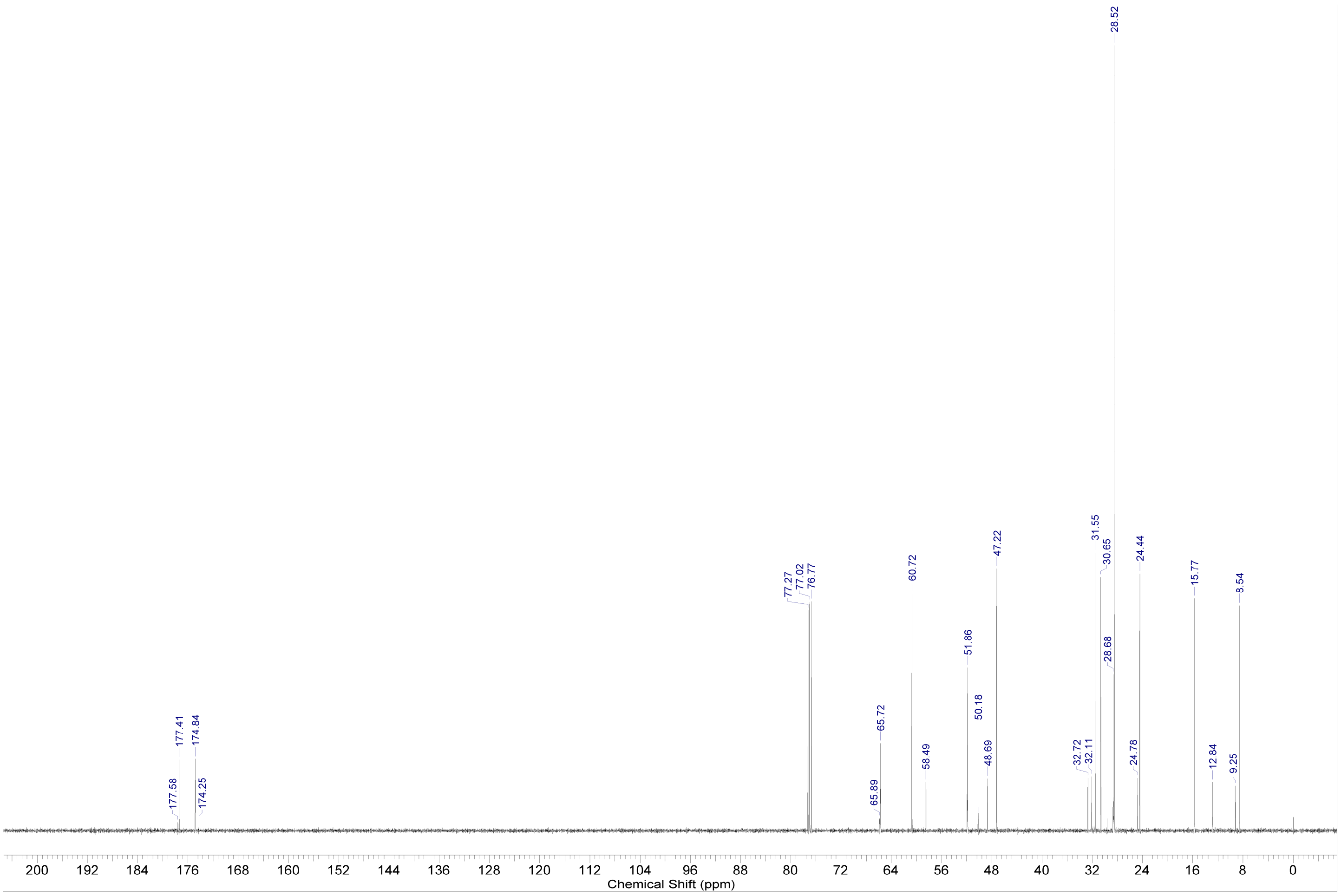

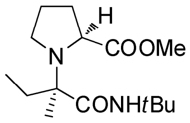
H
COOMe
N
CONHtBu

(2*S*,1*R*)-**1u**

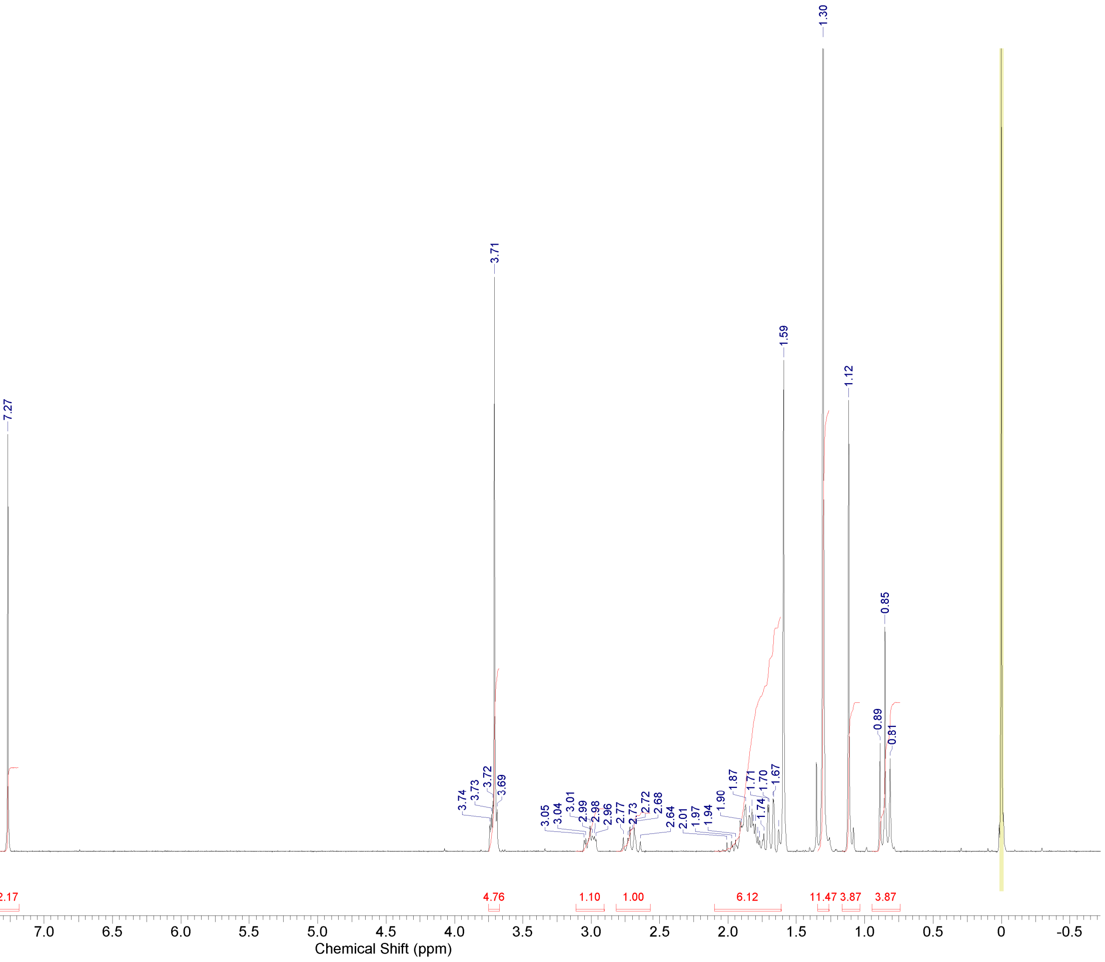
7.27
3.71
3.74
3.73
3.72
3.69
3.05
3.04
3.01
2.99
2.98
2.96
2.77
2.73
2.72
2.68
2.64
2.01
1.97
1.94
1.90
1.87
1.74
1.71
1.70
1.67
1.59
1.30
1.12
0.89
0.85
0.81
2.17
4.76
1.10
1.00
6.12
11.47
3.87
3.87
9.5 9.0 8.5 8.0 7.5 7.0 6.5 6.0 5.5 5.0 4.5 4.0 3.5 3.0 2.5 2.0 1.5 1.0 0.5 0 -0.5
Chemical Shift (ppm)

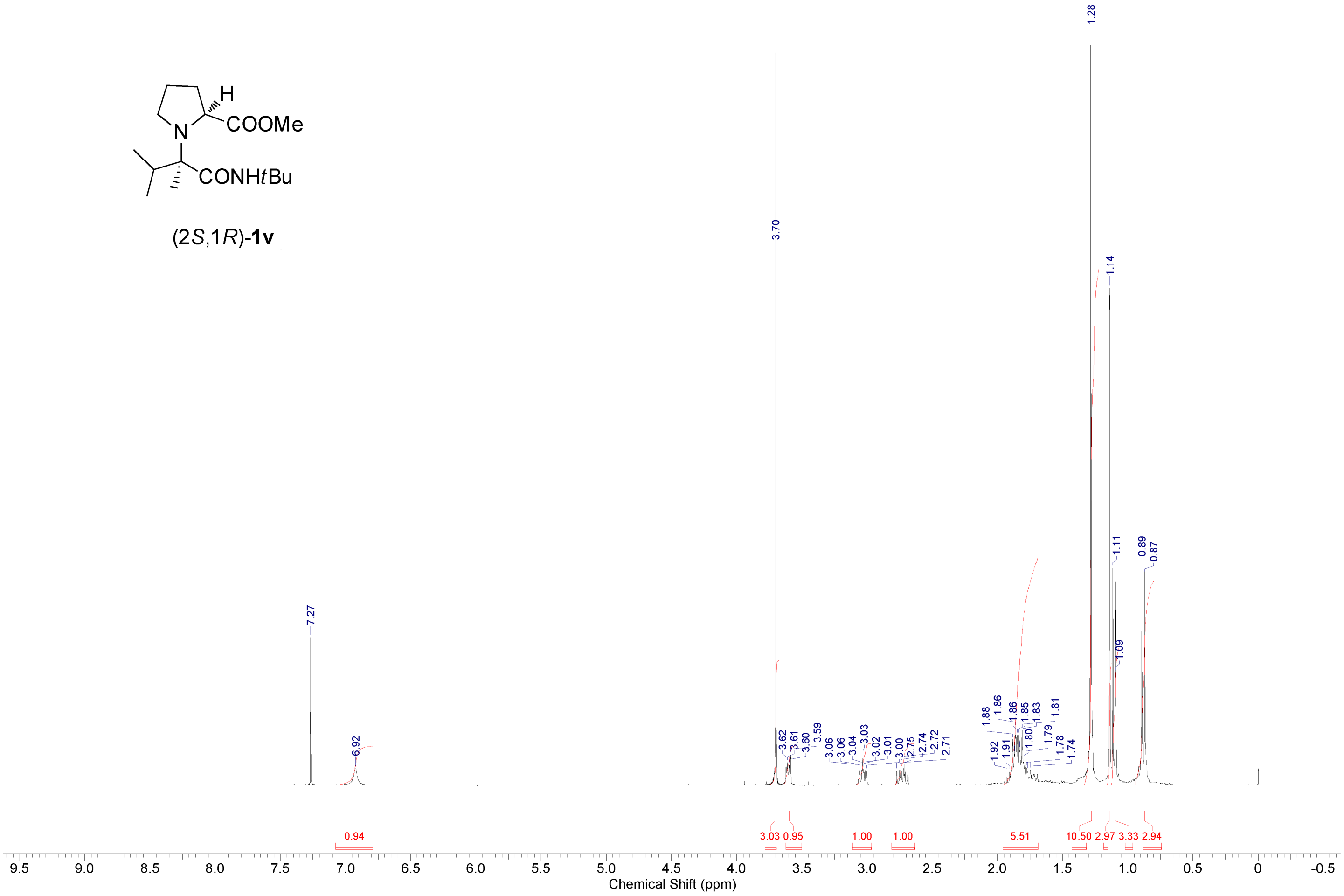

(2*S*,1*R*)-**1v**

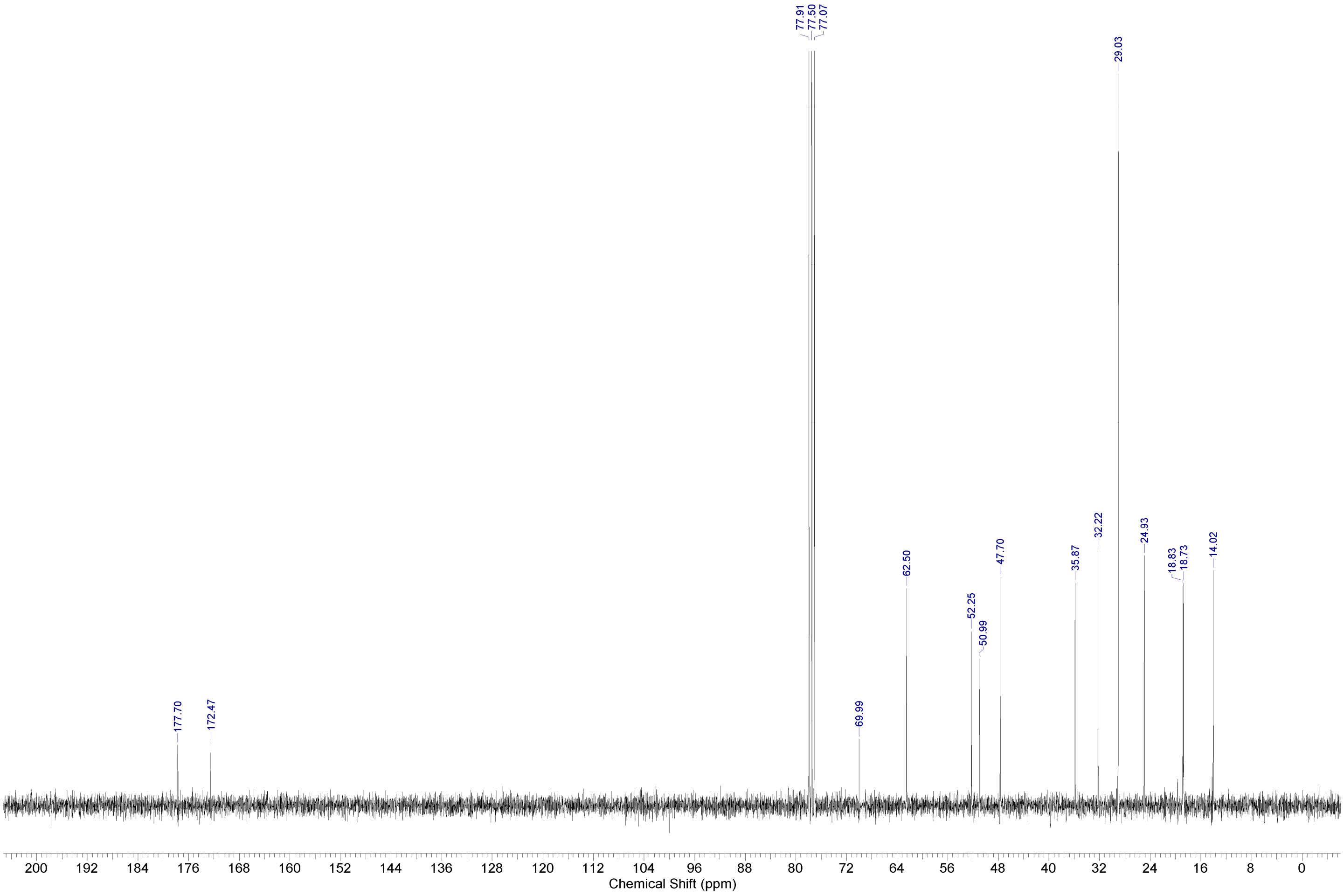

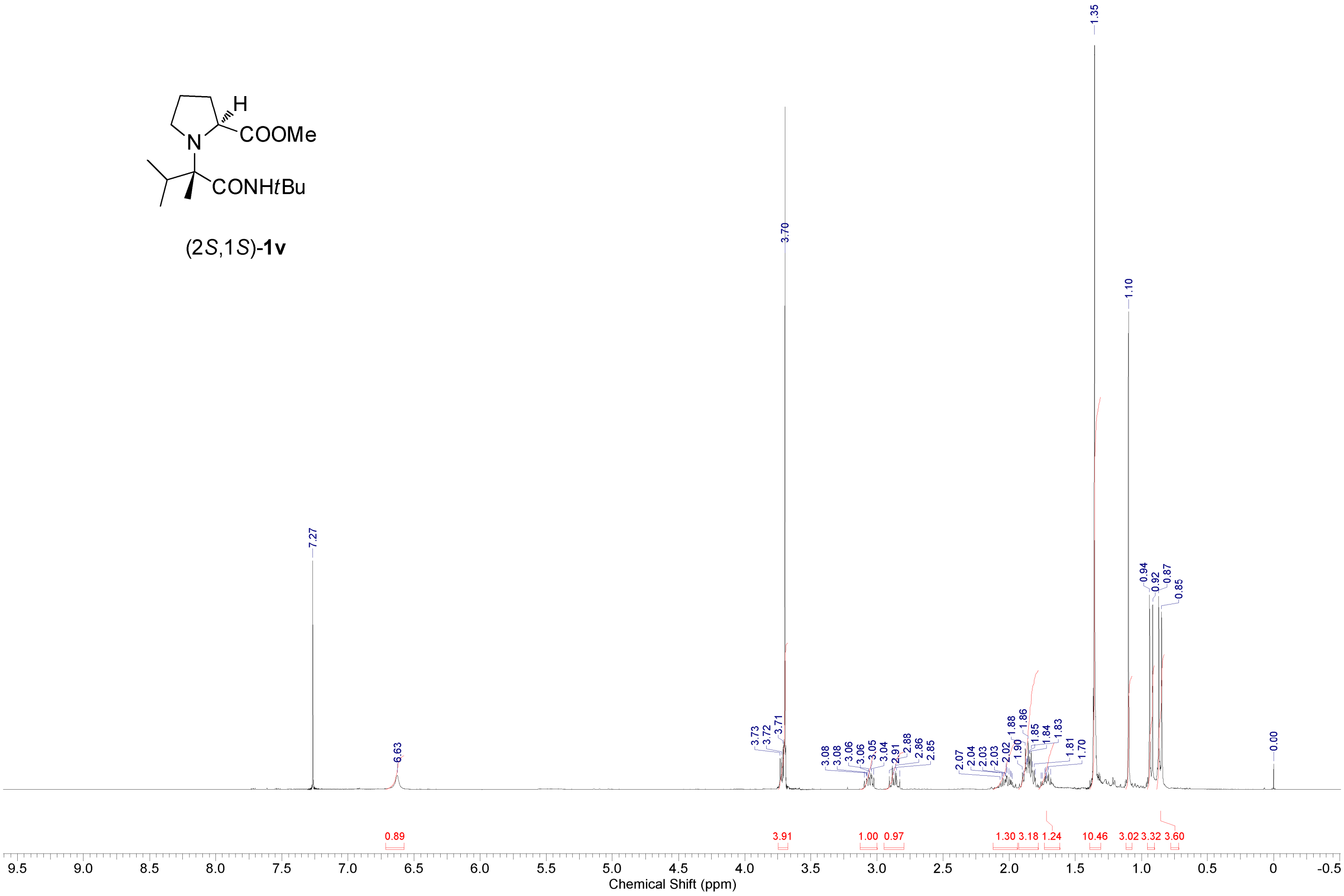
H
COOMe
N
CONHtBu
(2S,1S)-1v
7.27
6.63
3.70
3.73
3.72
3.71
3.08
3.08
3.06
3.06
3.05
3.04
2.91
2.88
2.86
2.85
2.07
2.04
2.03
2.03
2.02
1.90
1.88
1.86
1.85
1.84
1.83
1.81
1.70
1.35
1.10
0.94
0.92
0.87
0.85
0.00
0.89
3.91
1.00
0.97
1.30
3.18
1.24
10.46
3.02
3.32
3.60
9.5
9.0
8.5
8.0
7.5
7.0
6.5
6.0
5.5
5.0
4.5
4.0
3.5
3.0
2.5
2.0
1.5
1.0
0.5
0
-0.5
Chemical Shift (ppm)

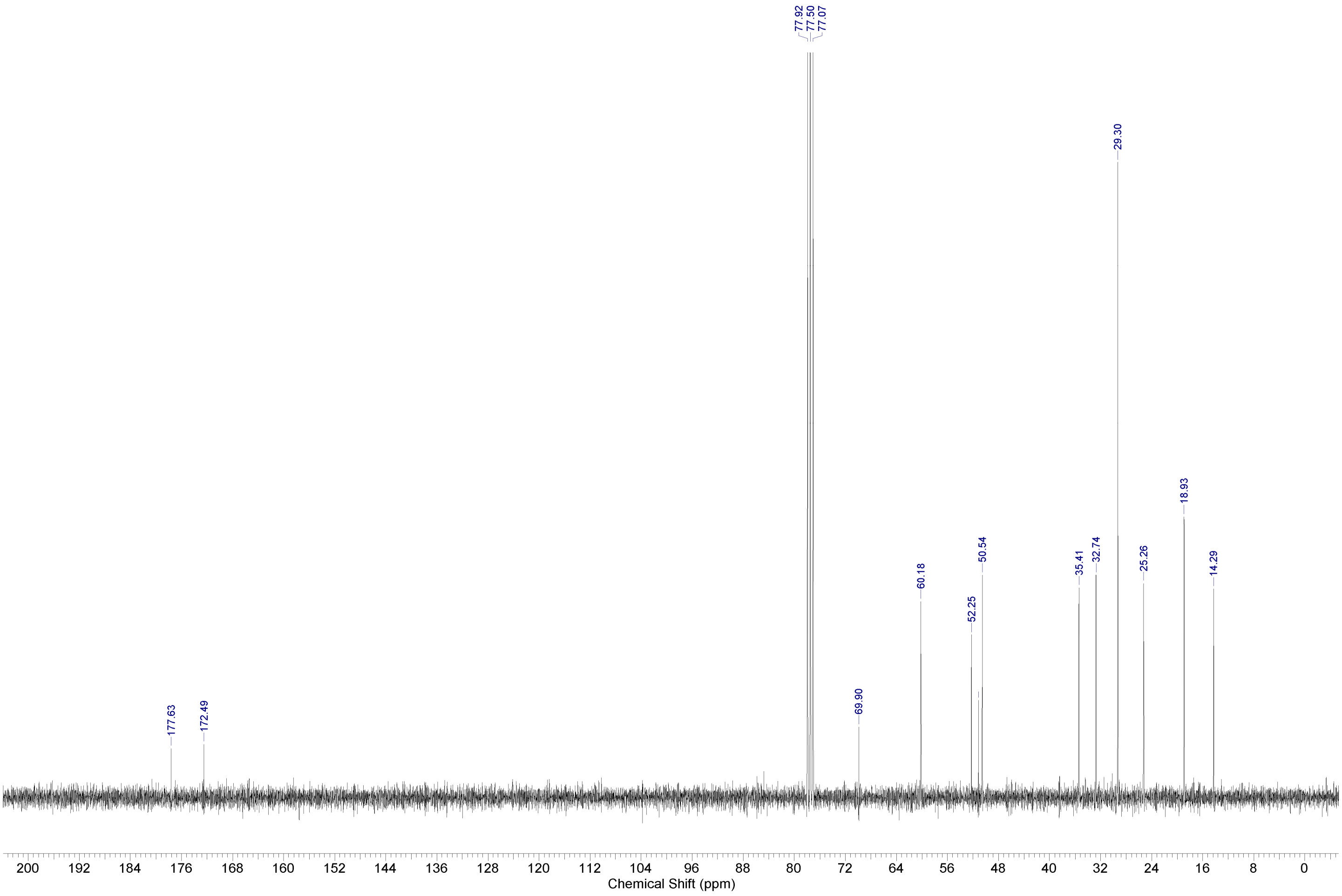
77.92
77.50
77.07
29.30
18.93
50.54
60.18
35.41
32.74
25.26
14.29
52.25
69.90
177.63
172.49
200
192
184
176
168
160
152
144
136
128
120
112
104
96
88
80
72
64
56
48
40
32
24
16
8
0
Chemical Shift (ppm)

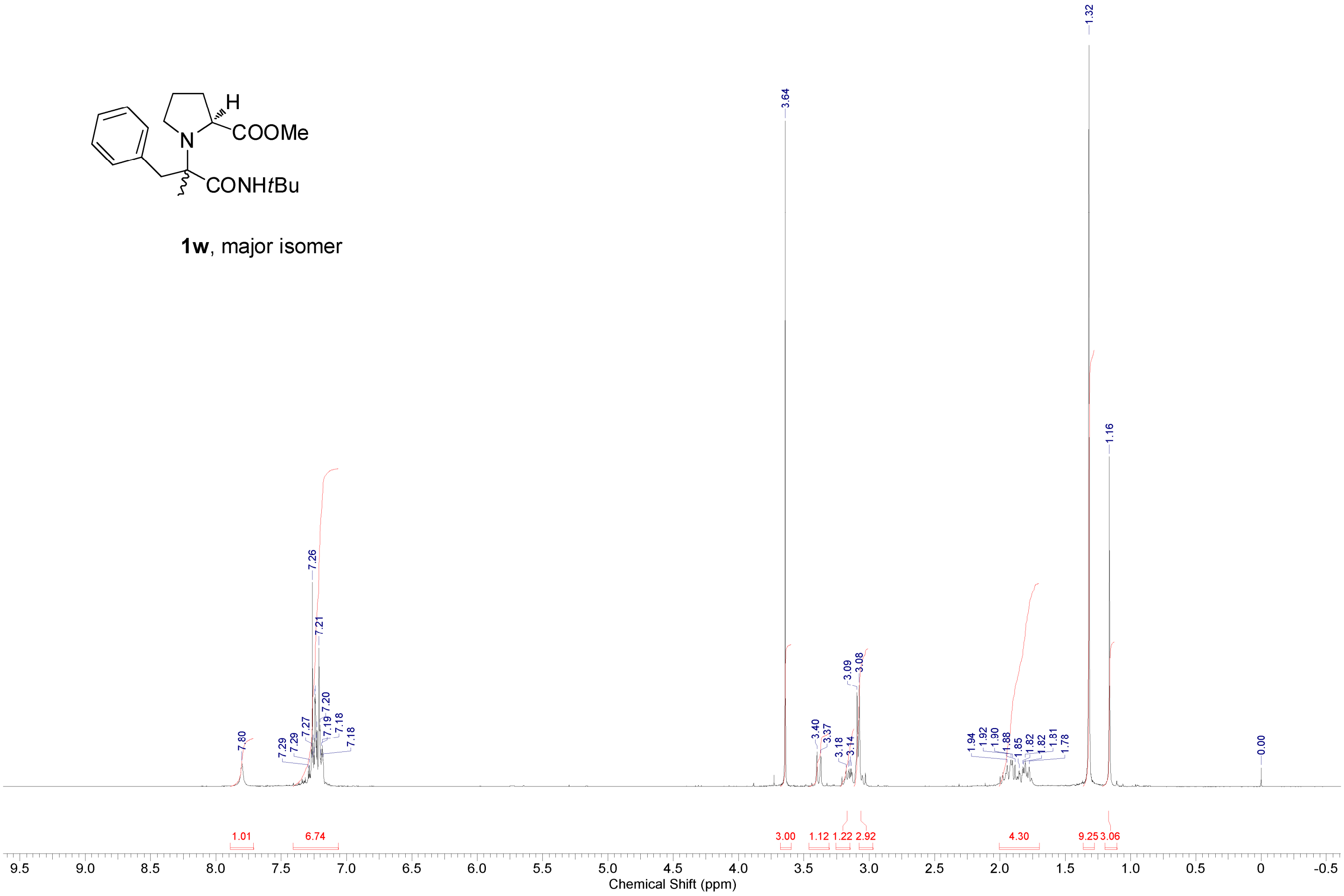

1w, major isomer

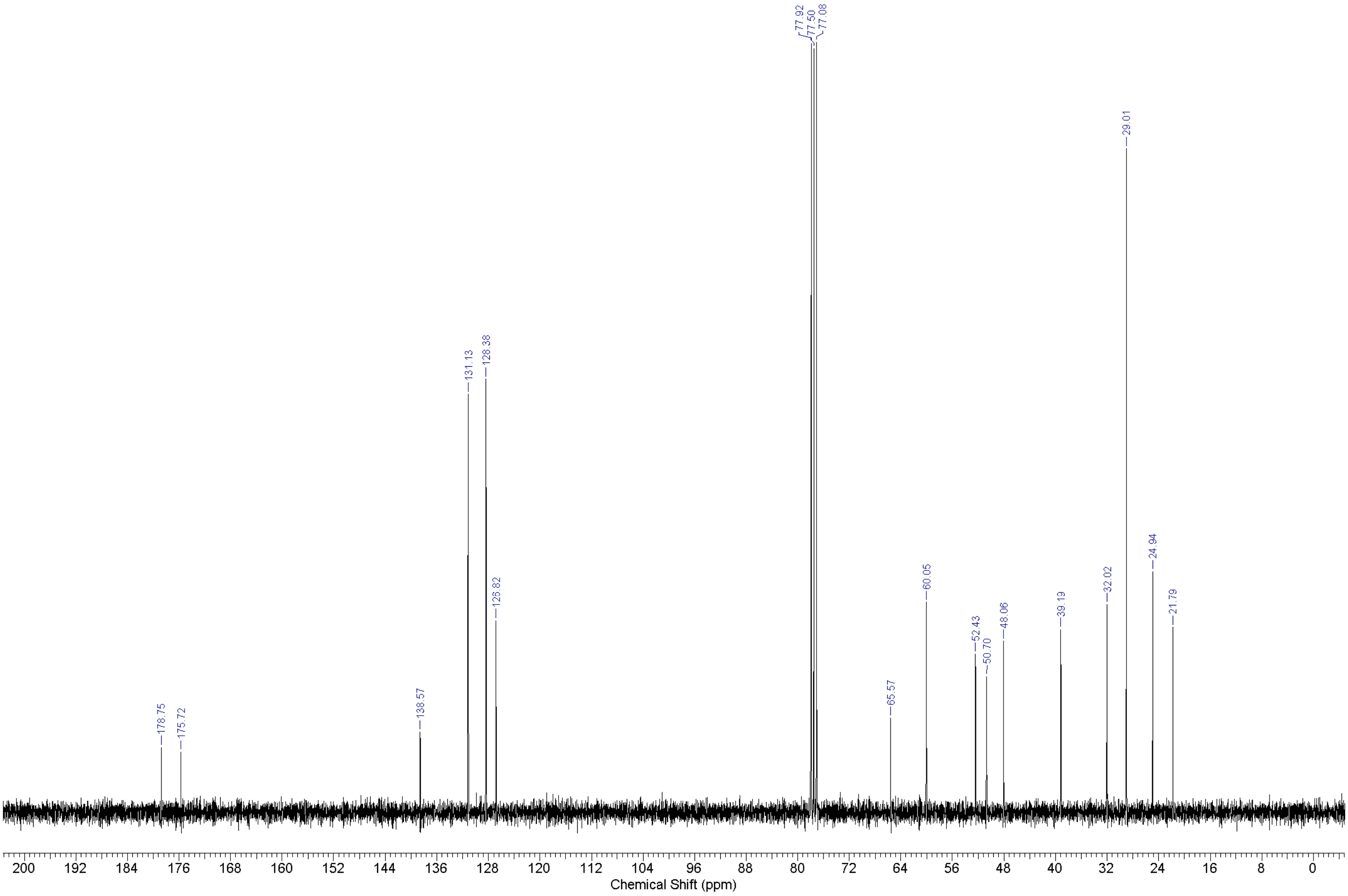

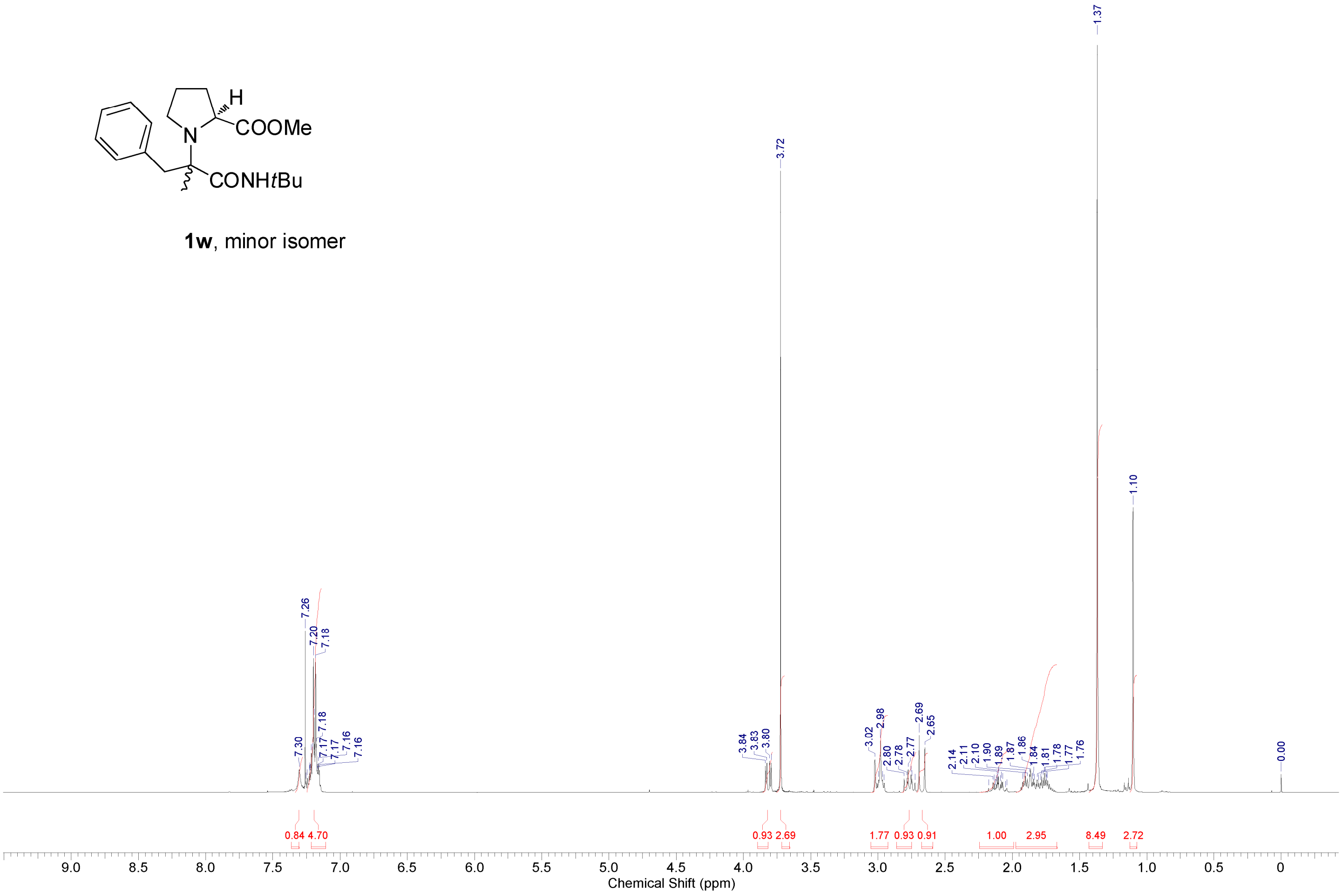
H
COOMe
N
CONHtBu
1w, minor isomer
7.30
7.26
7.20
7.18
7.18
7.17
7.17
7.16
7.16
3.84
3.83
3.80
3.72
3.02
2.98
2.80
2.78
2.77
2.69
2.65
2.14
2.11
2.10
1.90
1.89
1.87
1.86
1.84
1.81
1.78
1.77
1.76
1.37
1.10
0.00
0.84 4.70
0.93 2.69
1.77 0.93 0.91
1.00 2.95
8.49 2.72
Chemical Shift (ppm)

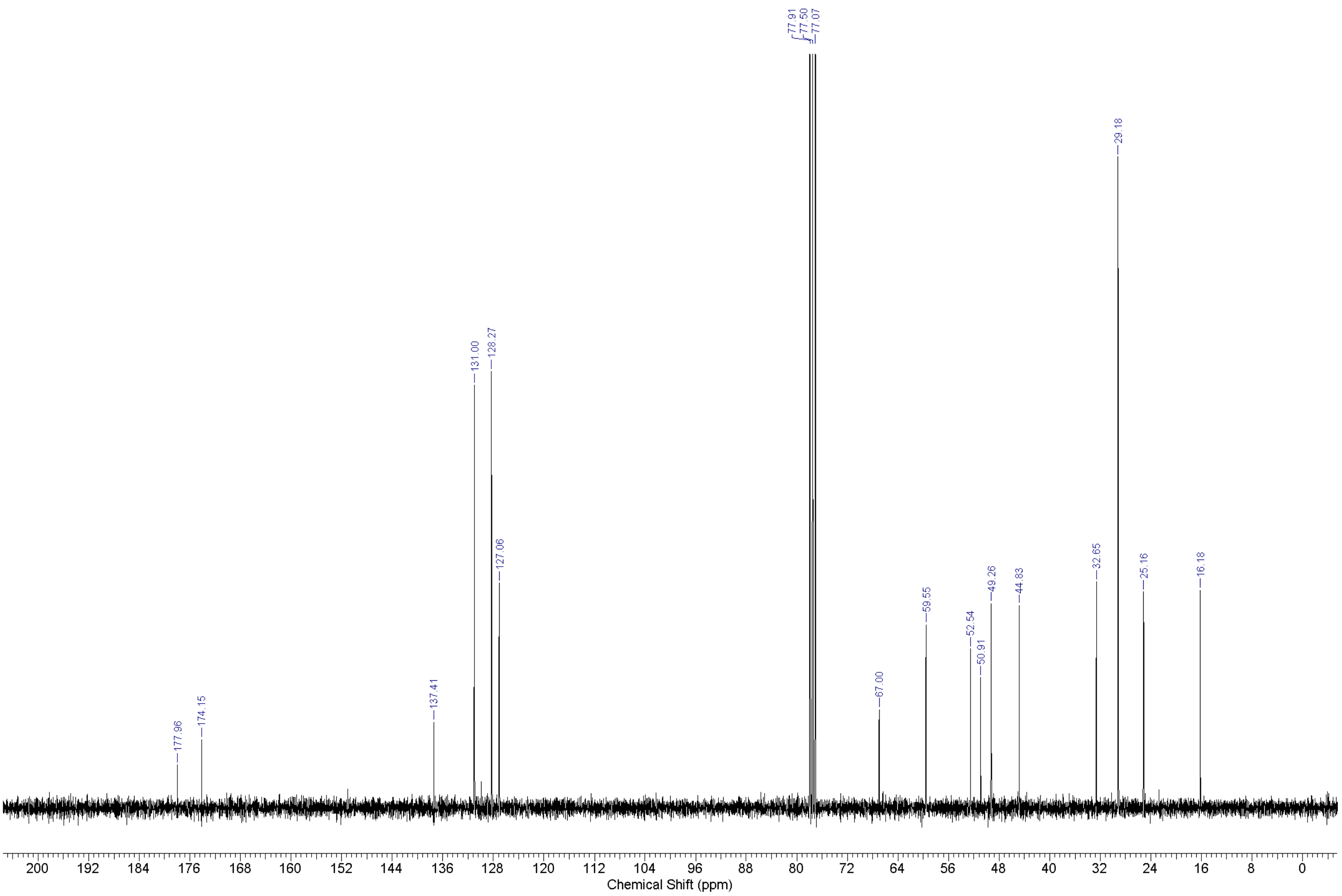
177.96
174.15
137.41
131.00
128.27
127.06
77.91
77.50
77.07
67.00
59.55
52.54
50.91
49.26
44.83
32.65
29.18
25.16
16.18
Chemical Shift (ppm)

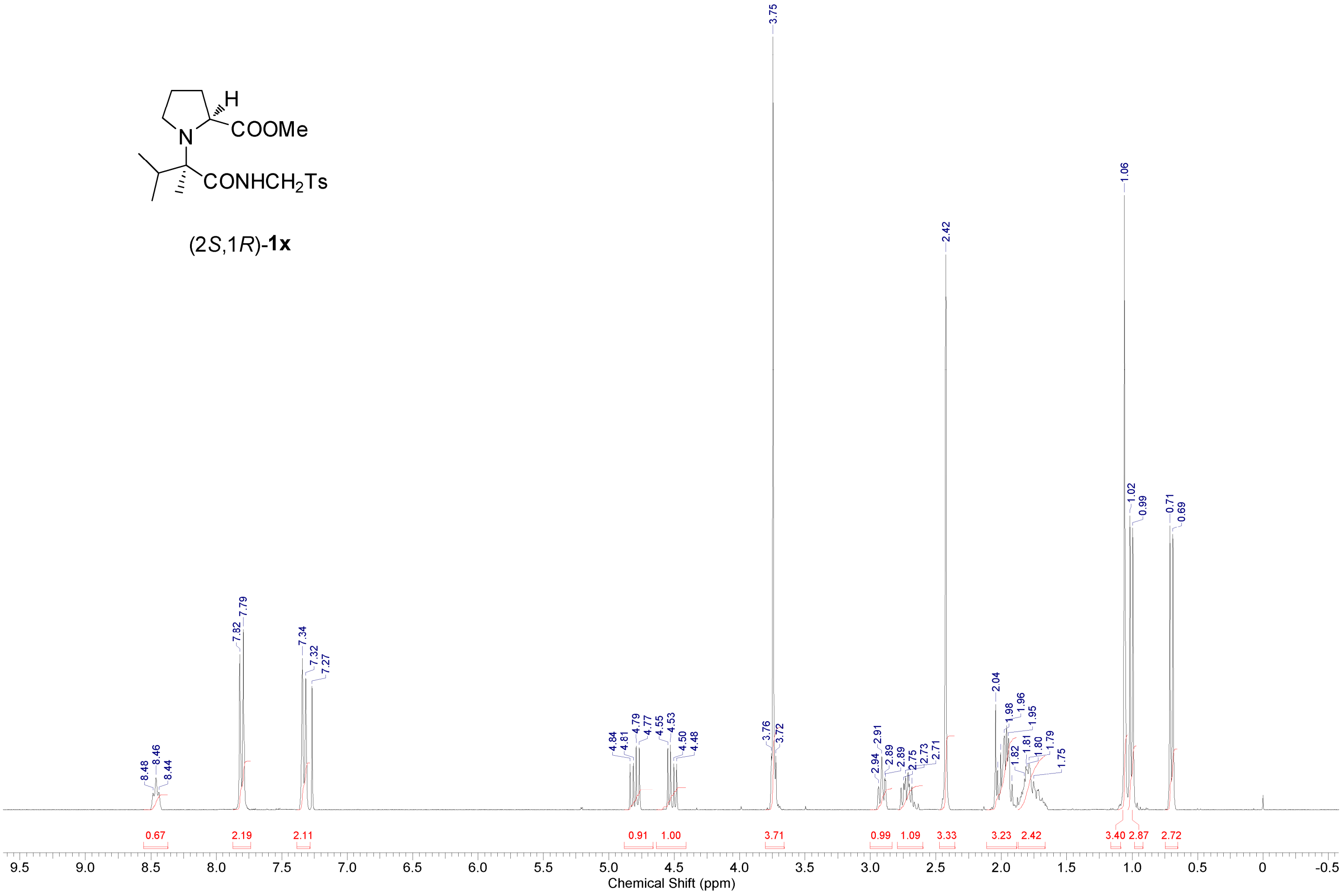
H
COOMe
N
CONHCH2Ts
(2S,1R)-1x
3.75
2.42
1.06
1.02
0.99
0.71
0.69
7.82 7.79
7.34
7.32
7.27
8.48
8.46
8.44
4.84
4.81
4.79
4.77
4.55
4.53
4.50
4.48
3.76
3.72
2.94
2.91
2.89
2.89
2.75
2.73
2.71
2.04
1.98
1.96
1.95
1.82
1.81
1.80
1.79
1.75
0.67
2.19
2.11
0.91
1.00
3.71
0.99
1.09
3.33
3.23
2.42
3.40
2.87
2.72
9.5 9.0 8.5 8.0 7.5 7.0 6.5 6.0 5.5 5.0 4.5 4.0 3.5 3.0 2.5 2.0 1.5 1.0 0.5 0 -0.5
Chemical Shift (ppm)

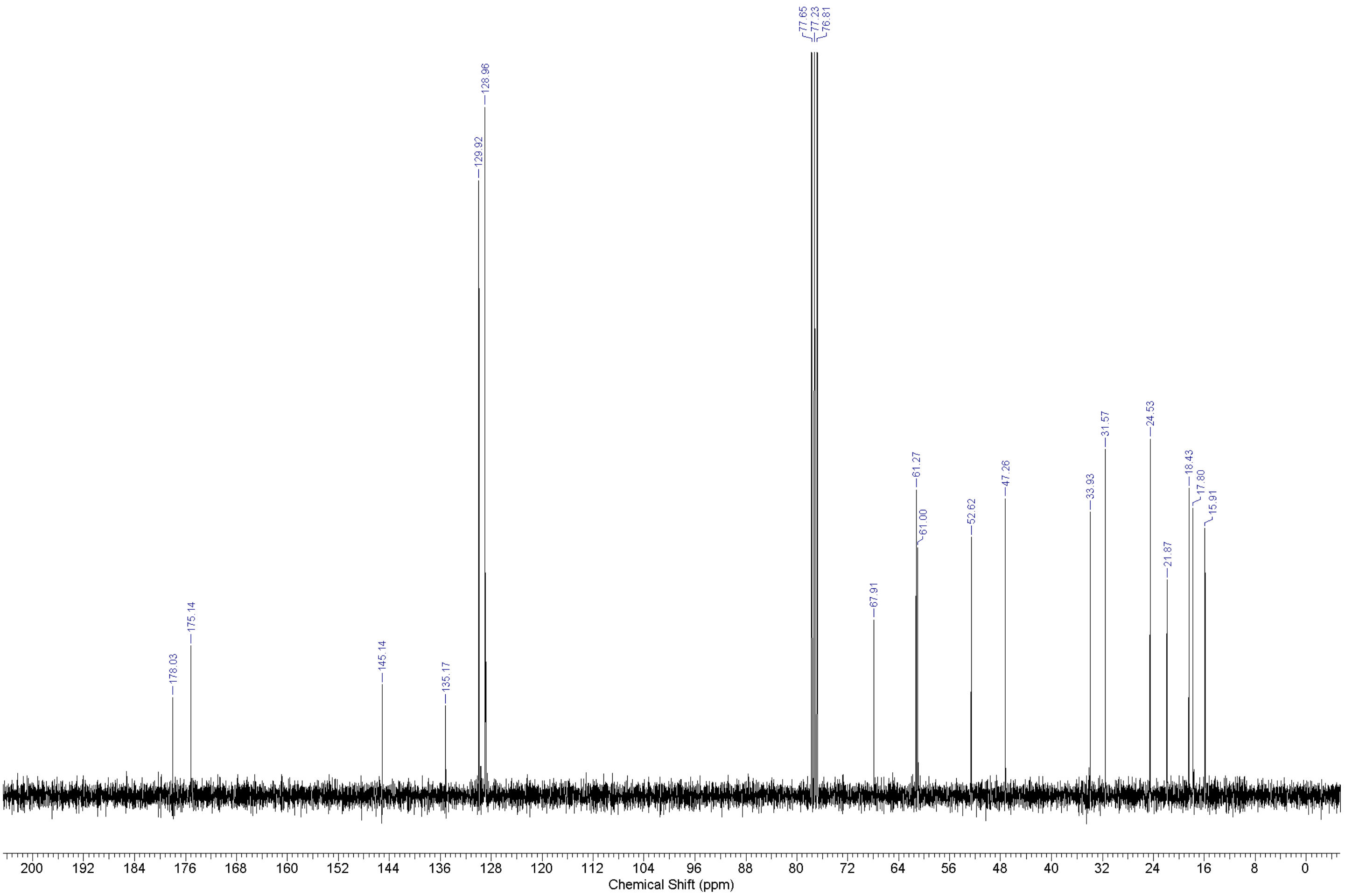
77.65
77.23
76.81
128.96
129.92
61.27
61.00
67.91
52.62
47.26
33.93
31.57
24.53
21.87
18.43
17.80
15.91
178.03
175.14
145.14
135.17
200 192 184 176 168 160 152 144 136 128 120 112 104 96 88 80 72 64 56 48 40 32 24 16 8 0
Chemical Shift (ppm)

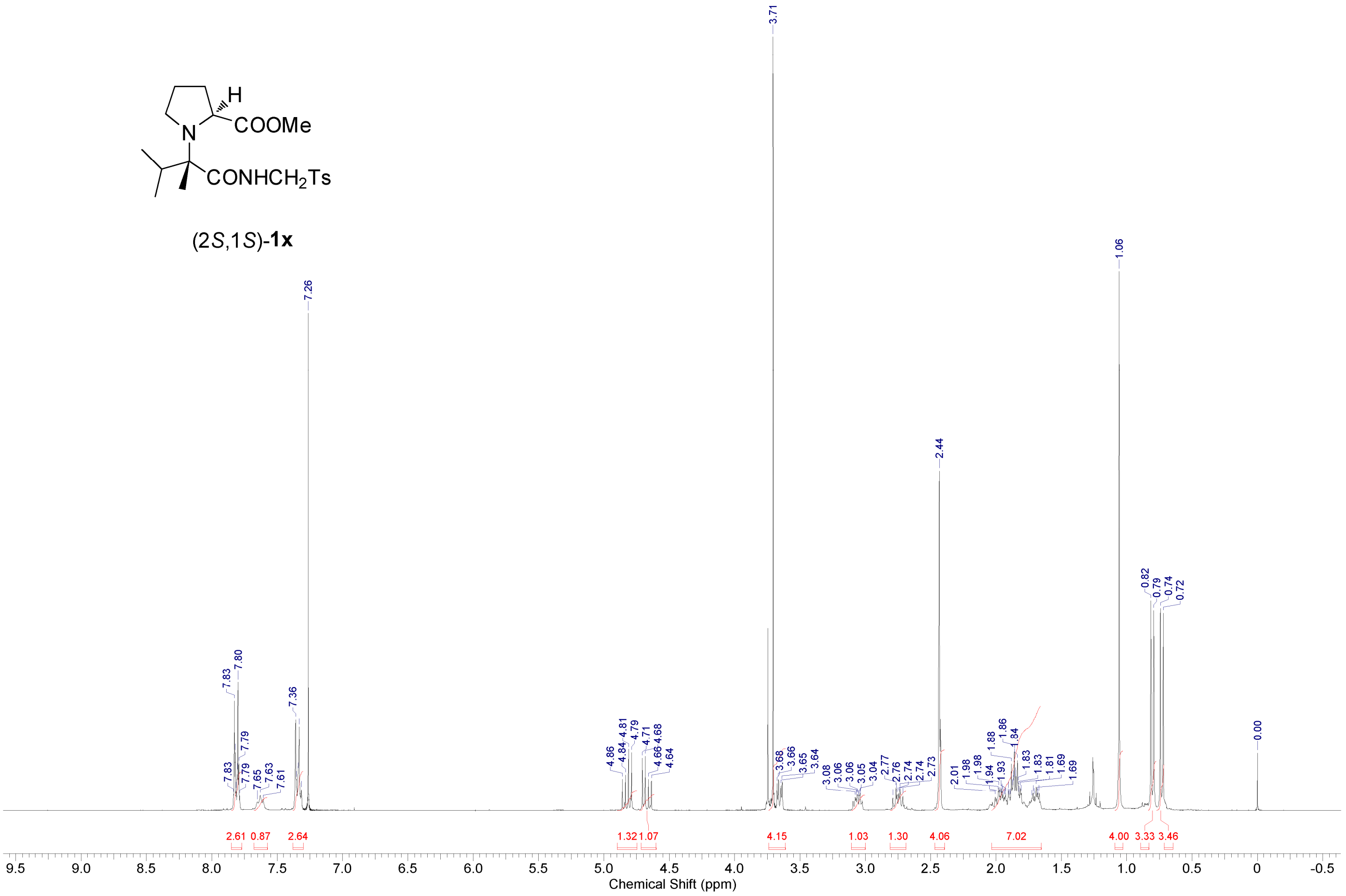
H
COOMe
N
CONHCH2Ts

(2*S*,1*S*)-**1x**

7.83
7.80
7.79
7.83
7.79
7.65
7.63
7.61
7.36
7.26
4.86
4.84
4.81
4.79
4.71
4.68
4.66
4.64
3.71
3.68
3.66
3.65
3.64
3.08
3.06
3.06
3.05
3.04
2.77
2.76
2.74
2.74
2.73
2.44
2.01
1.98
1.98
1.94
1.93
1.88
1.86
1.84
1.83
1.81
1.69
1.69
1.06
0.82
0.79
0.74
0.72
0.00
2.61 0.87 2.64
1.32 1.07
4.15
1.03
1.30
4.06
7.02
4.00 3.33 3.46
9.5 9.0 8.5 8.0 7.5 7.0 6.5 6.0 5.5 5.0 4.5 4.0 3.5 3.0 2.5 2.0 1.5 1.0 0.5 0 -0.5
Chemical Shift (ppm)

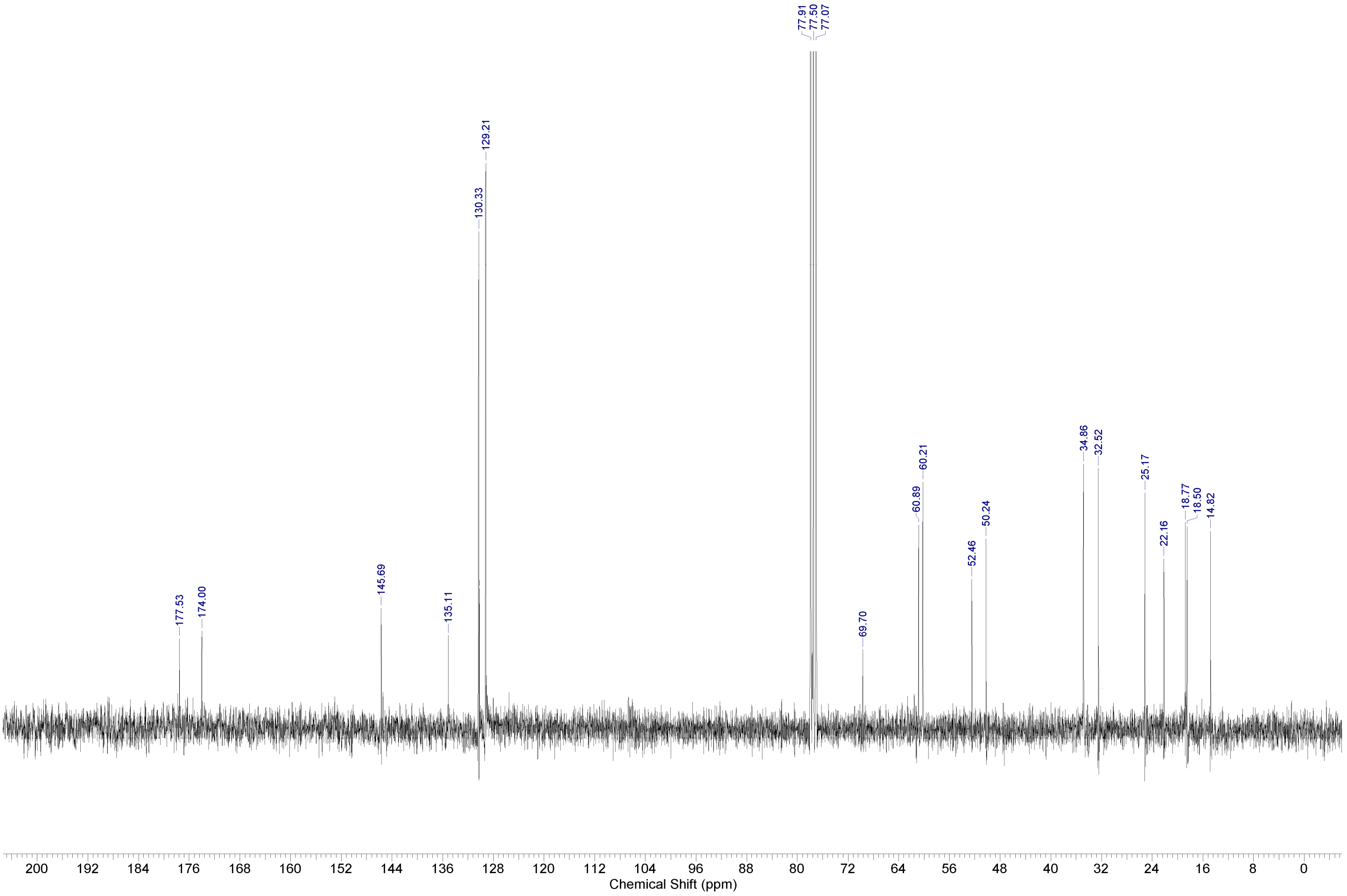
177.53
174.00
145.69
135.11
130.33
129.21
77.91
77.50
77.07
69.70
60.89
60.21
52.46
50.24
34.86
32.52
25.17
22.16
18.77
18.50
14.82
200 192 184 176 168 160 152 144 136 128 120 112 104 96 88 80 72 64 56 48 40 32 24 16 8 0
Chemical Shift (ppm)

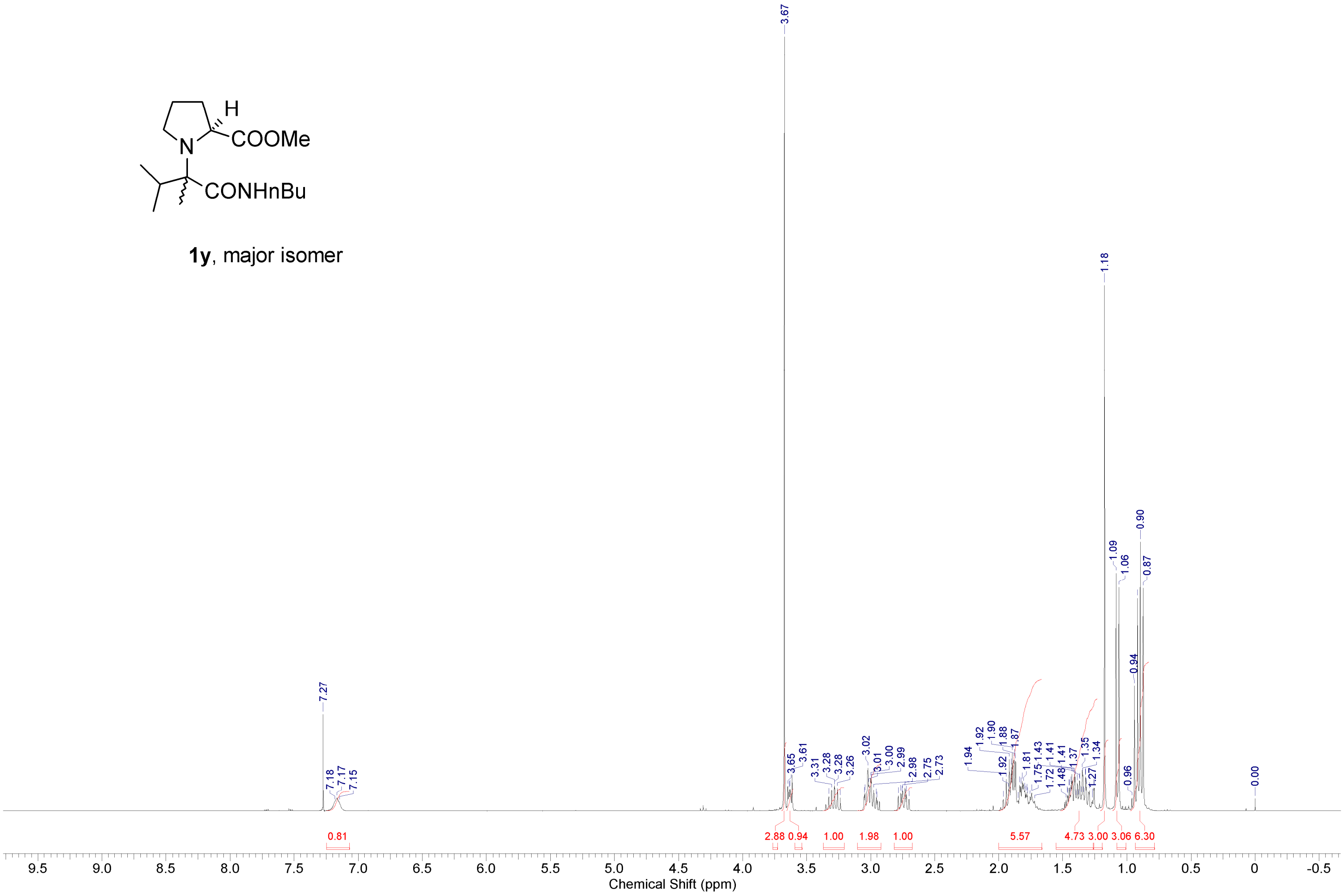
H
N
COOMe
CONHnBu
1y, major isomer
3.67
1.18
1.09
1.06
0.90
0.87
0.94
0.96
7.27
7.18
7.17
7.15
3.65
3.61
3.31
3.28
3.28
3.26
3.02
3.01
3.00
2.99
2.98
2.75
2.73
1.94
1.92
1.92
1.90
1.88
1.87
1.81
1.75
1.72
1.43
1.41
1.41
1.48
1.37
1.35
1.34
1.27
0.00
0.81
2.88
0.94
1.00
1.98
1.00
5.57
4.73
3.00
3.06
6.30
9.5
9.0
8.5
8.0
7.5
7.0
6.5
6.0
5.5
5.0
4.5
4.0
3.5
3.0
2.5
2.0
1.5
1.0
0.5
0
-0.5
Chemical Shift (ppm)

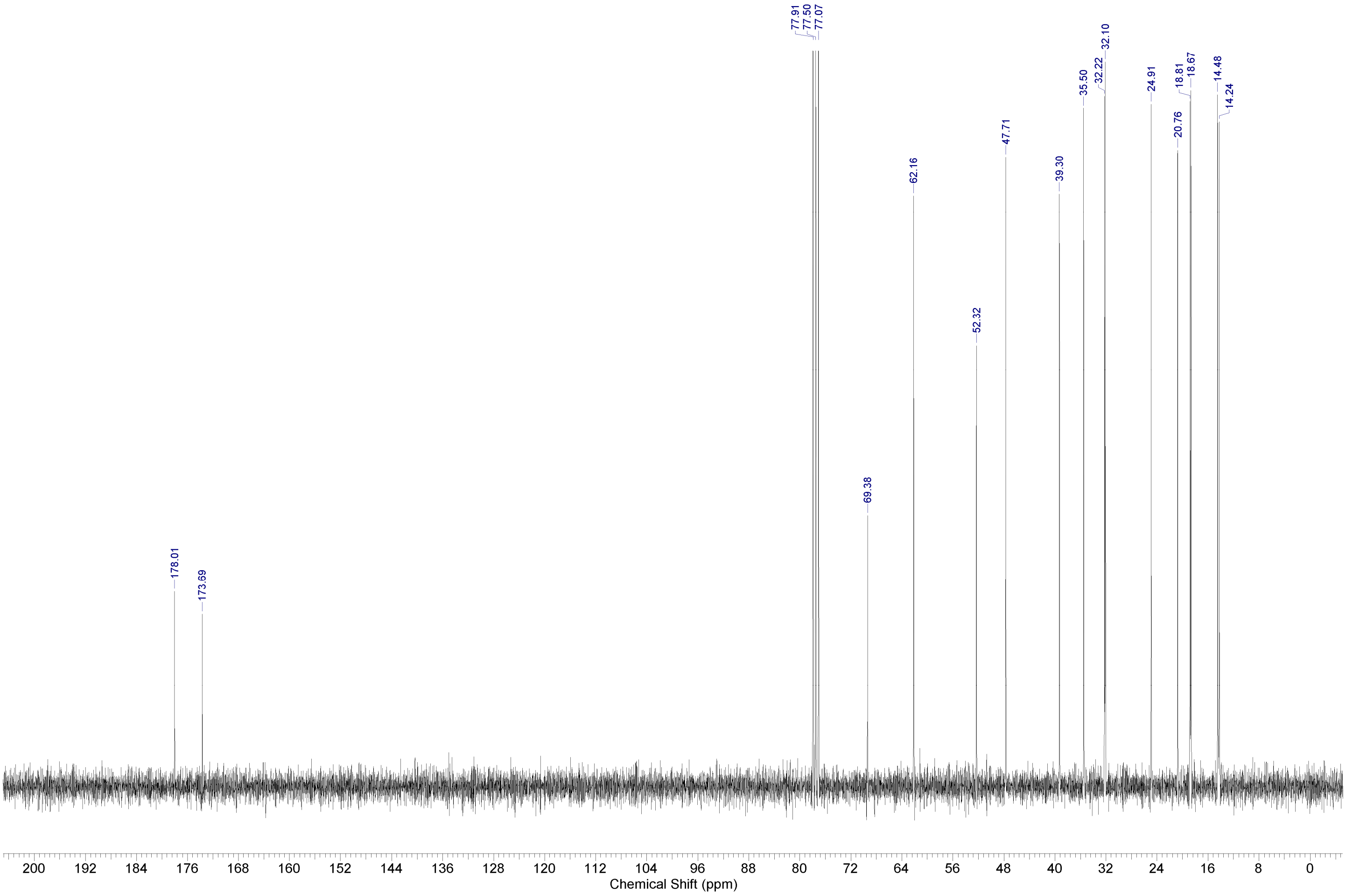
77.91
77.50
77.07
32.10
35.50
32.22
24.91
18.81
18.67
14.48
14.24
20.76
47.71
62.16
39.30
52.32
69.38
178.01
173.69
200
192
184
176
168
160
152
144
136
128
120
112
104
96
88
80
72
64
56
48
40
32
24
16
8
0
Chemical Shift (ppm)

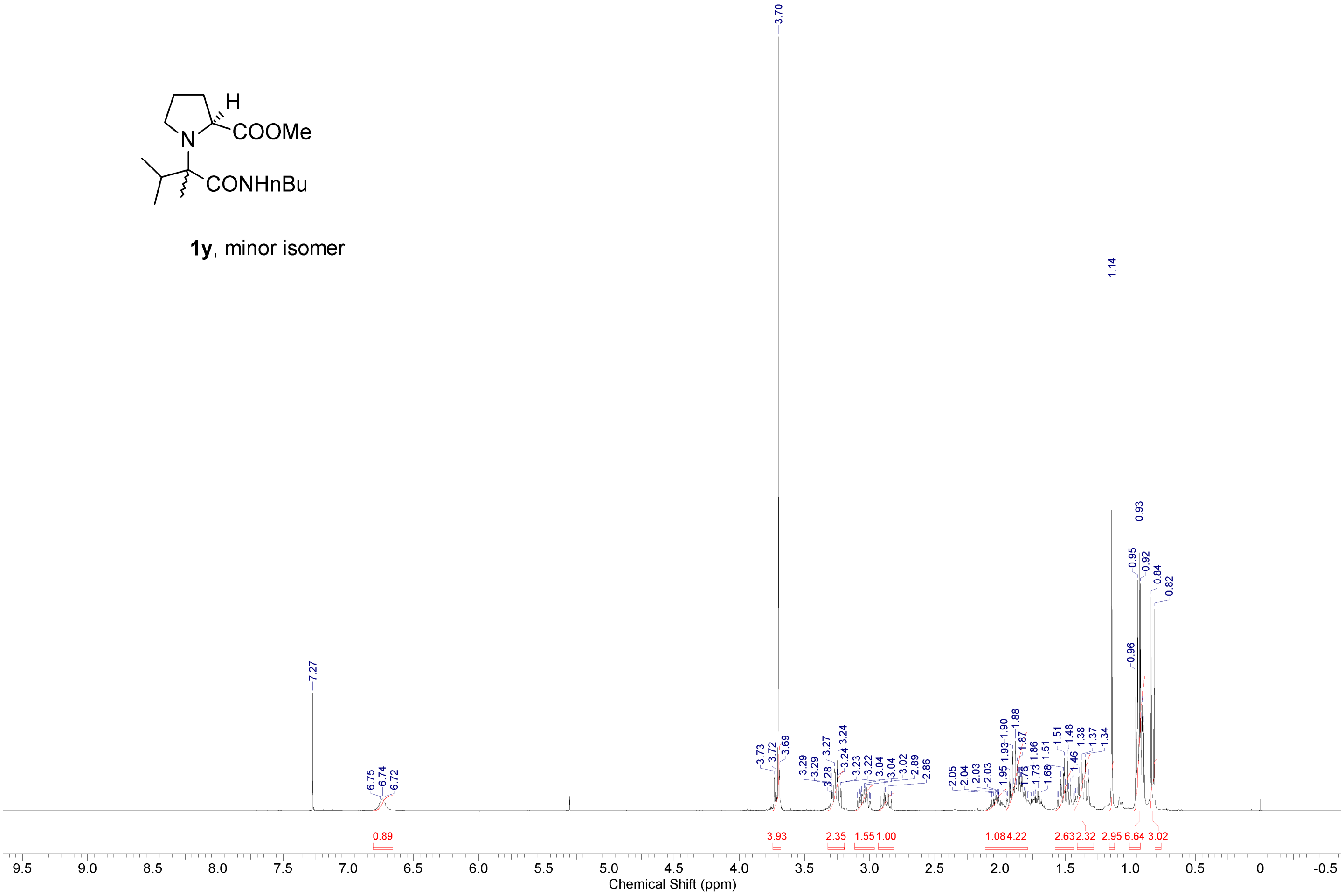

1y, minor isomer

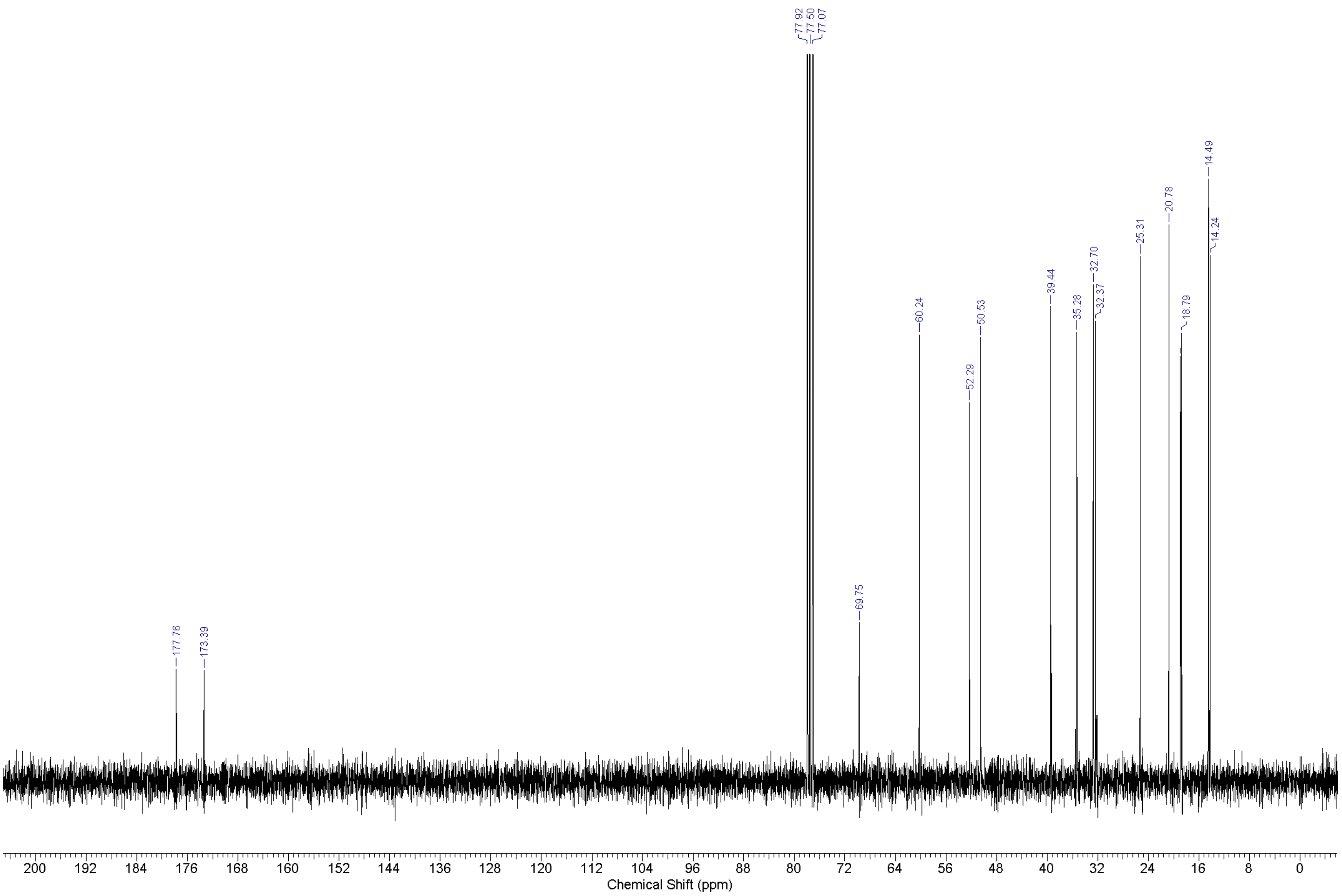

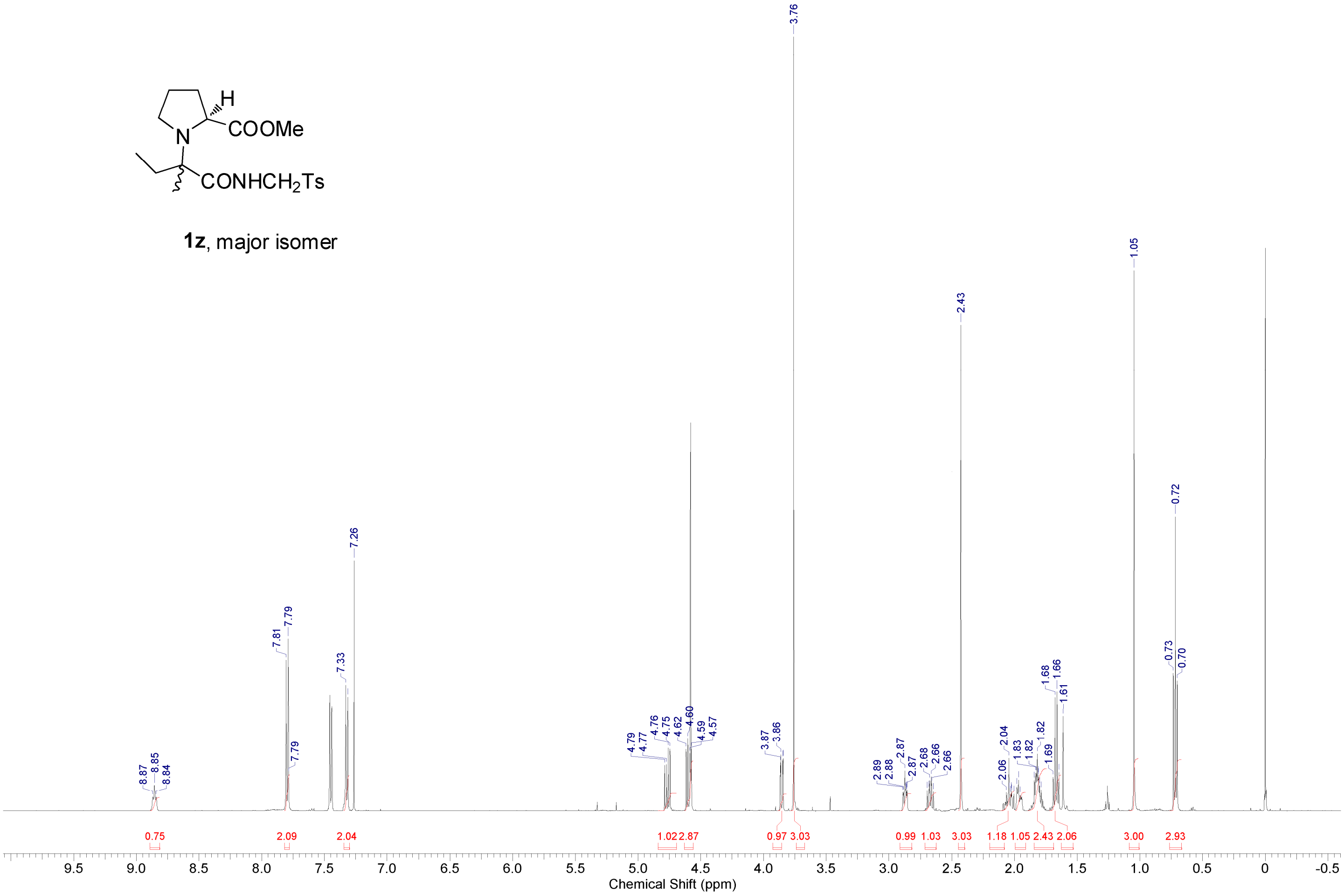
H
COOMe
N
CONHCH2Ts
1z, major isomer
3.76
1.05
2.43
0.72
7.26
7.81
7.79
7.33
0.73
0.70
1.68
1.66
1.61
7.79
8.87
8.85
8.84
4.79
4.77
4.76
4.75
4.62
4.60
4.59
4.57
3.87
3.86
2.89
2.88
2.87
2.87
2.68
2.66
2.66
2.06
2.04
1.83
1.82
1.82
1.69
0.75
2.09
2.04
1.02
2.87
0.97
3.03
0.99
1.03
3.03
1.18
1.05
2.43
2.06
3.00
2.93
9.5
9.0
8.5
8.0
7.5
7.0
6.5
6.0
5.5
5.0
4.5
4.0
3.5
3.0
2.5
2.0
1.5
1.0
0.5
0
-0.5
Chemical Shift (ppm)

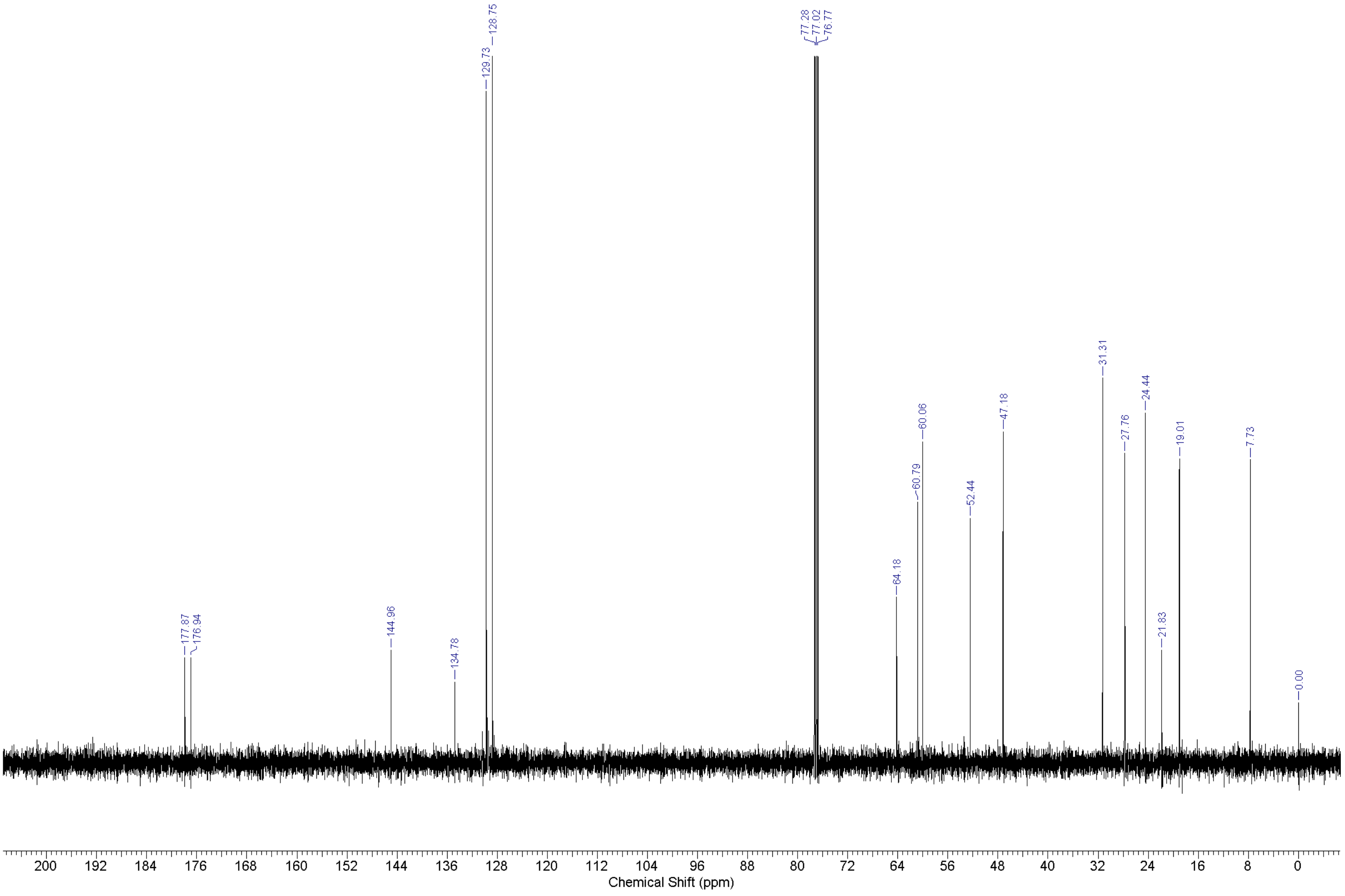
129.73
128.75
77.28
77.02
76.77
177.87
176.94
144.96
134.78
64.18
60.79
60.06
52.44
47.18
31.31
27.76
24.44
21.83
19.01
7.73
0.00
200
192
184
176
168
160
152
144
136
128
120
112
104
96
88
80
72
64
56
48
40
32
24
16
8
0
Chemical Shift (ppm)

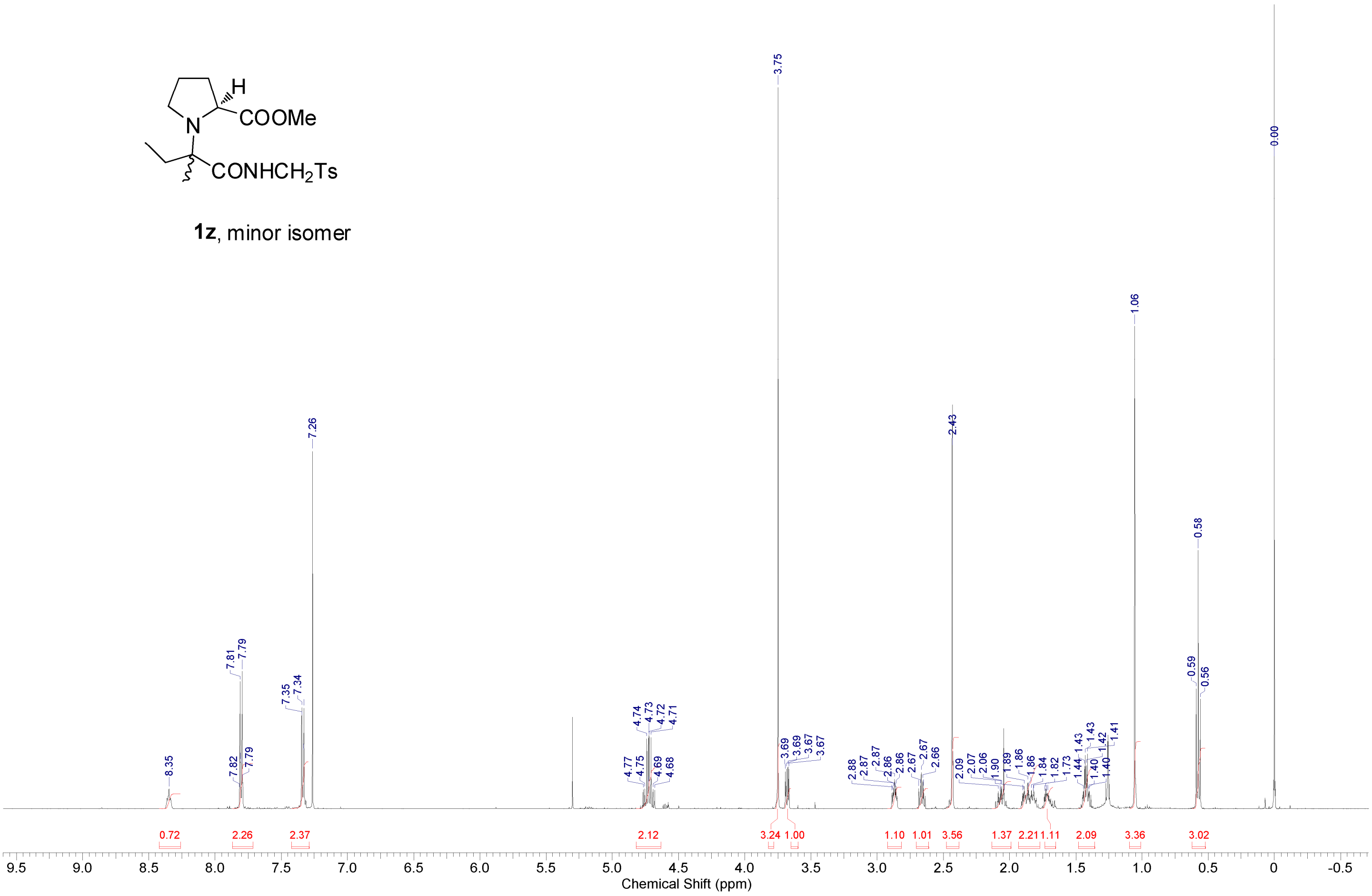
H
COOMe
N
CONHCH2Ts
1z, minor isomer
8.35
7.82
7.81
7.79
7.79
7.35
7.34
7.26
4.77
4.75
4.74
4.73
4.72
4.71
4.69
4.68
3.75
3.69
3.69
3.67
3.67
2.88
2.87
2.87
2.86
2.86
2.67
2.67
2.66
2.43
2.09
2.07
2.06
1.90
1.89
1.86
1.86
1.84
1.82
1.73
1.44
1.43
1.43
1.42
1.41
1.40
1.40
1.06
0.59
0.58
0.56
0.00
0.72
2.26
2.37
2.12
3.24
1.00
1.10
1.01
3.56
1.37
2.21
1.11
2.09
3.36
3.02
9.5
9.0
8.5
8.0
7.5
7.0
6.5
6.0
5.5
5.0
4.5
4.0
3.5
3.0
2.5
2.0
1.5
1.0
0.5
0
-0.5
Chemical Shift (ppm)

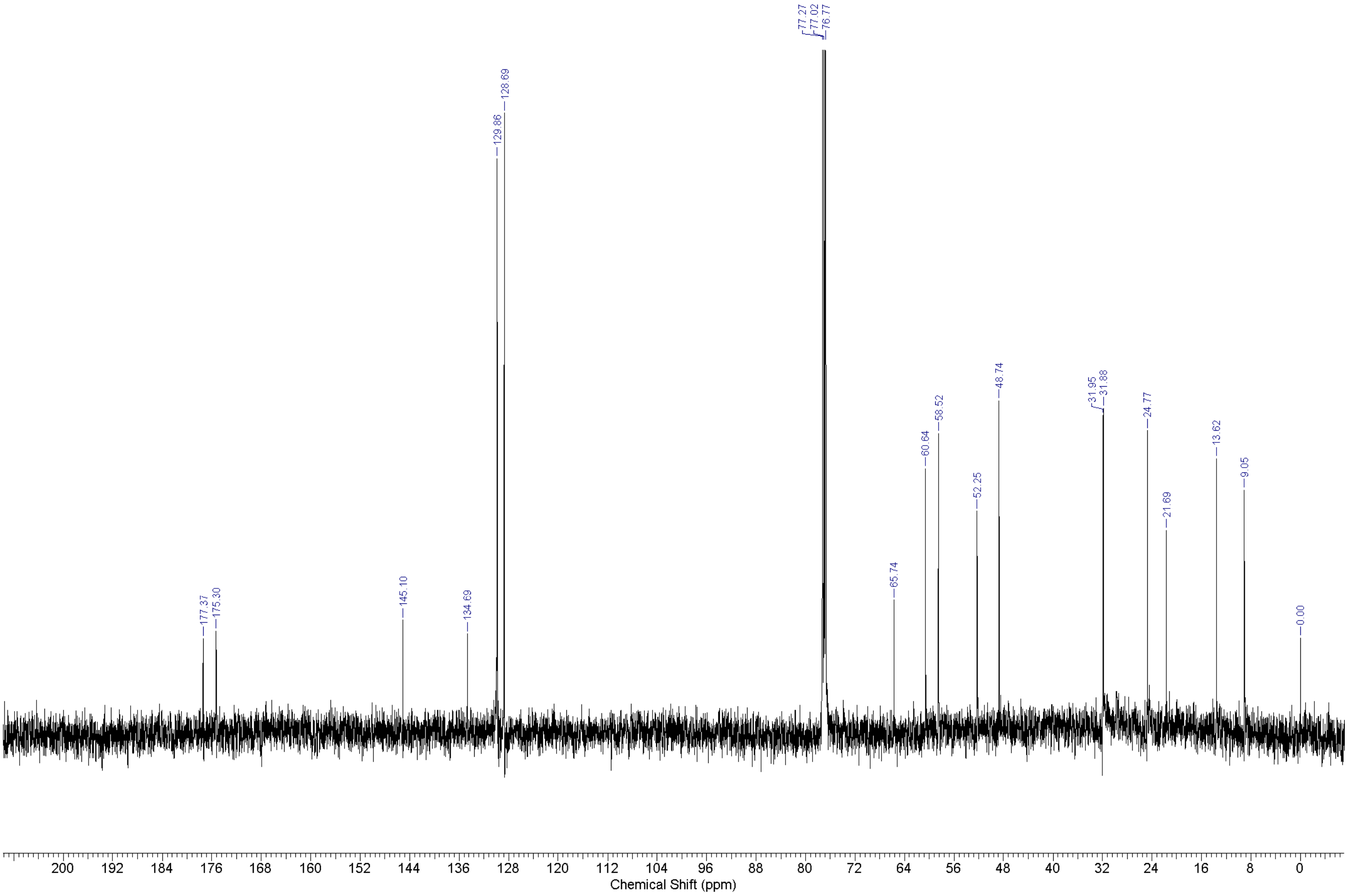
177.37
175.30
145.10
134.69
129.86
128.69
77.27
77.02
76.77
65.74
60.64
58.52
52.25
48.74
31.95
31.88
24.77
21.69
13.62
9.05
0.00
200
192
184
176
168
160
152
144
136
128
120
112
104
96
88
80
72
64
56
48
40
32
24
16
8
0
Chemical Shift (ppm)

(2*S*,1*R*)-**2u**

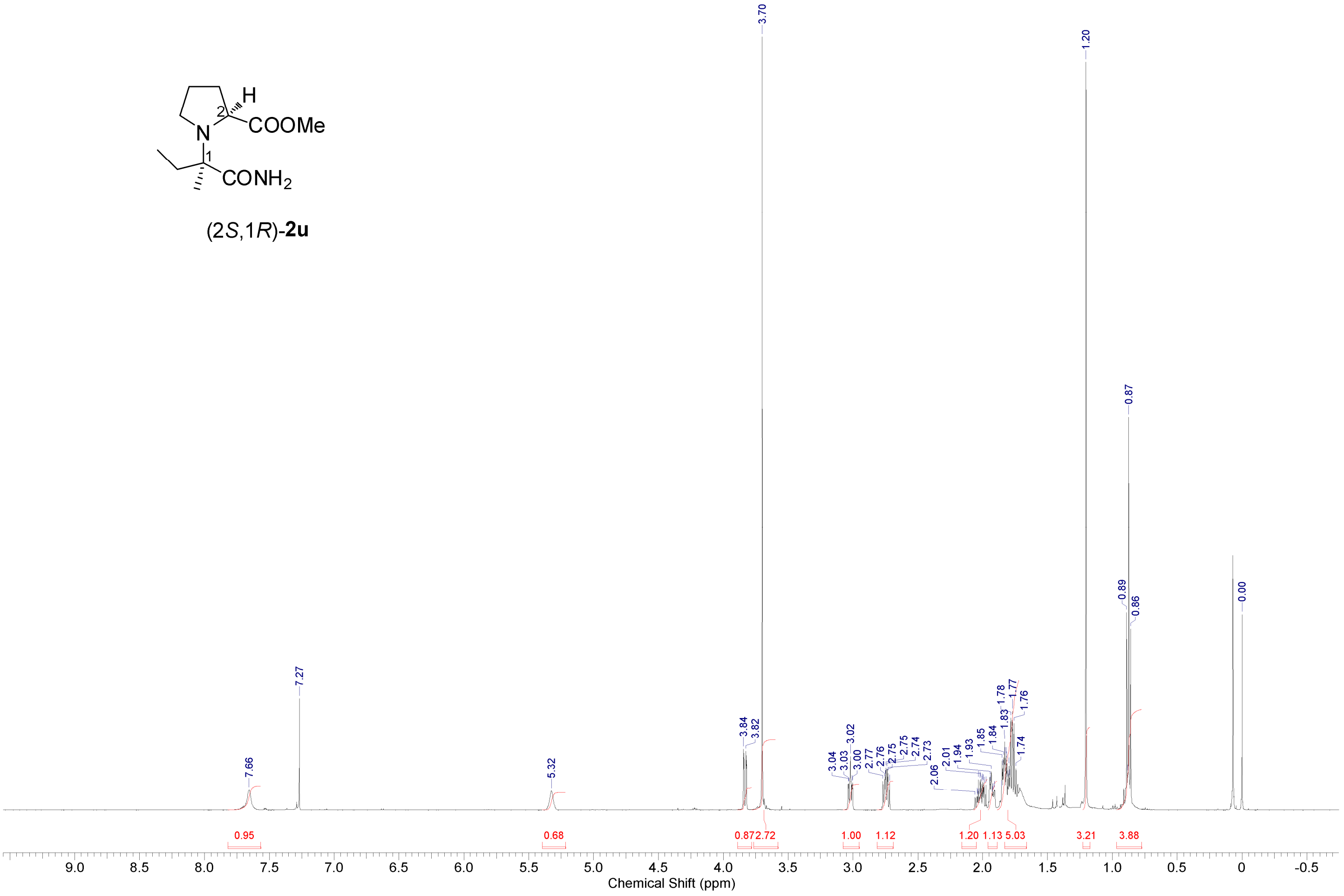
H
COOMe
N
2
1
CONH2
3.70
1.20
0.87
0.89
0.86
0.00
7.27
7.66
5.32
3.84
3.82
3.04
3.03
3.02
3.00
2.77
2.76
2.75
2.75
2.74
2.73
2.06
2.01
1.94
1.93
1.85
1.84
1.83
1.78
1.77
1.76
1.74
0.95
0.68
0.87
2.72
1.00
1.12
1.20
1.13
5.03
3.21
3.88
9.0
8.5
8.0
7.5
7.0
6.5
6.0
5.5
5.0
4.5
4.0
3.5
3.0
2.5
2.0
1.5
1.0
0.5
0
-0.5
Chemical Shift (ppm)

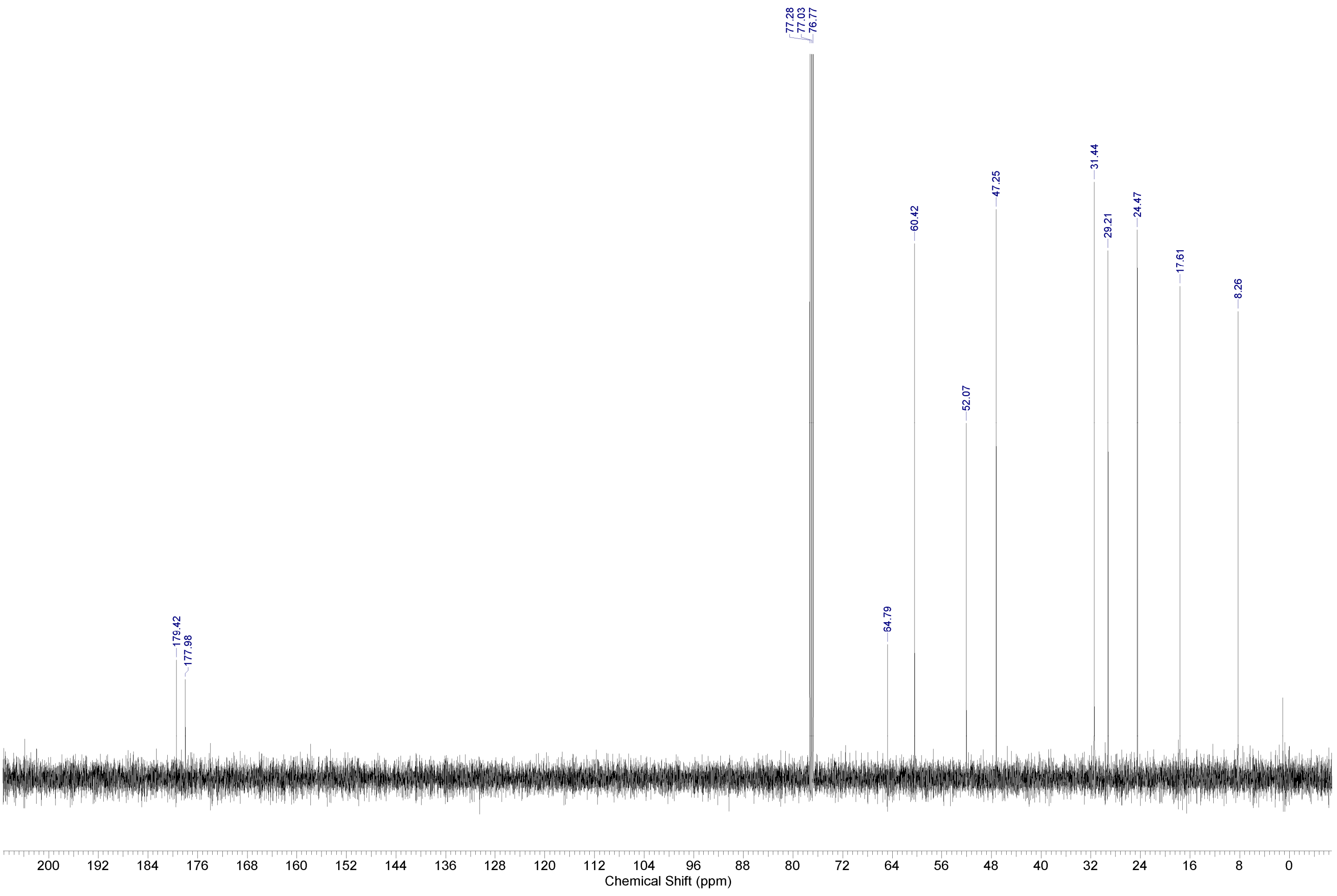
77.28
77.03
76.77
60.42
47.25
31.44
29.21
24.47
17.61
8.26
52.07
64.79
179.42
177.98
200
192
184
176
168
160
152
144
136
128
120
112
104
96
88
80
72
64
56
48
40
32
24
16
8
0
Chemical Shift (ppm)

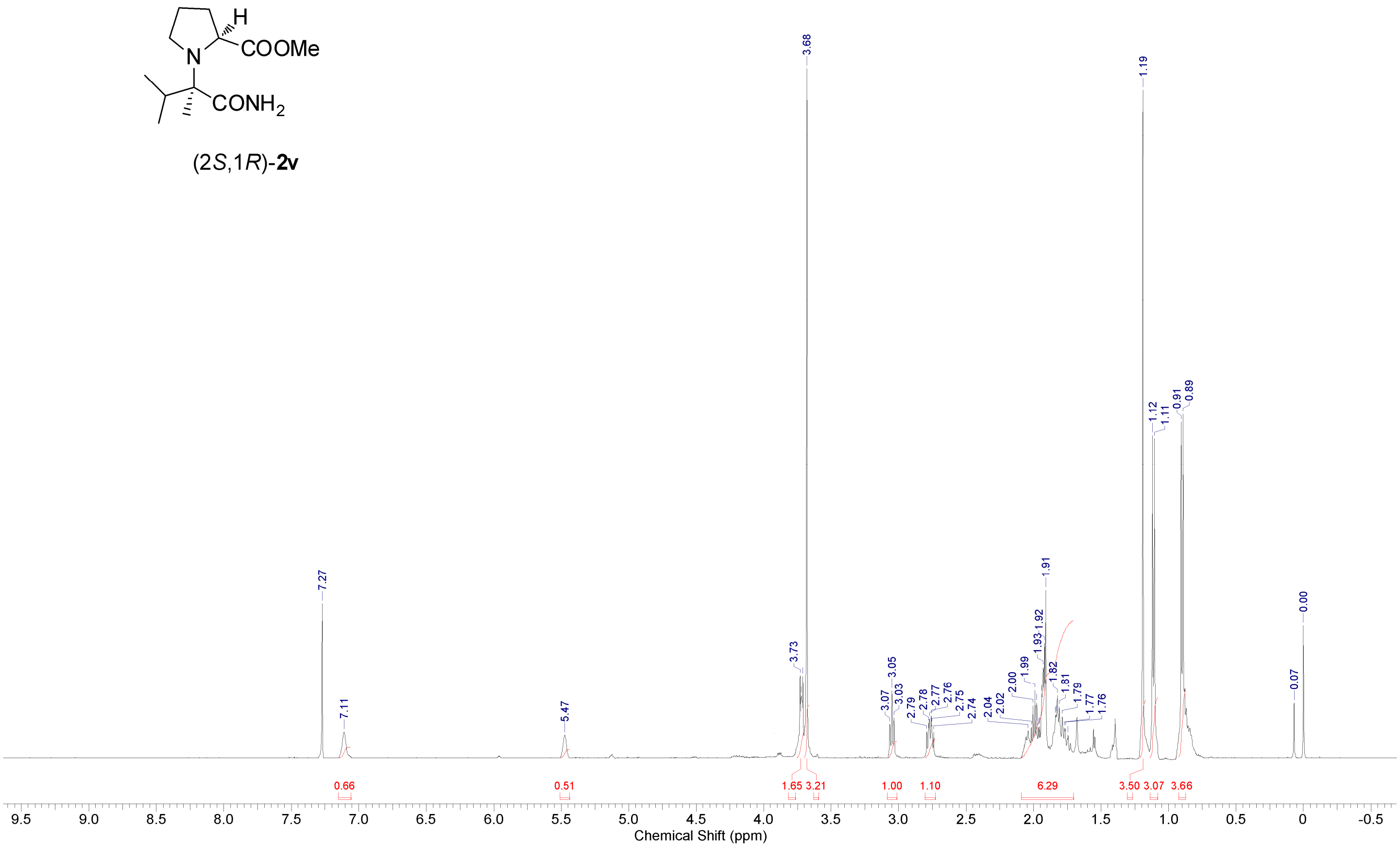
H
COOMe
N
CONH2
(2*S*,1*R*)-**2v**
7.27
7.11
5.47
3.73
3.68
3.07
3.05
3.03
2.79
2.78
2.77
2.76
2.75
2.74
2.04
2.02
2.00
1.99
1.93
1.92
1.91
1.82
1.81
1.79
1.77
1.76
1.19
1.12
1.11
0.91
0.89
0.07
0.00
0.66
0.51
1.65
3.21
1.00
1.10
6.29
3.50
3.07
3.66
Chemical Shift (ppm)

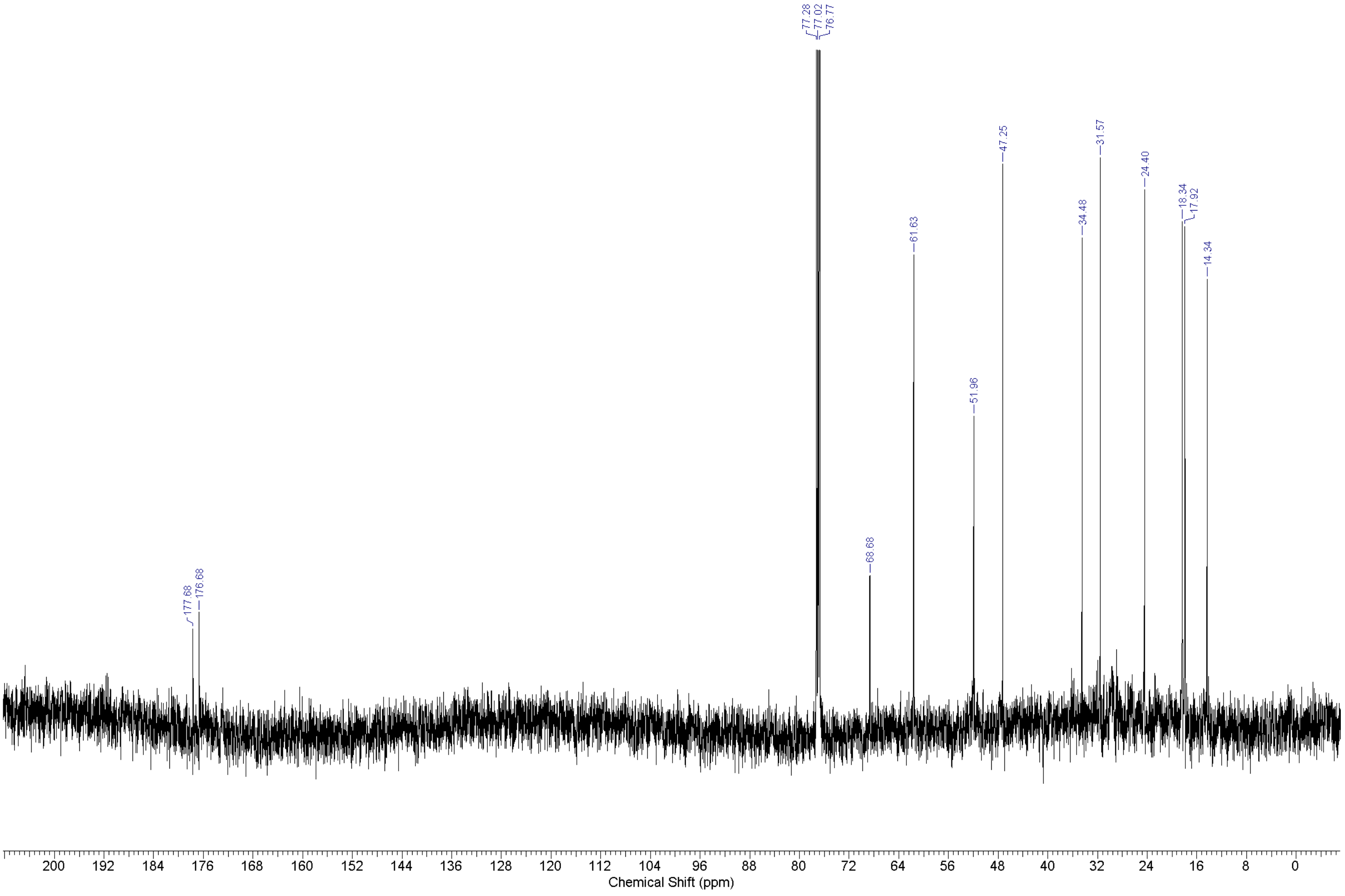
177.68
176.68
77.28
77.02
76.77
68.68
61.63
51.96
47.25
34.48
31.57
24.40
18.34
17.92
14.34
Chemical Shift (ppm)

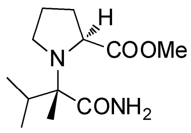
H
N
COOMe
CONH2

(2*S*,1*S*)-**2v**

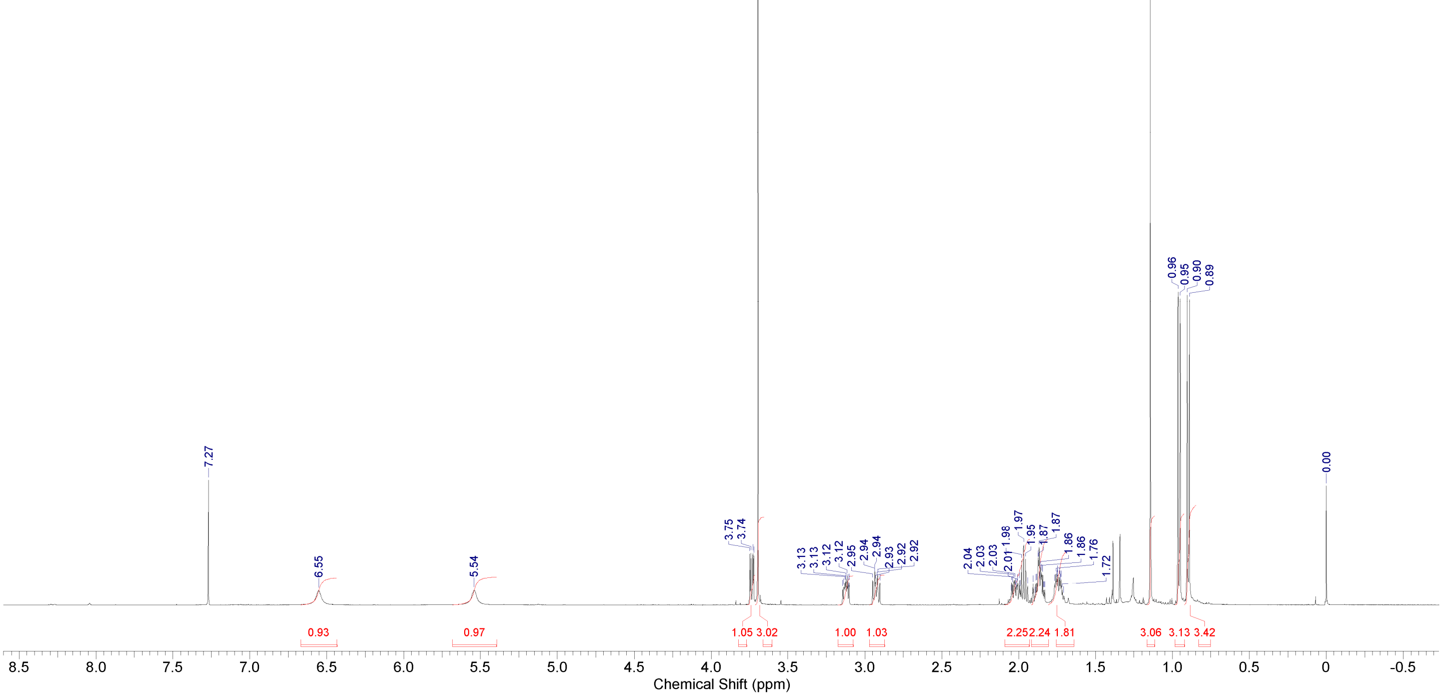
3.70
1.14
0.96 0.95 0.90 0.89
7.27
0.00
6.55
5.54
3.75 3.74
3.13 3.13 3.12 2.95 2.94 2.94 2.93 2.92 2.92
2.04 2.03 2.03 2.01 1.98 1.97 1.95 1.87 1.87 1.86 1.86 1.76 1.72
0.93 0.97 1.05 3.02 1.00 1.03 2.25 2.24 1.81 3.06 3.13 3.42
8.5 8.0 7.5 7.0 6.5 6.0 5.5 5.0 4.5 4.0 3.5 3.0 2.5 2.0 1.5 1.0 0.5 0 -0.5
Chemical Shift (ppm)

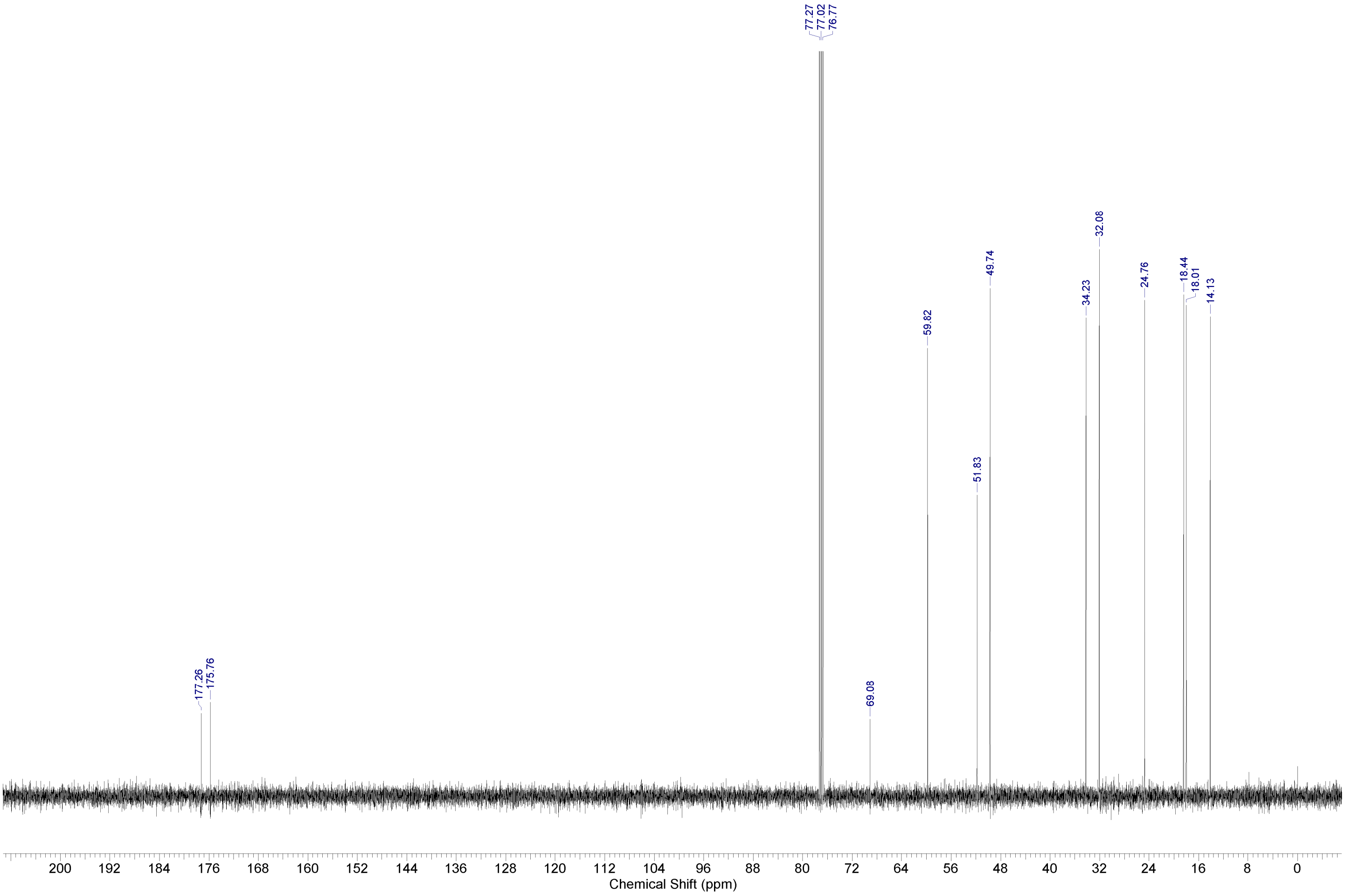
77.27 77.02 76.77
32.08
49.74
34.23
24.76
18.44 18.01 14.13
59.82
51.83
177.26 175.76
69.08
200 192 184 176 168 160 152 144 136 128 120 112 104 96 88 80 72 64 56 48 40 32 24 16 8 0
Chemical Shift (ppm)

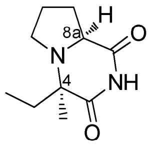
H
8a
O
N
NH
4
O

(4*R*,8a*S*)-**3u**

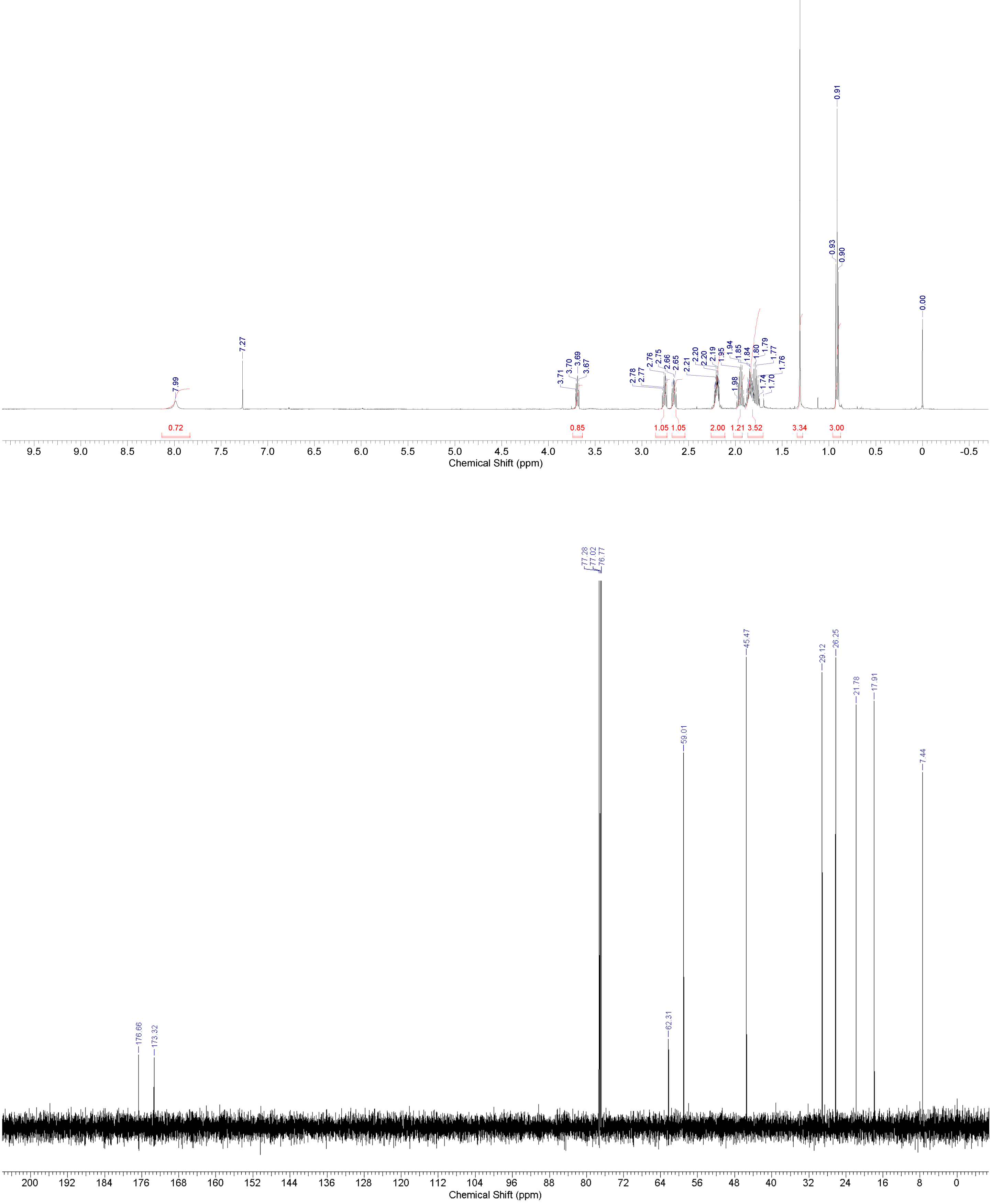
1.31
0.91
0.93
0.90
0.00
7.27
7.99
3.71
3.70
3.69
3.67
2.78
2.77
2.76
2.75
2.66
2.65
2.21
2.20
2.20
2.19
1.95
1.94
1.85
1.84
1.80
1.79
1.77
1.76
1.98
1.74
1.70
0.72
0.85
1.05
1.05
2.00
1.21
3.52
3.34
3.00
Chemical Shift (ppm)
77.28
77.02
76.77
45.47
29.12
26.25
21.78
17.91
59.01
7.44
176.66
173.32
62.31
Chemical Shift (ppm)

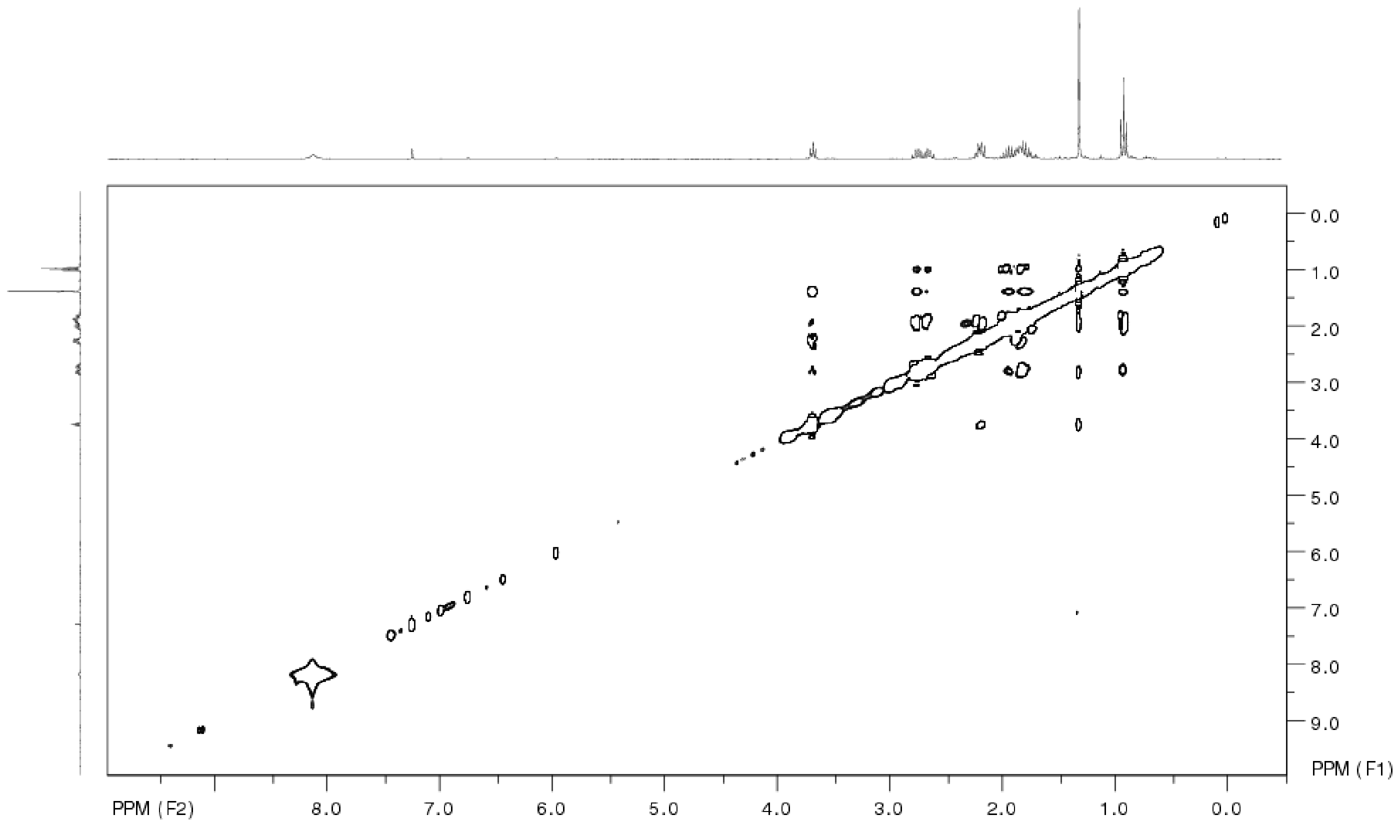
PPM (F2)
8.0
7.0
6.0
5.0
4.0
3.0
2.0
1.0
0.0
PPM (F1)
0.0
1.0
2.0
3.0
4.0
5.0
6.0
7.0
8.0
9.0

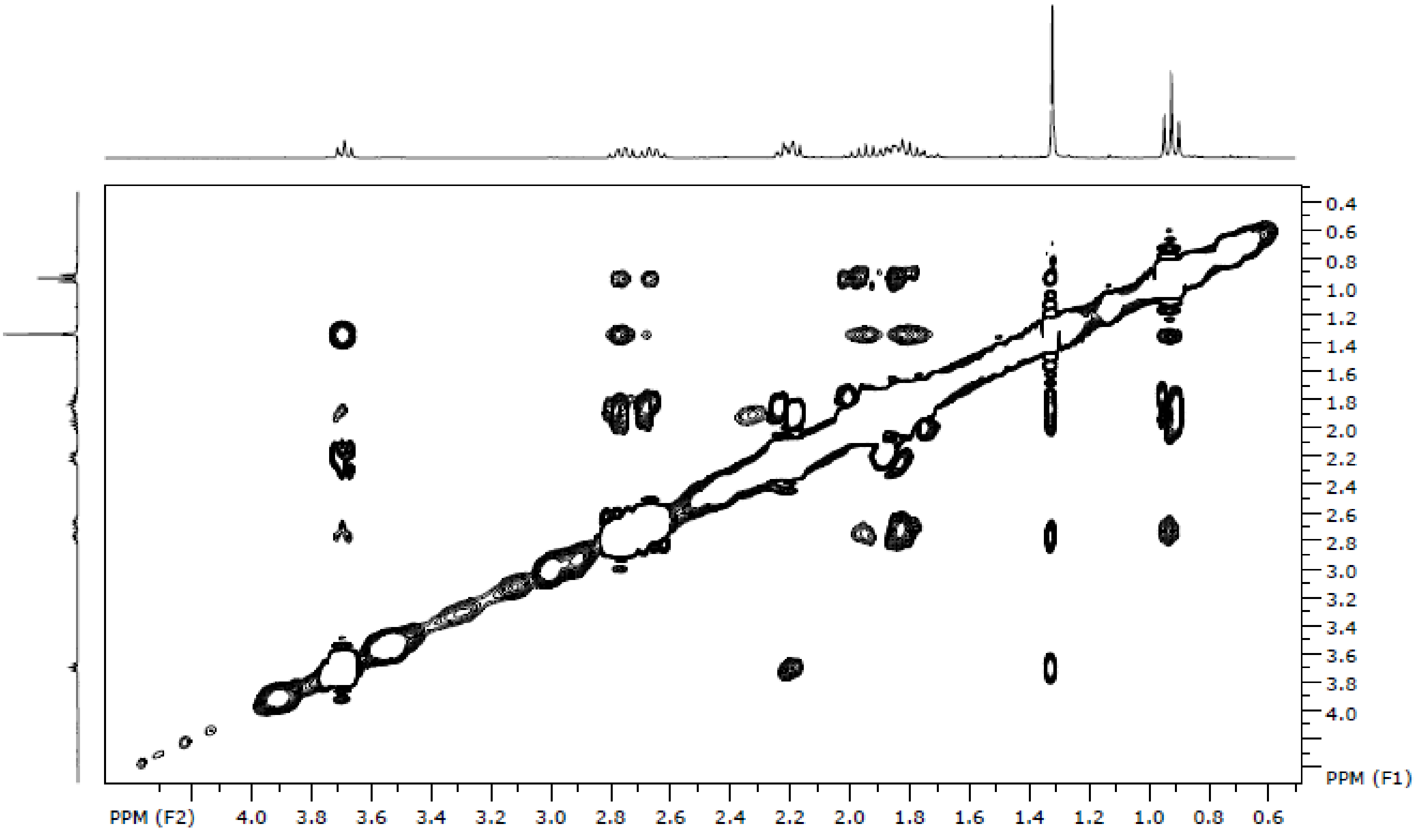
PPM (F2)
4.0
3.8
3.6
3.4
3.2
3.0
2.8
2.6
2.4
2.2
2.0
1.8
1.6
1.4
1.2
1.0
0.8
0.6
PPM (F1)
0.4
0.6
0.8
1.0
1.2
1.4
1.6
1.8
2.0
2.2
2.4
2.6
2.8
3.0
3.2
3.4
3.6
3.8
4.0

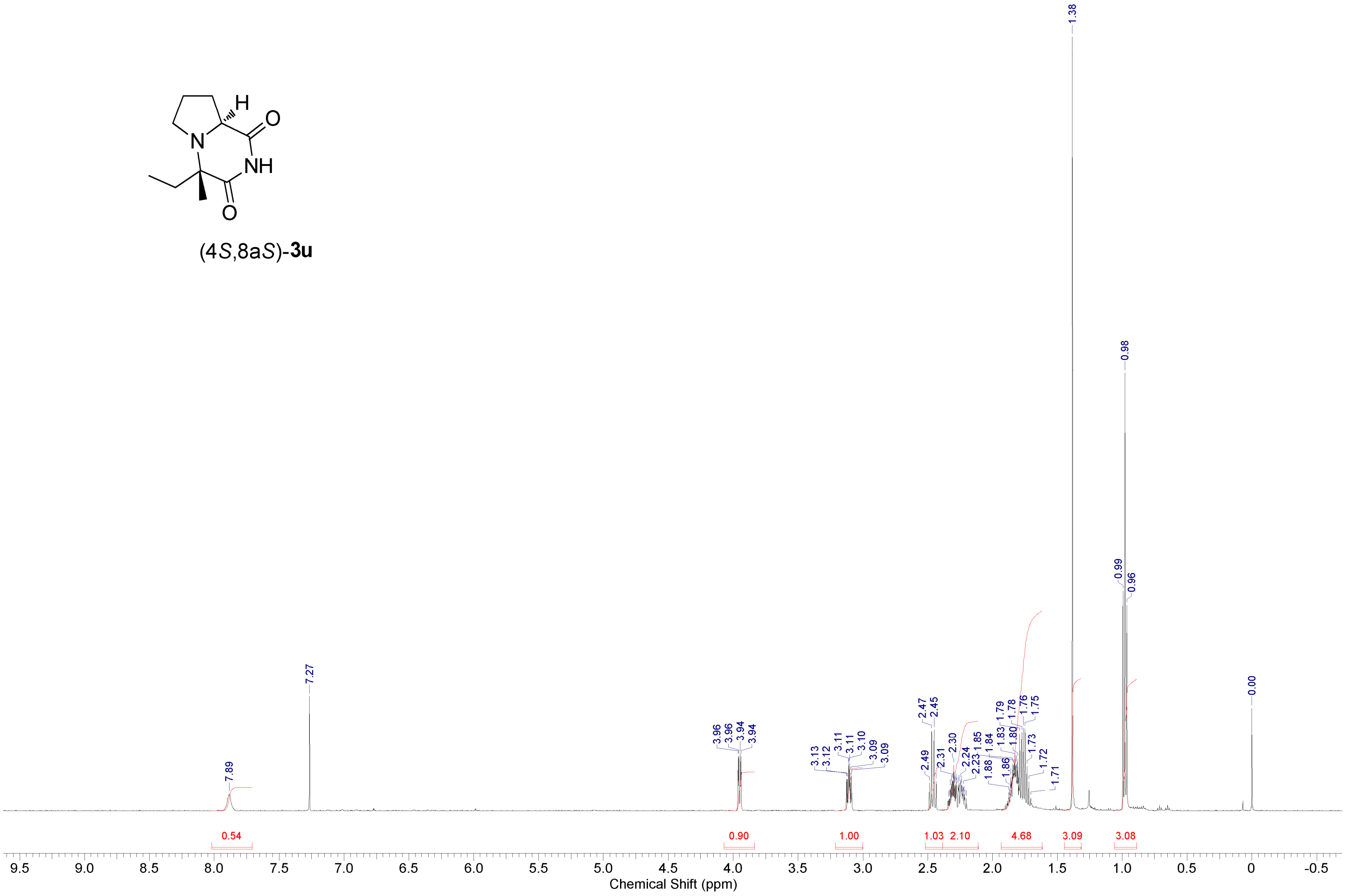
H
O
N
NH
O
7.89
7.27
3.96
3.96
3.94
3.94
3.13
3.12
3.11
3.11
3.10
3.09
3.09
2.49
2.47
2.45
2.31
2.30
2.24
2.23
1.88
1.86
1.85
1.84
1.83
1.80
1.79
1.78
1.76
1.75
1.73
1.72
1.71
1.38
0.99
0.98
0.96
0.00
0.54
0.90
1.00
1.03
2.10
4.68
3.09
3.08
Chemical Shift (ppm)

(4*S*,8a*S*)-**3u**

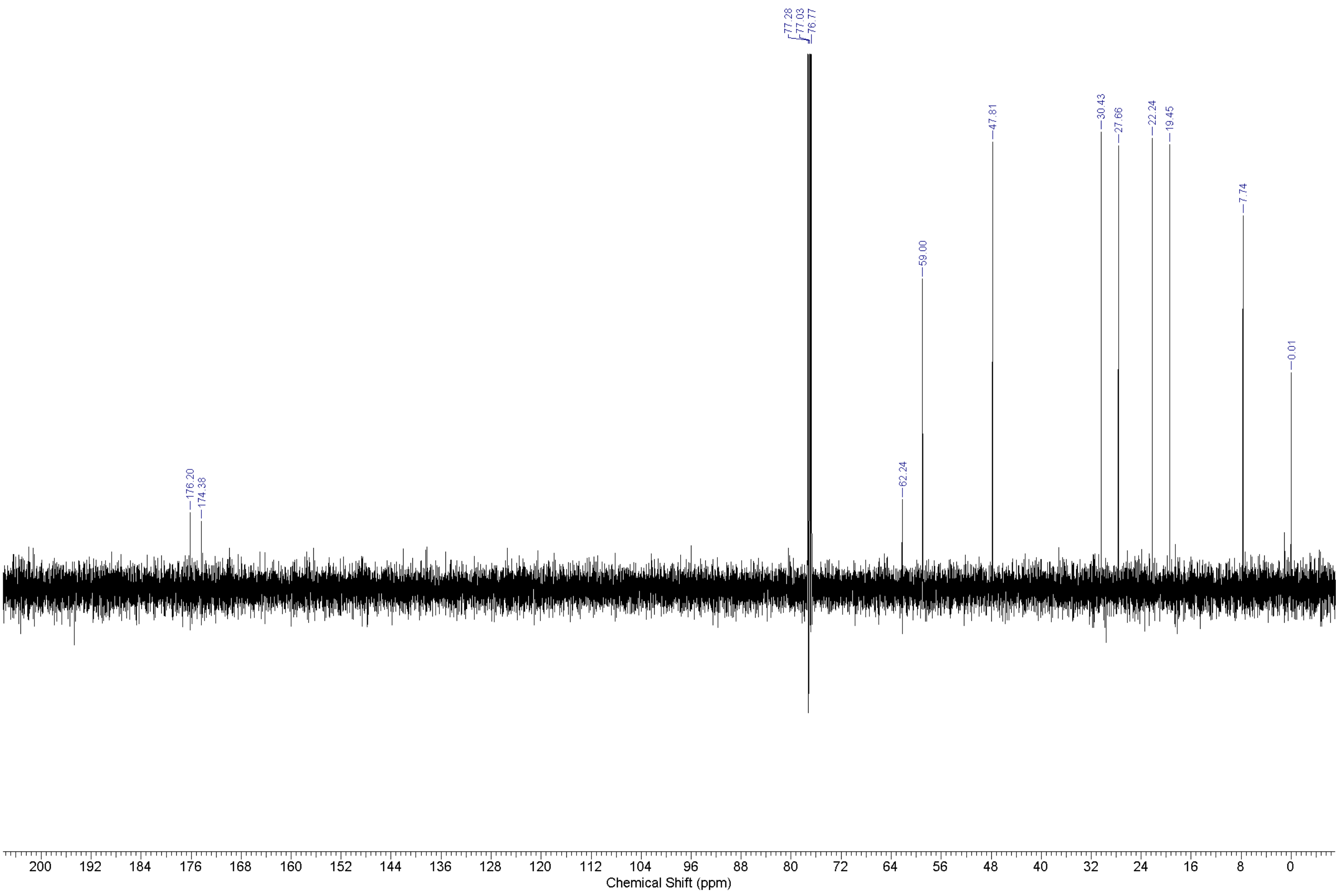
176.20
174.38
77.28
77.03
76.77
62.24
59.00
47.81
30.43
27.66
22.24
19.45
7.74
0.01
Chemical Shift (ppm)

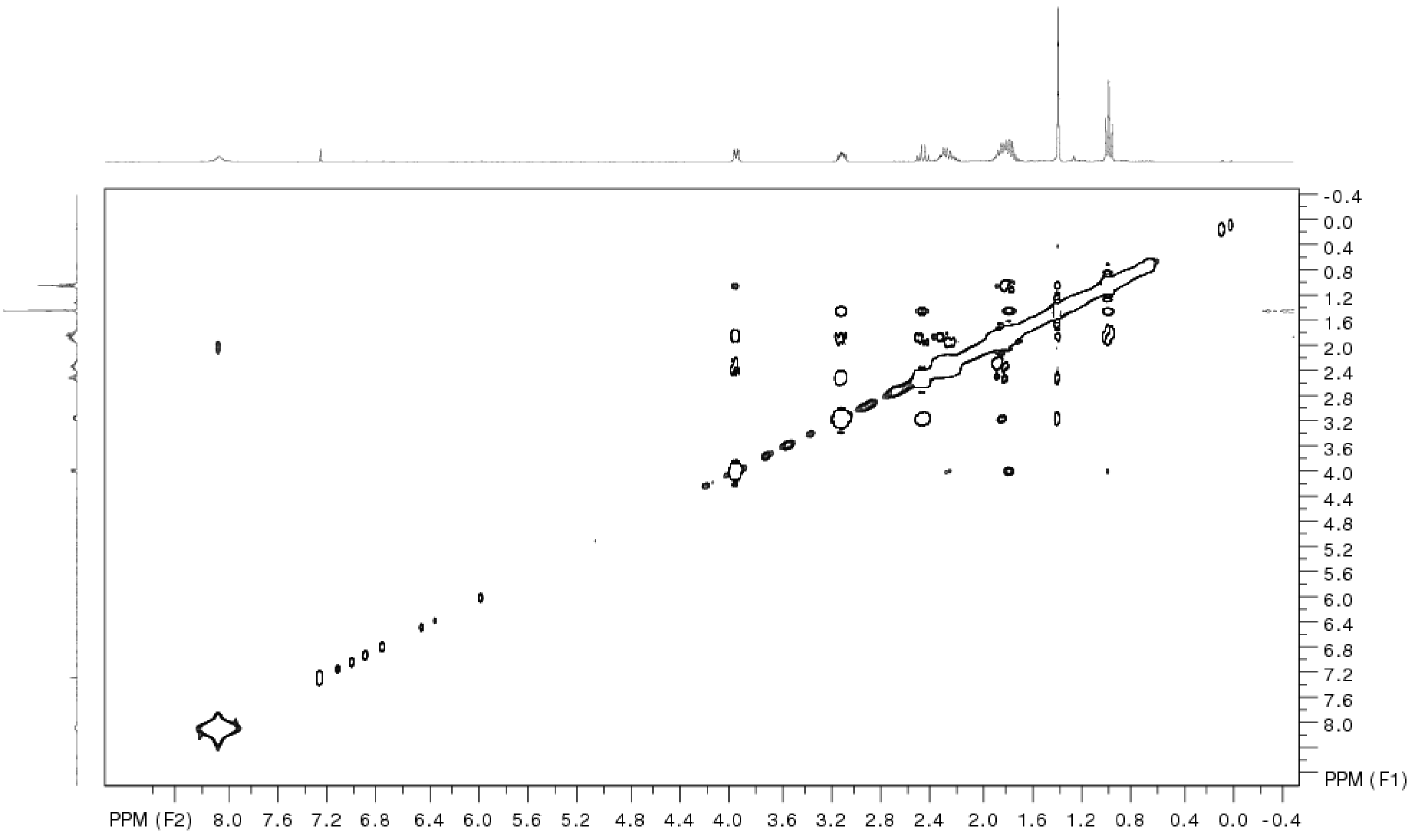
PPM (F2) 8.0 7.6 7.2 6.8 6.4 6.0 5.6 5.2 4.8 4.4 4.0 3.6 3.2 2.8 2.4 2.0 1.6 1.2 0.8 0.4 0.0 -0.4
PPM (F1)
-0.4 0.0 0.4 0.8 1.2 1.6 2.0 2.4 2.8 3.2 3.6 4.0 4.4 4.8 5.2 5.6 6.0 6.4 6.8 7.2 7.6 8.0

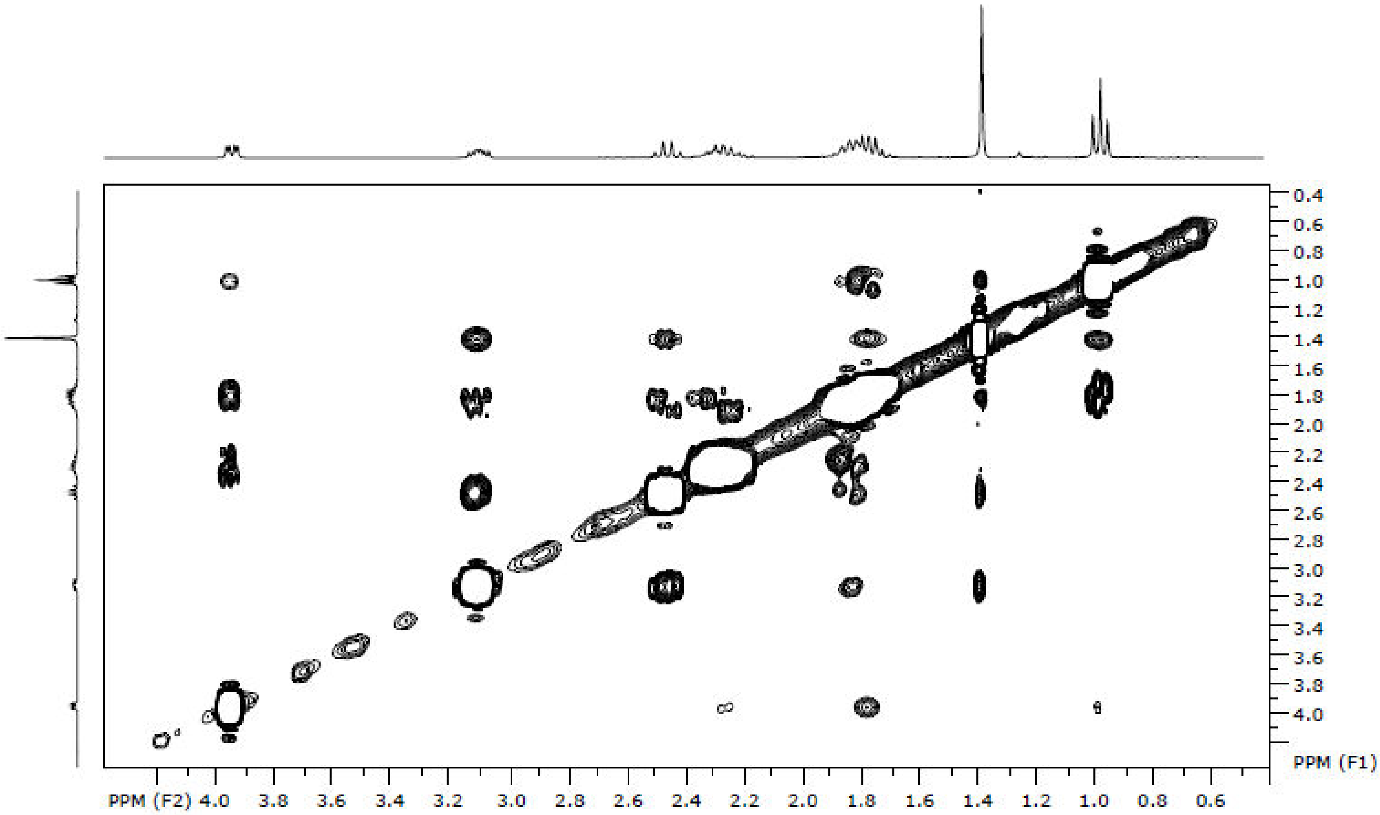
PPM (F2) 4.0 3.8 3.6 3.4 3.2 3.0 2.8 2.6 2.4 2.2 2.0 1.8 1.6 1.4 1.2 1.0 0.8 0.6
PPM (F1)
0.4 0.6 0.8 1.0 1.2 1.4 1.6 1.8 2.0 2.2 2.4 2.6 2.8 3.0 3.2 3.4 3.6 3.8 4.0

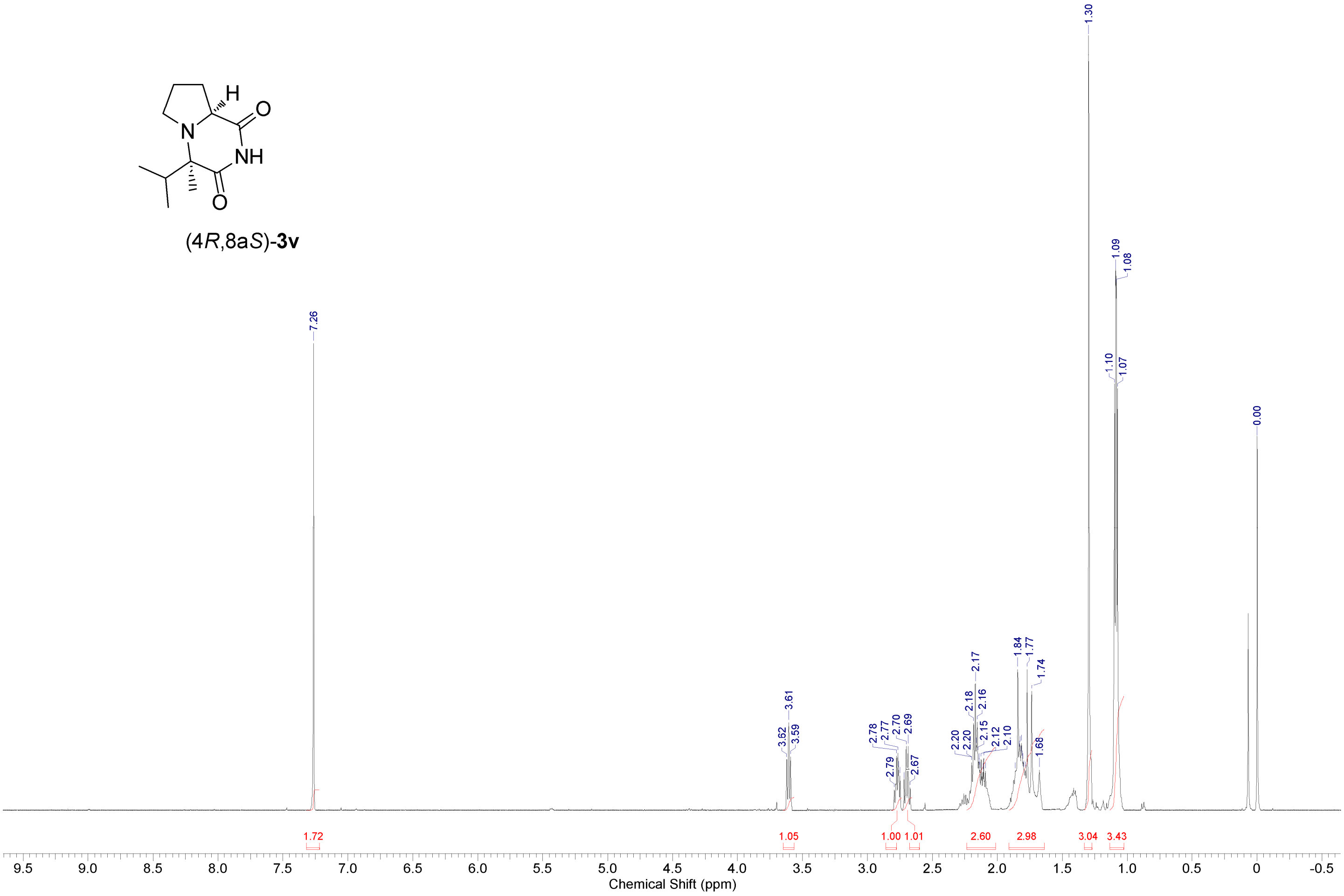
H
O
N
NH
O
7.26
3.62
3.61
3.59
2.79
2.78
2.77
2.70
2.69
2.67
2.20
2.20
2.18
2.17
2.16
2.15
2.12
2.10
1.84
1.77
1.74
1.68
1.30
1.10
1.09
1.08
1.07
0.00
1.72
1.05
1.00
1.01
2.60
2.98
3.04
3.43
Chemical Shift (ppm)

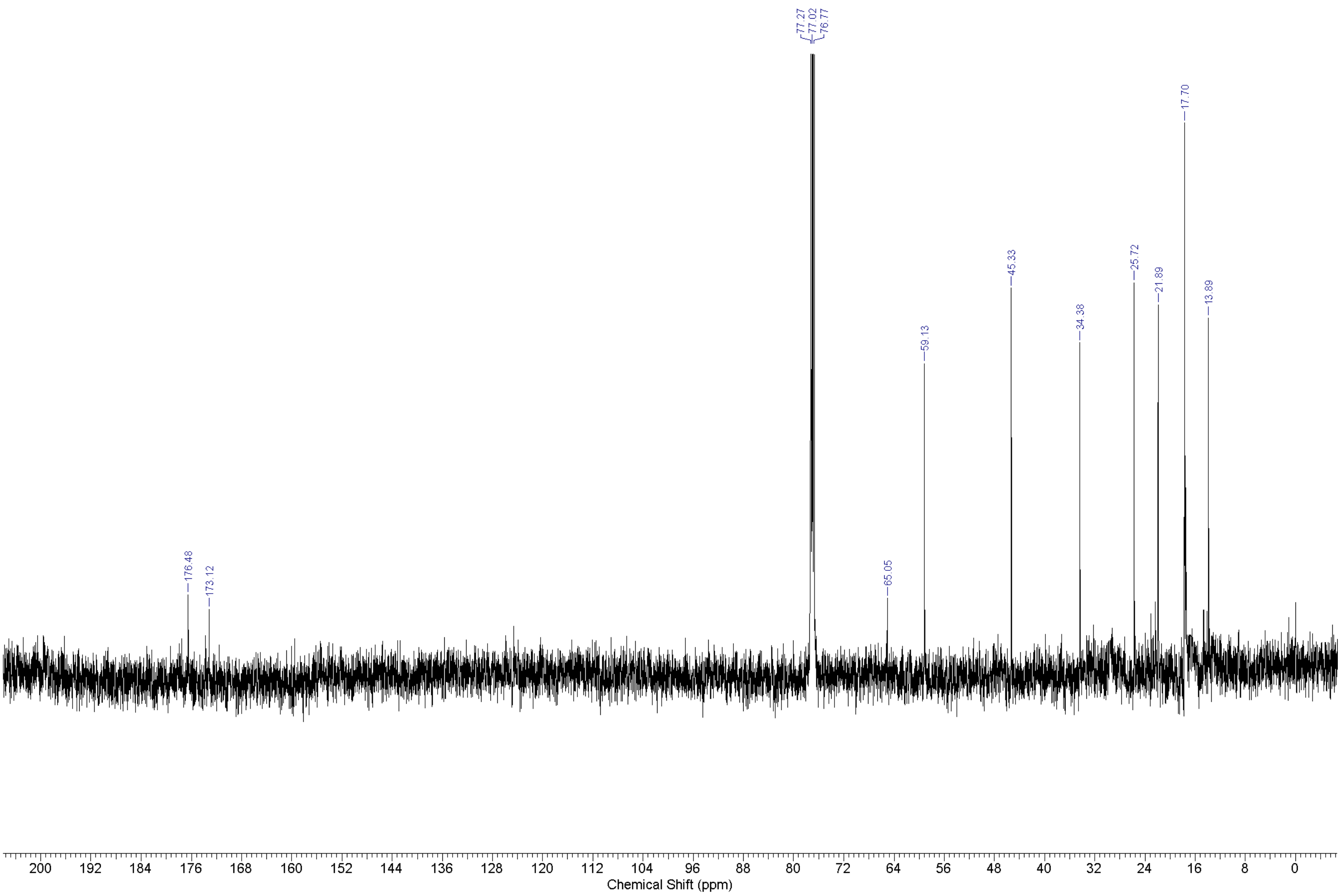
176.48
173.12
77.27
77.02
76.77
65.05
59.13
45.33
34.38
25.72
21.89
17.70
13.89
Chemical Shift (ppm)

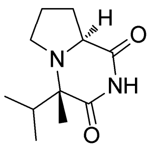
H
O
N
NH
O

(4*S*,8a*S*)-**3v**

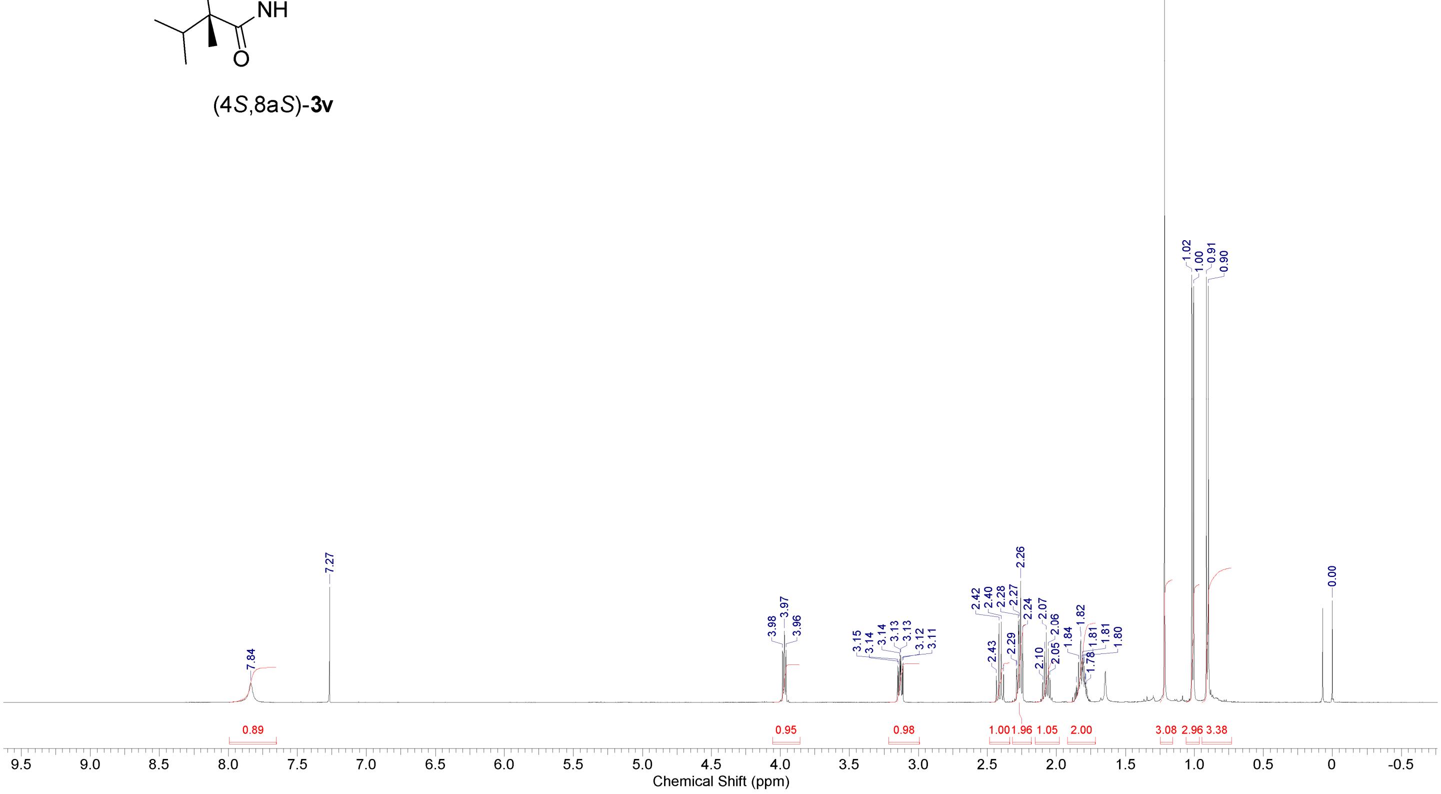
1.22
1.02
1.00
0.91
0.90
7.84
7.27
3.98
3.97
3.96
3.15
3.14
3.14
3.13
3.13
3.12
3.11
2.42
2.40
2.28
2.27
2.26
2.43
2.29
2.24
2.10
2.07
2.06
2.05
1.84
1.82
1.81
1.81
1.80
1.78
0.00
0.89
0.95
0.98
1.00
1.96
1.05
2.00
3.08
2.96
3.38
Chemical Shift (ppm)

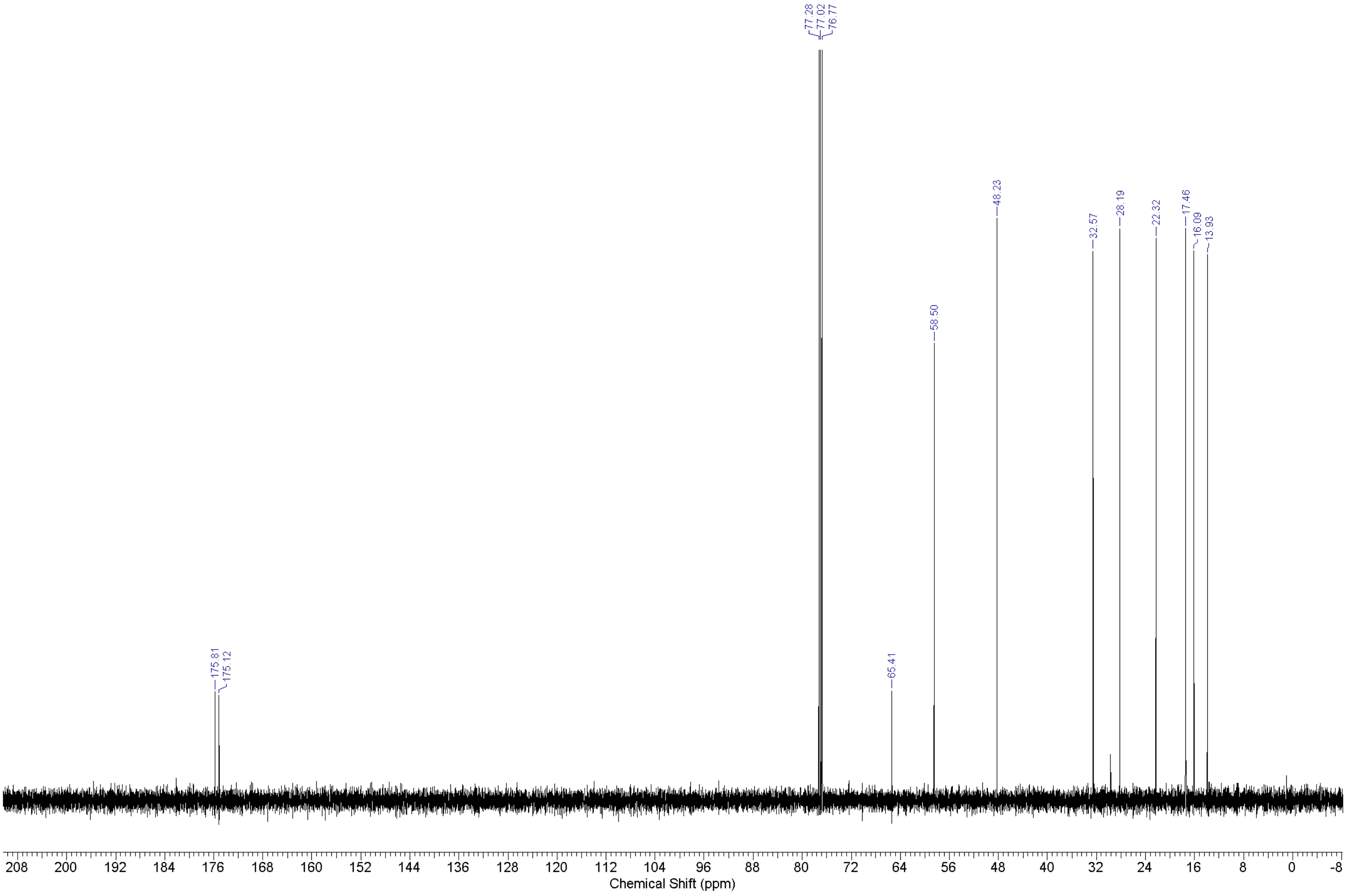
77.28
77.02
76.77
58.50
48.23
32.57
28.19
22.32
17.46
16.09
13.93
175.81
175.12
65.41
Chemical Shift (ppm)

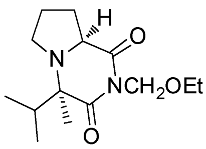

(4*R*,8a*S*)-**3x**

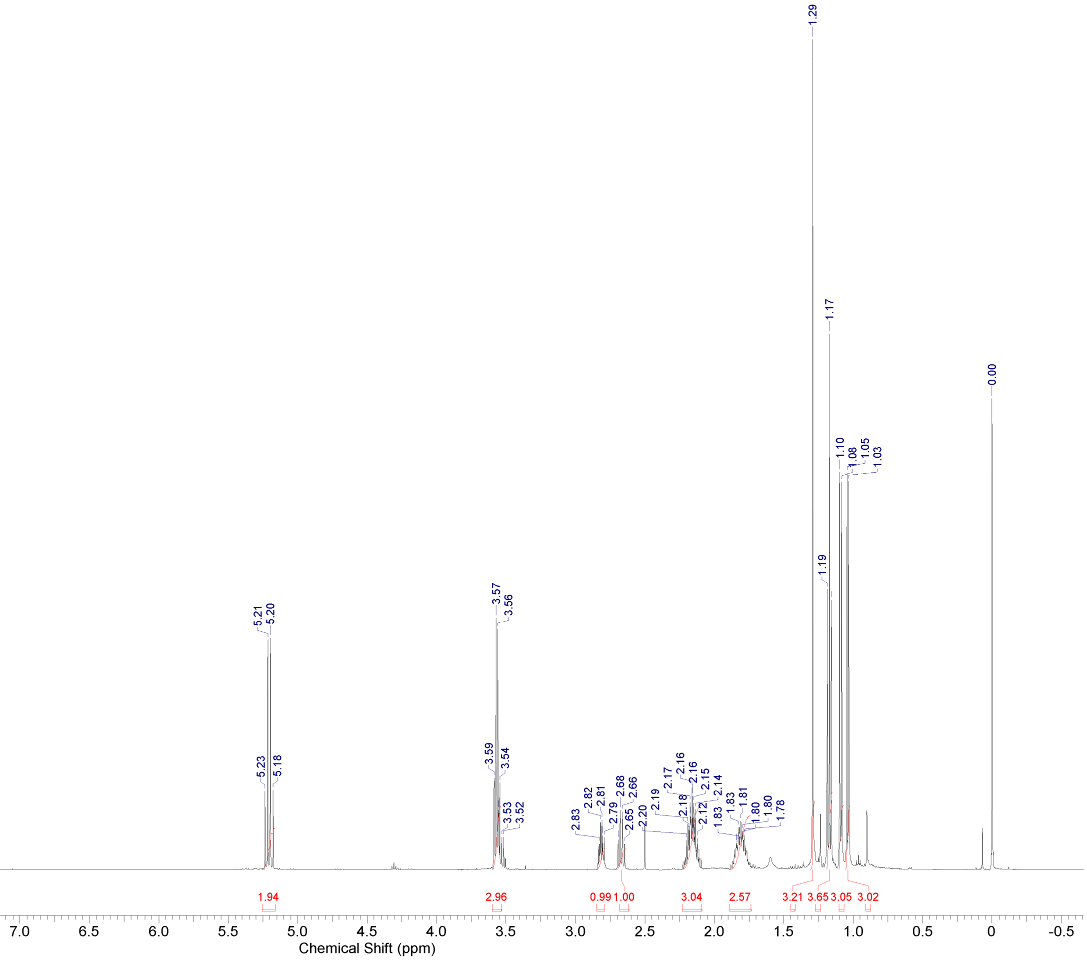

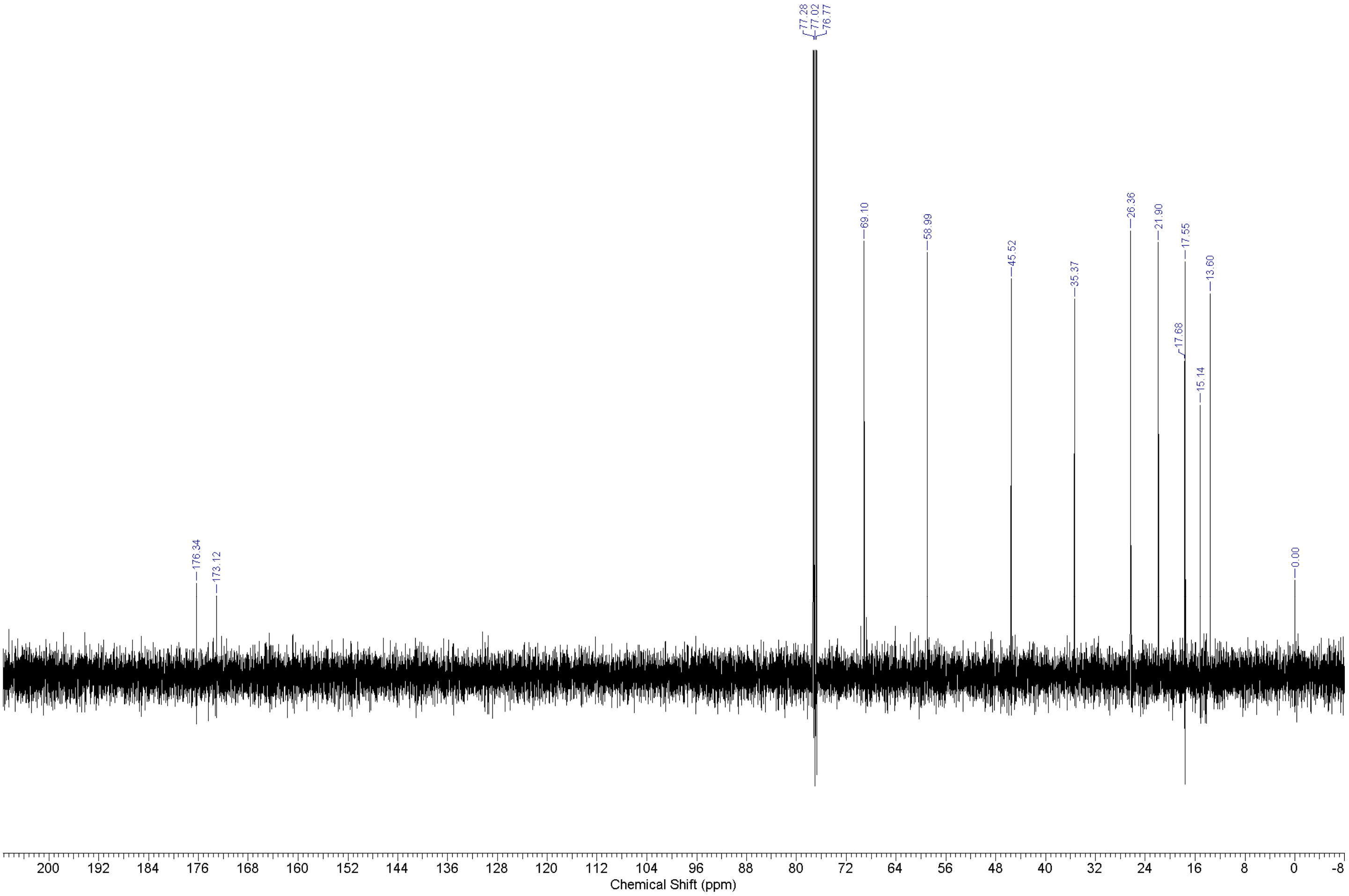

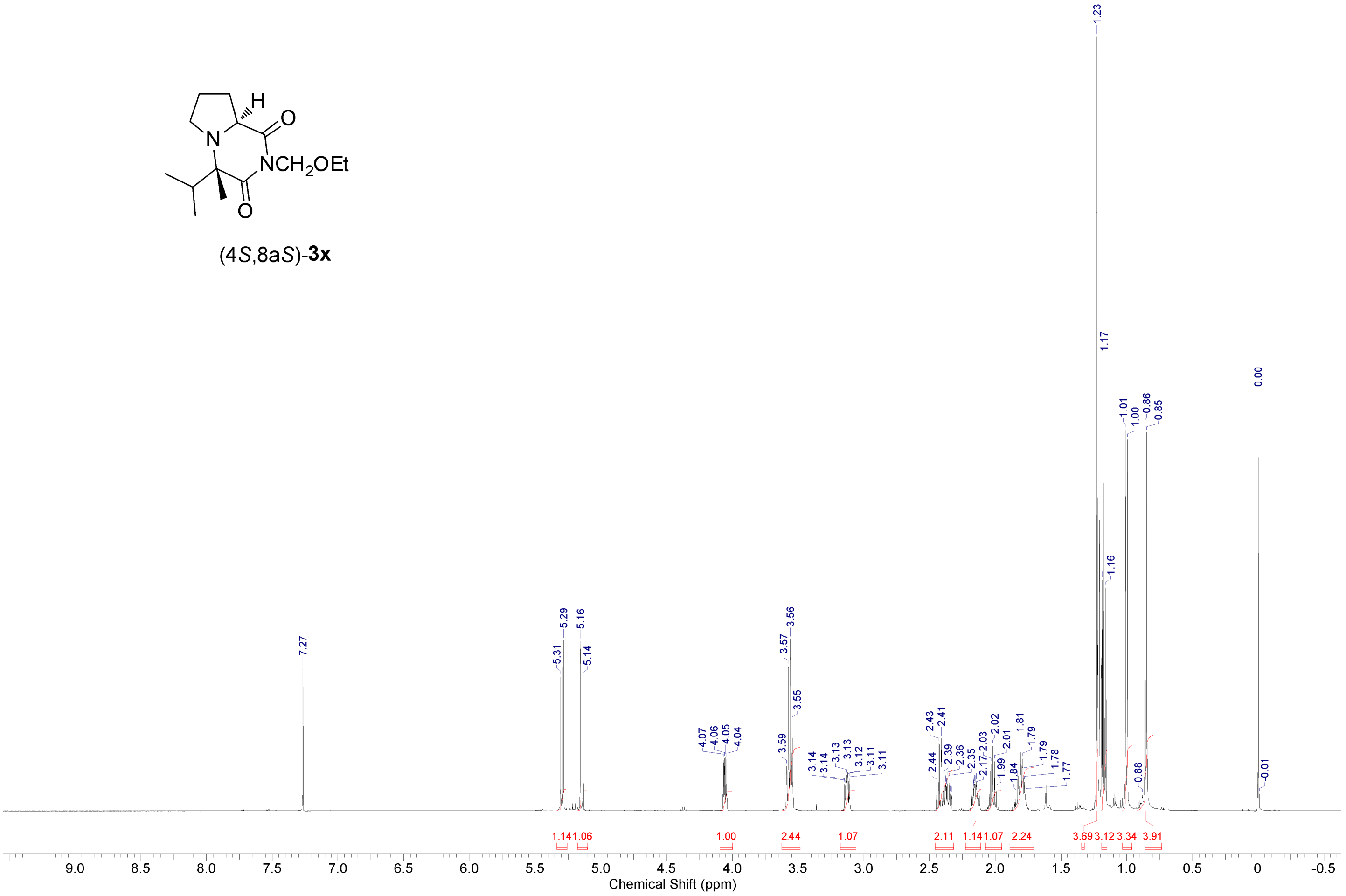
H
O
N
NCH2OEt
O
1.23
1.17
1.16
1.01
1.00
0.86
0.85
0.00
-0.01
7.27
5.31
5.29
5.16
5.14
4.07
4.06
4.05
4.04
3.59
3.57
3.56
3.55
3.14
3.14
3.13
3.13
3.12
3.11
3.11
2.44
2.43
2.41
2.39
2.36
2.35
2.17
2.03
2.02
2.01
1.99
1.84
1.81
1.79
1.78
1.77
0.88
1.14
1.06
1.00
2.44
1.07
2.11
1.14
1.07
2.24
3.69
3.12
3.34
3.91
Chemical Shift (ppm)

(4*S*,8a*S*)-**3x**

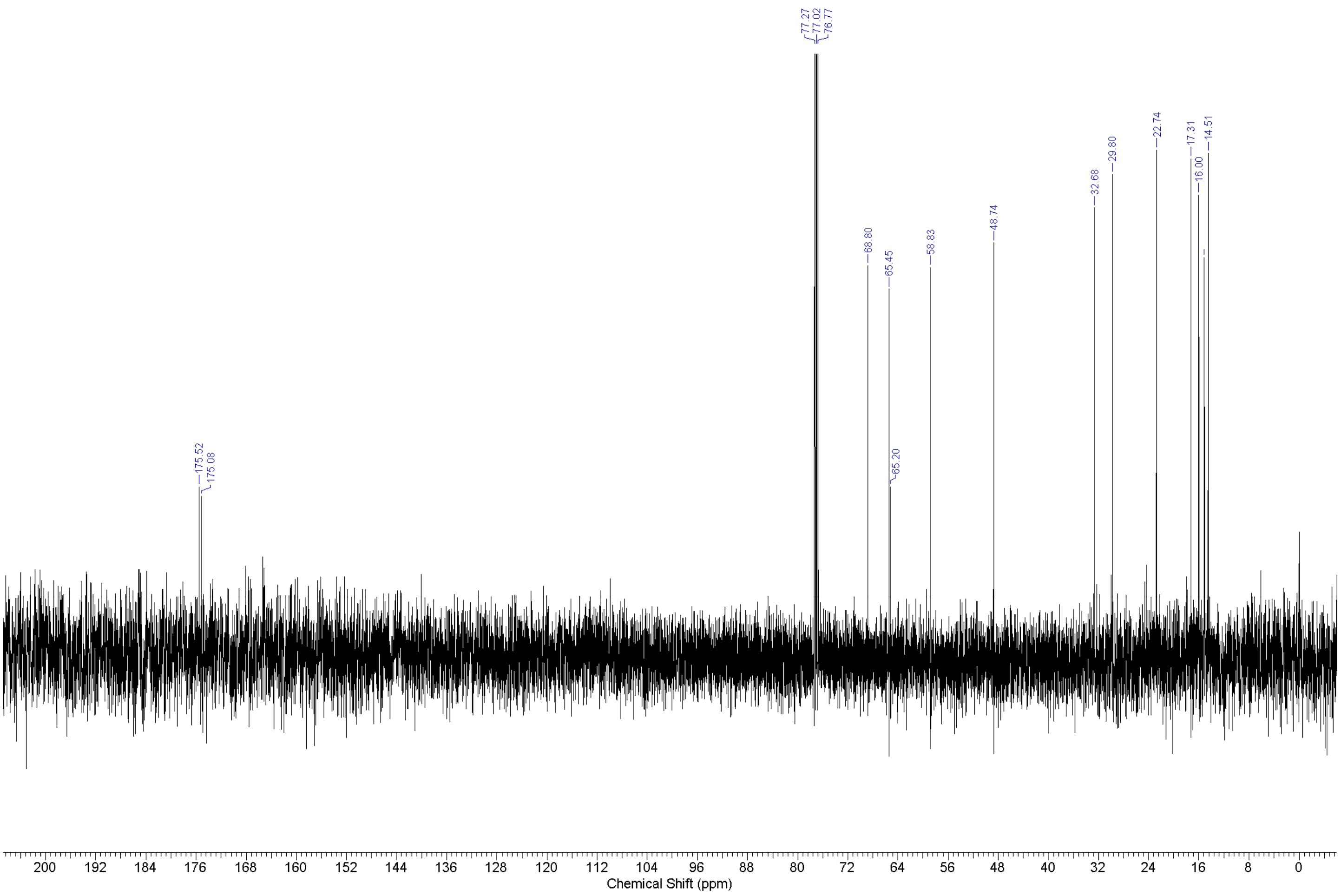
77.27
77.02
76.77
68.80
65.45
65.20
58.83
48.74
32.68
29.80
22.74
17.31
16.00
14.51
175.52
175.08
Chemical Shift (ppm)
